# What is personalized medicine: sharpening a vague term based on a systematic literature review

**DOI:** 10.1186/1472-6939-14-55

**Published:** 2013-12-21

**Authors:** Sebastian Schleidgen, Corinna Klingler, Teresa Bertram, Wolf H Rogowski, Georg Marckmann

**Affiliations:** 1 Ludwig-Maximilians-University Munich, Institute of Ethics, History and Theory of Medicine, Munich, Germany; 2 Helmholtz Zentrum Munich, German Research Center for Environmental Health, Institute of Health Economics and Health Care Management, Neuherberg, Germany; 3 Ludwig-Maximilians-University Munich, Institute and Outpatient Clinic for Occupational, Social and Environmental Medicine, Munich, Germany

**Keywords:** Biomarkers, Conceptual vagueness, Definition, Individualized medicine, Stratification, Timing

## Abstract

**Background:**

Recently, individualized or personalized medicine (PM) has become a buzz word in the academic as well as public debate surrounding health care. However, PM lacks a clear definition and is open to interpretation. This conceptual vagueness complicates public discourse on chances, risks and limits of PM. Furthermore, stakeholders might use it to further their respective interests and preferences. For these reasons it is important to have a shared understanding of PM. In this paper, we present a sufficiently precise as well as adequate definition of PM with the potential of wide acceptance.

**Methods:**

For this purpose, in a first step a systematic literature review was conducted to understand how PM is actually used in scientific practice. PubMed was searched using the keywords “individualized medicine”, “individualised medicine”, “personalized medicine” and “personalised medicine” connected by the Boolean operator OR. A data extraction tabloid was developed putting forward a means/ends-division. Full-texts of articles containing the search terms in title or abstract were screened for definitions. Definitions were extracted; according to the means/ends distinction their elements were assigned to the corresponding category. To reduce complexity of the resulting list, summary categories were developed inductively from the data using thematic analysis. In a second step, six well-known criteria for adequate definitions were applied to these categories to derive a so-called precising definition.

**Results:**

We identified 2457 articles containing the terms PM in title or abstract. Of those 683 contained a definition of PM and were thus included in our review. 1459 ends and 1025 means were found in the definitions. From these we derived the precising definition: PM seeks to improve stratification and timing of health care by utilizing biological information and biomarkers on the level of molecular disease pathways, genetics, proteomics as well as metabolomics.

**Conclusions:**

Our definition includes the aspects that are specific for developments labeled as PM while, on the other hand, recognizing the limits of these developments. Furthermore, it is supported by the quantitative analysis of PM definitions in the literature, which suggests that it it is widely acceptable and thus has the potential to avoid the above mentioned issues.

## Background

In recent years, individualized or personalized medicine (IM/PM)^a^ has become a buzz word in the academic as well as public debate surrounding health care. Promising to make health care more effective and efficient by tailored medical interventions it has become one of the core areas of public research funding and pharmaceutical research investment [1]. However, PM lacks a clear definition and is open to interpretation [2]. Consequently, a whole continuum of PM understandings exists, in which three main positions can be identified: (a) PM is not a new concept as medicine has always been individualized, (b) PM is holistic health care centered around the needs of the individual patient and (c) PM is treatment targeted at stratified subgroups (e.g. pharmacogenetics) [3].

The prevailing vagueness of the term poses several problems. First and foremost, it unduly complicates public discourse on chances, risks and limits of PM: if the meaning of a term like PM is not clearly defined, it is trivially impossible to debate questions of its matter as well as its (future) handling. As a consequence, it is difficult to develop regulatory mechanisms that ensure effectiveness as well as ethical acceptability of research on and provision of PM. Furthermore, stakeholders might utilize the terms’ vagueness to further their respective, especially economic interests and preferences. In the medical context, however, this seems to be morally unacceptable; on the contrary, medical actions ultimately must be directed towards the patients’ needs. Finally, PM’s underspecification may lead to unwarranted fears of patients as well as unfounded hopes like a perfectly tailored or patient-centered medicine [4,5]. Against this background, the goal of this paper is to help structuring the debate over PM’s meaning by developing a *sufficiently* precise definition, which is formally adequate while at the same time reflecting the actual scientific possibilities as well as limitations of medical measures labeled as PM.

A definition declares that a term (the *definiendum*) is equivalent with another set of terms whose meaning is well-established (the *definiens*) [6,7]. Hurley [8] differentiates, among others, between stipulative, lexical and precising definitions. While a lexical definition simply tries to capture the way a word is commonly used, a stipulative definition arbitrarily assigns a meaning to a certain expression, whereas a precising definition tries to reduce the vagueness of a term used in practice: “[a precising definition] differs from a stipulative definition because its definiendum is not a new term, but one whose usage is established, although vague. The makers of a precising definition, therefore, are not free to assign any meaning they choose to the definiendum. They must remain true to established usage as far as that is possible. The aim is to make a known term more precise. At the same time they must go beyond established usage if the vagueness of the definiendum is to be reduced” [9].

As the term PM is already established in public discourse, however vaguely defined, the goal of this paper can only be to derive a precising definition. For this purpose, we first have to describe the current usage of the term. As one central goal of our paper consists in clarifying the actual possibilities and limitations of PM, we decided to analyse its current usage in the sciences. This decision followed the assumption that the scientific debate on PM centers on its actual state-of-the-art. Analyzing the stakeholders’ discourse, on the other hand, would potentially have captured the respective hopes and interests instead of the actual development labeled as PM. Accordingly, we conducted a systematic review of definitions appearing in the academic literature. Based on those findings, in a second step, we developed a precising definition of PM which is formally adequate as well as sufficiently precise and hence can be regarded as an adequate basis for public discourse on PM.

## Methods

For our systematic review, PubMed was searched using the keywords “individualized medicine”, “individualised medicine”, “personalized medicine” and “personalised medicine” connected by the Boolean operator OR. We refrained from including MeSH-terms in the search strategy as the term “individualized medicine” specified in the thesaurus of PubMed contains a specific definition, namely “a therapeutic approach tailoring therapy for genetically defined subgroups of patients”. Including MeSH-terms would therefore have pre-selected certain articles according to this understanding of PM. Excluding MeSH-terms, on the contrary, allowed us to stay open to alternative understandings of PM. We only searched titles and abstracts to identify those articles in which PM is the main focus. We assumed that those articles were more likely to contain a definition of PM. We did not restrict the date of publication; our last research was conducted on August 15, 2012. Furthermore, we included only articles written in English as our goal was to capture the international debate. Subsequently, we checked full-text availability of the articles identified via the Bavarian State Library, which provides access to one of the most comprehensive online journal collections as well as print media in Germany. Where full-texts were not available, we contacted the authors given contact details were provided.

A data extraction tabloid was developed which puts forward a means/ends-division. This decision is based on the assumption that medical interventions are defined by the means they employ to reach certain ends. A stethoscope, for instance, is not defined by its form or color or technical details – they may vary –, but rather that it allows listening to internal sounds (means) to detect pathologies of the lung, heart or abdomen (ends).

Articles available were screened for definitions by SS and TB, definitions were extracted using the extraction tabloid by TB. This resulted in a list of ends and means constitutive for PM. For instance, from the definition “The purpose of personalized medicine is to identify the optimal treatment for each individual patient to maximize treatment benefit and minimize adverse effects. To achieve this goal, informative biomarkers need to be identified to stratify patients for specific therapies” [10] we derived the ends:

(a) to identify the optimal treatment for each individual patient;

(b) to maximize treatment benefit;

(c) to minimize adverse effects

as well as the means:

(a) identification of informative biomarkers

(b) stratification of patients.

We did not interpret the data, but followed the authors’ understanding of what constitutes ends and means. To reduce complexity of the resulting list, summary categories were developed inductively from the data using thematic analysis adapted to the research aim [11]. Two researchers, SS and CK, independently derived categories from the data. In case their assessments differed, discrepancies were discussed and resolved consensually (thereby ensuring inter-coder reliability). Where technical details were not clear, a medical expert was consulted for clarification.

## Results

**Figure 1:**
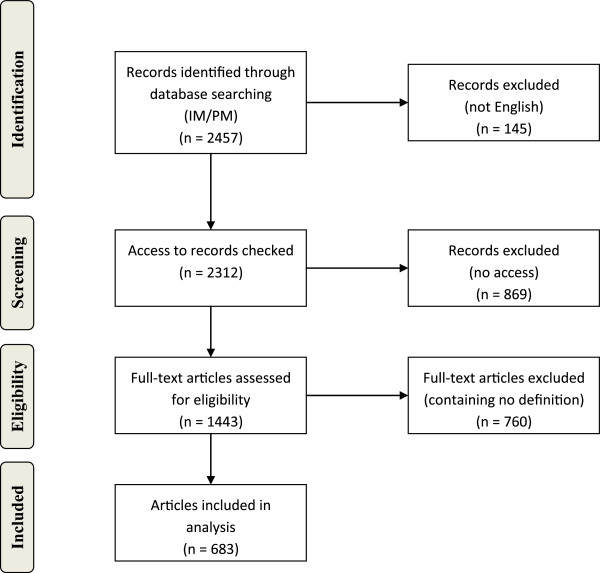
**Flow diagram of data collection process according to the PRISMA statement **[12]**.**

All in all, we identified 2457 articles containing the terms PM in title or abstract. 145 articles were not written in English and therefore excluded from further analysis. Full-texts were available for 1443 papers. Of those articles 683 contained a definition of PM and were thus included in our review (see also Figure 1).^b^

**Figure 2:**
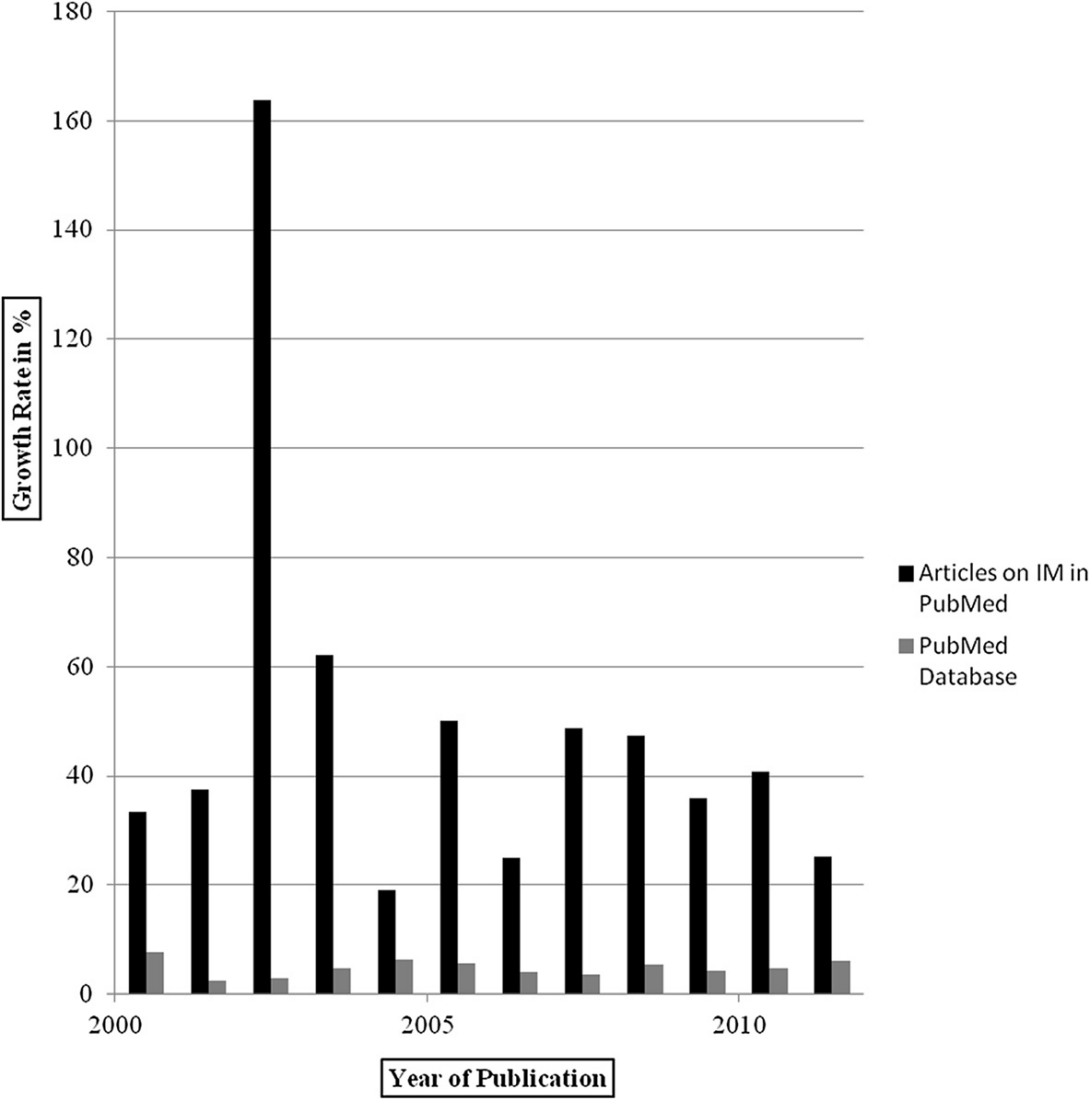
Annual growth rates of publications on PM in PubMed (2000-2011).

In our sample, PM was first mentioned in 1971, but the discourse on PM did not significantly intensify before 1999/2000. Figure 2 provides an overview of the annual growth rates of publications on PM in the period of 2000-2011 (we leave out 2012 because we only included articles published before August 15^th^): the average growth rate of literature written on PM was 49% per year. This is significantly higher than the annual increment of PubMed’s database in the period of 2000-2011, whose average growth rate was 4.8%. The growth rate of over 160% in 2002 might be most reasonably understood in the context of the completion of the human genome project and the resulting hopes in gene-based medicine.

**Figure 3:**
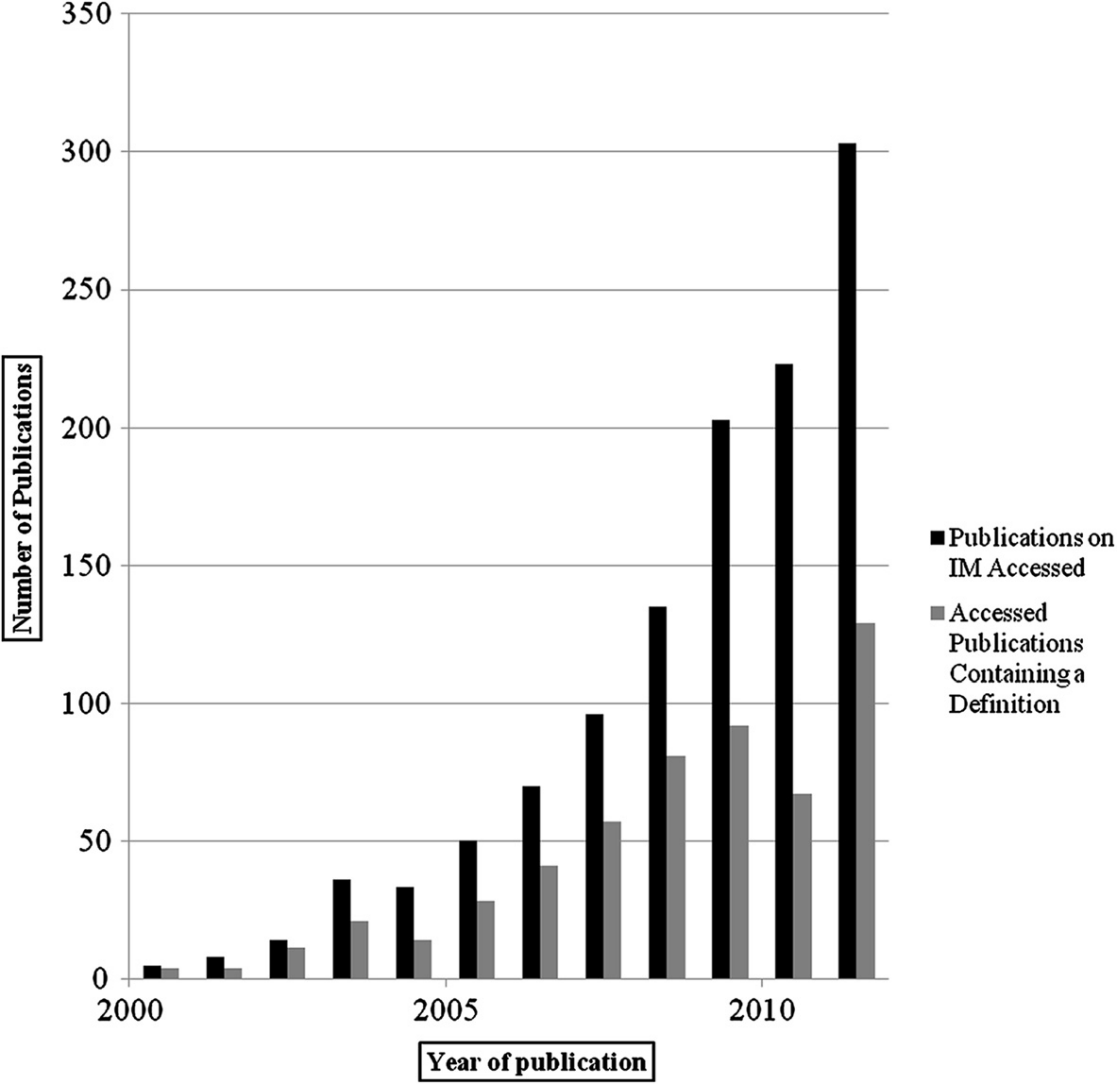
Development of publications on PM in PubMed (2000-2011).

Until 2009, each year an average of 60.4% of publications on PM included a definition. That might indicate awareness among scientists that the term still lacks a shared definition (Figure 3). For the same reason, however, it is problematic that almost half of the authors did not explicate their understanding of PM, thereby purporting a common understanding. Additionally, in the last three years an average of only 39.3% of publications included a definition which might suggest a decrease in awareness of the terms’ prevailing vagueness. However, more information is needed to substantiate this assumption.

Furthermore, the data proves that there is no consensus on the meaning of PM. After extracting and analyzing the definitions of PM, we found 1459 different ends and 1025 different means. A shortened version of the resulting category system is depicted in Tables 1 and 2 (see Additional file 1 for the complete tables containing the original definitions’ components).

**Table 1:** Ends of PM in the literature (short)

**Ends in (1459)**	
**1 Research (156)**	**C. Therapy (975)**
**A. Basic research (43)**	***Diagnosis (68)***
***On causes and processes of diseases (9)***	unspecified (18)
***On risk factors for diseases (3)***	tailored diagnosis/diagnostics (28)
***On disease classification (18)***	improved diagnosis/diagnostic measures (22)
***To further the development of new treatment measures (13)***	*unspecified (10)*
	*effectiveness/efficacy (5)*
**B. Research on new diagnostic and prognostic/predictive measures (30)**	*timing (6)*
	*tailored diagnostics (1)*
***Unspecified (10)***	***Prognosis/Prediction (158)***
***Regarding tailoring (2)***	unspecified (4)
***Regarding stratification (4)***	prediction of disease progression/recurrence (5)
***Regarding improved diagnostic/prognostic/ predictive measures (9)***	predictive information guiding clinical decision making (21)
unspecified (2)	prediction of treatment effects/effectiveness (60)
effectiveness (6)	
efficiency (1)	tailoring prognosis/prediction (39)
***Regarding companion diagnostics (5)***	stratification by predicted treatment effects (25)
**C. Research on new therapeutic measures (83)**	improvement of prognostic/predictive measures (4)
***Unspecified (15)***	*unspecified (2)*
***Regarding tailoring (15)***	*safety (1)*
***Regarding stratification (40)***	*effectiveness/efficacy (1)*
***Regarding improved treatment measures (13)***	***Treatment (749)***
unspecified (5)	unspecified (27)
safety (3)	choice of therapeutic measure (104)
effectiveness (3)	regarding treatment monitoring (12)
efficiency (2)	effectiveness/efficacy of treatment (16)
	efficiency of treatment (2)
**2 Drug approval (14)**	safety of treatment (6)
**A. Improved validation processes (1)**	treatment outcomes (2)
**B. Improved clinical trials (11)**	tailoring therapy/therapeutic measures (269)
***Unspecified (4)***	
***Efficiency (7)***	stratified therapeutic measure (18)
**C. Improved approval processes (2)**	improvement of therapy/therapeutic measures (293)

	*unspecified (41)*
**3 Health care (1202)**	*choice of treatment (32)*
**A. Health care in general (69)**	*effectiveness/efficacy (92)*
***Unspecified (1)***	*efficiency (2)*
***Decision making in health care (3)***	*timing (4)*
***Tailoring health care (37)***	*safety (83)*
***Improved health care (28)***	*tailoring treatment measures (39)*
unspecified (17)	
effectiveness/efficacy (1)	**4 Improved health (10)**
efficiency (2)	
safety (2)	**5 Further ends (77)**
choices in health care (1)	***Unspecified (11)***
tailoring health care (5)	***(Tailored) patient management (3)***
**B. Prevention (158)**	***Stratification (1)***
***Risk prognosis/prediction (82)***	***Reduce/control costs (30)***
unspecified (52)	***Improved effectiveness/efficacy (24)***
at the individual level (26)	***Improved timing (1)***
at the population level (3)	***Improved safety (3)***
improved risk prognosis/prediction (1)	***Improved quality of life (4)***
***Primary prevention (76)***	
unspecified (34)	
tailoring preventive measures (22)	
stratified preventive measures (1)	
improved prevention/preventive strategies (19)	
*unspecified (16)*	
*effectiveness (1)*	
*timing (2)*	

**Table 2:** Means of PM in the literature (short)

**Means in (1025)**	
**1 Research (97)**	*genetic information (417)*
**A. On individual differences (4)**	unspecified (19)
**B. On genetics/genomics (39)**	regarding knowledge of genes/genetic variation (215)
***Unspecified (11)***	regarding gene expression (13)
***Regarding the influence of genes on disease development/progression (5)***	regarding genomics/genomic variation (98)
***Regarding the influence of genes on drug response (22)***	regarding the transcriptome (4)
	regarding DNA/RNA(6)
***Regarding the interaction of genes with other factors that influence disease development/progression (1)***	regarding epigenetics (8)
	regarding gene-environment interaction (4)
**C. On further factors that influence disease progression/development (8)**	regarding pharmacogenetics/pharmacogenomics (41)
**D. On further factors that influence drug response (4)**	regarding further points (9)
	*information on proteomics (35)*
**E. On biomarkers (40)**	*information on metabolomics (14)*
**F. On new technologies (2)**	***Further points mentioned (6)***
	**B. Usage of environmental factors/information (33)**
**2 Application in patient care (928)**	**C. Usage of further individual factors/information (126)**
**A. Usage of clinical information/clinical indicators (718)**	***Unspecified (77)***
	***Age-related information (4)***
***Unspecified (11)***	***Phenotypic factors/information (19)***
***Using information on medical history (12)***	unspecified (5)
unspecified (6)	referring to gender (4)
regarding family history (6)	referring to weight (3)
***Using biological information/biomarkers (689)***	referring to membership of a certain (ethnic) group (7)
unspecified (81)	
of the individual (21)	***Personal preferences (4)***
of the disease (13)	***Behavior (22)***
on the molecular level (574)	unspecified (8)
*unspecified (101)*	referring to nutrition (5)
*information on disease pathways (7)*	referring to lifestyle (6)
	referring to toxins (3)
	**D. Usage of (specific) technology (51)**
	***Unspecified (17)***
	***(New) technology for genetic analysis (30)***
	***Information technology (4)***

It is important to note that the term PM is apparently used across different spheres of the healthcare system like patient care, research and even drug approval. What is also interesting is the huge diversity of definitional elements that we found ranging from “tailored wellness plan for individuals” to “facilitate validation of health care products”.

Nevertheless, the debate seems to focus on certain aspects. Regarding the ends of PM, the dominant theme is (improving) the treatment of patients. More than half of the ends we found in the literature concern therapeutic interventions and/or their improvement on different dimensions like safety, efficiency and effectiveness. More specifically, the discourse seems to focus on tailoring therapeutic interventions to a patient’s specific characteristics: about 25% of all ends collected refer to (improved) tailoring of therapeutic interventions. Regarding the means, the possibilities of molecular or more specifically genetic analysis can be identified as the key aspect of PM: approximately 50% of all means identified refer to genetics and genetic information.

## Discussion

### Six criteria of adequate definitions

As stated above, a precising definition intends to reduce the vagueness of terms *used in practice*. Hence, we have to start with the empirical information about the scientific usage of PM and search for a combination of ends and means which satisfies certain criteria. This raises the problem of adequately balancing the two main demands of a precising definition: staying close to the actual usage of a term while at the same time focusing its meaning for the sake of precision. One possible way to approach this problem would be to include definitional elements only if they occur with a certain frequency in the literature. This, however, requires establishing thresholds above which an element is to be included in our definition. As such thresholds necessarily are arbitrary, we chose an analytical approach instead. Accordingly, we used six criteria of adequate definitions which allowed us to filter the various components encountered in the literature. These criteria provide an analytical way to deal adequately with the two demands: sticking to the established usage of PM and reducing its vagueness. The criteria are:

1. A definition must be necessary, i.e. there must not exist any well-established term equivalent with its *definiens*,

2. a definition must be neither too broad nor too narrow, i.e. it must be adequately distinctive (For instance, the definition “antibiotics are drugs used to treat infectious diseases“ is too broad as antifungal agents – amongst others – are also used to treat infectious diseases. The definition “antibiotics are drugs used to treat streptococcal infections”, on the other hand, is too narrow as other bacterial infections can also be treated with antibiotics),

3. a definition must not be circular, i.e. the *definiendum* must not appear in the *definiens*,

4. a definition must not be redundant, i.e. it must not contain any components which are implied by any other of its components,

5. a definition must not be inconsistent, i.e. it must not include any logical contradictions, and

6. a definition must not be ambiguous, i.e. the *definiens* must be clearly described [8,9].

Obviously, many definitions we found in the literature do not meet these criteria. For instance, most of the mentioned ends and means are far too broad to specify PM adequately: defining PM as pursuing the *end* of “treating diseases” is not adequately distinctive (criterion 2), as any therapeutic measures’ end consists in treating diseases. The same holds for a definition of PM that refers to the *means* of “using clinical information”.

### Adequate ends of personalized medicine

When taking a closer look at the ends of PM encountered in the literature (Table 1), the first main category listed is research. Basic research as well as “research” on new diagnostic and therapeutic measures is to be excluded from the ends of PM as it violates criterion 5: in the medical context,^c^ research is always a means to a given end, but never an end in itself. Usually – although not always – the end consists in improving medical measures or to generate medical knowledge. Therefore it would not be consistent to include research as an end in a definition of PM.

The second main category is “drug approval” which neither is an adequate end for defining PM. After all, we search for a definition of individualized/personalized *medicine*. Accordingly, medicine is the *genus* of PM that denotes a main category to which a certain term belongs and which thereby specifies the limits of a term’s meaning. For instance, “hammer” belongs to the genus “tools” whereas “rose” belongs to the genus “flowers”. The genus “medicine”, however, does not imply the term “drug approval”. Rather, drug approval belongs to the genus “health policy”. Consequently, including drug approval in a definition of PM violates criterion 2 as it would imply an understanding of PM that transcends the *genus* “medicine”.

The category “further ends” is also inadequate for defining PM as the terms listed here are either too broad or too narrow. For instance, the ends of “decision making” or “reduce/control costs” do not even (necessarily) belong to the genus “medicine”, but rather to the genus “human action” or “business administration”, respectively.^d^ As such they are far too broad to define PM adequately. Somewhat narrower, yet still too broad are ends like “patient management” or “use stratification”: it seems to be an end of almost every medical intervention to manage patients and use stratification. On the other hand, some of the findings categorized as further ends like “tailored wellness plan for individuals” are too narrow to define PM adequately: medicine necessarily transcends wellness as it ultimately refers to the health of patients while the term wellness targets only one aspect of health (if it counts as health related at all). For many diseases, increases in wellness will not result in improvements in *health*.

The remaining categories “health care”^e^ and “improved health” are plausible candidates for inclusion. Before taking a closer look at their subcategories, we can generally exclude the subcategories “unspecified” from a definition of PM. To cut the argument short: all ends contained here are too broad to define PM adequately. Having said this, we will first turn to the subcategories “prevention” and “therapy”. To explain the argumentation underlying our decisions for exclusion, we have to take a closer look at the criterion of necessity (1): we assume that a new term becomes necessary only if changes occur or new discoveries are made that cannot be described by an already well-established term (for example, it was necessary to introduce the term “laptop” because the established term “personal computer” did not imply the mobility that the new device exhibits). Consequently, in the medical context, a new term is necessary if the ends and means of medical interventions change in a way that is not captured by any well-established term. Accordingly, the question is whether any of the subcategories of “prevention” and “treatment” necessitate the introduction of a new term. We argue that only categories speaking of *improvements* fulfill the necessity criterion. The other subcategories, e.g. “risk prognosis/prediction” or “primary prevention”, are already well-established terms which do not justify introducing a novel term. This holds for both the subcategories of “prevention” and of “therapy”. Accordingly, we excluded all subcategories except for the ones that refer to an improvement.

Across the categories included thus far we find subcategories that describe different dimensions of improvement: “effectiveness/efficacy”, “timing”, “tailoring”, “choice of treatment”, “efficiency”, and “safety”. Some of these specifications can be regarded as inadequate ends for defining PM because they violate the criterion of non-redundancy (4). In health care we are confronted with a hierarchy of ends. Certain ends (e.g. better timing of treatment) are not pursued for their own sake, but rather for the sake of certain higher-order ends (e.g. more effective treatment). To put it differently: effectiveness, efficiency and safety of an intervention are the dimensions that define improvements in healthcare. For instance, a medication is considered better as an alternative medication if it either results in a more effective treatment measured on some health-related indicator, has less side effects (safety) or is more efficient (i.e. costs less while producing the same health effect or has a bigger effect at the same costs). Improvements of treatment choices as well as of timing and tailoring always imply improvements on one or all of the dimensions “effectiveness”, “efficiency” and “safety”. Of course, improving treatment choices, tailoring and timing does not necessarily reach all of those higher-order ends. Nevertheless, they are the ultimate ends of any improvement in health care. Therefore, “effectiveness”, “efficiency” and “safety” are to be understood as a triad of ultimate ends of health care innovations that are implied in any adequate lower-order end. In accordance with criterion 4 we therefore excluded improvements in “effectiveness”, “efficiency” and “safety” from our definition as they are necessarily implied by any medical improvement.

Additionally, improvements in choices of treatment are themselves implied by improved tailoring of treatment. To be able to choose a treatment – or the *right* treatment, respectively – one needs to tailor the treatment to the specific (sub-)type of disease. An improvement in tailoring diagnostic, prognostic, or treatment measures therefore necessarily leads to an improvement in treatment choices which is therefore redundant and can be excluded. Left are improvements of tailoring and timing of preventive and therapeutic measures as ends of PM. Those ends, however, imply improvements in *health* as ultimate end of any improvement in *health care*. The category “improved health” can therefore be excluded from our definition in accordance with the criterion of non-redundancy (4) following the same line of argument (improved health as ultimate end is implied by improvements in tailoring and timing of prevention and therapy).

Consequently, only “improvement in tailoring and timing of health care” are plausible ends of PM. They satisfy criterion 4 and can be considered adequately distinctive (2), are neither circular (3) nor inconsistent (5) nor ambiguous (6).

### Adequate means of personalized medicine

However, we did not show so far that criterion 1 is ultimately satisfied: an improvement *could* potentially render a new term necessary, but it does not necessarily. Ultimately, many changes introduced to the health care system aim at improving timing and tailoring of prevention or therapy. However, not all of them require the introduction of a new term (e.g. new screening interventions to improve timing of cancer treatment). However, as stated above, a new term becomes necessary if the ends *and* the means of medical measures change in a way that is not captured by any well-established term. Consequently, we have to examine whether we can find means mentioned in the literature which satisfy criteria 1-6 and thereby specify the ends in a way that criterion 1 is satisfied.

First, the subcategories “unspecified” are excluded based on a similar argument as brought forward above: all means contained here are too broad to define PM adequately. Furthermore, only the means “utilizing biological information and biomarkers on the level of molecular disease pathways, genetics, proteomics as well as metabolomics” satisfy criterion 2. All other means in the category “application in the healthcare system” – excluding the category “usage of (specific) technology”^f^ – are not adequately distinctive as they are means employed in any medical measure (e.g. questions concerning gender and weight should be part of any routine anamnesis). Those means – usage of information on the genetic, proteomic, metabolomic and molecular pathway level – are adequately distinctive and can, furthermore, be considered new in the sense requested by criterion 1: they indicate the necessity of a novel term that emphasizes their use in contrast to using standard information like medical history or other non-genetic biomarkers (e.g. blood pressure). One could criticize that we call the use of genetic information new and adequately distinctive as it has long been informing medical decision-making, especially in the context of prenatal diagnostics. However, recent technological developments have rendered it possible to extend the informational base to genomic information, epigenetic information or pharmacogenetic information and also further information on the influence of single genes. The genetic information relevant in our context is of a new quality. We therefore did not exclude genetic information on the basis of criteria 1 and 2. It furthermore satisfies criteria 3-6.

### Combining ends and means: an adequate definition of personalized medicine

Accordingly, we can now derive an adequate precising definition of PM:

PM seeks to improve tailoring and timing of preventive and therapeutic measures by utilizing biological information and biomarkers on the level of molecular disease pathways, genetics, proteomics as well as metabolomics.

Although it is not clear whether improvement of tailoring and timing as ends satisfies criterion 1, tailoring and timing based on genetic/proteomic/metabolomic/molecular pathway-related information is conceptually new and therefore justifies a new term. Moreover, the definition satisfies criteria 2-6.

Several further remarks are important: First, it might not be clear why we have not included “research” as a means in our definition. As stated above, in the medical context, research is always a means for a given end where the end usually consists in improving medical measures or possibilities. In analogy to the argumentation concerning higher and lower-order ends, whenever medical measures, e.g. diagnostic tools, are applied, there usage is necessarily grounded on research. Hence, research is implied in any medical intervention or means – in the case of PM the use of information on molecular pathways, genetics, proteomics and metabolomics – and therefore has to be excluded from our definition in order to satisfy criterion 4.

Second, a similar point applies to the subcategory “usage of (specific) technology”: like any medical intervention, measures considered to be personalized utilize certain (specific) technologies. Our definition therefore analytically implies the utilization of specific technologies that allow for the measurement and evaluation of the respective biological information and biomarkers. “Usage of (specific) technology” must accordingly not be included in the definition.

Third, our definition contains “improving tailoring of prevention and therapy” as one end of PM. If this is understood on the individual level, tailoring becomes impossible and the definition would be useless: it is impossible to find the drug that works perfectly for a single individual. The processes we employ to test medical devices and pharmaceuticals are clinical trials involving patients *groups* and not individual patients. Accordingly, tailoring means no more than stratification. In turn, stratification means the detection of sub-groups of patients that benefit from a certain measure. Tailoring treatment to a patient can therefore only mean assigning the patient to a certain sub-group of patients that appear to respond particularly well to a specific intervention. Against the background of these practical considerations, we can slightly adapt our definition:

PM seeks to improve stratification and timing of preventive and therapeutic measures by utilizing biological information and biomarkers on the level of molecular disease pathways, genetics, proteomics as well as metabolomics.

A final remark regards the part of the definition that refers to “improved preventive and therapeutic measures”. “Improved health care” is equivalent to “improved preventive and therapeutic measures” as prevention and therapy are what constitutes health care. To put it differently, improved preventive and therapeutic measures are implied in improved health care and vice versa. According to criterion 4, we can replace the former by the latter in the definition, but should not include both formulations. For reasons of efficiency of formulation we decided to include the latter arriving at the final definition:

PM seeks to improve stratification and timing of health care by utilizing biological information and biomarkers on the level of molecular disease pathways, genetics, proteomics as well as metabolomics.

## Conclusions

Above we have shown that the literature exhibits a huge variety of definitions of PM. A shared understanding that could facilitate the discourse on limits and chances of PM and preselect reasonable arguments is lacking. At the same time the relative amount of PM definitions given in the literature has been decreasing over the last years which we might carefully interpret as a trend: it gives cause to worry that people conceive PM as a well-described concept. This is especially worrisome because interpretations like “tailored wellness plan for individuals” (ends) or “consider belief of patients” (means) demonstrate that PM can be misused as a flexible void with a positive connotation that stakeholders fill with divergent meanings according to their interests and preferences.

To forestall such developments, we tried to supply the discourse with a precising definition. Starting point was our empirical data. Based on these findings, we analytically derived a *sufficiently* precise definition of PM that satisfies well-established criteria of adequate definitions:^g^

PM seeks to improve stratification and timing of health care by utilizing biological information and biomarkers on the level of molecular disease pathways, genetics, proteomics as well as metabolomics.

Hence, an adequate definition of PM is one of type c) mentioned in the beginning as it refers to treatment targeted at stratified subgroups (e.g. pharmacogenetics). Interestingly, our definition is also supported by a quantitative analysis of PM definitions in the literature. The number of occurrences of definitional elements suggests that PM should be understood as the use of genetic information (417 occurrences) – e.g. knowing of a specific genetic variation – to improve the treatment of patients by better tailoring their treatment (308) – e.g. by choosing a drug that does not exhibit severe side effects in the patient subgroup showing a specific genetic variation. The fact that our definition converges with the understanding of the majority suggests that it is widely *acceptable*.

This, of course, does not entail that it actually *will be accepted*. We hope, however, to stimulate a discussion on the understanding of PM that might help clarifying conceptual differences between stakeholders concerned with PM as well as avoiding unrealistic over- and underestimations in the public discourse on PM mentioned in the beginning.

Finally, four issues need to be addressed: First, we only included the usage of information on the genetic, proteomic, metabolomic and molecular pathway level as means of PM. In medical practice, however, we will rarely witness a medical encounter where decisions are based exclusively on information of such biomarkers. Rather, in the majority of cases information from both standard biomarkers (like blood pressure) and new molecular biomarkers (for instance, the existence of a specific genetic variant) will be combined to come to a reasonable treatment decision. Therefore, we realize that PM can only be understood as an add-on to standard medical care. Consequently, the aspired improvements can only be realized by combining both approaches.

Second, regarding our empirical analysis a potential limitation is that we only screened 1443 full-texts for definitions of PM, although we found 2312 papers that included the term PM in title or abstract. This problem is, however, commonly encountered in systematic reviews. It can also be criticized that we only searched PubMed for definitions. Seemingly, this poses the risk of introducing a bias as it is unclear whether the papers not scanned by us contained further definitional elements. However, analyzing our sample of 1443 papers led to conceptual saturation meaning no new themes emerged after analyzing a certain amount of articles [11]. Furthermore, we had no reason to suspect that our sample was significantly different from the general population of papers.

Third, one may criticize our exclusion decisions. To derive a precising definition we had to interpret terms like “tailored”, “health care” or “adequately distinctive”. For example, we define health care as being constituted by preventive and therapeutic measures. Naturally, those assumptions can be discussed critically as most terms are not as clearly delineated as would be desirable for the construction of a definition. However, as these terms are well-established in scientific discourse and interpretation only becomes necessary at the margin, this should not weaken our analysis in any significant way. Our definition can therefore help to re-focus the discourse on PM by reducing its conceptual vagueness.

Fourth, as mentioned above, people debating PM hold understandings that diverge from what PM technology actually can achieve. As our results show, PM is *not* medicine with a special focus on the interests and preferences of the individual patient. For instance, PM does not include any reference to an adequate doctor-patient relationship. Hence, PM as such is not related to the term patient-centered medicine. Moving towards a more patient-centered medicine may be desirable, but cannot be achieved by solely furthering PM technology. To forestall false hopes attached to the concept and accordingly wrong decisions regarding investments, it might be reasonable to adapt terminology. Stratifying medicine, for example, would be a more appropriate term than personalized medicine to describe the developments currently labeled as PM.

## Endnotes

^a^ For pragmatic reasons we will only use the acronym PM to refer to both individualized as well as personalized medicine in the following. Besides, individualized and personalized medicine are often used synonymously in public discourse.

^b^ See Additional file 2 for an overview of all the papers identified (including information on whether they were scanned and included in our analysis).

^c^ By medical context we refer to medicine as in medical *care*. This results from the insight that PM is generally discussed as a means to improve medical care.

^d^ Of course, “*medical* decision making” would belong to the genus “medicine”. The rather general reference to “decision making”, however, does not necessarily belong to the genus “medicine”. Therefore, we refrained from including “decision making” in our definition.

^e^ By “health care” we understand medical care both on the population and on the individual level.

^f^ We will return to that category later.

^g^ Cf. [13] for an alternative, purely analytically derived definition of PM. By characterizing our definition as *sufficiently* precise, we want to point out that it obviously cannot be *perfectly* precise. The construction of a perfectly precise definition would demand defining every element of the *definiendum*, e.g. the terms “biomarker”, “proteomics” or “metabolomics”. It is sufficiently precise in the sense that it can decrease the chances of misusing the term PM. For this purpose, it is not necessary to provide an exact definition of terms like proteomics or metabolomics. Rather, it is sufficient to point out that certain biological sub-disciplines are decisive for PM’s development and implementation.

## Competing interests

The authors declare that they have no competing interests.

## Authors’ contributions

SS initiated the study and contributed to conception and design, acquisition of data, analysis and (analytical) interpretation of data. He was involved in drafting the manuscript and revised it. CK contributed to data analysis and interpretation and was involved in drafting the manuscript as well as revision. TB contributed to acquisition of data and revised the manuscript. WHR contributed to conception and design and was involved in drafting and revising the manuscript. GM contributed to data interpretation and was involved in drafting and revising the manuscript. All authors gave final approval of the paper.

## Pre-publication history

The pre-publication history for this paper can be accessed here:

http://www.biomedcentral.com/1472-6939/14/55/prepub

## Supplementary Material

Additional file 1 Ends and Means of PM in the Literature (Long). Click here for file

Additional file 2 Searched Papers Containing PM in Title/Abstract. Click here for file
