# Supplementary material for: What is personalized medicine: sharpening a vague term based on a systematic literature review
Source: BMC Med Ethics. 2013 Dec 21;14:55. doi: 10.1186/1472-6939-14-55 (PMC3878093; doi:10.1186/1472-6939-14-55)
Supplement: Additional file 1 — Ends and Means of PM in the Literature (Long). [file 1472-6939-14-55-S1.doc]

**Additional file 1 Ends and Means of PM in the Literature (Long)**

| **ENDS IN (1459)** | **MEANS IN (1025)** |
| --- | --- |
|  | |
| **1 RESEARCH (156)** | **1 RESEARCH (97)** |
| **A. Basic Research (43)** | **A. On Individual Differences (4)** |
| ***On causes and processes of diseases (9)*** | determine individual biological differences (1) |
| understanding the pathogenesis of disease (1) | understanding the unique molecular profile (1) |
| understand causality of disease (2) | understand genetic differences between individuals (1) |
| new insight into the causes of diseases (1) | understand phenotypic variability (1) |
| better understanding of the etiology of the disorder/disease (2) | **B. On Genetics/Genomics (39)** |
| improve our understanding of the disease processes (1) | ***Unspecified (11)*** |
| better understanding of (tumor) biology (1) | understanding of genomics (1) |
| gaining biological insights into pathways relevant for the diseases or trait under investigation (1) | identify genotypes (2) |
| ***On risk factors for diseases (3)*** | identify genetic variation (5) |
| understanding genetic risk for disease (1) | gain understanding of genetic makeup (1) |
| understanding environmental risk for disease (1) | discover genetic alterations (1) |
| understand disease risk (1) | develop genetic testing (1) |
| ***On disease classification (18)*** | ***Regarding the influence of genes on disease development/progression (5)*** |
| create a better system for disease classification (1) | Identification of the genetic basis of prognosis (1) |
| establishing a strictly individualized disease approach (1) | Identification of the genetic basis of survival (1) |
| defining/identifying disease subtypes (16) | identifying the genetic contribution to the diseased state (1) |
| ***To further the development of new treatment measures (13)*** | increasing knowledge of the genetic basis of disease (1) |
| facilitate the development of new therapies (1) | understanding the genetic mechanisms contributing to disease development (1) |
| facilitate the development of more effective therapies (1) | ***Regarding the influence of genes on drug response (22)*** |
| guidance of drug development (1) | study of genetically determined variation in drug response (2) |
| advancing drug development (1) | define the array of genetic variants that may underlie drug response (1) |
| optimize drug development (2) | investigate the relationship between drug response and genetic differences (1) |
| improve the process of drug development (2) | link genetic variation with differences in the drug responses (1) |
| facilitate discovery of new (health care) products (2) | understanding of influence of genes on variable drug/ treatment response (3) |
| contribute to discovery of new drugs (1) | Identification of the genetic basis of variable treatment response (3) |
| contribute to discovery of new medical products (1) | identification of genes involved in incorrect response to treatment (1) |
| improve process of drug discovery (1) | identifying/understanding the genetic contribution to drug efficiacy (2) |
| **B. Research on New Diagnostic and Prognostic/Predictive Measures (30)** | understand genetic influence on drug metabolism (1) |
| ***Unspecified (10)*** | identifying the genetic basis for treatment resistance (2) |
| new diagnostic tests (1) | identifying the genetic contribution to drug toxicty (1) |
| new screening tests (1) | understanding of influence of genes on risk of side effects (4) |
| developing new targets for detection of disease (1) | ***Regarding the interaction of genes with other factors that influence disease development/ progression (1)*** |
| discover prognostic tools (1) | increase understanding of gene-environment interactions (1) |
| refine prognosis (1) | **C. On Further Factors that Influence Disease Progression/Development (8)** |
| discover predictive markers (1) | understanding effect of age (1) |
| discover predictive tools (1) | understanding effect of gender (1) |
| develop predictive tools (1) | understanding of the impact of gender differences (1) |
| develop risk assessment profiles (1) | understanding effect of comorbidity (1) |
| facilitate predictive diagnostics (1) | identification of (molecular) pathways involved in disease progression (2) |
| ***Regarding tailoring (2)*** | ascertain the molecular basis of disease (1) |
| development of targeted agents (2) | understand the specific characteristics underlying a particular individual’s disease (1) |
| ***Regarding stratification (4)*** | understand the specific characteristics underlying a particular individual’s disease (1) |
| define subgroups of patients according to expected phenotype (1) | **D. On Further Factors that Influence Drug Response (4)** |
| define subgroups of patients according to clinical prognosis (1) | discover proteins responsible for differential response (1) |
| define subgroups of patients according to likelihood for disease to reoccur (1) | develop biomarkers for drug response (2) |
| define subgroups of patients according to predisposition to disease (1) | understand the physiological differences that underlie drug response (1) |
| ***Regarding improved diagnostic/prognostic/predictive measures (9)*** | **E. On Biomarkers (40)** |
| **unspecified (2)** | defining biomarkers (4) |
| enable better prognosis (2) | characterization of novel biomarkers (1) |
| **effectiveness (6)** | provide biomarker information (2) |
| understand variable drug response (2) | identification of biomarker (12) |
| identify best/optimal drug targets (3) | the identification of new biomarkers (2) |
| new drug target discovery (1) | discovery of biomarkers (2) |
| **efficiency (1)** | discovery of novel biomarkers (1) |
| develop more efficient diagnostic solutions (1) | develop new biomarkers (1) |
| ***Regarding companion diagnostics (5)*** | develop biomarkers (1) |
| discovery and application of companion diagnostics (1) | develop new biomarker diagnostics (1) |
| development of companion diagnostics (1) | development of sophisticated new biomarkers (2) |
| companion diagnostics (3) | identify prognostic biomarkers (1) |
| **C. Research on New Therapeutic Measures (83)** | identify predictive biomarkers (4) |
| ***Unspecified (15)*** | provide predictive biomarkers (1) |
| develop novel treatments (3) | provide diagnostic biomarkers (1) |
| development of novel therapies (1) | validate biomarkers (4) |
| develop new treatment strategies (1) | **F. On New Technologies (2)** |
| new and useful therapies (1) | develop technologies to determine gene expression profiles (1) |
| new therapeutic targets (3) | development of comparative diagnostic tests (1) |
| drug development (2) |  |
| develop new drugs (2) |  |
| new drugs (2) | **2 APPLICATION IN PATIENT CARE (928)** |
| ***Regarding tailoring (15)*** | **A. Usage of Clinical Information/Clinical Indicators (718)** |
| develop tailor-made treatment (1) | ***Unspecified (11)*** |
| achieve finely tuned treatment (1) | use clinical information (5) |
| develop specific therapies (1) | informed by clinical information (1) |
| create new line of tailored therapeutics (1) | based on clinical information (2) |
| create tailored therapies (1) | based on clinical data (1) |
| discover tailored therapies (1) | based on clinical profile (1) |
| generate tailor-made therapies (2) | use clinical characteristics (1) |
| develop precise therapies (1) | ***Using information on medical history (12)*** |
| develop targeted drugs (2) | **unspecified (6)** |
| develop better tailored drugs (1) | historical data (1) |
| design patient-specific drug therapy (1) | history taking (1) |
| more appropriate drugs (1) | take into account patients (medical) history (2) |
| develop customized medical treatments (1) | clinical history (1) |
| ***Regarding stratification (40)*** | integrating advances with clinical histories (1) |
| define subgroups of patients according to variation of treatment response (6) | **regarding family history (6)** |
| define subgroups of patients according to drug responsiveness (1) | family history (1) |
| define subgroups of patients likely to respond (6) | integrating advances with family histories (1) |
| define subgroups of patients not likely to respond (4) | based on family history (3) |
| define subgroups of patients most likely to respond (2) | use information about family history (1) |
| define subgroups of patients likely to benefit (3) | ***Using biological information/biomarkers (689)*** |
| define subgroups of patients most likely to benefit (11) | **unspecified (81)** |
| define subgroups of patients least/not likely to benefit (2) | biological attributes (2) |
| define target population (1) | based on biological data (1) |
| improve stratification of patients (2) | based on biology (3) |
| define subgroups of patients likely to experience adverse effects (2) | use of biological information (3) |
| ***Regarding improved treatment measures (13)*** | based on biological profile (3) |
| **unspecified (5)** | based on biological makeup (1) |
| develop optimized treatments (2) | based on biological variation (2) |
| design optimal healthcare strategies (1) | informed by biological characteristics (1) |
| designing optimal therapeutic interventions (1) | identify biologic targets (1) |
| rational drug design (1) | use systems biology (2) |
| **safety (3)** | biomarkers (9) |
| developing safer treatment (2) | based on biomarkers (6) |
| create safer drugs (1) | using biomarkers (11) |
| **effectiveness (3)** | using biomarker tests (1) |
| develop effective therapies (1) | rely on biomarkers (1) |
| release of highly effective drugs (1) | considering biomarkers (1) |
| reveal most appropriate therapeutic targets (1) | incorporating biomarkers (1) |
| **efficiency (2)** | measuring biomarkers (2) |
| develop more efficient therapeutic solutions (1) | application of biomarkers (1) |
| create more efficient drugs (1) | analysis of biomarkers (1) |
|  | depend on biomarker profile (1) |
| **2 DRUG APPROVAL (14)** | based on validated biomarkers (1) |
| **A. Improved Validation Processes (1)** | predictive biomarkers (7) |
| facilitate validation of health care products (1) | define predictive biomarkers (1) |
| **B. Improved Clinical Trials (11)** | based on predictive biomarkers (2) |
| ***Unspecified (4)*** | prognostic biomarkers (2) |
| contribute to clinical testing of new drugs (1) | based on prognostic biomarkers (1) |
| contribute to clinical testing of new biomarkers (1) | diagnostic biomarkers (2) |
| facilitate clinical testing of new products (1) | imaging based biomarkers (1) |
| enriched clinical trials (1) | biochemical markers (1) |
| ***Efficiency (7)*** | biomarkers for patient selection (1) |
| reduce time of clinical trials (2) | markers for risk prediction (1) |
| reduce cost of clinical trials (2) | utilizing individualized diagnostic information (1) |
| reduce failure rate of clinical trials (3) | based on laboratory information (1) |
| **C. Improved Approval Processes (2)** | identify allergies (1) |
| fewer withdrawels of marketed drugs (1) | use information on allergies (1) |
| decreased time to approval (1) | measure blood pressure (1) |
|  | measure body temperature (1) |
|  | metabolism (1) |
| **3 HEALTH CARE (1202)** | **of the individual (21)** |
| **A. Patient Care in General (69)** | biology of the individual (3) |
| ***Unspecified (1)*** | measured biological status of the patient (1) |
| manage care of individual (1) | unique biologic profile (3) |
| ***Decision making in health care(3)*** | identify individual microbial profiles (1) |
| make decisions about care (1) | use biological characterization of patient (2) |
| informed health care decisions (2) | the application of an individual’s biological  fingerprint (1) |
| ***Tailoring health care (37)*** | tailored biomarkers (1) |
| tailoring/tailored medical care (10) | individual differences in drug metabolism (7) |
| tailor medical care to an individual's needs (1) | incorporating physiological variables (1) |
| tailoring health care (1) | physiology of patient (1) |
| individualized medical care (1) | **of the disease (13)** |
| targeted approach to care (1) | use tumor markers (1) |
| customized medical care (4) | use information on the biology of the disease (1) |
| customization of patient care (1) | determinded by the characteristics of the disease (2) |
| patient-specific health care (2) | according to the characteristics of the disease (3) |
| tailor-made medicine (2) | data on characteristics of disease (1) |
| administer tailored medicine (1) | medical application of new insights into causes of  disease (1) |
| tailored medicine (4) | customized to special characteristics of a patient and  his/her disease (1) |
| individualized medicine (6) | use information on the biology of the disease (1) |
| patient-specific approaches (1) | individual patients’ specific disease parameter (1) |
| customized, patient-specific strategies (1) | address individual aspects of a patient’s disease state  (1) |
| targeted, patient-centric solutions (1) | **on the molecular level (574)** |
| ***Improved health care (28)*** | *unspecified (101)* |
| **unspecified (17)** | molecular biology (1) |
| optimize medical care (3) | molecular basis (2) |
| optimize care (2) | informed by molecular characteristics (1) |
| improve clinical care (1) | to molecular characteristics (1) |
| improve patient care (2) | based on molecular characteristics (3) |
| improve health care (4) | use molecular characterization (3) |
| improve quality of healthcare (1) | driven by molecular characterization of disease  (1) |
| provide better care (1) | testing the molecular characteristics (of tumor)  (1) |
| better medical care (2) | inside into molecular characteristics (1) |
| provide best possible patient care (1) | molecular analysis (2) |
| **effectiveness/efficacy (1)** | use molecular analysis (2) |
| more effective medicine (1) | supported by molecular analysis (1) |
| **efficiency (2)** | exploit knowledge of molecular profile (1) |
| improve efficiency of healthcare (2) | based on molecular profile (3) |
| **safety (2)** | based on molecular profiling (1) |
| improve safety of healthcare (1) | molecular profiling (5) |
| safer medicine (1) | molecular profile (3) |
| **choices in health care (1)** | link molecular profile with diagnosis and  treatment (1) |
| achieve optimal individual health-care decisions (1) | use molecular profile (4) |
| **tailoring health care (5)** | derived from molecular profile (1) |
| improved tailoring of care (1) | use molecular profiling technologies (1) |
| better targeted medical care (1) | identify molecular profiles (1) |
| a more focused personalized approach to care (1) | application of molecular profiles (1) |
| a more tailored approach to care (2) | consider molecular data (1) |
| **B. Prevention (158)** | application of molecular data (2) |
| ***Risk prognosis/prediction (82)*** | molecular (diagnostic) tests (3) |
| **unspecified (52)** | through molecular diagnostics (3) |
| identify predisposition to disease (1) | molecular diagnostic products (1) |
| predict susceptibility for a disease(1) | use molecular dagnostic testing (2) |
| quantify susceptibility to disease (1) | molecular variation (1) |
| determine disease susceptibility (3) | information about molecular variation (1) |
| predict disease (4) | knowledge of the molecular diversity (1) |
| prediction of disease etiology (3) | provide molecular information (1) |
| early identification of risks (4) | use genome-scale molecular information (1) |
| prediction of risk (4) | potential of molecular information (1) |
| ability to predict risk (1) | use molecular signatures (3) |
| emphasize risk prediction (1) | identify patients by molecular signatures (1) |
| determine risk for disease (2) | validating unique blood-based molecular  signatures (1) |
| evaluate disease risk (1) | molecular makeup (1) |
| predict risk for disease (2) | center on molecular makeup (1) |
| assess risk for disease (1) | molecularly targeted agents (1) |
| predictive outcomes (1) | molecular targets (2) |
| provide preventive diagnostics(1) | take advantage of molecular understanding of disease (1) |
| predictive intervention (1) | informed by a molecular understanding of  disease (1) |
| predictive medicine (1) | based on molecular aspects of diseases (1) |
| risk stratification (7) | target molecular defects (1) |
| aim at risk assessment (1) | use molecular markers (4) |
| identify risk factors (2) | based on molecular markers (1) |
| identify risk patients (8) | molecular imaging (2) |
| adapted prediction (1) | molecular approaches (1) |
| **at the individual level (26)** | based on molecular assays (1) |
| evaluating the individual patient’s intrinsic  susceptibility (1) | based on molecular features (2) |
| evaluating the individual patient’s extrinsic  susceptibility (1) | molecular classification (1) |
| evaluating the individual patient’s intrinsic  morbidity (1) | molecular tools (1) |
| evaluating the individual patient’s extrinsic  morbidity (1) | identification of molecular abnormalities (1) |
| genomic risk assessment (1) | exploit molecular traits (1) |
| genetic risk factors (1) | use molecular knowledge (1) |
| assessment of genetic protective factors (2) | molecular biomarkers (9) |
| individualized prediction (1) | uses molecular profiling technologies (1) |
| targeted prediction (1) | identify molecular targets (2) |
| individual risk assessment (8) | *information on disease pathways (7)* |
| individualizing risk assessment (2) | therapeutic agents that are able to selectively  block or redirect the disease pathways (1) |
| (assessing) individual variation in risk (2) | improved knowledge of molecular pathways (1) |
| reflect individual risk (1) | target pathways in drug development (1) |
| evaluation of individual risk factors (1) | targeted to metabolic pathway (2) |
| individual risk profiles (2) | disrupt a specific biochemical pathway (1) |
| **at the population level (3)** | variation in metabolic pathways (1) |
| determine the predisposition to disease at the  population level (1) | *genetic information (417)* |
| environmental risk factors (1) | unspecified (19) |
| environmental protective factors (1) | genetics (4) |
| **improved risk prognosis/prediction (1)** | based on genetics (6) |
| maximize prediction (1) | use of genetics (8) |
| ***Primary prevention (76)*** | incorporating genetics (1) |
| **unspecified (34)** | regarding knowledge of  genes/genetic variation (215) |
| prevent disease (16) | knowledge of genes (1) |
| preventive treatment (2) | according to genes (1) |
| preventive therapies (1) | genetic biomarkers (3) |
| permit preventive intervention (1) | use genetic biomarkers (1) |
| establish preventive measures (1) | genetic information (3) |
| recommend preventive measures (1) | use genetic information (15) |
| take preventive countermeasures (2) | based on genetic information (4) |
| take protective measures (1) | apply genetic information (1) |
| prevent predisposition to disease (2) | use of genetic variations (3) |
| avoid predisposition to disease (1) | based on genetic variations (11) |
| modify predisposition to disease (1) | genetic variation (2) |
| prospective approach to prevention (1) | directed by information of genetic  variation (1) |
| provide preventive therapeutics (1) | based on knowledge of genetic diversity  (1) |
| preventive intervention (1) | use information on genetic variation (1) |
| management of disease predisposition (2) | according to genetic variation (1) |
| **tailoring preventive measures (22)** | based on genetic profile (23) |
| tailored prevention (7) | use genetic profile (3) |
| tailor strategies for disease prevention (2) | according to genetic profile (1) |
| targeted prevention (2) | designed for genetic profiles (1) |
| adapted prevention (1) | target genetic profile (1) |
| individualized prevention (2) | customization to genetic profile (1) |
| patient specific prevention (1) | immunogenetic profile (1) |
| provide a tailored preventive regimen (1) | genetic profile (6) |
| preemptive individualization (1) | variation in genetic profiles (1) |
| tailored preventive interventions (2) | application of genetic profiles (1) |
| achieve personalized prevention (1) | produce genetic profiles (1) |
| provide focused prevention (1) | evaluate genetic profile (1) |
| measures adapted to a personal genetic  predisposition (1) | genetic profiling (1) |
| **stratified preventive measures (1)** | characterized by distinct genetic profiles  (1) |
| deliver stratified prevention (1) | determine unique genetic profile (1) |
| **improved prevention/preventive**  **strategies (19)** | use genetic makeup (4) |
| *unspecified (16)* | based on genetic makeup (16) |
| facilitate prevention (1) | according to genetic makeup (4) |
| more preventive treatment (1) | to genetic makeup (8) |
| improve prevention (2) | integrate knowledge on genetic makeup  (1) |
| maximize prevention (1) | impact of genetic makeup (1) |
| optimize preventive (health care) strategies (3) | use information on genetic makeup (2) |
| optimize preventive management (1) | in context of genetic makeup (1) |
| enable better prevention (1) | take genetic makeup into account (1) |
| improved management of predisposition to  disease (2) | in function of genetic makeup (1) |
| comprehensive prevention (2) | according to genetic composition (2) |
| minimize risk (1) | use genetic composition (1) |
| optimal outcome in management of  predisposition to disease (1) | consider genetic composition (1) |
| *effectiveness (1)* | understanding a patient’s genetic  composition (1) |
| effective disease prevention (1) | genetic characteristics (1) |
| *timing (2)* | use genetic characteristics (2) |
| accelerate the prevention of disease (1) | incorporating genetic characteristics (1) |
| deliver timely prevention (1) | informed by genetic characteristics (1) |
| **C. Therapy (975)** | genetic characterization of diseases(1) |
| ***Diagnosis (68)*** | exploit genetic data (1) |
| **unspecified (18)** | use of genetic markers (4) |
| diagnosis (3) | based on genetic markers (1) |
| diagnostics (1) | by genetic markers (1) |
| diagnose disease (7) | based on genetic factors (2) |
| diagnostic outcomes (1) | considering genetic factors (1) |
| determine an individual’s health status (2) | take genetic factors into account (1) |
| appropriate diagnosis (1) | based on gene mutations (1) |
| rational diagnosis (1) | genetic mutations (1) |
| prospective approach to diagnosis (1) | use gene mutations (1) |
| identify extent of disease (1) | use mutation data (1) |
| **tailored diagnosis/diagnostics (28)** | based on mutational profiling data (1) |
| targeted diagnostics (1) | discover genetic mutations (1) |
| individualized diagnosis (7) | depend on mutation status (1) |
| tailored diagnosis (10) | dictated by unique genetic signatures (1) |
| tailored diagnostic regimen (1) | analysis of genetic signature (1) |
| trace exact diagnosis down to individual patient (1) | based on genetic signatures (2) |
| patient specific disease detection (1) | emphasize genetic background (1) |
| tailored (disease) detection (3) | based on genetic background (1) |
| tailor strategies for disease detection (2) | driven by genetic analysis (1) |
| provide patients with personalized diagnostic  information (1) | based on genetic risk (1) |
| provide focused detection (1) | risk defined through genetics (1) |
| **improved diagnosis/diagnostic measures**  **(22)** | based on genetic determinants (1) |
| *unspecified (10)* | based on genetic traits (1) |
| better diagnosis (2) | based on genetically determined  pathophysiology (1) |
| improve diagnosis (3) | based on specific single nucleotide  polymorphisms (2) |
| optimize diagnostic strategies (1) | based on genetic polymorphisms (5) |
| maximize diagnosis (1) | identification of target gene  polymorphisms (1) |
| comprehensive approach to diagnosis (1) | based on genetic inheritance (1) |
| diagnose disease in asymptomatic individuals  (1) | consider genetic status (2) |
| diagnose diseases before symptom onset (1) | take genetic constitution into account (1) |
| *effectiveness/efficacy (5)* | unique genetic code (1) |
| more effective diagnosis (1) | assessing a patient's crucial gene (1) |
| more precise diagnosis (1) | genetic evaluation of individuals (1) |
| more accurate diagnosis (3) | apply genetic assays (1) |
| *timing (6)* | genotype (1) |
| accelerate the detection of disease (1) | based on genotype (14) |
| early diagnosis of disease (3) | based on genotyping (4) |
| early disease detection (2) | by genotyping (5) |
| *tailored diagnostics (1)* | use genotypic factors (1) |
| perfectly tailored diagnostics (1) | based on idea of using genotype (1) |
| ***Prognosis/Prediction (158)*** | regarding gene expression (13) |
| **unspecified (4)** | based on gene expression (2) |
| predict prognosis (1) | identify variable gene expression (1) |
| prognosis (2) | use gene expression data (1) |
| data on prognosis of disease (1) | based on gene expression profiling data  (1) |
| **prediction of disease**  **progression/recurrence (5)** | characterized by gene expression profiles  (1) |
| predict disease progression (4) | based on gene expression profiles (2) |
| predict recurrence (1) | use gene expression profiling (1) |
| **predictive information guiding clinical**  **decision making (21)** | based on gene expression profiling (2) |
| predict choice of drug prescription (1) | implementing genotype-phenotype data  into the clinical decision process (1) |
| guide choice of therapy (3) | Identifying genotype–phenotype  relationships (1) |
| guide therapeutic approach (1) | regarding genomics/genomic  variation (98) |
| guide medical decision making (4) | based on complete genetic information (1) |
| guide treatment choices (4) | use genomic analysis (1) |
| guide health care decisions (2) | based on genomic analysis (2) |
| guide clinical management (1) | apply genomic analysis (1) |
| predict which treatments will benefit patients (1) | based on genomic makeup (2) |
| predict best intervention (1) | based on genome-wide investigations (1) |
| predict best possible drug therapy (1) | understanding the unique properties of  each patient’s genome (1) |
| identify the optimal treatment (1) | use pangenomic information (1) |
| predict best possible drug dose (1) | genomic biomarkers (1) |
| **prediction of treatment**  **effects/effectiveness (60)** | genome/genomics (4) |
| predict therapy/treatment outcomes (6) | based on genomics/genome (9) |
| prediction of clinical outcomes (2) | use of genomics/genome (3) |
| prediction of therapeutic effects (1) | genomic attributes (1) |
| prediction of therapeutic efficiacy (3) | application of genomics (1) |
| predict drug efficiacy (2) | genomic methods (1) |
| predict effectiveness of therapy (1) | tailored to the specific state of an  individual’s genome (1) |
| prediction of (therapeutic) side effects (9) | apply genomic knowledge (1) |
| predict drug toxicity (3) | integrate genomic knowledge (2) |
| predict resistance (1) | integrate genomics (1) |
| prediction of response (5) | genomic information (1) |
| predict response to treatment (7) | use genomic information (17) |
| predict probability of drug response (1) | based on genomic information (6) |
| determine drug response (1) | exploit genomic information (1) |
| prediction of outcome (3) | drawn from information about genomic  variation (1) |
| anticipation of possible side-effects (1) | according to genomic variation (1) |
| information about differences in response to  intervention (1) | use genomic variation (1) |
| identification of variable treatment response (13) | based on analysis of genomic variation (1) |
| **tailoring prognosis/prediction (39)** | based on genomic profile (7) |
| individualized prognosis (3) | build genomic profile (1) |
| targeted prognosis (1) | genomic profile (4) |
| tailored prognosis (3) | based on genomic profiling data (1) |
| provide focused prognostic efforts (1) | that work well with genomic profile (1) |
| prediction of individual response to treatment (17) | describe genomic makeup (1) |
| prediction of individual response to drugs (9) | informed by genomic characteristics (1) |
| determine patient specific drug response (1) | use of genomic data (2) |
| define individual responsiveness to drugs (3) | based on genomic data (1) |
| determine individual variation on drug response (1) | application of genomic data (3) |
| **stratification by predicted treatment**  **effects (25)** | use of genomic markers (3) |
| stratification for specific treatment (modalities) (2) | based on genomic factors (1) |
| distinguish patients by variation in drug response  (1) | use genomic signatures (2) |
| identify patients likely to respond to treatment (2) | according to unique genomic background  (1) |
| identify patients unlikely to respond to treatment  (2) | exploit associations between genomic  variants and drug responses (2) |
| identify patients most likely to respond to treatment  (2) | use genomic blueprint (1) |
| identify patients likely to manifest side  effects/respond adversely (6) | sequencing genome (2) |
| identify responders (6) | regarding the transcriptome (4) |
| identify nonresponders (2) | consider transcriptomic data (1) |
| identify poor responders (1) | based on transcriptomic profile (1) |
| relate groups to certain treatment options (1) | use whole-blood transcriptome profiling  (1) |
| **improvement of prognostic/predictive**  **measures (4)** | transcriptomic methods (1) |
| *unspecified (2)* | regarding DNA/RNA(6) |
| improve prognosis (2) | use DNA (1) |
| *safety (1)* | use of DNA arrays-derived information  (1) |
| improving the prediction of safety of drugs (1) | through DNA assessment (1) |
| *effectiveness/efficacy (1)* | DNA analyis (1) |
| improving the prediction of efficiacy of drugs  (1) | use information about RNA (1) |
| ***Treatment (749)*** | based on microRNAs (1) |
| **unspecified (27)** | regarding epigenetics (8) |
| treatment (2) | epigenetic biomarkers (1) |
| treat disease (8) | based on epigenetics (2) |
| give adequate treatment (1) | based on epigenomics (1) |
| rational treatment (2) | epigenomic variation (1) |
| therapeutics (1) | based on epigenomic profile (2) |
| administer medication (1) | to describe epigenomic makeup (1) |
| drug treatment (1) | regarding gene-environment  interaction (4) |
| prescribe drugs (2) | identify gene by environment interactions  (1) |
| recommend drug treatment (1) | based on interaction of genes and the  environment (1) |
| curative treatment (1) | use of genetic-environmental markers (1) |
| cure disease (1) | interaction of genetic disposition and  environmental factors (1) |
| control disease (1) | regarding pharmacogenetics*/*  pharmacogenomics (41) |
| rational use of drug (2) | pharmacogenetics (3) |
| ablate the disease process (1) | pharmacogenetic profile (1) |
| management of disease (2) | based on pharmacogenetic information (4) |
| **choice of therapeutic measure (104)** | use pharmacogenetic information (1) |
| select appropriate treatment (4) | through pharmacogenetics(2) |
| identify appropriate therapies (1) | based on pharmacogenetics (2) |
| selection of appropriate therapeutic agent (1) | use pharmacogenetics (3) |
| selecting the right therapeutic strategy (1) | based on pharmacogenetic data (1) |
| avoid unnecessary interventions (1) | through knowledge of pharmacogenetics  (1) |
| define the appropriate intervention (1) | advances in pharmacogenetics (1) |
| select medication (2) | pharmacogenomics (9) |
| use of a specific medication regimen (1) | based on advances in pharmacogenomics  (1) |
| prescribe appropriate medications (1) | based on pharmacogenomic information  (3) |
| avoid unnecessary medication (1) | use pharmacogenomics testing (2) |
| adjusted use of drug (3) | use of pharmacogenomics (2) |
| determine appropriate drug (1) | application of pharmacogenomics(1) |
| choose suitable drug (2) | through knowledge of pharmacogenetics  (1) |
| prescribe suitable drug (1) | use pharmacogenomic profiling (1) |
| rational approach for prescribing drugs (1) | use better understanding of genetic basis  of drug resistance (1) |
| select appropriate drug dosage (1) | genetic characterization of response to  treatment (1) |
| determine appropriate drug dosage (2) | regarding further points (9) |
| determine drug dosage (1) | cytogenetic analysis (1) |
| select dose (2) | based on haplotypes (2) |
| dose modification (1) | by haplotyping, SNPs (1) |
| adjust dose (5) | tailored genetic counseling (1) |
| the right drug at the right time for the right  person/patient (49) | use nutrigenomics (1) |
| no traditional "one size fits it all" approach (18) | molecular phenotype (1) |
| no “trial and error” (3) | based on phenomic profile (1) |
| **regarding treatment monitoring (12)** | according to molecular phenotype (1) |
| monitoring of therapy (5) | *information on proteomics (35)* |
| provide optimal drug screening (1) | proteomic biomarkers (4) |
| patient monitoring (6) | proteomics (3) |
| **effectiveness/efficacy of treatment (16)** | use of proteomics (3) |
| effective treatment (3) | integrate proteomics (1) |
| use effective medical treatment (1) | build proteomic profile (1) |
| ability for effective treatment (1) | based on proteomic profile (4) |
| provide effective treatment (1) | based on proteomic profiling data (1) |
| effective therapy (4) | use proteomic profiling (1) |
| deliver effective therapies (1) | variation in proteomic profiles (1) |
| focus on effective therapies (1) | based on proteomic makeup (1) |
| efficacy of therapeutic interventions (1) | describe proteinomic makeup (1) |
| cope with individual differences in drug efficiacy  (1) | apply proteomic assays (1) |
| limit nonresponse to treatment (1) | use proteomic information (5) |
| provide effective tools against disease (1) | based on proteomic information (1) |
| **efficiency of treatment (2)** | proteomic evaluation of individual (1) |
| efficient therapy (1) | integrate proteomic knowledge (1) |
| economically feasible therapies(1) | consider proteomic data (1) |
| **safety of treatment (6)** | monitor (disease related) changes in patients  proteome (1) |
| use safe medical treatment (1) | certain characteristics in patients blood proteins  (1) |
| avoid (treatment) toxicity (4) | certain characteristics in cell surface proteins  (1) |
| prevent adverse drug reactions (1) | based on pharmacoproteomic information (1) |
| **treatment outcomes (2)** | *information on metabolomics (14)* |
| focus on therapeutic outcomes (1) | integrate metabolomics (1) |
| provide treatment benefits (1) | use information about metabolites(1) |
| **tailoring therapy/therapeutic measures**  **(269)** | consider metabolomic data (1) |
| adapted therapy (5) | use metabolomic analysis (1) |
| provide focused therapeutic efforts (1) | variation in metabolic profiles (1) |
| patient specific treatment (8) | drug-metabolizing enzymes (2) |
| select treatment for individual (patient) (1) | based on metabolic profile (1) |
| targeted treatment (12) | metabolic profile (1) |
| tailored/ tailoring treatment (39) | metabolomics (2) |
| tailored medical treatment (2) | targeted to metabolic receptor (1) |
| tailored treatment plans (1) | use metabolic status (1) |
| tailored treatment strategies (2) | metabolomic biomarkers (1) |
| tailor strategies for disease treatment (2) | ***Further points mentioned (6)*** |
| tailored therapeutic treatments (1) | detailed epidemiologic information on known risk markers (1) |
| custom-tailored treatment (4) | based on physiological factors (1) |
| individualized treatment (16) | based on symptoms (1) |
| develop individualized treatment plans (1) | use information on concurrent medication (1) |
| prescription of individualized treatment (1) | consider coexisting conditions (1) |
| individualized selection of treatment (1) | use information on comorbidity (1) |
| custom-made treatment (2) | **B. Usage of Environmental Factors/Information (33)** |
| customize treatment (3) | consider environment (1) |
| adapted treatment (3) | use environmental factors (4) |
| treatment that matches the patient (1) | consider environmental factors (1) |
| a treatment suited to the patient’s condition (1) | incorporating environmental factors (1) |
| apply specific therapy (1) | use environmental information (11) |
| prescribe specific therapies (1) | impact of environmental influences (1) |
| tailored therapy (29) | environmental data (1) |
| administer tailored therapy (1) | environmental state (1) |
| deliver custom-tailored therapies (1) | environmental attributes (1) |
| provide custom-tailored therapeutic approaches (1) | use environmental characteristics (1) |
| use of custom-tailored therapies (1) | based on environmental exposure (3) |
| tailoring the therapeutic strategy (1) | take environmental exposure into account (1) |
| molecularly tailored therapy (1) | based on analysis of environmental signature (1) |
| provide a tailored therapeutic regimen (1) | integrate knowledge on environmental conditions (1) |
| establish individualized therapy (1) | draw from information about differences in environmental determinants (1) |
| individualized therapy (8) | based on environmental information (2) |
| allow individualized therapy (1) | informed by environmental influences (1) |
| individualized therapeutic regimens (1) | **C. Usage of Further Individual Factors/Information (126)** |
| establish targeted therapy (1) | ***Unspecified (77)*** |
| targeted therapy (20) | use of all available patient data (1) |
| targeted therapeutics (3) | patients (unique) characteristics (4) |
| molecularly targeted therapy (6) | according to the specific circumstances of the patient (1) |
| application of targeted therapy (1) | using the patient’s characteristics (1) |
| specific targeted therapies (1) | characteristics of individual patient (6) |
| customized therapy (4) | determinded by the characteristics of the patient (1) |
| use of biologically targeted therapeutics (1) | according to individual characteristics (1) |
| targeted interventions (1) | according to the characteristics of the host (patient) (1) |
| tailored interventions (2) | noninvasive characterization of patient (1) |
| tailored therapeutic interventions (3) | patient-client characteristics (1) |
| patient-tailored interventions (1) | measurement of the patient’s characteristics (1) |
| customize medical interventions (1) | based on individual-level characteristics (1) |
| apply targeted interventions (1) | taking into account the individual variability (2) |
| selectively administered interventions (1) | based on our understanding of individual differences in patients (1) |
| tailored medication (2) | based on individual patient differences (2) |
| permit accurate tailoring of medications (1) | create awareness of individual differences (1) |
| prescribe corrective, tailored measures (1) | for each individual patient (5) |
| tailored pharmatherapeutical medicine/therapy (3) | on individual basis (1) |
| individualize pharmacotherapy (2) | in an individual patient (1) |
| pharmaceutical therapies tuned to individual patient  needs (1) | at level of individual patient (1) |
| prescribe targeted drugs (2) | to the individual (20) |
| treat with targeted drugs (1) | individuals/patients needs (8) |
| use of targeted drugs (3) | according to individual needs (1) |
| molecularly targeted drugs (4) | based on individual needs (1) |
| tailoring drugs (7) | take into account the needs of individual patients (1) |
| tailored prescriptions (3) | meet individual needs (2) |
| tailored drugs to genotype (5) | identify specific needs (1) |
| tailored drug regimens (2) | tailoring components to specific patients (1) |
| tailor drug regimens to genotype (1) | to patients circumstances (1) |
| match drug to genotype (1) | based on knowledge about the individual´s state (1) |
| tailor-made drugs (2) | patient´s unique condition (2) |
| tailored drug therapy (1) | based on individual susceptibilities (1) |
| individualized drug treatment (1) | use specific information about patient (2) |
| individualized drug therapy (2) | use patients individual data (1) |
| individualized prescription (1) | ***Age-related information (4)*** |
| individualized drug selection (2) | age (1) |
| patient-specific drug treatment (1) | consider age (2) |
| customized drugs (1) | attention to age (1) |
| personalized dosage (1) | ***Phenotypic factors/information (19)*** |
| tailored drug dosages (6) | **unspecified (5)** |
| individualized drug dosages (3) | based on phenotyping (2) |
| individualized dosing regimens (1) | use phenotypic profile (1) |
| targeted cure (1) | based on clinical phenotypes (1) |
| individualized disease management (2) | inside into phenotypic characteristics (1) |
| using tailored therapeutic regimens (1) | **referring to gender (4)** |
| decisions tailored to patients specific wants and  needs (1) | gender (2) |
| **stratified therapeutic measure (18)** | consider gender (1) |
| stratifying treatment (2) | attention to gender (1) |
| stratifying patients (16) | **referring to weight (3)** |
| **improvement of therapy/therapeutic**  **measures (293)** | consider weight (1) |
| *unspecified (41)* | based on weight (1) |
| most appropriate treatment (1) | based on body surface (1) |
| provide optimal treatment (1) | **referring to membership of a certain**  **(ethnic) group (7)** |
| optimal patient treatment (2) | ethnicity (1) |
| improve treatment (3) | targeted for ethnic groups (1) |
| optimize treatment (1) | attention to ethnic background (1) |
| optimized therapy (7) | take ethnicity into account (1) |
| improve therapy (2) | targeted for familial groups (1) |
| better utilize drugs (1) | targeted for demographic groups (2) |
| optimize prescription (1) | ***Personal preferences (4)*** |
| optimize drug therapies (2) | consider preferences of patient (1) |
| maximize cure (1) | use patients own preferences (1) |
| accelerate the cure of disease (1) | consider beliefs of patient (1) |
| optimal pharmacotherapy (1) | personal preferences (1) |
| receive most appropriate pharmacotherapy (1) | ***Behaviour (22)*** |
| improved management of disease (2) | **unspecified (8)** |
| optimize therapeutic management (1) | behavioral (3) |
| improve treatment outcome (6) | behavioral data (1) |
| optimal outcome in disease management (1) | based on patients health behavior (1) |
| maximize (positive) treatment outcomes (2) | behavioral factors (1) |
| maximize (treatment) benefits (4) | through targeted behavior modification (1) |
| *choice of treatment (32)* | drawn from information about differences in  behavioral determinants (1) |
| most appropriate therapeutic intervention (1) | **referring to nutrition (5)** |
| determine optimal drug dosage (3) | diet (2) |
| select best dose (2) | consider diet (1) |
| select the best drug (5) | identify nutritional factors (1) |
| most appropriate combination of drugs (2) | nutritional state (1) |
| identify most appropriate drug (2) | **referring to lifestyle (6)** |
| find the optimal treatment program (1) | lifestyle changes (1) |
| select the best therapy (1) | lifestyle state (1) |
| find most appropriate therapy (2) | based on lifestyle (1) |
| decisions about the best course of treatment (1) | use information about lifestlye (1) |
| optimize treatment dosage (2) | give (tailored) lifestyle advice (1) |
| optimized treatment selection (1) | exercise (1) |
| refined treatment decisions (1) | **referring to toxins (3)** |
| determine the optimal treatment (strategy) (3) | exposure to tobacco (1) |
| select most appropriate treatment (1) | exposure to alcohol (1) |
| determine most appropriate treatment regimen  (1) | exposure to drugs (1) |
| determine the best treatment strategy (1) | **D. Usage of (Specific) Technology (51)** |
| select best medication for the patient (1) | ***Unspecified (17)*** |
| delineating optimal therapeutic strategies (1) | using the most advanced and proper scientific and technological tools (1) |
| *effectiveness/efficacy (92)* | focused examination and specific laboratory and medical tests (1) |
| most effective treatment (2) | using laboratory tests (1) |
| maximize response to medication/drugs (3) | based on laboratory test resulsts (1) |
| maximize the likelihood of treatment response  (1) | use diagnostic tests (1) |
| improve patients response rate (4) | use screening tests (1) |
| improve therapeutic response (1) | technologies to identify human variability (1) |
| optimizing drug response (1) | where imaging data could be analyzed in real-time (1) |
| better response to treatment (1) | neuroimaging (1) |
| more effective treatment (7) | based on imaging (1) |
| more effective treatment approaches (1) | use of custom-tailored tools (1) |
| improve efficiacy of treatment (1) | use of custom-tailored technologies (1) |
| more effective therapy (5) | use nanotechnology (1) |
| improving therapeutic effectiveness (1) | using modern biotechnology (2) |
| assign more effective therapies (2) | using noninvasive disease monitoring procedures (1) |
| provide most effective therapeutic strategy (1) | appropriate disease screening (1) |
| maximize therapeutic effect (4) | ***(New) technology for genetic analysis (30)*** |
| improve efficiacy of therapy (2) | advances in genome science and technology (1) |
| maximize therapeutic efficacy (1) | based on functional and molecular imaging (1) |
| more precise therapies (1) | genome-wide gene expression microarray analysis (1) |
| more selective therapy (1) | gene-sequencing technology (1) |
| maximize therapeutic benefit (1) | use of DNA microarray (1) |
| more effective pharmacotherapy (3) | advances in DNA sequencing technology (1) |
| more effective drugs (2) | based on DNA sequenzing methods (1) |
| most effective drugs (2) | whole-genome sequencing (2) |
| prescribe more effective rugs (1) | anticipate Genome-wide screening (1) |
| optimize drug efficiacy (1) | integrating genomics technologies (1) |
| maximize drug efficiacy (2) | test for gene expression (1) |
| improve efficiacy of drug (2) | harnessing genomics and proteomics technologies (1) |
| more effective drug dosages (1) | application of high-throughput genomic technologies in individual patients (1) |
| most effective drug dosage (1) | use genomic research breakthroughs (1) |
| prescription of most effective medication (1) | do genetic testing (1) |
| drive the most effective clinical decisions (1) | use of genetic testing (7) |
| more predictable treatment (1) | require routine genetic testing (1) |
| improved treatment outcome (2) | genetic tools (1) |
| achieve better/improve clinical outcomes (5) | incorporation of genetic testing (1) |
| improve therapeutic outcome (1) | use of single-gene pretesting (1) |
| provide improved clinical outcome (1) | analysis of genetic testing of polymorphisms (1) |
| obtain optimal clinical effect (1) | by leveraging modern genotyping technology (1) |
| achieve optimal medical outcome (4) | test for genetic variation (1) |
| get best medical outcome (1) | ***Information technology (4)*** |
| achieve the best possible medical outcome (2) | use bioinformatics (1) |
| optimize treatment outcome (1) | by leveraging health information technologies (1) |
| achieve maximum therapeutic benefit (1) | use advances in health information technology (1) |
| obtain the best therapeutic results (1) | driven by information technologies (1) |
| optimize medical outcome (2) |  |
| improve recovery (1) |  |
| optimal therapeutic success (1) |  |
| increase patient survival (4) |  |
| improve survival outcomes (1) |  |
| optimal therapeutic benefit (1) |  |
| more compliance (2) |  |
| higher compliance with drug regimens (1) |  |
| *efficiency (2)* |  |
| more efficient medication (2) |  |
| *timing (4)* |  |
| early treatment (1) |  |
| early therapeutic intervention (1) |  |
| earlier interventions (1) |  |
| treat diseases before symptom onset (1) |  |
| *safety (83)* |  |
| safest treatment (1) |  |
| guarantee safer treatment (1) |  |
| provide safest therapeutic strategy (1) |  |
| safer medication (2) |  |
| improve safety of pharmaceutical therapies (2) |  |
| safer drugs (2) |  |
| prescribe safer drugs (1) |  |
| maximize drug safety (2) |  |
| improve safety of drug (1) |  |
| safer drug dosages (1) |  |
| reduce (treatment) toxicity (10) |  |
| minimize treatment toxicity (1) |  |
| limit treatment toxicity (1) |  |
| minimize adverse drug reactions/effects (26) |  |
| reduce adverse side effects of treatment (10) |  |
| spare side effects of drugs (3) |  |
| avoid adverse drug reactions (9) |  |
| fewer treatment side effects (1) |  |
| minimize drug interaction errors (1) |  |
| reduce complications in treatment (2) |  |
| improve drug safety (2) |  |
| improve/better drug tolerability (2) |  |
| limit side effects (1) |  |
| *tailoring treatment measures (39)* |  |
| perfectly tailored treatments (1) |  |
| more tailored treatment (1) |  |
| tailor therapeutics more coherently (1) |  |
| more targeted drugs (1) |  |
| more specific drugs (1) |  |
| better individualized treatment (1) |  |
| optimally match patient with treatment (1) |  |
| medication best suited for an individual (3) |  |
| tailor most suitable pharmacotherapy (1) |  |
| match each patient with most suitable drug (2) |  |
| best treatment for individual (3) |  |
| identify best therapy for individual (1) |  |
| prescription of specific therapeutics best suited  for individual (8) |  |
| prescription of specific therapeutics best suited  for an individual’s genotype (1) |  |
| identify therapy best suited for individual (3) |  |
| find best treatment for individual (1) |  |
| identify best treatment for individual (1) |  |
| treatment best suited for an individual (2) |  |
| select treatment best suited for individual (1) |  |
| select treatment best suited to individual  phenotype (1) |  |
| prescription of specific treatments best suited  for an individual’s genotype (1) |  |
| facilitate individualized curative therapies (1) |  |
| select the best medicine to suit each patient (1) |  |
| harnessing the best treatments for an individual  (1) |  |
|  |  |
| **4 IMPROVED HEALTH (10)** |  |
| advancing health (1) |  |
| optimize population health (1) |  |
| improving health (3) |  |
| achieve optimal health (1) |  |
| optimize individuals health (4) |  |
|  |  |
|  |  |
| **5 FURTHER ENDS (Unspecified) (77)** |  |
| ***Unspecified (11)*** |  |
| ensure activity (2) |  |
| decision making (4) |  |
| tailored wellness plan for individual (1) |  |
| incorporate management of personal data and clinical information (1) |  |
| Increased participation of the patient in health care (1) |  |
| minimize what patients most fear (1) |  |
| limit resistance (1) |  |
| ***(Tailored) patient management (3)*** |  |
| personalizing management regimens for patients (1) |  |
| patient management (1) |  |
| direct patient management. (1) |  |
| ***Stratification (1)*** |  |
| use stratification (1) |  |
| ***Reduce/control costs(30)*** |  |
| control costs (2) |  |
| affordable social costs (2) |  |
| reasonable costs (1) |  |
| cost-effectiveness (1) |  |
| reduce/lower costs (14) |  |
| spare costs (4) |  |
| save money (2) |  |
| limit costs (1) |  |
| avoid costs (3) |  |
| ***Improved effectiveness/efficacy(24)*** |  |
| assuring maximal efficiacy (1) |  |
| maintain efficiacy (1) |  |
| more effective (1) |  |
| maximize effectiveness (2) |  |
| improve effectiveness (3) |  |
| increase efficiacy (6) |  |
| maximize efficiacy (4) |  |
| improve/better efficiacy (5) |  |
| improved efficacy profile (1) |  |
| ***Improved timing(1)*** |  |
| determine optimal timing (1) |  |
| ***Improved safety (3)*** |  |
| maximize safety (1) |  |
| better safety (1) |  |
| improved safety profile (1) |  |
| ***Improved quality of life (4)*** |  |
| improve quality of life (4) |  |
