# Supplementary material for: What is personalized medicine: sharpening a vague term based on a systematic literature review
Source: BMC Med Ethics. 2013 Dec 21;14:55. doi: 10.1186/1472-6939-14-55 (PMC3878093; doi:10.1186/1472-6939-14-55)
Supplement: Additional file 2 — Searched Papers Containing PM in Title/Abstract. [file 1472-6939-14-55-S2.doc]

**Additional file 2 Searched Papers Containing PM in Title/Abstract 2012**

1. Abbate R, Cioni G, Marcucci R, Fatini C, Gensini GF. [Antithrombotic drugs in women: should we worry more about bleeding?]. G Ital Cardiol (Rome). 2012 Jun;13(6):407-13. doi: 10.1714/1073.11758. Italian.
2. Agúndez JA, Del Barrio J, Padró T, Stephens C, Farré M, Andrade RJ, Badimon L, García-Martín E, Vilahur G, Lucena MI. Trends in qualifying biomarkers in drug safety. Consensus of the 2011 meeting of the spanish society of clinical pharmacology. Front Pharmacol. 2012;3:2.
3. Agarwal A. Do companion diagnostics make economic sense for drug developers? N Biotechnol. 2012 May 1.
4. Agmon-Levin N, Mosca M, Petri M, Shoenfeld Y. Systemic lupus erythematosus one disease or many? Autoimmun Rev. 2012 Jun;11(8):593-5.
5. Allen TC. State pathology societies and the future of pathology. Arch Pathol Lab Med. 2012 Feb;136(2):139.
6. Álvarez P, Marchal JA, Boulaiz H, Carrillo E, Vélez C, Rodríguez-Serrano F, Melguizo C, Prados J, Madeddu R, Aranega A. 5-Fluorouracil derivatives: a patent review. Expert Opin Ther Pat. 2012 Feb;22(2):107-23. **NO ACCESS**
7. Amaria RN, Lewis KD, Jimeno A. Vemurafenib: the road to personalized medicine in melanoma. Drugs Today (Barc). 2012 Feb;48(2):109-18. Review. **NO ACCESS**
8. Amstad E, Reimhult E. Nanoparticle actuated hollow drug delivery vehicles. Nanomedicine (Lond). 2012 Jan;7(1):145-64. Review.
9. Anderson ER, Burmester JK, Caldwell MD. Evaluation of a Mitochondrial DANN Mutation in Maternally Inherited and Sporadic Cases of Dupuytren's Disease. Clin Med Res. 2012 May 25. **NO ACCESS**
10. Andoh A. Recent approaches of personalized medicine for inflammatory bowel disease. Nihon Shokakibyo Gakkai Zasshi. 2012 Mar;109(3):355-63. **NO ACCESS**
11. Andre F, Nowak F, Arnedos M, Lacroix L, Viens P, Calvo F. Biomarker discovery, development, and implementation in France: a report from the French National Cancer Institute and cooperative groups. Clin Cancer Res. 2012 Mar 15;18(6):1555-60.
12. Ansari D, Chen BC, Dong L, Zhou MT, Andersson R. Pancreatic cancer: translational research aspects and clinical implications. World J Gastroenterol. 2012 Apr 7;18(13):1417-24.
13. Ardini E, Galvani A. ALK Inhibitors, a Pharmaceutical Perspective. Front Oncol. 2012;2:17.
14. Arnedos M, André F, Farace F, Lacroix L, Besse B, Robert C, Soria JC, Eggermont AM. The challenge to bring personalized cancer medicine from clinical trials into routine clinical practice: the case of the Institut Gustave Roussy. Mol Oncol. 2012 Apr;6(2):204-10. **NO ACCESS**
15. Ashbee HR, Gilleece MH. Has the era of individualised medicine arrived for antifungals? A review of antifungal pharmacogenomics. Bone Marrow Transplant.2012 Jul;47(7):881-94. doi: 10.1038/bmt.2011.146.
16. Audenet F, Yates DR, Cancel-Tassin G, Cussenot O, Rouprêt M. Genetic pathways involved in carcinogenesis of clear cell renal cell carcinoma: genomics towards personalized medicine. BJU Int. 2012 Jun;109(12):1864-70. doi: 10.1111/j.1464-410X.2011.10661.x.
17. Aurand TC, Russell MS, March JC. Synthetic signaling networks for therapeutic applications. Curr Opin Biotechnol. 2012 Jan 27.
18. Ausborn NL, Le QT, Bradley JD, Choy H, Dicker AP, Saha D, Simko J, Story MD, Torossian A, Lu B. Molecular profiling to optimize treatment in non-small cell lung cancer: a review of potential molecular targets for radiation therapy by the translational research program of the radiation therapy oncology group. Int J Radiat Oncol Biol Phys. 2012 Jul 15;83(4):e453-64.
19. Aw W, Lezhava A, Andoh A, Tanaka H, Hayashizaki Y, Ishikawa T. The SmartAmp method: Rapid Detection of SNPs in Thiopurine S-Methyltransferase and ABC Transporters ABCC4 and ABCG2. Curr Drug Metab. 2012 May 16. **NO ACCESS**
20. Ballestrero A, Garuti A, Cirmena G, Rocco I, Palermo C, Nencioni A, Scabini S, Zoppoli G, Parodi S, Patrone F. Patient-tailored treatments with anti-EGFR monoclonal antibodies in advanced colorectal cancer: KRAS and beyond. Curr Cancer Drug Targets. 2012 May;12(4):316-28. **NO ACCESS**
21. Bandla S, Pennathur A, Luketich JD, Beer DG, Lin L, Bass AJ, Godfrey TE, Litle VR. Comparative genomics of esophageal adenocarcinoma and squamous cell carcinoma. Ann Thorac Surg. 2012 Apr;93(4):1101-6.
22. Bartlett G, Zgheib N, Manamperi A, Wang W, Hizel C, Kahveci R, Yazan Y. Pharmacogenomics in Primary Care: A Crucial Entry Point for Global Personalized Medicine? Curr Pharmacogenomics Person Med. 2012;10(2):101-105.
23. Barzegari A, Saei AA. Designing probiotics with respect to the native microbiome. Future Microbiol. 2012 May;7(5):571-5. **NO ACCESS**
24. Basu S. The scope and potentials of functional radionuclide imaging towards advancing personalized medicine in oncology: emphasis on PET-CT. Discov Med. 2012 Jan;13(68):65-73. Review. **NO ACCESS**
25. Baudhuin LM, Donato LJ, Uphoff TS. How novel molecular diagnostic technologies and biomarkers are revolutionizing genetic testing and patient care. Expert Rev Mol Diagn. 2012 Jan;12(1):25-37. doi: 10.1586/ERM.11.85. **NO ACCESS**
26. Baum RP, Kulkarni HR, Carreras C. Peptides and receptors in image-guided therapy: theranostics for neuroendocrine neoplasms. Semin Nucl Med. 2012 May;42(3):190-207.
27. Benichou J, Ben-Hamo R, Louzoun Y, Efroni S. Rep-Seq: uncovering the immunological repertoire through next-generation sequencing. Immunology. 2012 Mar;135(3):183-91. doi: 10.1111/j.1365-2567.2011.03527.x. Review.
28. Berman DM, Bosenberg MW, Orwant RL, Thurberg BL, Draetta GF, Fletcher CD, Loda M. Investigative pathology: leading the post-genomic revolution. Lab Invest. 2012 Jan;92(1):4-8. doi: 10.1038/labinvest.2011.147.
29. Bernet L, Martinez Benaclocha M, Castera C, Cano Muñoz R, Sevilla F, Alba J, de Dios Barranco J, Cordoba A, Garcia-Caballero T, Hardisson D, de Francisco Hernandez JM, Lazaro JM, Polo L, Riu F, Rezola R, Rojo F, Ruiz I, Hernándiz A, de la Cámara de Las Heras JM, Coupe VM. mRNA in situ hybridization (HistoSonda): a new diagnostic tool for HER2-status in breast cancer-a multicentric Spanish study. Diagn Mol Pathol. 2012 Jun;21(2):84-92.
30. Berry DA. Bayesian approaches for comparative effectiveness research. Clin Trials. 2012 Feb;9(1):37-47.
31. Besse B, Soria JC. [Personalized medicine in oncology: from hope to reality]. Rev Prat. 2012 Feb;62(2):245-6. French.
32. Black A, Morris D. Personalized medicine in metastatic non-small-cell lung cancer: promising targets and current clinical trials. Curr Oncol. 2012 Jun;19(Supplement 1):S73-S85.
33. Blay JY, Lacombe D, Meunier F, Stupp R. Personalised medicine in oncology: questions for the next 20 years. Lancet Oncol. 2012 May;13(5):448-9.
34. Bochud M, Fellmann F, Guessous I. [Are genetic tests useful for cardiovascular prevention?]. Rev Med Suisse. 2012 Mar 7;8(331):519-20, 522-4. French.
35. Borrebaeck CA. Viewpoints in Clinical Proteomics - when will proteomics deliver clinically useful information? Proteomics Clin Appl. 2012 Jun 14. doi: 10.1002/prca.201200020. **NO ACCESS**
36. Brüggenjürgen B, Kornbluth L, Ferrara JV, Willich SN. [Clinical and health economic challenges of personalized medicine]. Bundesgesundheitsblatt Gesundheitsforschung Gesundheitsschutz. 2012 May;55(5):710-4. German.
37. Braat H, Bruno M, Kuipers EJ, Peppelenbosch MP. Pancreatic cancer: promise for personalised medicine? Cancer Lett. 2012 May 1;318(1):1-8.
38. Braun A, Halwachs B, Geier M, Weinhandl K, Guggemos M, Marienhagen J, Ruff AJ, Schwaneberg U, Rabin V, Torres Pazmiño DE, Thallinger GG, Glieder A. MuteinDB: the mutein database linking substrates, products and enzymatic reactions directly with genetic variants of enzymes. Database (Oxford). 2012 Jun 21;2012:bas028.
39. Brenner MK. Personalized medicine: words that mean just what you choose? Mol Ther. 2012 Feb;20(2):241-2. doi: 10.1038/mt.2011.311.
40. Brightling CE, Gupta S, Gonem S, Siddiqui S. Lung damage and airway remodelling in severe asthma. Clin Exp Allergy. 2012 May;42(5):638-49. doi: 10.1111/j.1365-2222.2011.03917.x.
41. Brindle K. Watching tumours gasp and die with MRI: the promise of hyperpolarised 13C MR spectroscopic imaging. Br J Radiol. 2012 Jun;85(1014):697-708. Epub 2012 Apr 11. Review.
42. Brown NJ. Patient-oriented research in the era of personalized medicine. Clin Transl Sci. 2012 Apr;5(2):119-20. doi: 10.1111/j.1752-8062.2011.00394.x. **NO ACCESS**
43. Buckland M. Immunodeficiency: updating the personalized medicine approach to diagnostics and therapeutics. Expert Rev Clin Immunol. 2012 Mar;8(3):223-5.
44. Buford TW, Pahor M. Making preventive medicine more personalized: Implications for exercise-related research. Prev Med. 2012 Jul;55(1):34-6.
45. Burckart GJ, Green DJ. The personalized medicine revolution: Getting it right for children. Pediatr Transplant. 2012 Jan 9. doi: 10.1111/j.1399-3046.2011.01638.x.
46. Buriani A, Garcia-Bermejo ML, Bosisio E, Xu Q, Li H, Dong X, Simmonds MS, Carrara M, Tejedor N, Lucio-Cazana J, Hylands PJ. Omic techniques in systems biology approaches to traditional Chinese medicine research: present and future. J Ethnopharmacol. 2012 Apr 10;140(3):535-44.
47. Buyx AM, Strech D, Schmidt H. [Ethical issues raised by direct-to-consumer personal genome analysis and whole body scans: discussion and contextualisation of a report by the Nuffield Council on Bioethics]. Z Evid Fortbild Qual Gesundhwes. 2012;106(1):29-39. German.
48. Cantor CR. Company profile: Sequenom, Inc. Pharmacogenomics. 2012 Apr;13(5):529-31.
49. Capon F, Burden AD, Trembath RC, Barker JN. Psoriasis and other complex trait dermatoses: from Loci to functional pathways. J Invest Dermatol. 2012 Mar;132(3 Pt 2):915-22. doi: 10.1038/jid.2011.395.
50. Carlson B. In Search of the Perfect Business Model: As personalized medicine moves into the mainstream, makers of diagnostics must face a new economic reality. How to develop a value proposition in a healthcare market that is becoming increasingly elastic? Biotechnol Healthc. 2012 Spring;9(1):20-3.
51. Carter CS, Barch DM; CNTRICS Executive Committee. Imaging biomarkers for treatment development for impaired cognition: report of the sixth CNTRICS meeting: Biomarkers recommended for further development. Schizophr Bull. 2012 Jan;38(1):26-33.
52. Carter RL, Chan AW. Pluripotent Stem Cells Models for Huntington's Disease: Prospects and Challenges. J Genet Genomics. 2012 Jun 20;39(6):253-9. **NO ACCESS**
53. Caux C, Zitvogel L. Recent successes of cancer immunotherapy: a new dimension in personalized medicine? Target Oncol. 2012 Mar;7(1):1-2. **NO ACCESS**
54. Cecic IK, Li G, MacAulay C. Technologies supporting analytical cytology: clinical, research and drug discovery applications. J Biophotonics. 2012 Apr;5(4):313-26. doi: 10.1002/jbio.201100093. **NO ACCESS**
55. Chae H, Lee S, Park SH, Jang E, Lee SJ. Development and validation of a personality assessment instrument for traditional korean medicine: sasang personality questionnaire. Evid Based Complement Alternat Med. 2012;2012:657013..
56. Chang WY, Garcha K, Manias JL, Stanford WL. Deciphering the complexities of human diseases and disorders by coupling induced-pluripotent stem cells and systems genetics. Wiley Interdiscip Rev Syst Biol Med. 2012 Jul;4(4):339-50. doi: 10.1002/wsbm.1170.
57. Chen C, Peng J, Sun SR, Peng CW, Li Y, Pang DW. Tapping the potential of quantum dots for personalized oncology: current status and future perspectives. Nanomedicine (Lond). 2012 Mar;7(3):411-28.
58. Chen R, Mias GI, Li-Pook-Than J, Jiang L, Lam HY, Chen R, Miriami E, Karczewski KJ, Hariharan M, Dewey FE, Cheng Y, Clark MJ, Im H, Habegger L, Balasubramanian S, O'Huallachain M, Dudley JT, Hillenmeyer S, Haraksingh R, Sharon D, Euskirchen G, Lacroute P, Bettinger K, Boyle AP, Kasowski M, Grubert F, Seki S, Garcia M, Whirl-Carrillo M, Gallardo M, Blasco MA, Greenberg PL, Snyder P, Klein TE, Altman RB, Butte AJ, Ashley EA, Gerstein M, Nadeau KC, Tang H, Snyder M. Personal omics profiling reveals dynamic molecular and medical phenotypes. Cell. 2012 Mar 16;148(6):1293-307.
59. Cheng CJ, Slack FJ. The Duality of OncomiR Addiction in the Maintenance and Treatment of Cancer. Cancer J. 2012 May;18(3):232-7. **NO ACCESS**
60. Cheng L, Alexander RE, Maclennan GT, Cummings OW, Montironi R, Lopez-Beltran A, Cramer HM, Davidson DD, Zhang S. Molecular pathology of lung cancer: key to personalized medicine. Mod Pathol. 2012 Mar;25(3):347-69. doi: 10.1038/modpathol.2011.215. Epub 2012 Jan 27. Review. **NO ACCESS**
61. Cheng S, Koch WH, Wu L. Co-development of a companion diagnostic for targeted cancer therapy. N Biotechnol. 2012 Feb 25.
62. Chiu PH, Hsieh HY, Wang SC. Prescriptions of traditional Chinese medicine are specific to cancer types and adjustable to temperature changes. PLoS One. 2012;7(2):e31648.
63. Choi W, Jung H, Kim K, Lee S, Yoon S, Park J, Kim S, Cheon S, Eo W, Lee S. Rhus verniciflua stokes against advanced cancer: a perspective from the Korean Integrative Cancer Center. J Biomed Biotechnol. 2012;2012:874276.
64. Clancy JP, Jain M. Personalized Medicine in Cystic Fibrosis: Dawning of a New Era. Am J Respir Crit Care Med. 2012 Jun 21.
65. Clausson CM, Grundberg I, Weibrecht I, Nilsson M, Söderberg O. Methods for analysis of the cancer microenvironment and their potential for disease prediction, monitoring and personalized treatments. EPMA J. 2012 Mar 22;3(1):7.
66. Close SL. Pharmacogenetics and pharmacogenomics of thienopyridines: clinically relevant? Fundam Clin Pharmacol. 2012 Feb;26(1):19-26. doi: 10.1111/j.1472-8206.2011.00983.x. Epub 2011 Aug 31. Review.
67. Cohen J, Wilson A, Manzolillo K. Clinical and economic challenges facing pharmacogenomics. Pharmacogenomics J. 2012 Jan 10. doi: 10.1038/tpj.2011.63. **NO ACCESS**
68. Collier R. A race-based detour to personalized medicine. CMAJ. 2012 Apr 17;184(7):E351-3.
69. Corbacioglu S. Early phase clinical trials in pediatric hematology and oncology. Klin Padiatr. 2012 Apr;224(3):197-200. **NO ACCESS**
70. Cordero F, Beccuti M, Donatelli S, Calogero RA. Large disclosing the nature of computational tools for the analysis of next generation sequencing data. Curr Top Med Chem. 2012 Jun 1;12(12):1320-30. **NO ACCESS**
71. Cordero P, Ashley EA. Whole-Genome Sequencing in Personalized Therapeutics. Clin Pharmacol Ther. 2012 May 2. doi: 10.1038/clpt.2012.51
72. Couch RD, Mott BT. Personalized medicine: changing the paradigm of drug development. Methods Mol Biol. 2012;823:367-78. **NO ACCESS**
73. Cox AD, Der CJ. The RAF inhibitor paradox revisited. Cancer Cell. 2012 Feb 14;21(2):147-9.
74. Cun Y, Fröhlich H. Prognostic gene signatures for patient stratification in breast cancer - accuracy, stability and interpretability of gene selection approaches using prior knowledge on protein-protein interactions. BMC Bioinformatics. 2012 May 1;13(1):69.
75. D'Souza-Schorey C, Clancy JW. Tumor-derived microvesicles: shedding light on novel microenvironment modulators and prospective cancer biomarkers. Genes Dev. 2012 Jun 15;26(12):1287-99. **NO ACCESS**
76. Dammann M, Weber F. Personalized medicine: caught between hope, hype and the real world. Clinics (Sao Paulo). 2012;67 Suppl 1:91-7.
77. Dancey J. Genomics, personalized medicine and cancer practice. Clin Biochem. 2012 Apr;45(6):379-81.
78. Davies BR, Greenwood H, Dudley P, Crafter C, Yu DH, Zhang J, Li J, Gao B, Ji Q, Maynard J, Ricketts SA, Cross D, Cosulich S, Chresta CC, Page K, Yates J, Lane C, Watson R, Luke R, Ogilvie D, Pass M. Preclinical pharmacology of AZD5363, an inhibitor of AKT: pharmacodynamics, antitumor activity, and correlation of monotherapy activity with genetic background. Mol Cancer Ther. 2012 Apr;11(4):873-87. **NO ACCESS**
79. de Albuquerque A, Kaul S, Breier G, Krabisch P, Fersis N. Multimarker Analysis of Circulating Tumor Cells in Peripheral Blood of Metastatic Breast Cancer Patients: A Step Forward in Personalized Medicine. Breast Care (Basel). 2012 Feb;7(1):7-12.
80. de Leon J. Evidence-based medicine versus personalized medicine: are they enemies? J Clin Psychopharmacol. 2012 Apr;32(2):153-64. **NO ACCESS**
81. de Miranda DM, Mamede M, de Souza BR, de Almeida Barros AG, Magno LA, Alvim-Soares A Jr, Rosa DV, de Castro CJ Jr, Malloy-Diniz L, Gomez MV, De Marco LA, Correa H, Romano-Silva MA. Molecular medicine: a path towards a personalized medicine. Rev Bras Psiquiatr. 2012 Mar;34(1):82-91.
82. De Velasco MA, Uemura H. Preclinical Remodeling of Human Prostate Cancer through the PTEN/AKT Pathway. Adv Urol. 2012;2012:419348.
83. Debergh I, Vanhove C, Ceelen W. Innovation in cancer imaging. Eur Surg Res. 2012;48(3):121-30. Epub 2012 Apr 24.
84. Desiere F, Romano Spica V. Personalised Medicine in 2012: Editorial to the Special Issue of New Biotechnology on "MOLECULAR DIAGNOSTICS & PERSONALISED MEDICINE". N Biotechnol. 2012 Apr 5.
85. Dhamne SA, Brown RE, Covinsky MH, Dhamne C, Eldin K, Tatevian N. Clear Cell Sarcoma of Kidney: Morphoproteomic Analysis Reveals Genomic Correlates and Therapeutic Options. Pediatr Dev Pathol. 2012 Jan 18. **NO ACCESS**
86. Diaz FJ, Cogollo MR, Spina E, Santoro V, Rendon DM, de Leon J. Drug dosage individualization based on a random-effects linear model. J Biopharm Stat. 2012;22(3):463-84. **NO ACCESS**
87. Do H, Dobrovic A. Dramatic reduction of sequence artefacts from DNA isolated from formalin-fixed cancer biopsies by treatment with uracil- DNA glycosylase. Oncotarget. 2012 May;3(5):546-58.
88. Drews K, Jozefczuk J, Prigione A, Adjaye J. Human induced pluripotent stem cells--from mechanisms to clinical applications. J Mol Med (Berl). 2012 Jul;90(7):735-45.
89. Dry S, Grody WW, Papagni P. Stuck between a scalpel and a rock, or molecular pathology and legal-ethical issues in use of tissues for clinical care and research: what must a pathologist know? Am J Clin Pathol. 2012 Mar;137(3):346-55. Review.
90. Dutton-Regester K, Hayward NK. Reviewing the somatic genetics of melanoma: from current to future analytical approaches. Pigment Cell Melanoma Res. 2012 Mar;25(2):144-54. doi: 10.1111/j.1755-148X.2012.00975.x.
91. Eckhart AD, Beebe K, Milburn M. Metabolomics as a key integrator for "omic" advancement of personalized medicine and future therapies. Clin Transl Sci. 2012 Jun;5(3):285-8. doi: 10.1111/j.1752-8062.2011.00388.x. **NO ACCESS**
92. Edlund K, Larsson O, Ameur A, Bunikis I, Gyllensten U, Leroy B, Sundström M, Micke P, Botling J, Soussi T. Data-driven unbiased curation of the TP53 tumor suppressor gene mutation database and validation by ultradeep sequencing of human tumors. Proc Natl Acad Sci U S A. 2012 Jun 12;109(24):9551-6.
93. Eichler GS. Bioinformatics/biostatistics: microarray analysis. Methods Mol Biol. 2012;823:347-58. **NO ACCESS**
94. Eisfeld AK, Marcucci G, Liyanarachchi S, Döhner K, Schwind S, Maharry K, Leffel B, Döhner H, Radmacher MD, Bloomfield CD, Tanner SM, de la Chapelle A. Heritable polymorphism predisposes to high BAALC expression in acute myeloid leukemia. Proc Natl Acad Sci U S A. 2012 Apr 24;109(17):6668-73.
95. Engel J, Emons G, Pinski J, Schally AV. AEZS-108 : a targeted cytotoxic analog of LHRH for the treatment of cancers positive for LHRH receptors. Expert Opin Investig Drugs. 2012 Jun;21(6):891-9. **NO ACCESS**
96. Engel JB, Schally AV, Buchholz S, Seitz S, Emons G, Ortmann O. Targeted chemotherapy of endometrial, ovarian and breast cancers with cytotoxic analogs of luteinizing hormone-releasing hormone (LHRH). Arch Gynecol Obstet. 2012 Aug;286(2):437-42.
97. Erickson HS. Measuring molecular biomarkers in epidemiologic studies: laboratory techniques and biospecimen considerations. Stat Med. 2012 May 17. doi: 10.1002/sim.4485.
98. Fahey JW, Talalay P, Kensler TW. Notes from the field: "green" chemoprevention as frugal medicine. Cancer Prev Res (Phila). 2012 Feb;5(2):179-88. Review. **NO ACCESS**
99. Fan J, Deng X, Gallagher JW, Huang H, Huang Y, Wen J, Ferrari M, Shen H, Hu Y. Monitoring the progression of metastatic breast cancer on nanoporous silica chips. Philos Transact A Math Phys Eng Sci. 2012 May 28;370(1967):2433-47.
100. Farra N, Manickaraj AK, Ellis J, Mital S. Personalized Medicine in the Genomics Era: highlights from an international symposium on childhood heart disease. Future Cardiol. 2012 Mar;8(2):157-60.
101. Feldman EA. The Genetic Information Nondiscrimination Act (GINA): public policy and medical practice in the age of personalized medicine. J Gen Intern Med. 2012 Jun;27(6):743-6.
102. Finn L, Tan W. Proof of concept to clinical confirmation: evolving clinical trial designs for targeted agents. ISRN Oncol. 2012;2012:478607.
103. Fleck LM. Pharmacogenomics and personalized medicine: wicked problems, ragged edges and ethical precipices. N Biotechnol. 2012 Mar 14.
104. Floyd CN. Pharmacogenomic personalised medicine for antiplatelet agents is on the horizon. BMJ. 2012 Jun 19;344:e4198. doi: 10.1136/bmj.e4198
105. Foran JM. Targeted therapy of acute myeloid leukemia in 2012: towards individualized therapy. Hematology. 2012 Apr;17 Suppl 1:S137-40. **NO ACCESS**
106. Formentini I, Bobadilla M, Haefliger C, Hartmann G, Loghman-Adham M, Mizrahi J, Pomposiello S, Prunotto M, Meier M. Current drug development challenges in chronic kidney disease (CKD)--identification of individualized determinants of renal progression and premature cardiovascular disease (CVD). Nephrol Dial Transplant. 2012 Jun 25.
107. Frank E, Kupfer DJ, Rucci P, Lotz-Wallace M, Levenson J, Fournier J, Kraemer HC. Simultaneous evaluation of the harms and benefits of treatments in randomized clinical trials: demonstration of a new approach. Psychol Med. 2012 Apr;42(4):865-73.
108. Friede T, Parsons N, Stallard N. A conditional error function approach for subgroup selection in adaptive clinical trials. Stat Med. 2012 Aug 3. doi: 10.1002/sim.5541.
109. Frueh FW. On rat poison and human medicines: personalizing warfarin therapy. Trends Mol Med. 2012 Apr;18(4):201-5.
110. Fueldner C, Mittag A, Knauer J, Biskop M, Hepp P, Scholz R, Wagner U, Sack U, Emmrich F, Tárnok A, Lehmann J. Identification and evaluation of novel synovial tissue biomarkers in rheumatoid arthritis by laser scanning cytometry. Arthritis Res Ther. 2012 Jan 17;14(1):R8.
111. Galetta D, Rossi A, Pisconti S, Colucci G. The emerging role of ALK inhibitors in the treatment of advanced non-small cell lung cancer. Expert Opin Ther Targets. 2012 Apr;16 Suppl 2:S45-54. **NO ACCESS**
112. Gandhi L, Jänne PA. Crizotinib for ALK-Rearranged Non-Small Cell Lung Cancer: A New Targeted Therapy for a New Target. Clin Cancer Res. 2012 Jun 21.
113. Garraway LA, Jänne PA. Circumventing cancer drug resistance in the era of personalized medicine. Cancer Discov. 2012 Mar;2(3):214-26 **NO ACCESS**
114. Gerlinger M, Rowan AJ, Horswell S, Larkin J, Endesfelder D, Gronroos E, Martinez P, Matthews N, Stewart A, Tarpey P, Varela I, Phillimore B, Begum S, McDonald NQ, Butler A, Jones D, Raine K, Latimer C, Santos CR, Nohadani M, Eklund AC, Spencer-Dene B, Clark G, Pickering L, Stamp G, Gore M, Szallasi Z, Downward J, Futreal PA, Swanton C. Intratumor heterogeneity and branched evolution revealed by multiregion sequencing. N Engl J Med. 2012 Mar 8;366(10):883-92.
115. Gespach C. Guidance for life, cell death, and colorectal neoplasia by netrin dependence receptors. Adv Cancer Res. 2012;114:87-186. **NO ACCESS**
116. Gevaert O, Xu J, Hoang CD, Leung AN, Xu Y, Quon A, Rubin DL, Napel S, Plevritis SK. Non-Small Cell Lung Cancer: Identifying Prognostic Imaging Biomarkers by Leveraging Public Gene Expression Microarray Data--Methods and Preliminary Results. Radiology. 2012 Jun 21. **NO ACCESS**
117. Ghali JK. The next frontier of clinical trials: personalized medicine for devices. J Am Coll Cardiol. 2012 Apr 24;59(17):1519-20.
118. Gibeon D, Chung KF. The investigation of severe asthma to define phenotypes. Clin Exp Allergy. 2012 May;42(5):678-92. doi: 10.1111/j.1365-2222.2012.03959.x.
119. Girard N. Chemotherapy and targeted agents for thymic malignancies. Expert Rev Anticancer Ther. 2012 May;12(5):685-95.
120. Glurich I, Acharya A, Shukla S, Nycz G, Brilliant M. The oral-systemic personalized medicine model at Marshfield Clinic. Oral Dis. 2012 Feb 24. doi: 10.1111/j.1601-0825.2012.01921.x. **NO ACCESS**
121. Goldberg Y, Kosorok MR. Q-LEARNING WITH CENSORED DATA. Ann Stat. 2012 Feb 1;40(1):529-560. **NO ACCESS**
122. Gong J, Tong Y, Zhang HM, Wang K, Hu T, Shan G, Sun J, Guo AY. Genome-wide identification of SNPs in microRNA genes and the SNP effects on microRNA target binding and biogenesis. Hum Mutat. 2012 Jan;33(1):254-63. doi: 10.1002/humu.21641.
123. González-Beltrán A, Tagger B, Finkelstein A. Federated ontology-based queries over cancer data. BMC Bioinformatics. 2012 Jan 25;13 Suppl 1:S9. **NO ACCESS**
124. Gordon ES, Griffin G, Wawak L, Pang H, Gollust SE, Bernhardt BA. "It's not like judgment day": public understanding of and reactions to personalized genomic risk information. J Genet Couns. 2012 Jun;21(3):423-32.
125. Goyenvalle A, Wright J, Babbs A, Wilkins V, Garcia L, Davies KE. Engineering Multiple U7snRNA Constructs to Induce Single and Multiexon-skipping for Duchenne Muscular Dystrophy. Mol Ther. 2012 Jun;20(6):1212-21. doi: 10.1038/mt.2012.26.
126. Grade M, Wolff HA, Gaedcke J, Ghadimi BM. The molecular basis of chemoradiosensitivity in rectal cancer: implications for personalized therapies. Langenbecks Arch Surg. 2012 Apr;397(4):543-55.
127. Graham MM. Clinical molecular imaging with radiotracers: current status. Med Princ Pract. 2012;21(3):197-208. Epub 2011 Nov 30.
128. Graversen C, Olesen SS, Olesen AE, Steimle K, Farina D, Wilder-Smith OH, Bouwense SA, van Goor H, Drewes AM. The analgesic effect of pregabalin in patients with chronic pain is reflected by changes in pharmaco-EEG spectral indices. Br J Clin Pharmacol. 2012 Mar;73(3):363-72. doi: 10.1111/j.1365-2125.2011.04104.x.
129. Grayton HM, Fernandes C, Rujescu D, Collier DA. Copy number variations in neurodevelopmental disorders. Prog Neurobiol. 2012 Jul 17.
130. Greene BT, Hughes AD, King MR. Circulating Tumor Cells: The Substrate of Personalized Medicine? Front Oncol. 2012;2:69.
131. Gremese E, Carletto A, Padovan M, Atzeni F, Raffeiner B, Giardina AR, Favalli EG, Erre G, Gorla R, Galeazzi M, Foti R, Cantini F, Salvarani C, Olivieri I, Lapadula G, Ferraccioli G; for the Gruppo Italiano di Studio sulle Early Arthritis (GISEA). Obesity reduces the response rate to anti TNFŒ± in rheumatoid arthritis. an approach to a personalized medicine. Arthritis Care Res (Hoboken). 2012 Jun 21. doi: 10.1002/acr.21768. **NO ACCESS**
132. Gridelli C. Personalized medicine in the treatment of advanced nonsmall cell lung cancer: step-by-step progress. Curr Opin Oncol. 2012 Mar;24(2):115-6. **NO ACCESS**
133. Gruden K, Hren M, Herman A, Blejec A, Albrecht T, Selbig J, Bauer C, Schuchardt J, Or-Guil M, Zupančič K, Svajger U, Stabuc B, Ihan A, Kopitar AN, Ravnikar M, Kneževič M, Rožman P, Jeras M. A "crossomics" study analysing variability of different components in peripheral blood of healthy caucasoid individuals. PLoS One. 2012;7(1):e28761.
134. Guzauskas GF, Hughes DA, Bradley SM, Veenstra DL. A risk-benefit assessment of prasugrel, clopidogrel, and genotype-guided therapy in patients undergoing percutaneous coronary intervention. Clin Pharmacol Ther. 2012 May;91(5):829-37. doi: 10.1038/clpt.2011.303. **NO ACCESS**
135. Gvozdic K, Brandl EJ, Taylor DL, Müller DJ. Genetics and personalized medicine in antidepressant treatment. Curr Pharm Des. 2012 Jun 6. **NO ACCESS**
136. Halim SA, Newby LK, Ohman EM. Biomarkers in cardiovascular clinical trials: past, present, future. Clin Chem. 2012 Jan;58(1):45-53. Review.
137. Hall I. Conference scene: UK pharmacogenetics and stratified medicine network. Pharmacogenomics. 2012 Jan;13(1):19-20.
138. Haque SU, Morton D, Welch H. Biologics against cancer-specific receptors - challenges to personalised medicine from early trial results. Curr Opin Pharmacol. 2012 Aug;12(4):392-7.
139. Harrison N. Report on the pharmacogenomics and personalized medicine 2011 conference: part 1. Pharmacogenomics. 2012 Jan;13(2):141-5.
140. Hasan N, McColgan P, Bentley P, Edwards RJ, Sharma P. Towards the identification of blood biomarkers for acute stroke in humans: a comprehensive systematic review. Br J Clin Pharmacol. 2012 Aug;74(2):230-40. doi: 10.1111/j.1365-2125.2012.04212.x.
141. Hassett MJ, Silver SM, Hughes ME, Blayney DW, Edge SB, Herman JG, Hudis CA, Marcom PK, Pettinga JE, Share D, Theriault R, Wong YN, Vandergrift JL, Niland JC, Weeks JC. Adoption of gene expression profile testing and association with use of chemotherapy among women with breast cancer. J Clin Oncol. 2012 Jun 20;30(18):2218-26.
142. Haura EB. From modules to medicine: How modular domains and their associated networks can enable personalized medicine. FEBS Lett. 2012 May 7.
143. Heinzelmann E. Olten Meeting 2011 - where benign cells meet: November 23, 2011. Chimia (Aarau). 2012;66(1-2):61-3. **NO ACCESS**
144. Henney AM. The promise and challenge of personalized medicine: aging populations, complex diseases, and unmet medical need. Croat Med J. 2012 Jun 15;53(3):207-10.
145. Hewitt SM. Tissue microarrays as a tool in the discovery and validation of predictive biomarkers. Methods Mol Biol. 2012;823:201-14. **NO ACCESS**
146. Hirsh V. Future of personalized medicine in non-small-cell lung cancer. Curr Oncol. 2012 Jun;19(Supplement 1):S86.
147. Hodgson DR, Wellings R, Harbron C. Practical perspectives of personalized healthcare in oncology. N Biotechnol. 2012 Mar 15.
148. Hoelder S, Clarke PA, Workman P. Discovery of small molecule cancer drugs: successes, challenges and opportunities. Mol Oncol. 2012 Apr;6(2):155-76.
149. Hoge SK, Appelbaum PS. Ethics and neuropsychiatric genetics: a review of major issues. Int J Neuropsychopharmacol. 2012 Jan 25:1-11.
150. Hottenrott C. Personalized medicine for laparoscopic gastrectomy in gastric cancer. Surg Endosc. 2012 May 2.
151. Houtman R, de Leeuw R, Rondaij M, Melchers D, Verwoerd D, Ruijtenbeek R, Martens JW, Neefjes J, Michalides R. Serine-305 phosphorylation modulates estrogen receptor alpha binding to a coregulator peptide array, with potential application in predicting responses to tamoxifen. Mol Cancer Ther. 2012 Apr;11(4):805-16. **NO ACCESS**
152. Huang Y, Mucke L. Alzheimer mechanisms and therapeutic strategies. Cell. 2012 Mar 16;148(6):1204-22. Review.
153. Hughes AJ, Lin RK, Peehl DM, Herr AE. Microfluidic integration for automated targeted proteomic assays. Proc Natl Acad Sci U S A. 2012 Apr 17;109(16):5972-7.
154. Hunter P. Research and practice coming together. The advent of personalized medicine is bringing clinical research and practice closer together. EMBO Rep. 2012 Feb 1;13(2):109-12. doi: 10.1038/embor.2011.258.
155. Hurst DS. Fine-tuning our personalized medicine for otitis media. Otolaryngol Head Neck Surg. 2012 Jun;146(6):1040.
156. Ilie M, Hofman P. Pitfalls in lung cancer molecular pathology: how to limit them in routine practice? Curr Med Chem. 2012;19(16):2638-51. **NO ACCESS**
157. Irimia A, Chambers MC, Torgerson CM, Filippou M, Hovda DA, Alger JR, Gerig G, Toga AW, Vespa PM, Kikinis R, Van Horn JD. Patient-tailored connectomics visualization for the assessment of white matter atrophy in traumatic brain injury. Front Neurol. 2012;3:10.
158. Jain KK. Role of biological therapies in the development of personalized medicine. Expert Opin Biol Ther. 2012 Jan;12(1):1-5. Epub 2011 Nov 22. PubMed PMID: 22107592. **NO ACCESS**
159. Jansen JJ, Szymanska E, Hoefsloot HC, Smilde AK. Individual differences in metabolomics: individualised responses and between-metabolite relationships. Metabolomics. 2012 Jun;8(Suppl 1):94-104.
160. Jeraj R. MO-C-BRCD-01: Towards Personalized Medicine: Integration of Imaging into Therapy. Med Phys. 2012 Jun;39(6):3864. PubMed PMID: 22756862. **NO ACCESS**
161. Jiang X, Osl M, Kim J, Ohno-Machado L. Calibrating predictive model estimates to support personalized medicine. J Am Med Inform Assoc. 2012 Mar-Apr;19(2):263-74.
162. Jin Z, Hildebrandt N. Semiconductor quantum dots for in vitro diagnostics and cellular imaging. Trends Biotechnol. 2012 Jul;30(7):394-403.
163. Jing X, Kay S, Marley T, Hardiker NR, Cimino JJ. Incorporating personalized gene sequence variants, molecular genetics knowledge, and health knowledge into an EHR prototype based on the Continuity of Care Record standard. J Biomed Inform. 2012 Feb;45(1):82-92. **NO ACCESS**
164. Jungić, Tubić B, Skrepnik T. The role of biomarkers in the development of novel cancer therapies. Drug Metabol Drug Interact. 2012 May 30;27(2):89-99. doi: 10.1515/dmdi-2011-0036. **NO ACCESS**
165. Kaiser J. Personalized medicine. New cystic fibrosis drug offers hope, at a price. Science. 2012 Feb 10;335(6069):645. Erratum in: Science. 2012 Feb 24;335(6071):918.
166. Kamal AH, Miriovsky BJ, Currow DC, Abernethy AP. Improving the management of dyspnea in the community using rapid learning approaches. Chron Respir Dis. 2012 Feb;9(1):51-61. doi: 10.1177/1479972311433576. Review.
167. Kandoi G, Nanda A, Scaria V, Sivasubbu S. A case for pharmacogenomics in management of cardiac arrhythmias. Indian Pacing Electrophysiol J. 2012 Mar;12(2):54-64.
168. Kang H, Lee HY, Lee KS, Kim JH. Imaging-based tumor treatment response evaluation: review of conventional, new, and emerging concepts. Korean J Radiol. 2012 Jul;13(4):371-90.
169. Karczewski KJ, Tirrell RP, Cordero P, Tatonetti NP, Dudley JT, Salari K, Snyder M, Altman RB, Kim SK. Interpretome: a freely available, modular, and secure personal genome interpretation engine. Pac Symp Biocomput. 2012:339-50.
170. Karlsson M, Weber W. Therapeutic synthetic gene networks. Curr Opin Biotechnol. 2012 Feb 1.
171. Kaur JS, Petereit DG. Personalized medicine: challenge and promise. J Cancer Educ. 2012 Apr;27 Suppl 1:S12-7. **NO ACCESS**
172. Keeling P, Roth M, Zietlow T. The economics of personalized medicine: commercialization as a driver of return on investment. N Biotechnol. 2012 Jun 17.
173. Keevil SF. Physics and medicine: a historical perspective. Lancet. 2012 Apr 21;379(9825):1517-24.
174. Kelly CM, Pritchard KI. Personalized medicine: what exactly is it and can we truly measure it? J Clin Oncol. 2012 Jun 20;30(18):2173-4.
175. Kennedy MA, Joyce PR, Begg EJ. Institutional Profile: The Carney Centre for Pharmacogenomics: a New Zealand focus for personalized medicine research. Pharmacogenomics. 2012 Jun;13(8):865-8.
176. Kennedy SH, Downar J, Evans KR, Feilotter H, Lam RW, Macqueen GM, Milev R, Parikh SV, Rotzinger S, Soares C. The Canadian Biomarker Integration Network in Depression (CAN-BIND): Advances in Response Prediction. Curr Pharm Des. 2012 Jun 6. **NO ACCESS**
177. Keogh B. Era of personalized medicine may herald end of soaring cancer costs. J Natl Cancer Inst. 2012 Jan 4;104(1):12-3, 16-7.
178. Kerr KM. Personalized medicine for lung cancer: new challenges for pathology. Histopathology. 2012 Mar;60(4):531-46. doi: 10.1111/j.1365-2559.2011.03854.x.
179. Khoury MJ, Coates RJ, Fennell ML, Glasgow RE, Scheuner MT, Schully SD, Williams MS, Clauser SB. Multilevel research and the challenges of implementing genomic medicine. J Natl Cancer Inst Monogr. 2012 May;2012(44):112-20.
180. Kim DC, Wang X, Yang CR, Gao JX. A framework for personalized medicine: prediction of drug sensitivity in cancer by proteomic profiling. Proteome Sci. 2012 Jun 21;10 Suppl 1:S13.
181. Kim KH, Ku B, Kang N, Kim YS, Jang JS, Kim JY. Study of a vocal feature selection method and vocal properties for discriminating four constitution types. Evid Based Complement Alternat Med. 2012;2012:831543.
182. Kirilly E, Gonda X, Bagdy G. CB1 receptor antagonists: new discoveries leading to new perspectives. Acta Physiol (Oxf). 2012 May;205(1):41-60. doi: 10.1111/j.1748-1716.2012.02402.x.
183. Kirk R. Genetics: Personalized medicine and tumour heterogeneity. Nat Rev Clin Oncol. 2012 Mar 20;9(5):250. doi: 10.1038/nrclinonc.2012.46. **NO ACCESS**
184. Kitsios GD, Kent DM. Personalised medicine: not just in our genes. BMJ. 2012 Apr 3;344:e2161. doi: 10.1136/bmj.e2161.
185. Koch K. [Individualized medicine and what journalists can do to build a more realistic picture]. Z Evid Fortbild Qual Gesundhwes. 2012;106(1):23-8. Epub 2012 Jan 2. Review. German.
186. Kogan M, Fischer-Smith T, Kaminsky R, Lehmicke G, Rappaport J. CSF-1R up-regulation is associated with response to pharmacotherapy targeting tyrosine kinase activity in AML cell lines. Anticancer Res. 2012 Mar;32(3):893-9. **NO ACCESS**
187. Kohane IS. (Mis)treating the pharmacogenetic incidentalome. Nat Rev Drug Discov. 2012 Feb 1;11(2):89-90. doi: 10.1038/nrd3659.
188. Kollek R. [Individualisation of medicine: medical philosophy and societal implications of an ambiguous guiding principle]. Z Evid Fortbild Qual Gesundhwes. 2012;106(1):40-5. Epub 2012 Jan 10. German.
189. Krieckaert C, Rispens T, Wolbink G. Immunogenicity of biological therapeutics: from assay to patient. Curr Opin Rheumatol. 2012 May;24(3):306-11.
190. Krupp M, Marquardt JU, Sahin U, Galle PR, Castle J, Teufel A. RNA-Seq Atlas--a reference database for gene expression profiling in normal tissue by next-generation sequencing. Bioinformatics. 2012 Apr 15;28(8):1184-5.
191. Krzesinski JM, Scheen AJ. [Which blood pressure targets in patients with type 2 diabetes?]. Rev Med Liege. 2012 Jan;67(1):51-6. French.
192. Lahti JL, Tang GW, Capriotti E, Liu T, Altman RB. Bioinformatics and variability in drug response: a protein structural perspective. J R Soc Interface. 2012 Jul 7;9(72):1409-37.
193. Lamy PJ, Jacot W. Worldwide variations in EGFR somatic mutations: a challenge for personalized medicine. Diagn Pathol. 2012 Feb 1;7:13.
194. Landau R, Bollag LA, Kraft JC. Pharmacogenetics and anaesthesia: the value of genetic profiling. Anaesthesia. 2012 Feb;67(2):165-79. doi: 10.1111/j.1365-2044.2011.06918.x. Review.
195. Lane HY, Tsai GE, Lin E. Assessing gene-gene interactions in pharmacogenomics. Mol Diagn Ther. 2012 Feb 1;16(1):15-27. doi: 10.2165/11597270-000000000-00000. Review. **NO ACCESS**
196. Langer CJ. Exploring biomarkers in head and neck cancer. Cancer. 2012 Jan 26. doi: 10.1002/cncr.26718.
197. Langman LJ, Snozek CL. The challenges of personalized medicine. Clin Biochem. 2012 Apr;45(6):382-3.
198. Laurinavicius A, Laurinaviciene A, Dasevicius D, Elie N, Plancoulaine B, Bor C, Herlin P. Digital image analysis in pathology: benefits and obligation. Anal Cell Pathol (Amst). 2012;35(2):75-8. Review.
199. Layfield LJ, Bedrossian CW. Bridging the gap: cytopathology at the threshold of personalized medicine. Diagn Cytopathol. 2012 Jun;40(6):469-71. doi: 10.1002/dc.22868.
200. Leask A. Egr-ly awaiting a "personalized medicine" approach to treat scleroderma. J Cell Commun Signal. 2012 Jun;6(2):111-3.
201. Leask A. Toward personalized medicine in scleroderma: classification of scleroderma patients into stable "inflammatory" and "fibrotic" subgroups. J Invest Dermatol. 2012 May;132(5):1329-31. doi: 10.1038/jid.2012.67.
202. Ledet EH, D'Lima D, Westerhoff P, Szivek JA, Wachs RA, Bergmann G. Implantable sensor technology: from research to clinical practice. J Am Acad Orthop Surg. 2012 Jun;20(6):383-92. **NO ACCESS**
203. Lee DE, Koo H, Sun IC, Ryu JH, Kim K, Kwon IC. Multifunctional nanoparticles for multimodal imaging and theragnosis. Chem Soc Rev. 2012 Apr 7;41(7):2656-72. Epub 2011 Dec 21. Review.
204. Leek JT, Peng RD, Anderson RR. Personalized medicine: Keep a way open for tailored treatments. Nature. 2012 Apr 18;484(7394):318. doi: 10.1038/484318a.
205. Lett TA, Wallace TJ, Chowdhury NI, Tiwari AK, Kennedy JL, Müller DJ. Pharmacogenetics of antipsychotic-induced weight gain: review and clinical implications. Mol Psychiatry. 2012 Mar;17(3):242-66. doi: 10.1038/mp.2011.109. Epub 2011 Sep 6. Review.
206. Leung MY, Halpern MT, West ND. Pharmaceutical technology assessment: perspectives from payers. J Manag Care Pharm. 2012 Apr;18(3):256-64.
207. Levran O, Yuferov V, Kreek MJ. The genetics of the opioid system and specific drug addictions. Hum Genet. 2012 Jun;131(6):823-42.
208. Li J, Chen F, Cona MM, Feng Y, Himmelreich U, Oyen R, Verbruggen A, Ni Y. A review on various targeted anticancer therapies. Target Oncol. 2012 Mar;7(1):69-85. **NO ACCESS**
209. Li YY, Jones SJ. Drug repositioning for personalized medicine. Genome Med. 2012 Mar 30;4(3):27. **NO ACCESS**
210. Lin X, Tang W, Ahmad S, Lu J, Colby CC, Zhu J, Yu Q. Applications of targeted gene capture and next-generation sequencing technologies in studies of human deafness and other genetic disabilities. Hear Res. 2012 Jun;288(1-2):67-76.
211. Lindsay S. Biochemistry and semiconductor electronics--the next big hit for silicon? J Phys Condens Matter. 2012 Apr 25;24(16):164201.
212. Lindström S, Schumacher FR, Cox D, Travis RC, Albanes D, Allen NE, Andriole G, Berndt SI, Boeing H, Bueno-de-Mesquita HB, Crawford ED, Diver WR, Gaziano JM, Giles GG, Giovannucci E, Gonzalez CA, Henderson B, Hunter DJ, Johansson M, Kolonel LN, Ma J, Le Marchand L, Pala V, Stampfer M, Stram DO, Thun MJ, Tjonneland A, Trichopoulos D,Virtamo J,Weinstein SJ, Willett WC, Yeager M, Hayes RB, Severi G, Haiman CA, Chanock SJ, Kraft P. Common genetic variants in prostate cancer risk prediction--results from the NCI Breastand Prostate Cancer Cohort Consortium (BPC3). Cancer Epidemiol Biomarkers Prev. 2012 Mar;21(3):437-44. **NO ACCESS**
213. Lippok S, Seidel SA, Duhr S, Uhland K, Holthoff HP, Jenne D, Braun D. Direct detection of antibody concentration and affinity in human serum using microscale thermophoresis. Anal Chem. 2012 Apr 17;84(8):3523-30.
214. Lisanti MP, Tanowitz HB. Translational discoveries, personalized medicine, and living biobanks of the future. Am J Pathol. 2012 Apr;180(4):1334-6.
215. Liu J, Li S, Dunker AK, Uversky VN. Molecular profiling: an essential technology enabling personalized medicine in breast cancer. Curr Drug Targets. 2012 Apr;13(4):541-54. **NO ACCESS**
216. Liu T, Chen Q, Huang Y, Huang Q, Jiang L, Guo L. Low microRNA-199a expression in human amniotic epithelial cell feeder layers maintains human-induced pluripotent stem cell pluripotency via increased leukemia inhibitory factor expression. Acta Biochim Biophys Sin (Shanghai). 2012 Mar;44(3):197-206.
217. Liu T, Cheng W, Huang Y, Huang Q, Jiang L, Guo L. Human amniotic epithelial cell feeder layers maintain human iPS cell pluripotency via inhibited endogenous microRNA-145 and increased Sox2 expression. Exp Cell Res. 2012 Feb 15;318(4):424-34.
218. Liu Y, Welch MJ. Nanoparticles labeled with positron emitting nuclides: advantages, methods, and applications. Bioconjug Chem. 2012 Apr 18;23(4):671-82.
219. Longo DL. Tumor heterogeneity and personalized medicine. N Engl J Med. 2012 Mar 8;366(10):956-7.
220. Loos RJ. Genetic determinants of common obesity and their value in prediction. Best Pract Res Clin Endocrinol Metab. 2012 Apr;26(2):211-26. Review. **NO ACCESS**
221. Louca S, Pochet R, Schinzer D. Why another conference on personalized medicine? Croat Med J. 2012 Jun 15;53(3):205-6.
222. Louca S. Personalized medicine - a tailored health care system: challenges and opportunities. Croat Med J. 2012 Jun 15;53(3):211-3.
223. Lu W, Lu WQ. [Resistance and treatment strategy of gastrointestinal stromal tumor target therapy]. Zhonghua Wei Chang Wai Ke Za Zhi. 2012 Mar;15(3):309-12. Chinese.
224. Ludwig WD. [Possibilities and limitations of stratified medicine based on biomarkers and targeted therapies in oncology]. Z Evid Fortbild Qual Gesundhwes. 2012;106(1):11-22. Epub 2012 Jan 10. Review. German.
225. Luyten FP, Vanlauwe J. Tissue engineering approaches for osteoarthritis. Bone. 2012 Aug;51(2):289-96.
226. Lyon GJ. Personalized medicine: Bring clinical standards to human-genetics research. Nature. 2012 Feb 15;482(7385):300-1. doi: 10.1038/482300a.
227. Mühlhauser I. [Evidence based medicine and individualized medicine]. Z Evid Fortbild Qual Gesundhwes. 2012;106(1):3-4. Epub 2012 Jan 9. German.
228. Ma L, Barker J, Zhou C, Li W, Zhang J, Lin B, Foltz G, Küblbeck J, Honkakoski P. Towards personalized medicine with a three-dimensional micro-scale perfusion-based two-chamber tissue model system. Biomaterials. 2012 Jun;33(17):4353-61.
229. Maeda S. [Genetic study toward personalized medicine for diabetes mellitus]. Nihon Rinsho. 2012 May;70 Suppl 3:579-84. Japanese.
230. Maher ER. Genomics and epigenomics of renal cell carcinoma. Semin Cancer Biol. 2012 Jun 28.
231. Mak CM, Lee CY, Lam CW, Siu WK, Hung VC, Chan AY. Personalized medicine switching from insulin to sulfonylurea in permanent neonatal diabetes mellitus dictated by a novel activating ABCC8 mutation. Diagn Mol Pathol. 2012 Mar;21(1):56-9. **NO ACCESS**
232. Manco M, Dallapiccola B. Genetics of pediatric obesity. Pediatrics. 2012 Jul;130(1):123-33.
233. Manolopoulos VG, Ragia G, Alevizopoulos G. Pharmacokinetic interactions of selective serotonin reuptake inhibitors with other commonly prescribed drugs in the era of pharmacogenomics. Drug Metabol Drug Interact. 2012 Feb 29;27(1):19-31. doi: 10.1515/dmdi-2011-0033. **NO ACCESS**
234. Maojo V, García-Remesal M, Bielza C, Crespo J, Perez-Rey D, Kulikowski C. Biomedical informatics publications: a global perspective: part I: conferences. Methods Inf Med. 2012;51(1):82-90. **NO ACCESS**
235. Mardinoglu A, Nielsen J. Systems medicine and metabolic modelling. J Intern Med. 2012 Feb;271(2):142-54. doi: 10.1111/j.1365-2796.2011.02493.x. Review.
236. Marshall S, Sahm L, McCarthy S. Health literacy in Ireland: reading between the lines. Perspect Public Health. 2012 Jan;132(1):31-8. Review. Erratum in: Perspect Public Health. 2012 Mar;132(2):51. Marshall, Sara [corrected to Marshall, Sarah].
237. Masys DR, Jarvik GP, Abernethy NF, Anderson NR, Papanicolaou GJ, Paltoo DN, Hoffman MA, Kohane IS, Levy HP. Technical desiderata for the integration of genomic data into Electronic Health Records. J Biomed Inform. 2012 Jun;45(3):419-22. **NO ACCESS**
238. Matthews PM, Rabiner EA, Passchier J, Gunn RN. Positron emission tomography molecular imaging for drug development. Br J Clin Pharmacol. 2012 Feb;73(2):175-86. doi: 10.1111/j.1365-2125.2011.04085.x. Review.
239. McMahon FJ, Insel TR. Pharmacogenomics and personalized medicine in neuropsychiatry. Neuron. 2012 Jun 7;74(5):773-6.
240. Melichar B, Plebani M. Editorial: Targeted therapy for HER-2: personalized medicine for her, too. Clin Chem Lab Med. 2012 Jan;50(1):1-4. doi: 10.1515/cclm.2011.724. **NO ACCESS**
241. Mesko B, Zahuczky G, Nagy L. The triad of success in personalised medicine: pharmacogenomics, biotechnology and regulatory issues from a Central European perspective. N Biotechnol. 2012 Mar 10.
242. Mette L, Mitropoulos K, Vozikis A, Patrinos GP. Pharmacogenomics and public health: implementing 'populationalized' medicine. Pharmacogenomics. 2012 May;13(7):803-13.
243. Meyer JH. Neuroimaging markers of cellular function in major depressive disorder: implications for therapeutics, personalized medicine, and prevention. Clin Pharmacol Ther. 2012 Feb;91(2):201-14. doi: 10.1038/clpt.2011.285. **NO ACCESS**
244. Meyer UA. Personalized medicine: a personal view. Clin Pharmacol Ther. 2012 Mar;91(3):373-5. doi: 10.1038/clpt.2011.238. **NO ACCESS**
245. Miki D, Ochi H, Hayes CN, Aikata H, Chayama K. Hepatocellular carcinoma: towards personalized medicine. Cancer Sci. 2012 May;103(5):846-50. doi: 10.1111/j.1349-7006.2012.02242.x.
246. Mitropoulos K, Innocenti F, van Schaik RH, Lezhava A, Tzimas G, Kollia P, Macek M Jr, Fortina P, Patrinos GP. Institutional Profile: Golden Helix Institute of Biomedical Research: interdisciplinary research and educational activities in pharmacogenomics and personalized medicine. Pharmacogenomics. 2012 Mar;13(4):387-92.
247. Moch H, Blank PR, Dietel M, Elmberger G, Kerr KM, Palacios J, Penault-Llorca F, Rossi G, Szucs TD. Personalized cancer medicine and the future of pathology. Virchows Arch. 2012 Jan;460(1):3-8.
248. Molnar JM. [Personalized medicine in psychiatry - an old concept from a new aspect]. Neuropsychopharmacol Hung. 2012 Mar;14(1):2-3. Hungarian.
249. Monsma DJ, Monks NR, Cherba DM, Dylewski D, Eugster E, Jahn H, Srikanth S, Scott SB, Richardson PJ, Everts RE, Ishkin A, Nikolsky Y, Resau JH, Sigler R, Nickoloff BJ, Webb CP. Genomic characterization of explant tumorgraft models derived from fresh patient tumor tissue. J Transl Med. 2012 Jun 18;10(1):125.
250. Montagna RA. Meeting the technical challenges of personalized medicine and companion diagnostics. MLO Med Lab Obs. 2012 Jan;44(1):16, 18, 20 passim. **NO ACCESS**
251. Morabito F, Recchia AG, Mazzone C, Gentile M. Targeted therapy of multiple myeloma: the changing paradigm at the beginning of the new millennium. Curr Cancer Drug Targets. 2012 Jun 5. **NO ACCESS**
252. Moreira AL, Thornton RH. Personalized Medicine for Non-Small-Cell Lung Cancer: Implications of Recent Advances in Tissue Acquisition for Molecular and Histologic Testing. Clin Lung Cancer. 2012 Mar 14. **NO ACCESS**
253. Mouliere F, Thierry AR. The importance of examining the proportion of circulating DNA originating from tumor, microenvironment and normal cells in colorectal cancer patients. Expert Opin Biol Ther. 2012 Jun;12 Suppl 1:S209-15. **NO ACCESS**
254. Mura S, Couvreur P. Nanotheranostics for personalized medicine. Adv Drug Deliv Rev. 2012 Jun 21.
255. Murdoch TB, Detsky AS. Time to Recognize Our Fellow Travellers. J Gen Intern Med. 2012 May 16.
256. Murray JF. Personalized medicine: been there, done that, always needs work! Am J Respir Crit Care Med. 2012 Jun 15;185(12):1251-2.
257. Nagarajan R, Kodell RL. A selective voting convex-hull ensemble procedure for personalized medicine. AMIA Summits Transl Sci Proc. 2012;2012:87-94.
258. Najafzadeh M, Lynd LD, Davis JC, Bryan S, Anis A, Marra M, Marra CA. Barriers to integrating personalized medicine into clinical practice: a best-worst scaling choice experiment. Genet Med. 2012 May;14(5):520-6. doi: 10.1038/gim.2011.26. **NO ACCESS**
259. Nakagawa H. [Research and development for cancer biomarker]. Nihon Rinsho. 2012 May;70(5):743-7. Japanese.
260. Neri PM, Pollard SE, Volk LA, Newmark LP, Varugheese M, Baxter S, Aronson SJ, Rehm HL, Bates DW. Usability of a novel clinician interface for genetic results. J Biomed Inform. 2012 Apr 12. **NO ACCESS**
261. Nierenberg AA. Advancing the treatment of depression with personalized medicine. J Clin Psychiatry. 2012 May;73(5):e17. **NO ACCESS**
262. Niess JH, Klaus J, Stephani J, Pflüger C, Degenkolb N, Spaniol U, Mayer B, Lahr G, von Boyen GB. NOD2 polymorphism predicts response to treatment in Crohn's disease--first steps to a personalized therapy. Dig Dis Sci. 2012 Apr;57(4):879-86.
263. Nishimura T, Kato H, Ikeda N, Kihara M, Nomura M, Kato Y, Marko-Varga G. Cancer Phenotype Diagnosis and Drug Efficacy within Japanese Health Care. Int J Proteomics. 2012;2012:921901.
264. Novak DJ, Liew GJ, Liew CC. GeneNews Limited: bringing the blood transcriptome to personalized medicine. Pharmacogenomics. 2012 Mar;13(4):381-5.
265. Nowak JA. Personalized medicine? Who has the tissue? Arch Pathol Lab Med. 2012 Jun;136(6):590.
266. O'Leary RE, Shih JC, Hyland K, Kramer N, Asher YJ, Graham JM Jr. De novo microdeletion of Xp11.3 exclusively encompassing the monoamine oxidase A and B genes in a male infant with episodic hypotonia: A genomics approach to personalized medicine. Eur J Med Genet. 2012 May;55(5):349-53. **NO ACCESS**
267. Ohshiro T, Matsubara K, Tsutsui M, Furuhashi M, Taniguchi M, Kawai T. Single-molecule electrical random resequencing of DNA and RNA. Sci Rep. 2012;2:501.
268. Oliveras-Ferraros C, Massaguer Vall-Llovera A, Vazquez-Martin A, Salip DC, Queralt B, Cufí S, Martin-Castillo B, Bosch-Barrera J, Brunet J, De Llorens R, Menendez JA. Transcriptional upregulation of HER2 expression in the absence of HER2 gene amplification results in cetuximab resistance that is reversed by trastuzumab treatment. Oncol Rep. 2012 Jun;27(6):1887-92. doi: 10.3892/or.2012.1732. **NO ACCESS**
269. Oronsky BT, Scicinski JJ, Reid T, Knox S. Beyond antiangiogenesis: vascular modulation as an anticancer therapy-a review. Transl Oncol. 2012 Jun;5(3):133-40. Epub 2012 Jun 1.
270. Overby CL, Devine EB, Tarczy-Hornoch P, Kalet IJ. Deriving rules and assertions from pharmacogenomics knowledge resources in support of patient drug metabolism efficacy predictions. J Am Med Inform Assoc. 2012 Apr 26. **NO ACCESS**
271. Owen DR, Rupprecht R, Nutt DJ. Stratified medicine in psychiatry: a worrying example or new opportunity in the treatment of anxiety? J Psychopharmacol. 2012 Apr 20.
272. Papakostas GI. Surrogate markers of treatment outcome in major depressive disorder. Int J Neuropsychopharmacol. 2012 Jul;15(6):841-54.
273. Patowary A, Purkanti R, Singh M, Chauhan RK, Bhartiya D, Dwivedi OP, Chauhan G, Bharadwaj D, Sivasubbu S, Scaria V. Systematic analysis and functional annotation of variations in the genome of an Indian individual. Hum Mutat. 2012 Jul;33(7):1133-40. doi: 10.1002/humu.22091.
274. Pellegrini I, Rapti M, Extra JM, Petri-Cal A, Apostolidis T, Ferrero JM, Bachelot T, Viens P, Bertucci F, Julian-Reynier C. [Targeted chemotherapy for breast cancer: patients perception of the use of tumor gene profiling approaches to better adapt treatments]. Med Sci (Paris). 2012 Mar;28 Spec No 1:24-7. Review. French.
275. Pellegrini I, Rapti M, Extra JM, Petri-Cal A, Apostolidis T, Ferrero JM, Bachelot T, Viens P, Julian-Reynier C, Bertucci F. Tailored chemotherapy based on tumour gene expression analysis: breast cancer patients' misinterpretations and positive attitudes. Eur J Cancer Care (Engl). 2012 Mar;21(2):242-50. doi: 10.1111/j.1365-2354.2011.01300.x.
276. Peng CC, Chen KC, Lu HY, Peng RY. Treadmill exercise improved adriamycin-induced nephropathy. J Biol Regul Homeost Agents. 2012 Jan-Mar;26(1):15-28. **NO ACCESS**
277. Peng J. Meeting Report: EMBL Conference - Omics and Personalized Medicine: February 16-18, 2012, Heidelberg, Germany. Biotechnol J. 2012 Mar 15. doi: 10.1002/biot.201200122.
278. Peretz Y, Cameron C, Sékaly RP. Dissecting the HIV-specific immune response: a systems biology approach. Curr Opin HIV AIDS. 2012 Jan;7(1):17-23. Review. **NO ACCESS**
279. Perumal J, Khan O. Emerging disease-modifying therapies in multiple sclerosis. Curr Treat Options Neurol. 2012 Jun;14(3):256-63. **NO ACCESS**
280. Peterson TA, Nehrt NL, Park D, Kann MG. Incorporating molecular and functional context into the analysis and prioritization of human variants associated with cancer. J Am Med Inform Assoc. 2012 Mar-Apr;19(2):275-83. **NO ACCESS**
281. Pierobon M, Vanmeter AJ, Moroni N, Galdi F, Petricoin EF 3rd. Reverse-phase protein microarrays. Methods Mol Biol. 2012;823:215-35. **NO ACCESS**
282. Pino-Ángeles A, Reyes-Palomares A, Melgarejo E, Sánchez-Jiménez F. Histamine: an undercover agent in multiple rare diseases? J Cell Mol Med. 2012 Mar 21. doi: 10.1111/j.1582-4934.2012.01566.x. **NO ACCESS**
283. Poon AH, Hamid Q. Personalized medicine for asthma: Are we there yet? Ann Thorac Med. 2012 Apr;7(2):55-6.
284. Portelli M, Sayers I. Genetic basis for personalized medicine in asthma. Expert Rev Respir Med. 2012 Apr;6(2):223-36.
285. Prasongchean W, Ferretti P. Autologous stem cells for personalised medicine. N Biotechnol. 2012 Apr 27.
286. Printz C. Universities bring personalized medicine to the forefront of patient care: two programs aim to make customized treatments easily accessible Cancer. 2012 May 15;118(10):2563-4. doi: 10.1002/cncr.27600.
287. Puhalla S, Bhattacharya S, Davidson NE. Hormonal therapy in breast cancer: a model disease for the personalization of cancer care. Mol Oncol. 2012 Apr;6(2):222-36. **NO ACCESS**
288. Pulley JM, Denny JC, Peterson JF, Bernard GR, Vnencak-Jones CL, Ramirez AH, Delaney JT, Bowton E, Brothers K, Johnson K, Crawford DC, Schildcrout J, Masys DR, Dilks HH, Wilke RA, Clayton EW, Shultz E, Laposata M, McPherson J, Jirjis JN, Roden DM. Operational Implementation of Prospective Genotyping for Personalized Medicine: The Design of the Vanderbilt PREDICT Project. Clin Pharmacol Ther. 2012 Jul;92(1):87-95. doi: 10.1038/clpt.2011.371.
289. Puri R, Duong M, Uno K, Kataoka Y, Nicholls SJ. The emerging role of plasma lipidomics in cardiovascular drug discovery. Expert Opin Drug Discov. 2012 Jan;7(1):63-72. Epub 2011 Dec 12. **NO ACCESS**
290. Pyeritz RE. The family history: the first genetic test, and still useful after all those years? Genet Med. 2012 Jan;14(1):3-9. doi: 10.1038/gim.0b013e3182310bcf. Epub 2011 Oct 7. Review. **NO ACCESS**
291. Qu HQ, Li Q, Xu S, McCormick JB, Fisher-Hoch SP, Xiong M, Qian J, Jin L. Ancestry informative marker set for han chinese population. G3 (Bethesda). 2012 Mar;2(3):339-41.
292. Raspe E, Decraene C, Berx G. Gene expression profiling to dissect the complexity of cancer biology: pitfalls and promise. Semin Cancer Biol. 2012 Jun;22(3):250-60.
293. Rathore SS, Agarwal SK, Pande S, Singh SK, Mittal T, Mittal B. Therapeutic dosing of acenocoumarol: proposal of a population specific pharmacogenetic dosing algorithm and its validation in north Indians. PLoS One. 2012;7(5):e37844.
294. Ray P, De A. Reporter gene imaging in therapy and diagnosis. Theranostics. 2012;2(4):333-4.
295. Redis RS, Berindan-Neagoe I, Pop VI, Calin GA. Non-coding RNAs as theranostics in human cancers. J Cell Biochem. 2012 May;113(5):1451-9. doi: 10.1002/jcb.24038.
296. Richardson AL, Iglehart JD. BEAMing Up Personalized Medicine: Mutation Detection in Blood. Clin Cancer Res. 2012 Jun 15;18(12):3209-11.
297. Richmond ES, Dunn D. Biomarkers: an overview for oncology nurses. Semin Oncol Nurs. 2012 May;28(2):87-92. **NO ACCESS**
298. Rigby M. Personal Health, Person-centred Health and Personalised Medicine - Concepts, Consumers, Confusion and Challenges in the Informatics World. Yearb Med Inform. 2012;7(1):7-15. **NO ACCESS**
299. Rinaldi F, Schneider G, Clematide S. Relation mining experiments in the pharmacogenomics domain. J Biomed Inform. 2012 May 10. **NO ACCESS**
300. Ritter C, Sehrt D, Jaehde U. Editorial. Approaches to personalized medicine in oncology--the 9th Annual Meeting of CESAR in Greifswald. Int J Clin Pharmacol Ther. 2012 Jan;50(1):53-4. **NO ACCESS**
301. Roberts R, Stewart AF. 9p21 and the genetic revolution for coronary artery disease. Clin Chem. 2012 Jan;58(1):104-12.
302. Roda A, Guardigli M. Analytical chemiluminescence and bioluminescence: latest achievements and new horizons. Anal Bioanal Chem. 2012 Jan;402(1):69-76. Epub 2011 Oct 16. Review.
303. Rosbach KJ, Williams MD, Gillenwater AM, Richards-Kortum RR. Optical molecular imaging of multiple biomarkers of epithelial neoplasia: epidermal growth factor receptor expression and metabolic activity in oral mucosa. Transl Oncol. 2012 Jun;5(3):160-71.
304. Rosen Y, Upadhyay UM, Elman NM. Pharmacogenomics-based RNA interference nanodelivery: focus on solid malignant tumors. Expert Opin Drug Deliv. 2012 Jul;9(7):755-66. **NO ACCESS**
305. Ruben RJ. Response to "fine-tuning our personalized medicine for otitis media" by david s. Hurst. Otolaryngol Head Neck Surg. 2012 Jun;146(6):1040-1.
306. Rudick RA. The elusive biomarker for personalized medicine in multiple sclerosis: The search continues. Neurology. 2012 May 9.
307. Ruiz C, Tolnay M, Bubendorf L. Application of personalized medicine to solid tumors: opportunities and challenges. Swiss Med Wkly. 2012 Jun 19;142:0. doi: 10.4414/smw.2012.13587.
308. Ruppen-Cañás I, López-Casas PP, García F, Ximénez-Embún P, Muñoz M, Morelli MP, Real FX, Serna A, Hidalgo M, Ashman K. An improved quantitative mass spectrometry analysis of tumor specific mutant proteins at high sensitivity. Proteomics. 2012 May;12(9):1319-27. doi: 10.1002/pmic.201100611.
309. Sahay A, Brown M, Muzzio F, Takhistov P. Automated Drop-on-Demand System with Real-Time Gravimetric Control for Precise Dosage Formulation. J Lab Autom. 2012 Apr 24. **NO ACCESS**
310. Salari K, Watkins H, Ashley EA. Personalized medicine: hope or hype? Eur Heart J. 2012 Jul;33(13):1564-70.
311. Salvador-Carulla L, Mezzich JE. Person-centred medicine and mental health. Epidemiol Psychiatr Sci. 2012 Jun;21(2):131-7.
312. Samwald M, Coulet A, Huerga I, Powers RL, Luciano JS, Freimuth RR, Whipple F, Pichler E, Prud'hommeaux E, Dumontier M, Marshall MS. Semantically enabling pharmacogenomic data for the realization of personalized medicine. Pharmacogenomics. 2012 Jan;13(2):201-12. Review.
313. Sandmann T, Boutros M. Screens, maps & networks: from genome sequences to personalized medicine. Curr Opin Genet Dev. 2012 Feb;22(1):36-44
314. Sant S, Tao SL, Fisher OZ, Xu Q, Peppas NA, Khademhosseini A. Microfabrication technologies for oral drug delivery. Adv Drug Deliv Rev. 2012 May 1;64(6):496-507.
315. Savenije OE, Kerkhof M, Koppelman GH, Postma DS. Predicting who will have asthma at school age among preschool children. J Allergy Clin Immunol. 2012 Jun 14.
316. Scalvini A, Ferrari V, Bodei S, Arcangeli G, Consoli F, Spano P, Sigala S. Involvement of target gene polymorphisms in 5-Fluorouracil toxicity: a case report. Pharmacology. 2012;89(1-2):99-102.
317. Scheen AJ. Dipeptidylpeptidase-4 (DPP-4) inhibitors are favourable to glucagon-like peptide-1 (GLP-1) receptor agonists: yes. Eur J Intern Med. 2012 Mar;23(2):126-31. Epub 2011 Nov 15. Review. **NO ACCESS**
318. Scheer N, Kapelyukh Y, McEwan J, Beuger V, Stanley LA, Rode A, Wolf CR. Modeling human cytochrome P450 2D6 metabolism and drug-drug interaction by a novel panel of knockout and humanized mouse lines. Mol Pharmacol. 2012 Jan;81(1):63-72.
319. Schiavone MB, Bashir S, Herzog TJ. Biologic therapies and personalized medicine in gynecologic malignancies. Obstet Gynecol Clin North Am. 2012 Jun;39(2):131-44. **NO ACCESS**
320. Schumacher S, Nestler J, Otto T, Wegener M, Ehrentreich-Förster E, Michel D,Wunderlich K, Palzer S, Sohn K, Weber A, Burgard M, Grzesiak A, Teichert A, Brandenburg A, Koger B, Albers J, Nebling E, Bier FF. Highly-integrated lab-on-chip system for point-of-care multiparameter analysis. Lab Chip. 2012 Feb 7;12(3):464-73.
321. Scudellari M. Genomics contest underscores challenges of personalized medicine. Nat Med. 2012 Mar 6;18(3):326. doi: 10.1038/nm0312-326.
322. Shah RR, Shah DR. Personalised Medicine: Is it a pharmacogenetic mirage? Br J Clin Pharmacol. 2012 May 16. doi: 10.1111/j.1365-2125.2012.04328.x.
323. Shao L, Fan X, Cheng N, Wu L, Xiong H, Fang H, Ding D, Shi L, Cheng Y, Tong W. Shifting from population-wide to personalized cancer prognosis with microarrays. PLoS One. 2012;7(1):e29534.
324. Sharrer GT. Personalized medicine: ethics for clinical trials. Methods Mol Biol. 2012;823:35-48. **NO ACCESS**
325. Shen L, Lu ZH. [New perspectives on medical treatment of gastric cancer]. Zhonghua Wei Chang Wai Ke Za Zhi. 2012 Feb;15(2):103-8. Chinese.
326. Shigemizu D, Hu Z, Hung JH, Huang CL, Wang Y, DeLisi C. Using functional signatures to identify repositioned drugs for breast, myelogenous leukemia and prostate cancer. PLoS Comput Biol. 2012 Feb;8(2):e1002347.
327. Shim MS, Kwon YJ. Stimuli-responsive polymers and nanomaterials for gene delivery and imaging applications. Adv Drug Deliv Rev. 2012 Feb 4.
328. Shima K, Mizuma M, Hayashi H, Nakagawa K, Okada T, Sakata N, Omura N, Kitamura Y, Motoi F, Rikiyama T, Katayose Y, Egawa S, Ishii N, Horii A, Unno M. Potential utility of eGFP-expressing NOG mice (NOG-EGFP) as a high purity cancer sampling system. J Exp Clin Cancer Res. 2012 Jun 6;31(1):55.
329. Shoemaker LD, Achrol AS, Sethu P, Steinberg GK, Chang SD. Clinical neuroproteomics and biomarkers: from basic research to clinical decision making. Neurosurgery. 2012 Mar;70(3):518-25.
330. Shublaq NW, Coveney PV. Merging genomic and phenomic data for research and clinical impact. Stud Health Technol Inform. 2012;174:111-5. **NO ACCESS**
331. Shute N. Personalized medicine. Sci Am. 2012 May;306(5):44. **NO ACCESS**
332. Silindir M, Erdoğan S, Özer AY, Maia S. Liposomes and their applications in molecular imaging. J Drug Target. 2012 Jun;20(5):401-15.
333. Somberg J. Is there an imperative for generic profiling to hasten the age of personalized medicine. Am J Ther. 2012 May;19(3):163. **NO ACCESS**
334. Sorich MJ, McKinnon RA. Personalized medicine: Potential, barriers and contemporary issues. Curr Drug Metab. 2012 May 16. **NO ACCESS**
335. Spiegel AM, Hawkins M. 'Personalized medicine' to identify genetic risks for type 2 diabetes and focus prevention: can it fulfill its promise? Health Aff (Millwood). 2012 Jan;31(1):43-9. **NO ACCESS**
336. Squassina A, Severino G, Grech G, Fenech A, Borg J, Patrinos GP. Golden Helix Pharmacogenomics Days: educational activities on pharmacogenomics and personalized medicine. Pharmacogenomics. 2012 Apr;13(5):525-8.
337. Srikanth Babu BM, Pulla Reddy B, Priya VH, Munshi A, Surekha Rani H, Suman Latha G, Rao VD, Jyothy A. Cytokine gene polymorphisms in the susceptibility to acute coronary syndrome. Genet Test Mol Biomarkers. 2012 May;16(5):359-65. **NO ACCESS**
338. Srivastava SC. Paving the way to personalized medicine: production of some promising theragnostic radionuclides at Brookhaven National Laboratory. Semin Nucl Med. 2012 May;42(3):151-63.
339. Stankov K, Draskovic D, Mikov M. Ethical and legal aspects of oncogenomics. J BUON. 2012 Apr-Jun;17(2):383-8. **NO ACCESS**
340. Stegle O, Roth FP, Morris Q, Listgarten J. Personalized medicine: from genotypes and molecular phenotypes towards computed therapy. Pac Symp Biocomput. 2012:323-6. **NO ACCESS**
341. Stingl JC, Brockmöller J, Viviani R. Genetic variability of drug-metabolizing enzymes: the dual impact on psychiatric therapy and regulation of brain function. Mol Psychiatry. 2012 May 8. doi: 10.1038/mp.2012.42.
342. Stranger BE, Björkegren J, Dolan ME, Ritchie MD. Systems and genome-wide approaches unite to provide a route to personalized medicine. Genome Med. 2012 Mar 30;4(3):29. **NO ACCESS**
343. Stratz C, Amann M, Berg DD, Morrow DA, Neumann FJ, Hochholzer W. Novel biomarkers in cardiovascular disease: research tools or ready for personalized medicine? Cardiol Rev. 2012 May;20(3):111-7. Review. **NO ACCESS**
344. Streffer JR, Grachev ID, Fitzer-Attas C, Gomez-Mancilla B, Boroojerdi B, Bronzova J, Ostrowitzki S, Victor SJ, Fontoura P, Alexander R. Prerequisites to launch neuroprotective trials in Parkinson's disease: an industry perspective. Mov Disord. 2012 Apr 15;27(5):651-5. doi: 10.1002/mds.25017.
345. Strumberg D. Sorafenib for the treatment of renal cancer. Expert Opin Pharmacother. 2012 Feb;13(3):407-19. **NO ACCESS**
346. Stucker F, Marti HP, Hunger RE. Immunosuppressive drugs in organ transplant recipients--rationale for critical selection. Curr Probl Dermatol. 2012;43:36-48. Epub 2012 Feb 17. Review. **NO ACCESS**
347. Sung J, Wang Y, Chandrasekaran S, Witten DM, Price ND. Molecular signatures from omics data: From chaos to consensus. Biotechnol J. 2012 Apr 23. doi: 10.1002/biot.201100305. **NO ACCESS**
348. Szefler SJ. Advances in pediatric asthma in 2011: moving forward. J Allergy Clin Immunol. 2012 Jan;129(1):60-8. Review.
349. Tak PP. A personalized medicine approach to biologic treatment of rheumatoid arthritis: a preliminary treatment algorithm. Rheumatology (Oxford). 2012 Apr;51(4):600-9.
350. Takano EA, Rogers TM, Young RJ, Rayoo M, Kostos P, Ferguson R, Campbell IG, Debiec-Rychter M, Fox SB. The molecular characterisation of unusual subcutaneous spindle cell lesion of breast. J Clin Pathol. 2012 Aug;65(8):746-50.
351. Tan HT, Lee YH, Chung MC. Cancer proteomics. Mass Spectrom Rev. 2012 Mar 15. doi: 10.1002/mas.20356.
352. Terreno E, Uggeri F, Aime S. Image guided therapy: The advent of theranostic agents. J Control Release. 2012 Jul 20;161(2):328-37.
353. Teufel A, Marquardt JU, Dooley S, Lammert F, Galle PR. [Personalised hepatology - current concepts, developments and expectations in the post-genome era]. Z Gastroenterol. 2012 Jan;50(1):41-6. Review. German.
354. Thariani R, Veenstra DL, Carlson JJ, Garrison LP, Ramsey S. Paying for personalized care: cancer biomarkers and comparative effectiveness. Mol Oncol. 2012 Apr;6(2):260-6. **NO ACCESS**
355. Thompson CA. Industry panel discusses status of personalized medicine. Am J Health Syst Pharm. 2012 Mar 1;69(5):366. doi: 10.2146/news120017. **NO ACCESS**
356. Thunnissen E, Kerr KM, Herth FJ, Lantuejoul S, Papotti M, Rintoul RC, Rossi G, Skov BG, Weynand B, Bubendorf L, Katrien G, Johansson L, López-Ríos F, Ninane V, Olszewski W, Popper H, Jaume S, Schnabel P, Thiberville L, Laenger F. The challenge of NSCLC diagnosis and predictive analysis on small samples. Practical approach of a working group. Lung Cancer. 2012 Apr;76(1):1-18.
357. Togarrati PP, Suknuntha K. Generation of mature hematopoietic cells from human pluripotent stem cells. Int J Hematol. 2012 Jun;95(6):617-23.
358. Toh HC, Ha TC, Wee J. Personalised medicine in nasopharyngeal cancer. Lancet Oncol. 2012 Jun;13(6):568-9. Epub 2012 May 3. Erratum in: Lancet Oncol. 2012 Jun;13(6):e231.
359. Toledo RA, Sekiya T, Longuini VC, Coutinho FL, Lourenço DM Jr, Toledo SP. Narrowing the gap of personalized medicine in emerging countries: the case of multiple endocrine neoplasias in Brazil. Clinics (Sao Paulo). 2012;67 Suppl 1:3-6.
360. Tomczak N, Jańczewski D, Dorokhin D, Han MY, Vancso GJ. Enabling biomedical research with designer quantum dots. Methods Mol Biol. 2012;811:245-65. Review. **NO ACCESS**
361. Travers J, Weatherall M, Fingleton J, Beasley R. Towards individualised medicine for airways disease: identifying clinical phenotype groups. Eur Respir J. 2012 Apr;39(4):1033-4.
362. Trovato GM. Behavior, nutrition and lifestyle in a comprehensive health and disease paradigm: skills and knowledge for a predictive, preventive and personalized medicine. EPMA J. 2012 Mar 22;3(1):8.
363. Tukacs E, Korotij A, Maros-Szabo Z, Molnar AM, Hajdu A, Torok Z. Model requirements for Biobank Software Systems. Bioinformation. 2012;8(6):290-2.
364. Vaidyanathan G. Redefining clinical trials: the age of personalized medicine. Cell. 2012 Mar 16;148(6):1079-80.
365. Velikyan I, Xu H, Nair M, Hall H. Robust labeling and comparative preclinical characterization of DOTA-TOC and DOTA-TATE. Nucl Med Biol. 2012 Jul;39(5):628-39.
366. Verweij J, de Jonge M, Eskens F, Sleijfer S. Moving molecular targeted drug therapy towards personalized medicine: issues related to clinical trial design. Mol Oncol. 2012 Apr;6(2):196-203. **NO ACCESS**
367. Vig HS, Wang C. The evolution of personalized cancer genetic counseling in the era of personalized medicine. Fam Cancer. 2012 Mar 15.
368. Villoslada P, Baranzini S. Data integration and systems biology approaches for biomarker discovery: Challenges and opportunities for multiple sclerosis. J Neuroimmunol. 2012 Jul 15;248(1-2):58-65.
369. Vilos C, Velasquez LA. Therapeutic strategies based on polymeric microparticles. J Biomed Biotechnol. 2012;2012:672760.
370. Viollet B, Guigas B, Sanz Garcia N, Leclerc J, Foretz M, Andreelli F. Cellular and molecular mechanisms of metformin: an overview. Clin Sci (Lond). 2012 Mar;122(6):253-70. Review.
371. Vittos O, Toana B, Vittos A, Moldoveanu E. Lipoprotein-associated phospholipase A2 (Lp-PLA2): a review of its role and significance as a cardiovascular biomarker. Biomarkers. 2012 Jun;17(4):289-302.
372. Voit EO, Qi Z, Kikuchi S. Mesoscopic models of neurotransmission as intermediates between disease simulators and tools for discovering design principles. Pharmacopsychiatry. 2012 May;45 Suppl 1:S22-30. **NO ACCESS**
373. Wagle Shukla A, Okun MS. Personalized medicine in deep brain stimulation through utilization of neural oscillations. Neurology. 2012 Jun 12;78(24):1900-1.
374. Wald NJ, Morris JK. Personalized medicine: hope or hype. Eur Heart J. 2012 Jul;33(13):1553-4.
375. Walsh J, Arora M, Hosenfeld C, Ladabaum U, Kuppermann M, Knight SJ. Preferences for genetic testing to identify hereditary colorectal cancer: perspectives of high-risk patients, community members, and clinicians. J Cancer Educ. 2012 Mar;27(1):112-9. **NO ACCESS**
376. Wan TS, Ma ES. Molecular cytogenetics: an indispensable tool for cancer diagnosis. Chang Gung Med J. 2012 Mar-Apr;35(2):96-110.
377. Wang J, Pang GS, Chong SS, Lee CG. SNP Web Resources and Their Potential Applications in Personalized Medicine. Curr Drug Metab. 2012 May 16. **NO ACCESS**
378. Wang Q. Individualized medicine, health medicine, and constitutional theory in Chinese medicine. Front Med. 2012 Mar;6(1):1-7.
379. Wang Y, Schmid-Bindert G, Zhou C. Erlotinib in the treatment of advanced non-small cell lung cancer: an update for clinicians. Ther Adv Med Oncol. 2012 Jan;4(1):19-29.
380. Ware JS, Roberts AM, Cook SA. Republished review: Next generation sequencing for clinical diagnostics and personalised medicine: implications for the next generation cardiologist. Postgrad Med J. 2012 Apr;88(1038):234-9.
381. Wei DQ, Chen Q. Editorial: SNPs of Drug Metabolic Enzymes and Personalized Medicine Part II. Curr Drug Metab. 2012 May 16. **NO ACCESS**
382. Weir NA, Levine SJ. Achieving symptom control in patients with moderate asthma. Clin Med Insights Circ Respir Pulm Med. 2012;6:1-11.
383. Weiss ST. New approaches to personalized medicine for asthma: where are we? J Allergy Clin Immunol. 2012 Feb;129(2):327-34. Review.
384. Weller M, Stupp R, Hegi M, Wick W. Individualized targeted therapy for glioblastoma: fact or fiction? Cancer J. 2012 Jan-Feb;18(1):40-4. Review. **NO ACCESS**
385. Wells QS, Delaney JT, Roden DM. Genetic determinants of response to cardiovascular drugs. Curr Opin Cardiol. 2012 May;27(3):253-61. **NO ACCESS**
386. Weyers A, Yang B, Yoon DS, Park JH, Zhang F, Lee KB, Linhardt RJ. A structural analysis of glycosaminoglycans from lethal and nonlethal breast cancer tissues: toward a novel class of theragnostics for personalized medicine in oncology? OMICS. 2012 Mar;16(3):79-89. **NO ACCESS**
387. Wheeler HE, Dolan ME. Lymphoblastoid cell lines in pharmacogenomic discovery and clinical translation. Pharmacogenomics. 2012 Jan;13(1):55-70. Review.
388. Whitcomb DC. What is personalized medicine and what should it replace? Nat Rev Gastroenterol Hepatol. 2012 May 22;9(7):418-24. doi: 10.1038/nrgastro.2012.100. **NO ACCESS**
389. Wieland M, Fussenegger M. Reprogrammed cell delivery for personalized medicine. Adv Drug Deliv Rev. 2012 Jun 19.
390. Wild DJ, Ding Y, Sheth AP, Harland L, Gifford EM, Lajiness MS. Systems chemical biology and the Semantic Web: what they mean for the future of drug discovery research. Drug Discov Today. 2012 May;17(9-10):469-74. **NO ACCESS**
391. Willer CJ, Mohlke KL. Finding genes and variants for lipid levels after genome-wide association analysis. Curr Opin Lipidol. 2012 Apr;23(2):98-103. **NO ACCESS**
392. Wilson MA, Nathanson KL. Molecular testing in melanoma. Cancer J. 2012 Mar-Apr;18(2):117-23.
393. Windeler J. [Individualized medicine - our (lack of) understanding]. Z Evid Fortbild Qual Gesundhwes. 2012;106(1):5-10. Epub 2011 Sep 22. German.
394. Wong H, Yau T. Targeted therapy in the management of advanced gastric cancer: are we making progress in the era of personalized medicine? Oncologist. 2012;17(3):346-58. **NO ACCESS**
395. Workman P, Clarke PA, Al-Lazikani B. Personalized medicine: patient-predictive panel power. Cancer Cell. 2012 Apr 17;21(4):455-8. doi: 10.1016/j.ccr.2012.03.030.
396. Wujcik DM. What do our patients think personalized medicine means? The answer may surprise you. ONS Connect. 2012 Feb;27(2):7. **NO ACCESS**
397. Yamaguchi R, Perkins G. Finding a Panacea among combination cancer therapies. Cancer Res. 2012 Jan 1;72(1):18-23.
398. Yancy CW, Lee DC. Personalized medicine in heart failure: are we there yet? JACC Cardiovasc Imaging. 2012 Apr;5(4):419-21. **NO ACCESS**
399. Yang L, Valdes R Jr, Taubert D, Linder MW. A diagnostic informatics approach for stratifying risk outcome based on combined genotype effects. Ther Drug Monit. 2012 Jun;34(3):283-8. **NO ACCESS**
400. Yang X, Zhang B, Zhu J. Functional Genomics- and Network-driven Systems Biology Approaches for Pharmacogenomics and Toxicogenomics. Curr Drug Metab. 2012 May 16. **NO ACCESS**
401. Yeang CH, Ma GC, Shih JC, Yang YS, Chen CP, Chang SP, Wu SH, Liu CS, Kuo SJ, Chou HC, Hwu WL, Cameron AD, Ginsberg NA, Lin YS, Chen M. Genome-wide gene expression analysis implicates the immune response and lymphangiogenesis in the pathogenesis of fetal chylothorax. PLoS One. 2012;7(4):e34901.
402. Yim SW, Kim T, Laurence TA, Partono S, Kim D, Kim Y, Weiss S, Reitmair A. Four-color alternating-laser excitation single-molecule fluorescence spectroscopy for next-generation biodetection assays. Clin Chem. 2012 Apr;58(4):707-16.
403. Yokota T, Duddy W, Echigoya Y, Kolski H. Exon skipping for nonsense mutations in Duchenne muscular dystrophy: too many mutations, too few patients? Expert Opin Biol Ther. 2012 Jun 1. **NO ACCESS**
404. Yoshikawa K, Shimada M, Kurita N. Personalized medicine for laparoscopic gastrectomy used to treat gastric cancer. Surg Endosc. 2012 Apr 27.
405. Yu JX, Kodibagkar VD, Hallac RR, Liu L, Mason RP. Dual 19F/1H MR gene reporter molecules for in vivo detection of β-galactosidase. Bioconjug Chem. 2012 Mar 21;23(3):596-603.
406. Zappa A, Splendiani A, Romano P. Towards linked open gene mutations data.BMC Bioinformatics. 2012 Mar 28;13 Suppl 4:S7.
407. Zarco MF, Vess TJ, Ginsburg GS. The oral microbiome in health and disease and the potential impact on personalized dental medicine. Oral Dis. 2012 Mar;18(2):109-20. doi: 10.1111/j.1601-0825.2011.01851.x.
408. Zhang A, Sun H, Wang P, Han Y, Wang X. Future perspectives of personalized medicine in traditional Chinese medicine: a systems biology approach. Complement Ther Med. 2012 Feb-Apr;20(1-2):93-9.
409. Zhang T, Zhao M, Pang Y, Zhang W, Angela Liu L, Wei DQ. Recent progress on bioinformatics, functional genomics, and metabolomics research of cytochrome p450 and its impact on drug discovery. Curr Top Med Chem. 2012 Jun 1;12(12):1346-55. **NO ACCESS**
410. Zhang W, Zhang WJ, Zhu J, Kong FC, Li YY, Wang HY, Yang YH, Wang C. Genetic polymorphisms are associated with variations in warfarin maintenance dose in Han Chinese patients with venous thromboembolism. Pharmacogenomics. 2012 Feb;13(3):309-21.
411. Zhou L, Beuerman RW. Tear analysis in ocular surface diseases. Prog Retin Eye Res. 2012 Jun 23.
412. Zieba A, Grannas K, Söderberg O, Gullberg M, Nilsson M, Landegren U. Molecular tools for companion diagnostics. N Biotechnol. 2012 May 24.
413. Zimmer L, Vaubel J, Livingstone E, Schadendorf D. Side effects of systemic oncological therapies in dermatology. J Dtsch Dermatol Ges. 2012 Jul;10(7):475-86. doi: 10.1111/j.1610-0387.2012.07942.x.
414. Zuckerman R, Milne CP. Market watch: industry perspectives on personalized medicine. Nat Rev Drug Discov. 2012 Mar 1;11(3):178. doi: 10.1038/nrd3677.

**No author stated:**

1. [Mental health care for young people: early intervention in psychosis]. Seishin Shinkeigaku Zasshi. 2012;114(3):303-9. Japanese.
2. Personalized medicine comes to cystic fibrosis. Am J Med Genet A. 2012 Feb;158A(2):viii-ix. doi: 10.1002/ajmg.a.35226.
3. Succinct Healthcare Communications. Companion diagnostics: changing patient management. Ecancermedicalscience. 2012;6:244.
4. What happened to personalized medicine? Nat Biotechnol. 2012 Jan 9;30(1):1. doi: 10.1038/nbt.2096.

**2011**

1. Abadi-Korek I, Shemer J. [Personalized medicine--the future is already here]. Harefuah. 2011 May;150(5):451-2, 490. Hebrew.
2. Abernethy AP, Basch E. Clinical cancer informatics: creating pathways for personalized medicine and rapid learning cancer care from the guest editors. Cancer J. 2011 Jul-Aug;17(4):195-6. **NO ACCESS**
3. Abrahams JP, Apweiler R, Balling R, Bertero MG, Bujnicki JM, Chayen NE, Chène P, Corthals GL, Dylag T, Förster F, Heck AJ, Henderson PJ, Herwig R, Jehenson P, Kokalj SJ, Laue E, Legrain P, Martens L, Migliorini C, Musacchio A, Podobnik M, Schertler GF, Schreiber G, Sixma TK, Smit AB, Stuart D, Svergun DI, Taussig MJ. "4D Biology for health and disease" workshop report. N Biotechnol. 2011 Jul;28(4):291-3.
4. Ahmed J, Meinel T, Dunkel M, Murgueitio MS, Adams R, Blasse C, Eckert A, Preissner S, Preissner R. CancerResource: a comprehensive database of cancer-relevant proteins and compound interactions supported by experimental knowledge. Nucleic Acids Res. 2011 Jan;39(Database issue):D960-7. doi: 10.1093/nar/gkq910.
5. Alduaij W, Illidge TM. The future of anti-CD20 monoclonal antibodies: are we making progress? Blood. 2011 Mar 17;117(11):2993-3001. **NO ACCESS**
6. Alterovitz G, Tuthill C, Rios I, Modelska K, Sonis S. Personalized medicine for mucositis: Bayesian networks identify unique gene clusters which predict the response to gamma-D-glutamyl-L-tryptophan (SCV-07) for the attenuation of chemoradiation-induced oral mucositis. Oral Oncol. 2011 Oct;47(10):951-5. **NO ACCESS**
7. An G, Bartels J, Vodovotz Y. In Silico Augmentation of the Drug Development Pipeline: Examples from the study of Acute Inflammation. Drug Dev Res. 2011 Mar 1;72(2):187-200.
8. Anderson KC. New insights into therapeutic targets in myeloma. Hematology Am Soc Hematol Educ Program. 2011;2011:184-90. Review.
9. Andrews TD, Baird JW, Wallace WA, Harrison DJ. Routinely obtained diagnostic material as a source of RNA for personalized medicine in lung cancer patients. J Thorac Oncol. 2011 May;6(5):884-8.
10. Arar N, Seo J, Abboud HE, Parchman M, Noel P. Veterans' experience in using the online Surgeon General's family health history tool. Per Med. 2011 Sep 1;8(5):523-532.
11. Arebi N, Bullas DC, Dukes GE, Gurmany S, Hicks KJ, Kamm MA, Hobson AR. Distinct neurophysiological profiles in irritable bowel syndrome. Am J Physiol Gastrointest Liver Physiol. 2011 Jun;300(6):G1086-93.
12. Arnold GL, Vockley J. Thoroughly modern medicine. Mol Genet Metab. 2011 Sep-Oct;104(1-2):1-2.
13. Aronson SJ, Clark EH, Babb LJ, Baxter S, Farwell LM, Funke BH, Hernandez AL, Joshi VA, Lyon E, Parthum AR, Russell FJ, Varugheese M, Venman TC, Rehm HL. The GeneInsight Suite: a platform to support laboratory and provider use of DNA-based genetic testing. Hum Mutat. 2011 May;32(5):532-6. doi: 10.1002/humu.21470.
14. Arribas-Ayllon M, Sarangi S, Clarke A. Promissory accounts of personalisation in the commercialisation of genomic knowledge. Commun Med. 2011;8(1):53-66. **NO ACCESS**
15. Ashley CE, Carnes EC, Phillips GK, Padilla D, Durfee PN, Brown PA, Hanna TN, Liu J, Phillips B, Carter MB, Carroll NJ, Jiang X, Dunphy DR, Willman CL, Petsev DN, Evans DG, Parikh AN, Chackerian B, Wharton W, Peabody DS, Brinker CJ. The targeted delivery of multicomponent cargos to cancer cells by nanoporous particle-supported lipid bilayers. Nat Mater. 2011 May;10(5):389-97 **NO ACCESS**
16. Aw W, Lezhava A, Hayashizaki Y, Ishikawa T. A new trend in personalized medicine: rapid detection of SNPs in drug transporter genes by the SmartAmp method. Clin Pharmacol Ther. 2011 Apr;89(4):617-20. Epub 2011 Feb 23. Erratum in: Clin Pharmacol Ther. 2011 Jun;89(6):928. Hyashizaki, Y [corrected to Hayashizaki, Y]. **NO ACCESS**
17. Aw W, Ota I, Toyoda Y, Lezhava A, Sakai Y, Gomi T, Hayashizaki Y, Ishikawa T. Pharmacogenomics of human ABC transporters: detection of clinically important SNPs by SmartAmp2 method. Curr Pharm Biotechnol. 2011 Apr;12(4):693-704. **NO ACCESS**
18. Azmi AS, Beck FW, Sarkar FH, Mohammad RM. Network perspectives on HDM2 inhibitor chemotherapy combinations. Curr Pharm Des. 2011;17(6):640-52. **NO ACCESS**
19. Bækvad-Hansen M, Nordestgaard BG, Dahl M. Surfactant protein B polymorphisms, pulmonary function and COPD in 10,231 individuals. Eur Respir J. 2011 Apr;37(4):791-9.
20. Balmaña J, Domchek SM, Tutt A, Garber JE. Stumbling blocks on the path to personalized medicine in breast cancer: the case of PARP inhibitors for BRCA1/2-associated cancers. Cancer Discov. 2011 Jun;1(1):29-34. **NO ACCESS**
21. Baranov VS. [Personalized medicine: expectations, disappointments and hopes]. Vestn Ross Akad Med Nauk. 2011;(9):27-35. Russian.
22. Barritt AS 4th, Darling JM, Hayashi PH. New and Evolving Management Paradigms for Hepatitis C after Liver Transplantation. Curr Hepat Rep. 2011 Sep;10(3):179-185. **NO ACCESS**
23. Becla L, Lunshof JE, Gurwitz D, Schulte In den Bäumen T, Westerhoff HV, Lange BM, Brand A. Health technology assessment in the era of personalized health care. Int J Technol Assess Health Care. 2011 Apr;27(2):118-26. **NO ACCESS**
24. Bell H. Personalized medicine. Minn Med. 2011 Feb;94(2):22-7.
25. Mills RE, Pittard WS, Mullaney JM, Farooq U, Creasy TH, Mahurkar AA, Kemeza DM, Strassler DS, Ponting CP, Webber C, Devine SE. Natural genetic variation caused by small insertions and deletions in the human genome. Genome Res. 2011 Jun;21(6):830-9.
26. Bennett MR, Devarajan P. Proteomic analysis of acute kidney injury: biomarkers to mechanisms. Proteomics Clin Appl. 2011 Feb;5(1-2):67-77. doi: 10.1002/prca.201000066. Review.
27. Benson RA, Patakas A, McQueenie R, Ross K, McInnes IB, Brewer JM, Garside P. Arthritis in space and time--to boldly go! FEBS Lett. 2011 Dec 1;585(23):3640-8. Review.
28. Bergis D, Zeuzem S, Trojan J. Adding trastuzumab to standard chemotherapy in HER2-positive esophagogastric adenocarcinoma: a further step toward personalized medicine. Gastroenterology. 2011 Jan;140(1):356-8; discussion 358. **NO ACCESS**
29. Berry DA. Adaptive clinical trials in oncology. Nat Rev Clin Oncol. 2011 Nov 8;9(4):199-207. doi: 10.1038/nrclinonc.2011.165. **NO ACCESS**
30. Bertotti A, Migliardi G, Galimi F, Sassi F, Torti D, Isella C, Corà D, Di Nicolantonio F, Buscarino M, Petti C, Ribero D, Russolillo N, Muratore A, Massucco P, Pisacane A, Molinaro L, Valtorta E, Sartore-Bianchi A, Risio M, Capussotti L, Gambacorta M, Siena S, Medico E, Sapino A, Marsoni S, Comoglio PM, Bardelli A, Trusolino L. A molecularly annotated platform of patient-derived xenografts ("xenopatients") identifies HER2 as an effective therapeutic target in cetuximab-resistant colorectal cancer. Cancer Discov. 2011 Nov;1(6):508-23. **NO ACCESS**
31. Betsou F, Parida SK, Guillerm M. Infectious diseases biobanking as a catalyst towards personalized medicine: Mycobacterium tuberculosis paradigm. Tuberculosis (Edinb). 2011 Nov;91(6):524-32.
32. Bianchini G, Gianni L. Surrogate markers for targeted therapy-based treatment activity and efficacy. J Natl Cancer Inst Monogr. 2011;2011(43):91-4.
33. Biasucci LM, Della Bona R. Prognostic biomarkers in ST-segment elevation myocardial infarction: a step toward personalized medicine or a tool in search of an application? J Am Coll Cardiol. 2011 Jan 4;57(1):37-9.
34. Blazer KR, Macdonald DJ, Culver JO, Huizenga CR, Morgan RJ, Uman GC, Weitzel JN. Personalized cancer genetics training for personalized medicine: Improving community-based healthcare through a genetically literate workforce. Genet Med. 2011 May 27. **NO ACCESS**
35. Bloss CS, Jeste DV, Schork NJ. Genomics for disease treatment and prevention. Psychiatr Clin North Am. 2011 Mar;34(1):147-66. **NO ACCESS**
36. Blum HE. [Personalized medicine 2011]. Dtsch Med Wochenschr. 2011 Mar;136(10):459-60. Epub 2011 Mar 1. German.
37. Blum HE. [Personalized medicine]. Praxis (Bern 1994). 2011 Feb 2;100(3):159-66. Review. German.
38. Boersma GJ, Benthem L, van Beek AP, van Dijk G, Scheurink AJ. Personality, a key factor in personalized medicine? Eur J Pharmacol. 2011 Jun 13. **NO ACCESS**
39. Bonter K, Desjardins C, Currier N, Pun J, Ashbury FD. Personalised medicine in Canada: a survey of adoption and practice in oncology, cardiology and family medicine. BMJ Open. 2011 Jul 29;1(1):e000110.
40. Boone C. From chemical genomics to personalized medicine--tackling the genotype to phenotype problem. Mol Biosyst. 2011 Jan;7(1):13. **NO ACCESS**
41. Boone PM, Wiszniewski W, Lupski JR. Genomic medicine and neurological disease. Hum Genet. 2011 Jul;130(1):103-21.
42. Borrebaeck CA, Wingren C. Recombinant antibodies for the generation of antibody arrays. Methods Mol Biol. 2011;785:247-62. Review. **NO ACCESS**
43. Bosch E, Ezcurra D. Individualised controlled ovarian stimulation (iCOS): maximising success rates for assisted reproductive technology patients. Reprod Biol Endocrinol. 2011 Jun 21;9:82.
44. Bouganim N, Dranitsaris G, Amir E, Clemons M. Optimising the use of bone-targeted agents in patients with metastatic cancers: a practical guide for medical oncologists. Support Care Cancer. 2011 Nov;19(11):1687-96.
45. Braš M, Dorđević V, Milunović V, Brajković L, Miličić D, Konopka L. Person-centered medicine versus personalized medicine: is it just a sophism? A view from chronic pain management. Psychiatr Danub. 2011 Sep;23(3):246-50. Review.
46. Brabec P, Dusek L. [Clinical registers as a necessary support for personalized medicine]. Cesk Patol. 2011 Oct;47(4):173-5. Review. Czech.
47. Brandon D, Alazraki A, Halkar RK, Alazraki NP. The role of single-photon emission computed tomography and SPECT/computed tomography in oncologic imaging. Semin Oncol. 2011 Feb;38(1):87-108. Review.
48. Branicki W, Liu F, van Duijn K, Draus-Barini J, Pośpiech E, Walsh S, Kupiec T, Wojas-Pelc A, Kayser M. Model-based prediction of human hair color using DNA variants. Hum Genet. 2011 Apr;129(4):443-54.
49. Brennan DJ, O'Connor DP, Laursen H, McGee SF, McCarthy S, Zagozdzon R, Rexhepaj E, Culhane AC, Martin FM, Duffy MJ, Landberg G, Ryden L, Hewitt SM, Kuhar MJ, Bernards R, Millikan RC, Crown JP, Jirström K, Gallagher WM. The cocaine- and amphetamine-regulated transcript mediates ligand-independent activation of ERα and is an independent prognostic factor in node-negative breast cancer. Oncogene. 2011 Dec 5. doi: 10.1038/onc.2011.519.
50. Brothers KB. Biobanking in pediatrics: the human nonsubjects approach. Per Med. 2011 Jan;8(1):79. **NO ACCESS**
51. Browman G, Hebert PC, Coutts J, Stanbrook MB, Flegel K, Macdonald N. Personalized medicine: a windfall for science, but what about patients? CMAJ. 2011 Jul 18.
52. Brown MJ. Personalised medicine for hypertension. BMJ. 2011 Jul 28;343:d4697. doi: 10.1136/bmj.d4697.
53. Brown RE, Dorion RP, Trowbridge C, Stammers AH, Fitt W, Davis J. Algorithmic and consultative integration of transfusion medicine and coagulation: a personalized medicine approach with reduced blood component utilization. Ann Clin Lab Sci. 2011 Summer;41(3):211-6. **NO ACCESS**
54. Bühler M, Mann K. Alcohol and the Human Brain: A Systematic Review of Different Neuroimaging Methods. Alcohol Clin Exp Res. 2011 Jul 20. doi: 10.1111/j.1530-0277.2011.01540.x. **NO ACCESS**
55. Buhnik-Rosenblau K, Danin-Poleg Y, Kashi Y. Predominant effect of host genetics on levels of Lactobacillus johnsonii bacteria in the mouse gut. Appl Environ Microbiol. 2011 Sep;77(18):6531-8.
56. Butrick M, Roter D, Kaphingst K, Erby LH, Haywood C Jr, Beach MC, Levy HP. Patient reactions to personalized medicine vignettes: an experimental design. Genet Med. 2011 May;13(5):421-8.
57. Cai W, Hong H. Peptoid and Positron Emission Tomography: an Appealing Combination. Am J Nucl Med Mol Imaging. 2011;1(1):76-79.
58. Cai W, Zhang Y, Kamp TJ. Imaging of Induced Pluripotent Stem Cells: From Cellular Reprogramming to Transplantation. Am J Nucl Med Mol Imaging. 2011 Aug 1;1(1):18-28.
59. Caley CF. Interpreting and applying CYP450 genomic test results to psychotropic medications. J Pharm Pract. 2011 Oct;24(5):439-46. Epub 2011 Sep 22. Review.
60. Campbell JJ, Davidenko N, Caffarel MM, Cameron RE, Watson CJ. A multifunctional 3D co-culture system for studies of mammary tissue morphogenesis and stem cell biology. PLoS One. 2011;6(9):e25661.
61. Carlsten C, Halperin A, Crouch J, Burke W. Personalized medicine and tobacco-related health disparities: is there a role for genetics? Ann Fam Med. 2011 Jul-Aug;9(4):366-71.
62. Carr DF, Alfirevic A. Report on the 8th Annual Cold Spring Harbor/Wellcome Trust meeting on Pharmacogenomics and Personalized Medicine. Pharmacogenomics. 2011 Mar;12(3):315-7. **NO ACCESS**
63. Carreiro AV, Anunciação O, Carriço JA, Madeira SC. Prognostic prediction through biclustering-based classification of clinical gene expression time series. J Integr Bioinform. 2011 Sep 15;8(3):175. doi:10.2390/biecoll-jib-2011-175. **NO ACCESS**
64. Catenacci DV, Kozloff M, Kindler HL, Polite B. Personalized colon cancer care in 2010. Semin Oncol. 2011 Apr;38(2):284-308. Review.
65. Chan MK, Guest PC, Levin Y, Umrania Y, Schwarz E, Bahn S, Rahmoune H. Converging evidence of blood-based biomarkers for schizophrenia: An update. Int Rev Neurobiol. 2011;101:95-144. **NO ACCESS**
66. Chan SL, Suo C, Lee SC, Goh BC, Chia KS, Teo YY. Translational aspects of genetic factors in the prediction of drug response variability: a case study of warfarin pharmacogenomics in a multi-ethnic cohort from Asia. Pharmacogenomics J. 2011 Mar 8. **NO ACCESS**
67. Chapman BP, Roberts B, Duberstein P. Personality and longevity: knowns, unknowns, and implications for public health and personalized medicine. J Aging Res. 2011;2011:759170. Epub 2011 Jul 10.
68. Charron D. HLA, immunogenetics, pharmacogenetics and personalized medicine. Vox Sang. 2011 Jan;100(1):163-6. doi: 10.1111/j.1423-0410.2010.01438.x. Review.
69. Chatterjee B, Pancholi J. Prakriti-based medicine: A step towards personalized medicine. Ayu. 2011 Apr;32(2):141-6.
70. Chen C, Sun SR, Gong YP, Qi CB, Peng CW, Yang XQ, Liu SP, Peng J, Zhu S, Hu MB, Pang DW, Li Y. Quantum dots based molecular classification of breast cancer by quantitative spectroanalysis of hormone receptors and HER2. Biomaterials. 2011 Jul 9. **NO ACCESS**
71. Chen PL, Fann CS, Chu CC, Chang CC, Chang SW, Hsieh HY, Lin M, Yang WS, Chang TC. Comprehensive genotyping in two homogeneous Graves' disease samples reveals major and novel HLA association alleles. PLoS One. 2011 Jan 28;6(1):e16635. doi: 10.1371/journal.pone.0016635.
72. Chen Q, Zhang T, Wang JF, Wei DQ. Advances in human cytochrome p450 and personalized medicine. Curr Drug Metab. 2011 Jun 1;12(5):436-44. **NO ACCESS**
73. Chen R, Butte AJ. The reference human genome demonstrates high risk of type 1 diabetes and other disorders. Pac Symp Biocomput. 2011:231-42.
74. Chetty C, Rao JS, Lakka SS. Matrix metalloproteinase pharmacogenomics in non-small-cell lung carcinoma. Pharmacogenomics. 2011 Apr;12(4):535-46. doi: 10.2217/pgs.10.207. Review. **NO ACCESS**
75. Chiang A, Million RP. Personalized medicine in oncology: next generation. Nat Rev Drug Discov. 2011 Dec 1;10(12):895-6. doi: 10.1038/nrd3603.
76. Chiang PM, Wong PC. Differentiation of an embryonic stem cell to hemogenic endothelium by defined factors: essential role of bone morphogenetic protein 4. Development. 2011 Jul;138(13):2833-43. **NO ACCESS**
77. Chin L, Andersen JN, Futreal PA. Cancer genomics: from discovery science to personalized medicine. Nat Med. 2011 Mar;17(3):297-303. **NO ACCESS**
78. Chouchane L, Mamtani R, Dallol A, Sheikh JI. Personalized medicine: a patient-centered paradigm. J Transl Med. 2011 Dec 1;9:206.
79. Christ GJ. Potential applications of gene therapy/transfer to the treatment of lower urinary tract diseases/disorders. Handb Exp Pharmacol. 2011;(202):255-65. Review. **NO ACCESS**
80. Chu E. Molecular profiling and personalized medicine: from the guest editor. Cancer J. 2011 Mar-Apr;17(2):69-70. **NO ACCESS**
81. Chun YS, Byun K, Lee B. Induced pluripotent stem cells and personalized medicine: current progress and future perspectives. Anat Cell Biol. 2011 Dec;44(4):245-55. Epub 2011 Dec 30.
82. Ciardiello F, Normanno N. HER2 signaling and resistance to the anti-EGFR monoclonal antibody cetuximab: a further step toward personalized medicine for patients with colorectal cancer. Cancer Discov. 2011 Nov;1(6):472-4. **NO ACCESS**
83. Ciarimboli G. Role of organic cation transporters in drug-induced toxicity. Expert Opin Drug Metab Toxicol. 2011 Feb;7(2):159-74. **NO ACCESS**
84. Ciccolini J, Mercier C, Dahan L, André N. Integrating pharmacogenetics into gemcitabine dosing-time for a change? Nat Rev Clin Oncol. 2011 Feb 8;8(7):439-44. doi: 10.1038/nrclinonc.2011.1. **NO ACCESS**
85. Cima I, Schiess R, Wild P, Kaelin M, Schüffler P, Lange V, Picotti P, Ossola R, Templeton A, Schubert O, Fuchs T, Leippold T, Wyler S, Zehetner J, Jochum W, Buhmann J, Cerny T, Moch H, Gillessen S, Aebersold R, Krek W. Cancer genetics-guided discovery of serum biomarker signatures for diagnosis and prognosis of prostate cancer. Proc Natl Acad Sci U S A. 2011 Feb 22;108(8):3342-7.
86. Clark SL, Adkins DE, van den Oord EJ. Analysis of efficacy and side effects in CATIE demonstrates drug response subgroups and potential for personalized medicine. Schizophr Res. 2011 Nov;132(2-3):114-20. **NO ACCESS**
87. Clarke A, Thirlaway K. Genetic counselling for personalised medicine. Hum Genet. 2011 Jul;130(1):27-31.
88. Coggi PT. A European view on the future of personalised medicine in the EU. Eur J Public Health. 2011 Feb;21(1):6-7.
89. Cohen J. Dearth of clinically useful diagnostics limits growth of personalized medicine. Expert Rev Clin Pharmacol. 2011 Sep;4(5):527-9. **NO ACCESS**
90. Cohen KB, Garten Y, Hahn U, Shah NH. MINING THE PHARMACOGENOMICS LITERATURE -Workshop Introduction. Pac Symp Biocomput. 2011:362-3. **NO ACCESS**
91. Comabella M, Vandenbroeck K. Pharmacogenomics and multiple sclerosis: moving toward individualized medicine. Curr Neurol Neurosci Rep. 2011 Oct;11(5):484-91. doi: 10.1007/s11910-011-0211-1. Review. **NO ACCESS**
92. Coulter ID. Comparative effectiveness research: does the emperor have clothes? Altern Ther Health Med. 2011 Mar-Apr;17(2):8-15. **NO ACCESS**
93. Couzin-Frankel J. Personalized medicine. Pushing the envelope in neuroblastoma therapy. Science. 2011 Sep 16;333(6049):1569-71.
94. Cox KJ, Shomin CD, Ghosh I. Tinkering outside the kinase ATP box: allosteric (type IV) and bivalent (type V) inhibitors of protein kinases. Future Med Chem. 2011 Jan;3(1):29-43. Review. **NO ACCESS**
95. Curran ME, Platero S. Diagnostics and personalized medicine: observations from the World Companion Diagnostics Summit. Pharmacogenomics. 2011 Apr;12(4):465-70.
96. da Cunha Santos G, Boerner SL, Geddie WR. Maximizing the yield of lymph node cytology: Lessons learned from rapid onsite evaluation of image- and endoscopic-guided biopsies of hilar and mediastinal lymph nodes. Cancer Cytopathol. 2011 Jun 29. doi: 10.1002/cncy.20166. **NO ACCESS**
97. Damia G, Broggini M, Marsoni S, Venturini S, Generali D. New omics information for clinical trial utility in the primary setting. J Natl Cancer Inst Monogr. 2011;2011(43):128-33.
98. Davis AP, Rosenstein MC, Wiegers TC, Mattingly CJ. DiseaseComps: a metric that discovers similar diseases based upon common toxicogenomic profiles at CTD. Bioinformation. 2011;7(4):154-6. Epub 2011 Oct 14.
99. Davis DA, Chawla NV. Exploring and exploiting disease interactions from multi-relational gene and phenotype networks. PLoS One. 2011;6(7):e22670.
100. DE LA Vega FM, Bustamante CD, Leal SM. GENOME-WIDE ASSOCIATION MAPPING AND RARE ALLELES: FROM POPULATION GENOMICS TO PERSONALIZED MEDICINE – Session Introduction. Pac Symp Biocomput. 2011:74-5.
101. de Rooy DP, van der Linden MP, Knevel R, Huizinga TW, van der Helm-van Mil AH. Predicting arthritis outcomes--what can be learned from the Leiden Early Arthritis Clinic? Rheumatology (Oxford). 2011 Jan;50(1):93-100. **NO ACCESS**
102. de Souza JA, Olopade OI. CYP2D6 genotyping and tamoxifen: an unfinished story in the quest for personalized medicine. Semin Oncol. 2011 Apr;38(2):263-73. Review.
103. Degoma EM, Rivera G, Lilly SM, Usman MH, Mohler ER 3rd. Personalized vascular medicine: individualizing drug therapy. Vasc Med. 2011 Oct;16(5):391-404. doi: 10.1177/1358863X11422251. Review.
104. Delbaldo C, Albert S, Dreyer C, Sablin MP, Serova M, Raymond E, Faivre S. Predictive biomarkers for the activity of mammalian target of rapamycin (mTOR) inhibitors. Target Oncol. 2011 Jun;6(2):119-24. **NO ACCESS**
105. Denman B, Goodman SR. Emerging and neglected tropical diseases: translational application of proteomics. Exp Biol Med (Maywood). 2011 Jul 7. **NO ACCESS**
106. Di Martino MT, Arbitrio M, Leone E, Guzzi PH, Rotundo MS, Ciliberto D, Tomaino V, Fabiani F, Talarico D, Sperlongano P, Doldo P, Cannataro M, Caraglia M, Tassone P, Tagliaferri P. Single nucleotide polymorphisms of ABCC5 and ABCG1 transporter genes correlate to irinotecan-associated gastrointestinal toxicity in colorectal cancer patients: a DMET microarray profiling study. Cancer Biol Ther. 2011 Nov 1;12(9):780-7.
107. Ding Q, Luckhardt T, Hecker L, Zhou Y, Liu G, Antony VB, Deandrade J, Thannickal VJ. New insights into the pathogenesis and treatment of idiopathic pulmonary fibrosis. Drugs. 2011 May 28;71(8):981-1001. doi: 10.2165/11591490-000000000-00000. **NO ACCESS**
108. Distefano JK, Watanabe RM. Pharmacogenetics of Anti-Diabetes Drugs. Pharmaceuticals (Basel). 2010 Aug 1;3(8):2610-2646.
109. Dizdarevic S, Peters AM. Imaging of multidrug resistance in cancer. Cancer Imaging. 2011 Mar 1;11:1-8. Review. **NO ACCESS**
110. Dodgen TM, Cromarty AD, Pepper MS. Quantitative plasma analysis using automated online solid-phase extraction with column switching LC-MS/MS for characterising cytochrome P450 2D6 and 2C19 metabolism. J Sep Sci. 2011 May;34(10):1102-10. doi: 10.1002/jssc.201000920.
111. Donnan JR, Ungar WJ, Mathews M, Hancock-Howard RL, Rahman P. A cost effectiveness analysis of thiopurine methyltransferase testing for guiding 6-mercaptopurine dosing in children with acute lymphoblastic leukemia. Pediatr Blood Cancer. 2011 Aug;57(2):231-9. doi: 10.1002/pbc.22936.
112. Donnan JR, Ungar WJ, Mathews M, Rahman P. Systematic review of thiopurine methyltransferase genotype and enzymatic testing strategies. Ther Drug Monit. 2011 Apr;33(2):192-9.
113. Donovan MJ, Costa J, Cordon-Cardo C. Personalized approach to prostate cancer prognosis. Arch Esp Urol. 2011 Oct;64(8):783-91. Review. English, Spanish. **NO ACCESS**
114. Doughty E, Kertesz-Farkas A, Bodenreider O, Thompson G, Adadey A, Peterson T, Kann MG. Toward an automatic method for extracting cancer- and other disease-related point mutations from the biomedical literature. Bioinformatics. 2011 Feb 1;27(3):408-15. **NO ACCESS**
115. Dowd SE, Wolcott RD, Kennedy J, Jones C, Cox SB. Molecular diagnostics and personalised medicine in wound care: assessment of outcomes. J Wound Care. 2011 May;20(5):232, 234-9. **NO ACCESS**
116. Drake RR, Cazares LH, Jones EE, Fuller TW, Semmes OJ, Laronga C. Challenges to developing proteomic-based breast cancer diagnostics. OMICS. 2011 May;15(5):251-9. **NO ACCESS**
117. Dubois E, Fertin M, Burdese J, Amouyel P, Bauters C, Pinet F. Cardiovascular proteomics: translational studies to develop novel biomarkers in heart failure and left ventricular remodeling. Proteomics Clin Appl. 2011 Feb;5(1-2):57-66. doi: 10.1002/prca.201000056.
118. Duchesne GM, Wang SC, Hicks RJ. Imaging, radiation oncology and randomised trials: time for a change? J Med Imaging Radiat Oncol. 2011 Feb;55(1):97-100. doi: 10.1111/j.1754-9485.2010.02235.x.
119. Dupouy S, Mourra N, Doan VK, Gompel A, Alifano M, Forgez P. The potential use of the neurotensin high affinity receptor 1 as a biomarker for cancer progression and as a component of personalized medicine in selective cancers. Biochimie. 2011 Sep;93(9):1369-78. **NO ACCESS**
120. Ebben JD, Zorniak M, Clark PA, Kuo JS. Introduction to induced pluripotent stem cells: advancing the potential for personalized medicine. World Neurosurg. 2011 Sep-Oct;76(3-4):270-5. Review.
121. Eicher D, Merten CA. Microfluidic devices for diagnostic applications. Expert Rev Mol Diagn. 2011 Jun;11(5):505-19. **NO ACCESS**
122. Eifler AC, Thaxton CS. Nanoparticle therapeutics: FDA approval, clinical trials, regulatory pathways, and case study. Methods Mol Biol. 2011;726:325-38. **NO ACCESS**
123. Elahi SF, Wang TD. Future and advances in endoscopy. J Biophotonics. 2011 Aug;4(7-8):471-81. doi: 10.1002/jbio.201100048.
124. Elespuru RK. Assessment of heritable genetic effects using new genetic tools and sentinels in an era of personalized medicine. Environ Mol Mutagen. 2011 May;52(4):253-63. doi: 10.1002/em.20637.
125. Elespuru RK. Assessment of heritable genetic effects using new genetic tools and sentinels in an era of personalized medicine. Environ Mol Mutagen. 2011 Jan 6.
126. Engstrom PF, Bloom MG, Demetri GD, Febbo PG, Goeckeler W, Ladanyi M, Loy B, Murphy K, Nerenberg M, Papagni P, Robson M, Sweetman RW, Tunis S, Demartino J, Larsen JK; National Comprehensive Cancer Network. NCCN molecular testing white paper: effectiveness, efficiency, and reimbursement. J Natl Compr Canc Netw. 2011 Dec;9 Suppl 6:S1-16.
127. European Society of Radiology. Medical imaging in personalised medicine: a white paper of the research committee of the European Society of Radiology (ESR). Insights Imaging. 2011 Dec;2(6):621-630.
128. Facio FM, Brooks S, Loewenstein J, Green S, Biesecker LG, Biesecker BB. Motivators for participation in a whole-genome sequencing study: implications for translational genomics research. Eur J Hum Genet. 2011 Jul 6. doi: 10.1038/ejhg.2011.123. **NO ACCESS**
129. Fahim RE, Kessler PD, Fuller SA, Kalnik MW. Nicotine vaccines. CNS Neurol Disord Drug Targets. 2011 Dec;10(8):905-15. **NO ACCESS**
130. Feehan M, Hartman J, Durante R, Morrison MA, Miller JW, Kim IK, Deangelis MM. Identifying subtypes of patients with neovascular age-related macular degeneration by genotypic and cardiovascular risk characteristics. BMC Med Genet. 2011 Jun 17;12:83.
131. Fenstermacher DA, Wenham RM, Rollison DE, Dalton WS. Implementing personalized medicine in a cancer center. Cancer J. 2011 Nov-Dec;17(6):528-36. Review.
132. Fernald GH, Capriotti E, Daneshjou R, Karczewski KJ, Altman RB. Bioinformatics challenges for personalized medicine. Bioinformatics. 2011 Jul 1;27(13):1741-8. Epub 2011 May 19.
133. Ferreira L, Quiros Y, Sancho-Martínez SM, García-Sánchez O, Raposo C, López-Novoa JM, González-Buitrago JM, López-Hernández FJ. Urinary levels of regenerating islet-derived protein III β and gelsolin differentiate gentamicin from cisplatin-induced acute kidney injury in rats. Kidney Int. 2011 Mar;79(5):518-28. **NO ACCESS**
134. Fiţa IG, Enciu AM, Stănoiu BP.New insights on Alzheimer's disease diagnostic. Rom J Morphol Embryol. 2011;52(3 Suppl):975-9. Review.
135. Finley SD, Engel-Stefanini MO, Imoukhuede PI, Popel AS. Pharmacokinetics and pharmacodynamics of VEGF-neutralizing antibodies. BMC Syst Biol. 2011 Nov 21;5:193.
136. Fiore LD, D'Avolio LW. Detours on the road to personalized medicine: barriers to biomarker validation and implementation. JAMA. 2011 Nov 2;306(17):1914-5. P
137. Fisher R, Larkin J, Swanton C. Delivering preventive, predictive and personalised cancer medicine for renal cell carcinoma: the challenge of tumour heterogeneity. EPMA J. 2011 Dec 22;3(1):1.
138. Flaig TW, Theodorescu D. Bladder cancer in 2011: the dawn of personalized medicine. Nat Rev Urol. 2011 Dec 20;9(2):65-6. doi: 10.1038/nrurol.2011.220. Review. **NO ACCESS**
139. Fountain J, Gunby P. Improving clinician-patient communication of health risks when diagnostic test information is imprecise. N Z Med J. 2011 Sep 9;124(1342):39-47. **NO ACCESS**
140. Fröhlich H. Network based consensus gene signatures for biomarker discovery in breast cancer. PLoS One. 2011;6(10):e25364.
141. Franc MA, Warner AW, Cohen N, Shaw PM, Groenen P, Snapir A. Current practices for DNA sample collection and storage in the pharmaceutical industry, and potential areas for harmonization: perspective of the I-PWG. Clin Pharmacol Ther. 2011 Apr;89(4):546-53. **NO ACCESS**
142. Freire AC, Basit AW, Choudhary R, Piong CW, Merchant HA. Does sex matter? The influence of gender on gastrointestinal physiology and drug delivery. Int J Pharm. 2011 Aug 30;415(1-2):15-28. Review.
143. Frueh FW. Considerations for safety pharmacogenetics in clinical practice. Drug Discov Today. 2011 Oct;16(19-20):898-901. Epub 2011 Aug 24. Review.
144. Früh M. The search for improved systemic therapy of non-small cell lung cancer--what are today's options? Lung Cancer. 2011 Jun;72(3):265-70. **NO ACCESS**
145. Fueyo J, Gomez-Manzano C, Yung WK. Advances in translational research in neuro-oncology. Arch Neurol. 2011 Mar;68(3):303-8. Review.
146. Gajria D, Chandarlapaty S. HER2-amplified breast cancer: mechanisms of trastuzumab resistance and novel targeted therapies. Expert Rev Anticancer Ther. 2011 Feb;11(2):263-75.
147. Galvão ER, Martins LM, Ibiapina JO, Andrade HM, Monte SJ. Breast cancer proteomics: a review for clinicians. J Cancer Res Clin Oncol. 2011 Jun;137(6):915-25. **NO ACCESS**
148. Gambarin-Gelwan M, Jacobson IM. Chronic hepatitis C genotype 2 and 3: Are we ready for personalized medicine? J Hepatol. 2011 Apr 15. **NO ACCESS**
149. Gao Y, Chen JC, Wang Z, Peng HY, Li M, Zhu ZY, Chen HY. [Genotyping for CYP2C19 with multiplex PCR point mutation screening technology]. Zhonghua Yi Xue Za Zhi. 2011 Aug 30;91(32):2293-6. Chinese.
150. Garg V, Arora S, Gupta C. Cloud computing approaches to accelerate drug discovery value chain. Comb Chem High Throughput Screen. 2011 Dec;14(10):861-71. Review. **NO ACCESS**
151. Generali D, Symmans WF, Berruti A, Fox SB. Predictive immunohistochemical biomarkers in the context of neoadjuvant therapy for breast cancer. J Natl Cancer Inst Monogr. 2011;2011(43):99-102.
152. George B, Kopetz S. Predictive and prognostic markers in colorectal cancer. Curr Oncol Rep. 2011 Jun;13(3):206-15. **NO ACCESS**
153. Ginghina C, Bejan I, Ceck CD. Modern risk stratification in coronary heart disease. J Med Life. 2011 Nov 14;4(4):377-86. Epub 2011 Nov 24.
154. Ginghină C, Botezatu CD, Șerban M, Jurcuţ R. A personalized medicine target: heart failure in women. J Med Life. 2011 Aug 15;4(3):280-6. Epub 2011 Aug 25. PubMed PMID: 22567052; PubMed Central PMCID: PMC3168816.
155. Glubb DM, Innocenti F. Mechanisms of genetic regulation in gene expression: examples from drug metabolizing enzymes and transporters. Wiley Interdiscip Rev Syst Biol Med. 2011 May-Jun;3(3):299-313. doi: 10.1002/wsbm.125 **NO ACCESS**
156. Glück S. Does the addition of chemotherapy to adjuvant endocrine treatment add any benefit in ER-positive early breast cancer: can we rely on large randomized control trials in the era of personalized medicine? Ann Oncol. 2011 Jul 15. **NO ACCESS**
157. Glunde K, Bhujwalla ZM. Metabolic tumor imaging using magnetic resonance spectroscopy. Semin Oncol. 2011 Feb;38(1):26-41. Review.
158. Gollust SE, Gordon ES, Zayac C, Griffin G, Christman MF, Pyeritz RE, Wawak L, Bernhardt BA. Motivations and Perceptions of Early Adopters of Personalized Genomics: Perspectives from Research Participants. Public Health Genomics. 2011 Jun 3. **NO ACCESS**
159. Gómez-Barrena E, Rosset P, Müller I, Giordano R, Bunu C, Layrolle P, Konttinen YT, Luyten FP. Bone regeneration: stem cell therapies and clinical studies in orthopaedics and traumatology. J Cell Mol Med. 2011 Jun;15(6):1266-86. doi: 10.1111/j.1582-4934.2011.01265.x. **NO ACCESS**
160. Gómez-Durán A, Pacheu-Grau D, López-Pérez MJ, Montoya J, Ruiz-Pesini E. Mitochondrial pharma-Q-genomics: targeting the OXPHOS cytochrome b. Drug Discov Today. 2011 Mar;16(5-6):176-80. **NO ACCESS**
161. Gonzalez A, Clemente JC, Shade A, Metcalf JL, Song S, Prithiviraj B, Palmer BE, Knight R. Our microbial selves: what ecology can teach us. EMBO Rep. 2011 Jul 1. doi: 10.1038/embor.2011.137.
162. Gossett DR, Weaver WM, Ahmed NS, Di Carlo D. Sequential array cytometry: multi-parameter imaging with a single fluorescent channel. Ann Biomed Eng. 2011 Apr;39(4):1328-34.
163. Gottlieb A, Stein GY, Ruppin E, Sharan R. PREDICT: a method for inferring novel drug indications with application to personalized medicine. Mol Syst Biol. 2011 Jun 7;7:496. doi: 10.1038/msb.2011.26.
164. Grady BJ, Ritchie MD. Statistical Optimization of Pharmacogenomics Association Studies: Key Considerations from Study Design to Analysis. Curr Pharmacogenomics Person Med. 2011 Mar 1;9(1):41-66.
165. Grady BJ, Torstenson ES, McLaren PJ, DE Bakker PI, Haas DW, Robbins GK, Gulick RM, Haubrich R, Ribaudo H, Ritchie MD. Use of biological knowledge to inform the analysis of gene-gene interactions involved in modulating virologic failure with efavirenz-containing treatment regimens in art-naïve actg clinical trials participants. Pac Symp Biocomput. 2011:253-64.
166. Gridelli C, De Marinis F, Di Maio M, Cortinovis D, Cappuzzo F, Mok T. Gefitinib as first-line treatment for patients with advanced non-small-cell lung cancer with activating Epidermal Growth Factor Receptor mutation: implications for clinical practice and open issues. Lung Cancer. 2011 Apr;72(1):3-8. **NO ACCESS**
167. Groenen PJ, Blokx WA, Diepenbroek C, Burgers L, Visinoni F, Wesseling P, van Krieken JH. Preparing pathology for personalized medicine: possibilities for improvement of the pre-analytical phase. Histopathology. 2011 Jul;59(1):1-7. doi: 10.1111/j.1365-2559.2010.03711.x.
168. Groenen PM. Four decades of modern pharmacogenomics: from promise to clinical utility. World PGX Summit 2011. Boston, USA, 26-27 April, 2011. Pharmacogenomics. 2011 Sep;12(9):1249-52.
169. Gruver AM, Portier BP, Tubbs RR. Molecular pathology of breast cancer: the journey from traditional practice toward embracing the complexity of a molecular classification. Arch Pathol Lab Med. 2011 May;135(5):544-57. Review.
170. Guest PC, Martins-de-Souza D, Vanattou-Saifoudine N, Harris LW, Bahn S. Abnormalities in metabolism and hypothalamic-pituitary-adrenal axis function in schizophrenia. Int Rev Neurobiol. 2011;101:145-68. **NO ACCESS**
171. Guest PC, Schwarz E, Krishnamurthy D, Harris LW, Leweke FM, Rothermundt M, van Beveren NJ, Spain M, Barnes A, Steiner J, Rahmoune H, Bahn S. Altered levels of circulating insulin and other neuroendocrine hormones associated with the onset of schizophrenia. Psychoneuroendocrinology. 2011 Aug;36(7):1092-6. **NO ACCESS**
172. Gunter L, Zhu J, Murphy S. Variable selection for qualitative interactions in personalized medicine while controlling the family-wise error rate. J Biopharm Stat. 2011 Nov;21(6):1063-78. **NO ACCESS**
173. Gur RC, Irani F, Seligman S, Calkins ME, Richard J, Gur RE. Challenges and opportunities for genomic developmental neuropsychology: examples from the Penn-Drexel collaborative battery. Clin Neuropsychol. 2011 Aug;25(6):1029-41. Epub 2011 Sep 9. Review. **NO ACCESS**
174. Gurwitz D, Lunshof JE. Personalized participatory medicine: sharing knowledge and uncertainty. Genome Med. 2011 Oct 27;3(10):69.
175. Haga SB, Kawamoto K, Agans R, Ginsburg GS. Consideration of patient preferences and challenges in storage and access of pharmacogenetic test results. Genet Med. 2011 Jun 13. **NO ACCESS**
176. Hage DS, Anguizola J, Barnaby O, Jackson A, Yoo MJ, Papastavros E, Pfaunmiller E, Sobansky M, Tong Z. Characterization of drug interactions with serum proteins by using high-performance affinity chromatography. Curr Drug Metab. 2011 May;12(4):313-28. **NO ACCESS**
177. Haidar AN, Zasada SJ, Coveney PV, Abdallah AE, Beckles B, Jones MA. Audited credential delegation: a usable security solution for the virtual physiological human toolkit. Interface Focus. 2011 Jun 6;1(3):462-73.
178. Hanash S, Taguchi A. Application of proteomics to cancer early detection. Cancer J. 2011 Nov-Dec;17(6):423-8. **NO ACCESS**
179. Hanash SM, Baik CS, Kallioniemi O. Emerging molecular biomarkers--blood-based strategies to detect and monitor cancer. Nat Rev Clin Oncol. 2011 Mar;8(3):142-50. doi: 10.1038/nrclinonc.2010.220. Review.
180. Hankowski KE, Hamazaki T, Umezawa A, Terada N. Induced pluripotent stem cells as a next-generation biomedical interface. Lab Invest. 2011 Jul;91(7):972-7. doi: 10.1038/labinvest.2011.85.
181. Hann MC, Lau PE, Tempest HG. Meiotic recombination and male infertility: from basic science to clinical reality? Asian J Androl. 2011 Mar;13(2):212-8. **NO ACCESS**
182. Heidbuchel H. Telemonitoring of implantable cardiac devices: hurdles towards personalised medicine. Heart. 2011 Jun;97(11):931-9. Review.
183. Henderson R, Schumacher M. Clinical epidemiology and individualized medicine. Biom J. 2011 Mar;53(2):167-9. doi: 10.1002/bimj.201000257. Epub 2011 Feb 11. **NO ACCESS**
184. Heneberg P. [Advances in clinical treatment of malignant melanoma: B-RAF kinase inhibition]. Klin Onkol. 2011;24(4):256-64. Review. Czech.
185. Hester RL, Iliescu R, Summers R, Coleman TG. Systems biology and integrative physiological modelling. J Physiol. 2011 Mar 1;589(Pt 5):1053-60. **NO ACCESS**
186. Heuveling DA, de Bree R, van Dongen GA. The potential role of non-FDG-PET in the management of head and neck cancer. Oral Oncol. 2011 Jan;47(1):2-7.
187. Hewitt RE. Biobanking: the foundation of personalized medicine. Curr Opin Oncol. 2011 Jan;23(1):112-9. Review.
188. Hinneburg I. [Individualized medicine between expectations and reality]. Med Monatsschr Pharm. 2011 Jun;34(6):212-4. German.
189. Ho CL, Liu IH, Wu YH, Chen LC, Chen CL, Lee WC, Chuang CH, Lee TW, Lin WJ, Shen LH, Chang CH. Molecular Imaging, Pharmacokinetics, and Dosimetry of In-AMBA in Human Prostate Tumor-Bearing Mice. J Biomed Biotechnol. 2011;2011:101497.
190. Hoffman MA, Williams MS. Electronic medical records and personalized medicine. Hum Genet. 2011 Jul;130(1):33-9.
191. Hoggatt J. Personalized medicine--trends in molecular diagnostics: exponential growth expected in the next ten years. Mol Diagn Ther. 2011 Feb 1;15(1):53-5. doi: 10.2165/11534880-000000000-00000. PubMed PMID: 21469770.
192. Holbrook JD, Parker JS, Gallagher KT, Halsey WS, Hughes AM, Weigman VJ, Lebowitz PF, Kumar R. Deep sequencing of gastric carcinoma reveals somatic mutations relevant to personalized medicine. J Transl Med. 2011 Jul 25;9(1):119.
193. Hong ML, Song Y, Li XM. Effects and mechanisms of actions of Chinese herbal medicines for asthma. Chin J Integr Med. 2011 Jul;17(7):483-91.
194. Hoppe R, Brauch H, Kroetz DL, Esteller M. Exploiting the complexity of the genome and transcriptome using pharmacogenomics towards personalized medicine. Genome Biol. 2011 Jan 17;12(1):301. **NO ACCESS**
195. Hulse NC, Ranade-Kharkar P, Post H, Wood GM, Williams MS, Haug PJ. Development and early usage patterns of a consumer-facing family health history tool. AMIA Annu Symp Proc. 2011;2011:578-87.
196. Hunter P. A new target for personalized medicine. EMBO Rep. 2011 Dec 1;12(12):1229-32. doi: 10.1038/embor.2011.219.
197. Ihle NT, Powis G, Kopetz S. PI-3-Kinase inhibitors in colorectal cancer. Curr Cancer Drug Targets. 2011 Feb;11(2):190-8. Review. **NO ACCESS**
198. Inoue H, Yamanaka S. The use of induced pluripotent stem cells in drug development. Clin Pharmacol Ther. 2011 May;89(5):655-61. **NO ACCESS**
199. Institute of Medicine (US) Forum on Neuroscience and Nervous System Disorders. Sex Differences and Implications for Translational Neuroscience Research: Workshop Summary. Washington (DC): National Academies Press (US); 2011. **NO ACCESS**
200. Isaacs JD, Ferraccioli G. The need for personalised medicine for rheumatoid arthritis. Ann Rheum Dis. 2011 Jan;70(1):4-7.
201. Itzhaki I, Maizels L, Huber I, Zwi-Dantsis L, Caspi O, Winterstern A, Feldman O, Gepstein A, Arbel G, Hammerman H, Boulos M, Gepstein L. Modelling the long QT syndrome with induced pluripotent stem cells. Nature. 2011 Mar 10;471(7337):225-9. **NO ACCESS**
202. Itzhaki I, Rapoport S, Huber I, Mizrahi I, Zwi-Dantsis L, Arbel G, Schiller J, Gepstein L. Calcium handling in human induced pluripotent stem cell derived cardiomyocytes. PLoS One. 2011 Apr 1;6(4):e18037.
203. Ivandic B, Frey N. [New approaches and indications for the analysis of platelet function in cardiology]. Hamostaseologie. 2011 May 2;31(2):73-6. Epub 2010 Dec 9. German.
204. Jørgensen JT. A challenging drug development process in the era of personalized medicine. Drug Discov Today. 2011 Oct;16(19-20):891-7. Epub 2011 Sep 18. Review.
205. Jackson DB, Sood AK. Personalized cancer medicine--advances and socio-economic challenges. Nat Rev Clin Oncol. 2011 Oct 11;8(12):735-41. doi: 10.1038/nrclinonc.2011.151.
206. Jain KK. Nanobiotechnology and personalized medicine. Prog Mol Biol TranslSci. 2011;104:325-54. Review. **NO ACCESS**
207. Jain KK. The role of nanobiotechnology in the development of personalized medicine. Med Princ Pract. 2011;20(1):1-3.
208. Jamshidi N, Miller FJ, Mandel J, Evans T, Kuo MD. Individualized therapy of HHT driven by network analysis of metabolomic profiles. BMC Syst Biol. 2011 Dec 20;5:200. **NO ACCESS**
209. Janku F, Garrido-Laguna I, Petruzelka LB, Stewart DJ, Kurzrock R. Novel therapeutic targets in non-small cell lung cancer. J Thorac Oncol. 2011 Sep;6(9):1601-12. Review. **NO ACCESS**
210. Janssens AC, Wilde AA, van Langen IM. The sense and nonsense of direct-to-consumer genetic testing for cardiovascular disease. Neth Heart J. 2011 Feb;19(2):85-88.
211. Jayasinghe SN. Bio-electrosprays: from bio-analytics to a generic tool for the health sciences. Analyst. 2011 Mar 7;136(5):878-90. Epub 2011 Jan 27. Review.
212. Jenkins M, Flynn A, Smart T, Harbron C, Sabin T, Ratnayake J, Delmar P, Herath A, Jarvis P, Matcham J; PSI Biomarker Special Interest Group. A statistician's perspective on biomarkers in drug development. Pharm Stat. 2011 Nov-Dec;10(6):494-507. doi: 10.1002/pst.532.
213. Jones BL. The impact of pharmacogenetics in the treatment of allergic disease and asthma. Mo Med. 2011 Sep-Oct;108(5):361-5. **NO ACCESS**
214. Jones DS. How Personalized Medicine Became Genetic, and Racial: Werner Kalow and the Formations of Pharmacogenetics. J Hist Med Allied Sci. 2011 Sep 10.
215. Jones LK, Zou F, Kheifets A, Rybnikov K, Berry D, Tan AC. Confident predictability: identifying reliable gene expression patterns for individualized tumor classification using a local minimax kernel algorithm. BMC Med Genomics. 2011 Jan 24;4:10.
216. Jones LP, Stefansson S, Kim MS, Ahn SN. Comparison of radioimmuno and carbon nanotube field-effect transistor assays for measuring insulin-like growth factor-1 in a preclinical model of human breast cancer. J Nanobiotechnology. 2011 Sep 2;9:36.
217. Joyner MJ. Exercise testing and disease risk: individualized medicine without the "omics"? J Appl Physiol. 2011 Dec;111(6):1539. Epub 2011 Sep 22. **NO ACCESS**
218. Kaldy J. Personalized medicine: genetic component to providing quality care. Consult Pharm. 2011 Sep;26(9):618-27. **NO ACCESS**
219. Kamat AM, Mathew P. Bladder cancer: imperatives for personalized medicine. Oncology (Williston Park). 2011 Sep;25(10):951-8, 960. Review. **NO ACCESS**
220. Kang JG, Ko JH, Kim YS. Pros and cons of using aberrant glycosylation as companion biomarkers for therapeutics in cancer. BMB Rep. 2011 Dec;44(12):765-71. Review.
221. Kateb B, Chiu K, Black KL, Yamamoto V, Khalsa B, Ljubimova JY, Ding H, Patil R, Portilla-Arias JA, Modo M, Moore DF, Farahani K, Okun MS, Prakash N, Neman J, Ahdoot D, Grundfest W, Nikzad S, Heiss JD. Nanoplatforms for constructing new approaches to cancer treatment, imaging, and drug delivery: what should be the policy? Neuroimage. 2011 Jan;54 Suppl 1:S106-24. Review. **NO ACCESS**
222. Kato H, Nishimura T, Ikeda N, Yamada T, Kondo T, Saijo N, Nishio K, Fujimoto J, Nomura M, Oda Y, Lindmark B, Maniwa J, Hibino H, Unno M, Ito T, Sawa Y, Tojo H, Egawa S, Edula G, Lopez M, Wigmore M, Inase N, Yoshizawa Y, Nomura F, Marko-Varga G. Developments for a growing Japanese patient population: facilitating new technologies for future health care. J Proteomics. 2011 May 16;74(6):759-64.
223. Kawai K. [Regulation of pharmacokinetics and targeting-confirmative personalized medicine]. Nippon Hoshasen Gijutsu Gakkai Zasshi. 2011;67(3):249-59. Japanese.
224. Keld RR, Ang YS. Targeting key signalling pathways in oesophageal adenocarcinoma: a reality for personalised medicine? World J Gastroenterol. 2011 Jun 21;17(23):2781-90.
225. Kelkar SS, Reineke TM. Theranostics: combining imaging and therapy. Bioconjug Chem. 2011 Oct 19;22(10):1879-903.
226. Kelley RK, Van Bebber SL, Phillips KA, Venook AP. Personalized medicine and oncology practice guidelines: a case study of contemporary biomarkers in colorectal cancer. J Natl Compr Canc Netw. 2011 Jan;9(1):13-25. **NO ACCESS**
227. Kennedy MJ, Phan H, Benavides S, Potts A, Sorensen S. The role of the pediatric pharmacist in personalized medicine and clinical pharmacogenomics for children: pediatric pharmacogenomics working group. J Pediatr Pharmacol Ther. 2011 Apr;16(2):118-22.
228. Khalil MM, Tremoleda JL, Bayomy TB, Gsell W. Molecular SPECT Imaging: An Overview. Int J Mol Imaging. 2011;2011:796025.
229. Khan NA, Peterson JF. A surveillance tool to support quality assurance and research in personalized medicine. AMIA Annu Symp Proc. 2011;2011:701-8.
230. Kiesslich R, Goetz M, Hoffman A, Galle PR. New imaging techniques and opportunities in endoscopy. Nat Rev Gastroenterol Hepatol. 2011 Sep 6;8(10):547-53. doi: 10.1038/nrgastro.2011.152. Review. **NO ACCESS**
231. Kim ST, Lim do H, Jang KT, Lim T, Lee J, Choi YL, Jang HL, Yi JH, Baek KK, Park SH, Park YS, Lim HY, Kang WK, Park JO. Impact of KRAS mutations on clinical outcomes in pancreatic cancer patients treated with first-line gemcitabine-based chemotherapy. Mol Cancer Ther. 2011 Oct;10(10):1993-9. **NO ACCESS**
232. Kinkorová J. [European Association for Predictive Preventive & Personalised Medicine. Personalised medicine in the European Union]. Cas Lek Cesk. 2011;150(7):402-4. Czech.
233. Kinkorová J. [Preparation of the Eighth Framework Programme of the European Union--Health Priority. European Commission organizes proposal of aims in biomedical research prospecting to 2020]. Cas Lek Cesk. 2011;150(9):502-5. Czech.
234. Kirilly E, Gonda X, Bagdy G. CB(1) receptor antagonists: new discoveries leading to new perspectives. Acta Physiol (Oxf). 2011 Dec 26. doi: 10.1111/j.1748-1716.2011.02402.x.
235. Kitazawa K. [Drug safety--from patients' perspective]. Yakugaku Zasshi. 2011;131(6):881-3. Japanese.
236. Komatsu H, Nakayama K, Togari T, Suzuki K, Hayashi N, Murakami Y, Iioka Y, Osaka W, Yagasaki K, Nakamura S, Neumann J, Ueno NT. Information sharing and case conference among the multidisciplinary team improve patients' perceptions of care. Open Nurs J. 2011;5:79-85.
237. Kondo J, Endo H, Okuyama H, Ishikawa O, Iishi H, Tsujii M, Ohue M, Inoue M. Retaining cell-cell contact enables preparation and culture of spheroids composed of pure primary cancer cells from colorectal cancer. Proc Natl Acad Sci U S A. 2011 Apr 12;108(15):6235-40.
238. Konoshita T; the Genomic Disease Outcome Consortium (G-DOC) Study Investigators. Do Genetic Variants of the Renin-Angiotensin System Predict Blood Pressure Response to Renin-Angiotensin System-Blocking Drugs? A Systematic Review of Pharmacogenomics in the Renin-Angiotensin System. Curr Hypertens Rep. 2011 May 13. **NO ACCESS**
239. Konstantopoulos N, Foletta VC, Segal DH, Shields KA, Sanigorski A, Windmill K, Swinton C, Connor T, Wanyonyi S, Dyer TD, Fahey RP, Watt RA, Curran JE, Molero JC, Krippner G, Collier GR, James DE, Blangero J, Jowett JB, Walder KR. A gene expression signature for insulin resistance. Physiol Genomics. 2011 Feb 11;43(3):110-20.
240. Koo H, Huh MS, Sun IC, Yuk SH, Choi K, Kim K, Kwon IC. In vivo targeted delivery of nanoparticles for theranosis. Acc Chem Res. 2011 Oct 18;44(10):1018-28.
241. Koziol JA. New tricks with old dogs: personalised medicine and clinical trials. Br J Cancer. 2011 Aug 23;105(5):599-601. doi: 10.1038/bjc.2011.299.
242. Kraft M. Asthma phenotypes and interleukin-13--moving closer to personalized medicine. N Engl J Med. 2011 Sep 22;365(12):1141-4.
243. Kuderer NM, Lyman GH. Personalized medicine and cancer supportive care: appropriate use of colony-stimulating factor support of chemotherapy. J Natl Cancer Inst. 2011 Jun 22;103(12):910-3.
244. Kumar CS, Mohammad F. Magnetic nanomaterials for hyperthermia-based therapy and controlled drug delivery. Adv Drug Deliv Rev. 2011 Aug 14;63(9):789-808. **NO ACCESS**
245. Kumar D. The personalised medicine. A paradigm of evidence-based medicine. Ann Ist Super Sanita. 2011;47(1):31-40. Review.
246. Kumar M, Zhao X, Wang XW. Molecular carcinogenesis of hepatocellular carcinoma and intrahepatic cholangiocarcinoma: one step closer to personalized medicine? Cell Biosci. 2011 Jan 24;1(1):5.
247. Kupczyk M, Dahlén B, Dahlén SE. Which anti-inflammatory drug should we use in asthma? Pol Arch Med Wewn. 2011 Dec;121(12):455-9. Review. **NO ACCESS**
248. La Thangue NB, Kerr DJ. Predictive biomarkers: a paradigm shift towards personalized cancer medicine. Nat Rev Clin Oncol. 2011 Aug 23;8(10):587-96. doi: 10.1038/nrclinonc.2011.121. Review. **NO ACCESS**
249. Ladabaum U, Wang G, Terdiman J, Blanco A, Kuppermann M, Boland CR, Ford J, Elkin E, Phillips KA. Strategies to identify the lynch syndrome among patients with colorectal cancer: a cost-effectiveness analysis. Ann Intern Med. 2011 Jul 19;155(2):69-79.
250. Laing RE, Hess P, Shen Y, Wang J, Hu SX. The role and impact of SNPs in pharmacogenomics and personalized medicine. Curr Drug Metab. 2011 Jun 1;12(5):460-86. **NO ACCESS**
251. Lammers T, Aime S, Hennink WE, Storm G, Kiessling F. Theranostic Nanomedicines. Acc Chem Res. 2011 May 5. **NO ACCESS**
252. Langanke M, Brothers KB, Erdmann P, Weinert J, Krafczyk-Korth J, Dörr M, Hoffmann W, Kroemer HK, Assel H. Comparing different scientific approaches to personalized medicine: research ethics and privacy protection. Per Med. 2011 Jul;8(4):437-444.
253. Laudien M, Häsler R, Wohlers J, Böck J, Lipinski S, Bremer L, Podschun R, Ambrosch P, Lamprecht P, Rosenstiel P, Till A. Molecular signatures of a disturbed nasal barrier function in the primary tissue of Wegener's granulomatosis. Mucosal Immunol. 2011 Mar 16. **NO ACCESS**
254. Lawson HA, Cady JE, Partridge C, Wolf JB, Semenkovich CF, Cheverud JM. Genetic effects at pleiotropic loci are context-dependent with consequences for the maintenance of genetic variation in populations. PLoS Genet. 2011 Sep;7(9):e1002256.
255. Lazary J, Juhasz G, Hunyady L, Bagdy G. Personalized medicine can pave the way for the safe use of CB₁ receptor antagonists. Trends Pharmacol Sci. 2011 May;32(5):270-80.
256. Lee J, Seo JW, Jun HJ, Ki CS, Park SH, Park YS, Lim HY, Choi MG, Bae JM, Sohn TS, Noh JH, Kim S, Jang HL, Kim JY, Kim KM, Kang WK, Park JO. Impact of MET amplification on gastric cancer: possible roles as a novel prognostic marker and a potential therapeutic target. Oncol Rep. 2011 Jun;25(6):1517-24. doi: 10.3892/or.2011.1219.
257. Lee JS, Kim JH, Park YY, Mills GB. Systems biology approaches to decoding the genome of liver cancer. Cancer Res Treat. 2011 Dec;43(4):205-11.
258. Lee LA. How will personalized medicine impact the practice of gastroenterology? Clin Gastroenterol Hepatol. 2011 Apr;9(4):305-7.
259. Legrain P, Aebersold R, Archakov A, Bairoch A, Bala K, Beretta L, Bergeron J, Borchers CH, Corthals GL, Costello CE, Deutsch EW, Domon B, Hancock W, He F, Hochstrasser D, Marko-Varga G, Salekdeh GH, Sechi S, Snyder M, Srivastava S, Uhlén M, Wu CH, Yamamoto T, Paik YK, Omenn GS. The human proteome project: current state and future direction. Mol Cell Proteomics. 2011 Jul;10(7):M111.009993. **NO ACCESS**
260. Legrain P, Aebersold R, Archakov A, Bairoch A, Bala K, Beretta L, Bergeron J, Borchers C, Corthals GL, Costello CE, Deutsch EW, Domon B, Hancock W, He F, Hochstrasser D, Marko-Varga G, Salekdeh GH, Sechi S, Snyder M, Srivastava S, Uhlen M, Hu CH, Yamamoto T, Paik YK, Omenn GS. The human proteome project: Current state and future direction. Mol Cell Proteomics. 2011 Apr 29.
261. Li C. Personalized medicine - the promised land: are we there yet? Clin Genet. 2011 May;79(5):403-12. doi: 10.1111/j.1399-0004.2010.01609.x.
262. Li J, Su Z, Ma ZQ, Slebos RJ, Halvey P, Tabb DL, Liebler DC, Pao W, Zhang B. A bioinformatics workflow for variant peptide detection in shotgun proteomics. Mol Cell Proteomics. 2011 May;10(5):M110.006536. **NO ACCESS**
263. Li SD, Tagami T, Ho YF, Yeang CH. Deciphering causal and statistical relations of molecular aberrations and gene expressions in NCI-60 cell lines. BMC Syst Biol. 2011 Nov 4;5:186.
264. Li-Wan-Po A, Farndon P. Barking up the wrong genome--we are not alone. J Clin Pharm Ther. 2011 Apr;36(2):125-7. doi: 10.1111/j.1365-2710.2010.01240.x.
265. Liang SY, Phillips KA, Wang G, Keohane C, Armstrong J, Morris WM, Haas JS. Tradeoffs of using administrative claims and medical records to identify the use of personalized medicine for patients with breast cancer. Med Care. 2011 Jun;49(6):e1-8. **NO ACCESS**
266. Lillie EO, Patay B, Diamant J, Issell B, Topol EJ, Schork NJ. The n-of-1 clinical trial: the ultimate strategy for individualizing medicine? Per Med. 2011 Mar;8(2):161-173.
267. Lim MD, Dickherber A, Compton CC. Before you analyze a human specimen, think quality, variability, and bias. Anal Chem. 2011 Jan 1;83(1):8-13. Review. **NO ACCESS**
268. Linardou H, Briasoulis E, Dahabreh IJ, Mountzios G, Papadimitriou C, Papadopoulos S, Bafaloukos D, Kosmidis P, Murray S. All about KRAS for clinical oncology practice: gene profile, clinical implications and laboratory recommendations for somatic mutational testing in colorectal cancer. Cancer Treat Rev. 2011 May;37(3):221-33. Review. **NO ACCESS**
269. Linta L, Stockmann MC, Kleinhans KN, Boeckers A, Storch A, Zaehres H, Lin Q, Barbi G, Boeckers TM, Kleger A, Liebau S. Rat embryonic fibroblasts improve reprogramming of human keratinocytes into induced pluripotent stem cells. Stem Cells Dev. 2011 Jun 23. **NO ACCESS**
270. Lisanti MP, Martinez-Outschoorn UE, Pavlides S, Whitaker-Menezes D, Pestell RG, Howell A, Sotgia F. Accelerated aging in the tumor microenvironment: Connecting aging, inflammation and cancer metabolism with personalized medicine. Cell Cycle. 2011 Jul 1;10(13). **NO ACCESS**
271. Liu G, Cheng Y, Zhao W, Jin Z, Shan H, Xu G. Single-base extension and ELISA-based approach for single-nucleotide polymorphisms genotyping. Appl Biochem Biotechnol. 2011 Mar;163(5):573-6. **NO ACCESS**
272. Liu KW, Hu B, Cheng SY. Platelet-derived growth factor receptor alpha in glioma: a bad seed. Chin J Cancer. 2011 Sep;30(9):590-602.
273. Loeb LA. Human cancers express mutator phenotypes: origin, consequences and targeting. Nat Rev Cancer. 2011 Jun;11(6):450-7. **NO ACCESS**
274. Lopes P, Dalgleish R, Oliveira JL. WAVe: web analysis of the variome. Hum Mutat. 2011 Jul;32(7):729-34. doi: 10.1002/humu.21499.
275. Loscalzo J. Systems biology and personalized medicine: a network approach to human disease. Proc Am Thorac Soc. 2011 May;8(2):196-8. **NO ACCESS**.
276. Lotan TL, Gurel B, Sutcliffe S, Esopi D, Liu W, Xu J, Hicks JL, Park BH, Humphreys E, Partin AW, Han M, Netto GJ, Isaacs WB, De Marzo AM. PTEN protein loss by immunostaining: analytic validation and prognostic indicator for a high risk surgical cohort of prostate cancer patients. Clin Cancer Res. 2011 Oct 15;17(20):6563-73.
277. Lowery A, Han Z. Assessment of tumor response to tyrosine kinase inhibitors. Front Biosci. 2011 Jun 1;17:1996-2007. **NO ACCESS**
278. Loyd JE. Pulmonary arterial hypertension: insights from genetic studies. Proc Am Thorac Soc. 2011 May;8(2):154-7. **NO ACCESS**
279. Luciano JS, Andersson B, Batchelor C, Bodenreider O, Clark T, Denney CK, Domarew C, Gambet T, Harland L, Jentzsch A, Kashyap V, Kos P, Kozlovsky J, Lebo T, Marshall SM, McCusker JP, McGuinness DL, Ogbuji C, Pichler E, Powers RL, Prud'hommeaux E, Samwald M, Schriml L, Tonellato PJ, Whetzel PL, Zhao J, Stephens S, Dumontier M. The Translational Medicine Ontology and Knowledge Base: driving personalized medicine by bridging the gap between bench and bedside. J Biomed Semantics. 2011 May 17;2 Suppl 2:S1.
280. Lytkin NI, McVoy L, Weitkamp JH, Aliferis CF, Statnikov A. Expanding the understanding of biases in development of clinical-grade molecular signatures: a case study in acute respiratory viral infections. PLoS One. 2011;6(6):e20662.
281. Mínguez B, Lachenmayer A. Diagnostic and prognostic molecular markers in hepatocellular carcinoma. Dis Markers. 2011;31(3):181-90. Review. **NO ACCESS**
282. Ma Q, Lu AY. Pharmacogenetics, pharmacogenomics, and individualized medicine. Pharmacol Rev. 2011 Jun;63(2):437-59. doi: 10.1124/pr.110.003533.
283. Ma ZS, Abdo Z, Forney LJ. Caring about trees in the forest: incorporating frailty in risk analysis for personalized medicine. Per Med. 2011 Nov;8(6):681-688. **NO ACCESS**
284. Maász A, Szolnoki Z, Balikó L, Melegh B. [Genetics of ischemic stroke: where are we now?]. Orv Hetil. 2011 Mar 20;152(12):455-63. Review. Hungarian.
285. Macklis RM, Sharma N. Convergence technology in cancer medicine. Expert Rev Med Devices. 2011 Mar;8(2):263-73. Review. **NO ACCESS**
286. Macpherson JL, Rasko JE. Cellular therapy in the Asia-Pacific region. A guide for the future pathologist. Pathology. 2011 Oct;43(6):616-26. Review. **NO ACCESS**
287. Maiti S, Kumar KH, Castellani CA, O'Reilly R, Singh SM. Ontogenetic de novo copy number variations (CNVs) as a source of genetic individuality: studies on two families with MZD twins for schizophrenia. PLoS One. 2011 Mar 2;6(3):e17125.
288. Majewski J, Schwartzentruber J, Lalonde E, Montpetit A, Jabado N. What can exome sequencing do for you? J Med Genet. 2011 Sep;48(9):580-9.
289. Makowsky R, Pajewski NM, Klimentidis YC, Vazquez AI, Duarte CW, Allison DB, de los Campos G. Beyond missing heritability: prediction of complex traits. PLoS Genet. 2011 Apr;7(4):e1002051.
290. Malagelada JR. Diseases of the digestive tract: is prevention possible and feasible? Dig Dis. 2011;29(2):255-63.
291. Malandrino N, Smith RJ. Personalized medicine in diabetes. Clin Chem. 2011 Feb;57(2):231-40. **NO ACCESS**
292. Mandrekar SJ, Sargent DJ. Design of clinical trials for biomarker research in oncology. Clin Investig (Lond). 2011 Dec;1(12):1629-1636. **NO ACCESS**
293. Manolopoulos VG, Dechairo B, Huriez A, Kühn A, Llerena A, van Schaik RH, Yeo KT, Ragia G, Siest G. Pharmacogenomics and personalized medicine in clinical practice. Pharmacogenomics. 2011 May;12(5):597-610. **NO ACCESS**
294. Manolopoulos VG, Ragia G, Tavridou A. Pharmacogenomics of oral antidiabetic medications: current data and pharmacoepigenomic perspective. Pharmacogenomics. 2011 Aug;12(8):1161-91. Review.
295. March JS. Looking to the future of research in pediatric anxiety disorders. Depress Anxiety. 2011 Jan;28(1):88-98.
296. Marken PA. Personalized medicine: are we preparing our students for the knowledge revolution? Am J Pharm Educ. 2011 Apr 11;75(3):48.
297. Marko-Varga G, Fehniger TE, Rezeli M, Döme B, Laurell T, Végvári A. Drug localization in different lung cancer phenotypes by MALDI mass spectrometry imaging. J Proteomics. 2011 Jun 10;74(7):982-92.
298. Marko-Varga G. BioBanking - The Holy Grail of novel drug and diagnostic developments? J Clin Bioinforma. 2011 May 13;1(1):14.
299. Markowitz SM, Park ER, Delahanty LM, O'Brien KE, Grant RW. Perceived impact of diabetes genetic risk testing among patients at high phenotypic risk for type 2 diabetes. Diabetes Care. 2011 Mar;34(3):568-73. **NO ACCESS**
300. Marquardt JU, Galle PR, Teufel A. Molecular diagnosis and therapy of hepatocellular carcinoma (HCC); An emerging field for advanced technologies. J Hepatol. 2011 Jul 20. **NO ACCESS**
301. Martinez-Outschoorn UE, Prisco M, Ertel A, Tsirigos A, Lin Z, Pavlides S, Wang C, Flomenberg N, Knudsen ES, Howell A, Pestell RG, Sotgia F, Lisanti MP. Ketones and lactate increase cancer cell "stemness," driving recurrence, metastasis and poor clinical outcome in breast cancer: achieving personalized medicine via Metabolo-Genomics. Cell Cycle. 2011 Apr 15;10(8):1271-86.
302. Martinez-Outschoorn UE, Prisco M, Ertel A, Tsirigos A, Lin Z, Pavlides S, Wang C, Flomenberg N, Knudsen ES, Howell A, Pestell RG, Sotgia F, Lisanti MP. Ketones and lactate increase cancer cell "stemness," driving recurrence, metastasis, and poor clinical outcome in breast cancer: Achieving personalized medicine via Metabolo-Genomics. Cell Cycle. 2011 Apr 15;10(8).
303. Mayanil T, Wegbreit E, Fitzgerald J, Pavuluri M. Emerging biosignature of brain function and intervention in pediatric bipolar disorder. Minerva Pediatr. 2011 Jun;63(3):183-200.
304. McCabe LL, McCabe ER. Personalized medicine for individuals with Down syndrome. Mol Genet Metab. 2011 Sep-Oct;104(1-2):7-9.
305. McCarty CA, Garber A, Reeser JC, Fost NC; Personalized Medicine Research Project Community Advisory Group and Ethics and Security Advisory Board. Study newsletters, community and ethics advisory boards, and focus group discussions provide ongoing feedback for a large biobank. Am J Med Genet A. 2011 Apr;155A(4):737-41. doi: 10.1002/ajmg.a.33896.
306. McDonald SA, Watson MA, Rossi J, Becker CM, Jaques DP, Pfeifer JD. A new paradigm for biospecimen banking in the personalized medicine era. Am J Clin Pathol. 2011 Nov;136(5):679-84.
307. McGhee SA. How the practice of allergy shows the promise and challenge of personalized medicine. Mol Genet Metab. 2011 Sep-Oct;104(1-2):3-6.
308. McKinnon R, Anderson C. Transforming pharmaceutical education to accelerate the acceptance and implementation of personalized medicine. Am J Pharm Educ. 2011 Aug 10;75(6):107. **NO ACCESS**
309. McLaughlin R, Hylton N. MRI in breast cancer therapy monitoring. NMR Biomed. 2011 Jun 21. doi: 10.1002/nbm.1739.
310. McNiven EM, German JB, Slupsky CM. Analytical metabolomics: nutritional opportunities for personalized health. J Nutr Biochem. 2011 Nov;22(11):995-1002. Review. **NO ACCESS**
311. Meany DL, Chan DW. Aberrant glycosylation associated with enzymes as cancer biomarkers. Clin Proteomics. 2011 Jun 3;8(1):7.
312. Mehta R, Jain RK, Badve S. Personalized medicine: the road ahead. Clin Breast Cancer. 2011 Mar;11(1):20-6. Review. **NO ACCESS**
313. Melichar B, Plebani M. Targeted therapy for HER-2: personalized medicine for her, too. Clin Chem Lab Med. 2011 Sep 29. **NO ACCESS**
314. Mesko B, Poliska S, Nagy L. Gene expression profiles in peripheral blood for the diagnosis of autoimmune diseases. Trends Mol Med. 2011 Apr;17(4):223-33.
315. Meslin EM, Garba I. Biobanking and public health: is a human rights approach the tie that binds? Hum Genet. 2011 Jul 15. **NO ACCESS**
316. Mestroni L, Merlo M, Taylor MR, Camerini F, Sinagra G. Heart failure and personalized medicine. J Cardiovasc Med (Hagerstown). 2011 Jan;12(1):6-12. Review.
317. Mestroni L, Taylor MR. Pharmacogenomics, personalized medicine, and heart failure. Discov Med. 2011 Jun;11(61):551-61. **NO ACCESS**
318. Metodiev MV. Biomarkers research in Europe: focus on personalized medicine. Expert Rev Mol Diagn. 2011 Sep;11(7):689-90.
319. Meyer J, Ostrzinski S, Fredrich D, Havemann C, Krafczyk J, Hoffmann W. Efficient data management in a large-scale epidemiology research project. Comput Methods Programs Biomed. 2011 Jan 20. **NO ACCESS**
320. Meyer JS, Howden SE, Wallace KA, Verhoeven AD, Wright LS, Capowski EE, Pinilla I, Martin JM, Tian S, Stewart R, Pattnaik B, Thomson J, Gamm DM. Optic Vesicle-like Structures Derived from Human Pluripotent Stem Cells Facilitate a Customized Approach to Retinal Disease Treatment. Stem Cells. 2011 Jun 15. doi: 10.1002/stem.674. **NO ACCESS**
321. Milane L, Duan Z, Amiji M. Therapeutic efficacy and safety of paclitaxel/lonidamine loaded EGFR-targeted nanoparticles for the treatment of multi-drug resistant cancer. PLoS One. 2011;6(9):e24075.
322. Milleron B. [Editorial. NSCLC and personalized medicine: yesterday, today and tomorrow]. Rev Pneumol Clin. 2011 Jun;67 Suppl 1:S1-2. French.
323. Miossec P, Verweij CL, Klareskog L, Pitzalis C, Barton A, Lekkerkerker F, Reiter S, Laslop A, Breedveld F, Abadie E, Flamion B, Dere W, Mpofu S, Goel N, Ethgen D, Mitlak B, Ormarsdóttir S, Rao R, Tsouderos Y, Reginster JY; Group for Respect of Ethics and Excellence in Science (GREES). Biomarkers and personalised medicine in rheumatoid arthritis: a proposal for interactions between academia, industry and regulatory bodies. Ann Rheum Dis. 2011 Oct;70(10):1713-8. Review.
324. Mishra L. Health care reform: how personalized medicine could help bundling of care for liver diseases. Hepatology. 2011 Feb;53(2):379-81. doi: 10.1002/hep.24144.
325. Mitsiades CS, Davies FE, Laubach JP, Joshua D, San Miguel J, Anderson KC, Richardson PG. Future directions of next-generation novel therapies, combination approaches, and the development of personalized medicine in myeloma. J Clin Oncol. 2011 May 10;29(14):1916-23. **NO ACCESS**
326. Miyachi H. [Companion diagnostics: significances and issues in its clinical application]. Rinsho Byori. 2011 Jun;59(6):602-9. Review. Japanese.
327. Modi S, Krahn A, Yee R. Current concepts in pacing 2010-2011: the right and wrong way to pace. Curr Treat Options Cardiovasc Med. 2011 Oct;13(5):370-84. **NO ACCESS**
328. Mok TS. Personalized medicine in lung cancer: what we need to know. Nat Rev Clin Oncol. 2011 Aug 23;8(11):661-8. doi: 10.1038/nrclinonc.2011.126. Review.
329. Mollberg N, Surati M, Demchuk C, Fathi R, Salama AK, Husain AN, Hensing T, Salgia R. Mind-mapping for lung cancer: towards a personalized therapeutics approach. Adv Ther. 2011 Mar;28(3):173-94.
330. Morrison KE. Whole-genome sequencing informs treatment: personalized medicine takes another step forward. Clin Chem. 2011 Dec;57(12):1638-40.
331. Msall ME. Personalized medicine and the importance of measuring functioning and participation over time: lessons from achondroplasia. Dev Med Child Neurol. 2011 Oct;53(10):882-3. doi: 10.1111/j.1469-8749.2011.04087.x.
332. Mueller C, Edmiston KH, Carpenter C, Gaffney E, Ryan C, Ward R, White S, Memeo L, Colarossi C, Petricoin EF 3rd, Liotta LA, Espina V. One-step preservation of phosphoproteins and tissue morphology at room temperature for diagnostic and research specimens. PLoS One. 2011;6(8):e23780.
333. Mukhopadhyay A, Curtin N, Plummer R, Edmondson RJ. PARP inhibitors and epithelial ovarian cancer: an approach to targeted chemotherapy and personalised medicine. BJOG. 2011 Mar;118(4):429-32. doi: 10.1111/j.1471-0528.2010.02838.x. Epub 2011 Jan 18. Review.
334. Nagrath D, Caneba C, Karedath T, Bellance N. Metabolomics for mitochondrial and cancer studies. Biochim Biophys Acta. 2011 Jun;1807(6):650-63. **NO ACCESS**
335. Naik S. Towards personalized medicine - the role of methotrexate. Indian J Med Res. 2011 Mar;133(3):253-5.
336. Nakajima M, Yokoi T. MicroRNAs from biology to future pharmacotherapy: Regulation of cytochrome P450s and nuclear receptors. Pharmacol Ther. 2011 Sep;131(3):330-7. **NO ACCESS**
337. Naldini L. Ex vivo gene transfer and correction for cell-based therapies. Nat Rev Genet. 2011 May;12(5):301-15. **NO ACCESS**
338. Nam HY, Shim SM, Han BG, Jeon JP. Human lymphoblastoid cell lines: a goldmine for the biobankomics era. Pharmacogenomics. 2011 Jun;12(6):907-17. **NO ACCESS**
339. Napoletani D, Signore M, Sauer T, Liotta L, Petricoin E. Homologous control of protein signaling networks. J Theor Biol. 2011 Jun 21;279(1):29-43.
340. Natoli G, Capalbo C. The end of the beginning of personalized medicine. Ann Oncol. 2011 Dec;22(12):2695.
341. Nejak-Bowen KN, Monga SP. Beta-catenin signaling, liver regeneration and hepatocellular cancer: sorting the good from the bad. Semin Cancer Biol. 2011 Feb;21(1):44-58. Review.
342. Nelson DR, Conlon M, Baralt C, Johnson JA, Clare-Salzler MJ, Rawley-Payne M. University of Florida Clinical and Translational Science Institute: transformation and translation in personalized medicine. Clin Transl Sci. 2011 Dec;4(6):400-2. doi: 10.1111/j.1752-8062.2011.00348.x. **NO ACCESS**
343. Nersting J, Borst L, Schmiegelow K. Challenges in implementing individualized medicine illustrated by antimetabolite therapy of childhood acute lymphoblastic leukemia. Clin Proteomics. 2011 Jun 3;8(1):8.
344. Nestorova GG, Guilbeau EJ. Thermoelectric method for sequencing DNA. Lab Chip. 2011 May 21;11(10):1761-9.
345. Nevedomskaya E, Mayboroda OA, Deelder AM. Cross-platform analysis of longitudinal data in metabolomics. Mol Biosyst. 2011 Dec;7(12):3214-22.
346. Nguyen DX. Tracing the origins of metastasis. J Pathol. 2011 Jan;223(2):195-204. doi: 10.1002/path.2801
347. Nicholson JK, Wilson ID, Lindon JC. Pharmacometabonomics as an effector for personalized medicine. Pharmacogenomics. 2011 Jan;12(1):103-11. Review. **NO ACCESS**
348. Nilsson RJ, Balaj L, Hulleman E, van Rijn S, Pegtel DM, Walraven M, Widmark A, Gerritsen WR, Verheul HM, Vandertop WP, Noske DP, Skog J, Würdinger T. Blood platelets contain tumor-derived RNA biomarkers. Blood. 2011 Sep 29;118(13):3680-3.
349. Nolan GP. Flow cytometry in the post fluorescence era. Best Pract Res Clin Haematol. 2011 Dec;24(4):505-8. doi: 10.1016/j.beha.2011.09.005. Review.
350. Novembre J, Ramachandran S. Perspectives on human population structure at the cusp of the sequencing era. Annu Rev Genomics Hum Genet. 2011 Sep 22;12:245-74. Review.
351. O'Connor JP, Rose CJ, Jackson A, Watson Y, Cheung S, Maders F, Whitcher BJ, Roberts C, Buonaccorsi GA, Thompson G, Clamp AR, Jayson GC, Parker GJ. DCE-MRI biomarkers of tumour heterogeneity predict CRC liver metastasis shrinkage following bevacizumab and FOLFOX-6. Br J Cancer. 2011 Jun 28;105(1):139-45. doi: 10.1038/bjc.2011.191.
352. Oberpichler-Schwenk H. ["Personalized medicine" between genome and person]. Med Monatsschr Pharm. 2011 Dec;34(12):443. German.
353. Odierna DH, Afable-Munsuz A, Ikediobi O, Beattie M, Knight S, Ko M, Wilson A, Ponce NA. Early developments in gene-expression profiling of breast tumors: potential for increasing black-white patient disparities in breast cancer outcomes? Per Med. 2011 Nov;8(6):669-679. **NO ACCESS**
354. Offit K. Personalized medicine: new genomics, old lessons. Hum Genet. 2011 Jul;130(1):3-14.
355. Ogino S, Galon J, Fuchs CS, Dranoff G. Cancer immunology--analysis of host and tumor factors for personalized medicine. Nat Rev Clin Oncol. 2011 Aug 9;8(12):711-9. doi: 10.1038/nrclinonc.2011.122. Review.
356. Ogita S, Lorusso P. Targeting phosphatidylinositol 3 kinase (PI3K)-Akt beyond rapalogs. Target Oncol. 2011 Jun;6(2):103-17. **NO ACCESS**
357. Opal SM. The evolution of the understanding of sepsis, infection, and the host response: a brief history. Crit Care Nurs Clin North Am. 2011 Mar;23(1):1-27. **NO ACCESS**
358. Orlando LA, Hauser ER, Christianson C, Powell KP, Buchanan AH, Chesnut B, Agbaje AB, Henrich VC, Ginsburg G. Protocol for implementation of family health history collection and decision support into primary care using a computerized family health history system. BMC Health Serv Res. 2011 Oct 11;11:264.
359. Osaki A, Saeki T. [Current status and future direction of postoperative adjuvant therapy in breast cancer]. Gan To Kagaku Ryoho. 2011 Sep;38(9):1406-11. Japanese.
360. Ozdemir V, Muljono DH, Pang T, Ferguson LR, Manamperi A, Samper S, Someya T, Tassé AM, Tsai SJ, Zhou HH, Lee EJ. Asia-Pacific Health 2020 and Genomics without Borders: Co-Production of Knowledge by Science and Society Partnership for Global Personalized Medicine. Curr Pharmacogenomics Person Med. 2011 Mar 1;9(1):1-5.
361. Pagel M, Chopra A. Ytterbium chelated to 1,4,7,10-tetraazacyclododecane-1,4,7-triacetic acid,10-orthoaminoanilide. 2011 Nov 26 [updated 2012 Jan 05]. Molecular Imaging and Contrast Agent Database (MICAD) [Internet]. Bethesda (MD): National Center for Biotechnology Information (US); 2004-2011. Available from http://www.ncbi.nlm.nih.gov/books/NBK83154/
362. Pajic M, Scarlett CJ, Chang DK, Sutherland RL, Biankin AV. Preclinical strategies to define predictive biomarkers for therapeutically relevant cancer subtypes. Hum Genet. 2011 Jul;130(1):93-101.
363. Panicco P, Dodhia VR, Fantuzzi A, Gilardi G. Enzyme-based amperometric platform to determine the polymorphic response in drug metabolism by cytochromes P450. Anal Chem. 2011 Mar 15;83(6):2179-86. **NO ACCESS**
364. Park KS. The search for genetic risk factors of type 2 diabetes mellitus. Diabetes Metab J. 2011 Feb;35(1):12-22. doi: 10.4093/dmj.2011.35.1.12.
365. Pasche B, Absher D. Whole-genome sequencing: a step closer to personalized medicine. JAMA. 2011 Apr 20;305(15):1596-7. **NO ACCESS**
366. Patel RS, Ye S. Genetic determinants of coronary heart disease: new discoveries and insights from genome-wide association studies. Heart. 2011 Sep;97(18):1463-73. Epub 2011 Jul 26. Review.
367. Patel SA, Rameshwar P. Stem Cell Transplantation for Hematological Malignancies: Prospects for Personalized Medicine and Co-therapy with Mesenchymal Stem Cells. Curr Pharmacogenomics Person Med. 2011 Sep 1;9(3):229-239.
368. Pearson JF, Brownstein CA, Brownstein JS. Potential for electronic health records and online social networking to redefine medical research. Clin Chem. 2011 Feb;57(2):196-204.
369. Perez RO. Predicting response to neoadjuvant treatment for rectal cancer: a step toward individualized medicine. Dis Colon Rectum. 2011 Sep;54(9):1057-8. **NO ACCESS**
370. Pichler WJ, Naisbitt DJ, Park BK. Immune pathomechanism of drug hypersensitivity reactions. J Allergy Clin Immunol. 2011 Mar;127(3 Suppl):S74-81.
371. Pierceall WE, Sprott KM, Heikkinen T, Heikkila P, Alaparthi L, Aittomaki K, Al-Adhami M, Villegas-Bergazzi V, Meyer JL, Kutok JL, Bartkova J, Bartek J, Nevanlinna H, Weaver DT, Blomqvist C. Utilization of fluorescence in situ hybridization with cytokeratin discriminators in TOP2A assessment of chemotherapy-treated patients with breast cancer. Hum Pathol. 2011 Dec 26.
372. Pipkorn R, Wiessler M, Waldeck W, Lorenz P, Muehlhausen U, Fleischhacker H, Koch M, Braun K. Enhancement of the click chemistry for the inverse diels alder technology by functionalization of amide-based monomers. Int J Med Sci. 2011;8(5):387-96.
373. Plenge RM, Bridges SL Jr. Personalized medicine in rheumatoid arthritis: miles to go before we sleep. Arthritis Rheum. 2011 Mar;63(3):590-3. doi: 10.1002/art.30126.
374. Plouffe BD, Lewis LH, Murthy SK. Computational design optimization for microfluidic magnetophoresis. Biomicrofluidics. 2011 Mar 30;5:13413.
375. Poh N, de Lusignan S. Data-modelling and visualisation in chronic kidney disease (CKD): a step towards personalised medicine. Inform Prim Care. 2011;19(2):57-63.
376. Poland GA, Ovsyannikova IG, Kennedy RB, Haralambieva IH, Jacobson RM. Vaccinomics and a new paradigm for the development of preventive vaccines against viral infections. OMICS. 2011 Sep;15(9):625-36. Epub 2011 Jul 6. Review.
377. Poo DC, Cai S, Mah JT. UASIS: Universal Automatic SNP Identification System. BMC Genomics. 2011 Nov 30;12 Suppl 3:S9.
378. Porcelli S, Drago A, Fabbri C, Gibiino S, Calati R, Serretti A. Pharmacogenetics of antidepressant response. J Psychiatry Neurosci. 2011 Mar;36(2):87-113. Review.
379. Porcelli S, Fabbri C, Spina E, Serretti A, De Ronchi D. Genetic polymorphisms of cytochrome P450 enzymes and antidepressant metabolism. Expert Opin Drug Metab Toxicol. 2011 Sep;7(9):1101-15. Epub 2011 Jul 8. Review. **NO ACCESS**
380. Postma MJ, Boersma C, Vandijck D, Vegter S, Le HH, Annemans L. Health technology assessments in personalized medicine: illustrations for cost-effectiveness analysis. Expert Rev Pharmacoecon Outcomes Res. 2011 Aug;11(4):367-9.
381. Potosky AL, Malin JL, Kim B, Chrischilles EA, Weeks JC. Re: personalized medicine and cancer supportive care: appropriate use of colony-stimulating factor support of chemotherapy. J Natl Cancer Inst. 2011 Dec 21;103(24):1899-901; author reply 1901-3
382. Pravettoni G, Gorini A. A P5 cancer medicine approach: why personalized medicine cannot ignore psychology. J Eval Clin Pract. 2011 Jun 16. doi: 10.1111/j.1365-2753.2011.01709.x. **NO ACCESS**
383. Price MJ, Barker CM. Functional testing methods for the antiplatelet effect of P2Y12 receptor antagonists. Biomark Med. 2011 Feb;5(1):43-51. Review. **NO ACCESS**
384. Printz C. Lung cancer a hot topic at AACR meeting: Studies offer promise in detection, prevention, and personalized medicine. Cancer. 2011 Jun 15;117(12):2583-5. doi: 10.1002/cncr.26258.
385. Priori SG. Induced pluripotent stem cell-derived cardiomyocytes and long QT syndrome: is personalized medicine ready for prime time? Circ Res. 2011 Sep 30;109(8):822-4. doi: 10.1161/CIRCRESAHA.111.253724.
386. Pritchard CC, Grady WM. Colorectal cancer molecular biology moves into clinical practice. Gut. 2011 Jan;60(1):116-29. Epub 2010 Oct 4. Review.
387. Průcha M, Matejovic M, Sedlácková L. [Diagnostics of sepsis]. Cas Lek Cesk. 2011;150(4-5):283-8. Review. Czech.
388. Pyeritz RE. The family history: The first genetic test, and still useful after all those years? Genet Med. 2011 Oct 7. **NO ACCESS**
389. Rahme RJ, Batjer HH, Bendok BR. Guidelines or individualized medicine: can they be reconciled? Neurosurgery. 2011 Oct;69(4):N19-20.
390. Rakowska PD, Ryadnov MG. Nano-enabled biomarker discovery and detection. Biomark Med. 2011 Jun;5(3):387-96. **NO ACCESS**
391. Reddy PJ, Jain R, Paik YK, Downey R, Ptolemy AS, Ozdemir V, Srivastava S. Personalized Medicine in the Age of Pharmacoproteomics: A Close up on India and Need for Social Science Engagement for Responsible Innovation in Post-Proteomic Biology. Curr Pharmacogenomics Person Med. 2011 Mar 1;9(1):67-75.
392. Reed D, Altiok S. Metastatic soft tissue sarcoma chemotherapy: an opportunity for personalized medicine. Cancer Control. 2011 Jul;18(3):188-95.
393. Reeser JC, Payne E, Kitchner T, McCarty CA. Apolipoprotein e4 genotype increases the risk of being diagnosed with posttraumatic fibromyalgia. PM R. 2011 Mar;3(3):193-7.
394. Reynolds F, Panneer N, Tutino CM, Wu M, Skrabal WR, Moskaluk C, Kelly KA. A functional proteomic method for biomarker discovery. PLoS One. 2011;6(7):e22471.
395. Rezeli M, Végvári A, Fehniger TE, Laurell T, Marko-Varga G. Moving towards high density clinical signature studies with a human proteome catalogue developing multiplexing mass spectrometry assay panels. J Clin Bioinforma. 2011 Feb 8;1(1):7.
396. Ricart AD. Antibody-drug conjugates of calicheamicin derivative: gemtuzumab ozogamicin and inotuzumab ozogamicin. Clin Cancer Res. 2011 Oct 15;17(20):6417-27. Review.
397. Rinott R, Carmeli B, Kent C, Landau D, Maman Y, Rubin Y, Slonim N. Prognostic data-driven clinical decision support - formulation and implications. Stud Health Technol Inform. 2011;169:140-4. **NO ACCESS**
398. Roach JC, Glusman G, Hubley R, Montsaroff SZ, Holloway AK, Mauldin DE, Srivastava D, Garg V, Pollard KS, Galas DJ, Hood L, Smit AF. Chromosomal haplotypes by genetic phasing of human families. Am J Hum Genet. 2011 Sep 9;89(3):382-97. doi: 10.1016/j.ajhg.2011.07.023.
399. Roach M 3rd, Alberini JL, Pecking AP, Testori A, Verrecchia F, Soteldo J, Ganswindt U, Joyal JL, Babich JW, Witte RS, Unger E, Gottlieb R. Diagnostic and therapeutic imaging for cancer: therapeutic considerations and future directions. J Surg Oncol. 2011 May 1;103(6):587-601. doi: 10.1002/jso.21805. Review.
400. Roden DM. Personalized medicine and the genotype-phenotype dilemma. J Interv Card Electrophysiol. 2011 Jun;31(1):17-23.
401. Rogers JC, Taylor AT. Teaching about genetic testing issues in the undergraduate classroom: a case study. J Genet Couns. 2011 Jun;20(3):231-40. doi: 10.1007/s10897-011-9352-2.
402. Romer T, Leonhardt H, Rothbauer U. Engineering antibodies and proteins for molecular in vivo imaging. Curr Opin Biotechnol. 2011 Jun 25. **NO ACCESS**
403. Romero-Morelos P, Peralta-Rodríguez R, Mendoza-Rodríguez M, Valdivia-Flores A, Marrero-Rodríguez D, Paniagua-García L, Rodríguez-Cabrales J, Parra-Melquiádez M, Salcedo-Vargas M. [The nanotechnology as a support for diagnosis and prognosis in cancer research]. Rev Med Inst Mex Seguro Soc. 2011 Nov-Dec;49(6):621-30. Spanish.
404. Rosenbach AE, Koria P, Goverman J, Kotz KT, Gupta A, Yu M, Fagan SP, Irimia D, Tompkins RG. Microfluidics for T- lymphocyte cell separation and inflammation monitoring in burn patients. Clin Transl Sci. 2011 Feb;4(1):63-8. doi: 10.1111/j.1752-8062.2010.00255.x.
405. Rossi S, Christ-Neumann M, Rüping S, Buffa F, Wegener D, McVie G, Coveney P, Graf N, Delorenzi M. p-Medicine: From data sharing and integration via VPH models to personalized medicine. Ecancermedicalscience. 2011;5:218.
406. Roukos DH. Trastuzumab and beyond: sequencing cancer genomes and predicting molecular networks. Pharmacogenomics J. 2011 Apr;11(2):81-92. Review. **NO ACCESS**
407. Rowland M, Peck C, Tucker G. Physiologically-based pharmacokinetics in drug development and regulatory science. Annu Rev Pharmacol Toxicol. 2011 Feb 10;51:45-73. Review. **NO ACCESS**
408. Ruben RJ. Otitis media: the application of personalized medicine. Otolaryngol Head Neck Surg. 2011 Nov;145(5):707-12.
409. Rubinow DR, Girdler SS. Hormones, heart disease, and health: individualized medicine versus throwing the baby out with the bathwater. Depress Anxiety. 2011 Jun;28(6):E1-E15. doi: 10.1002/da.20833. Review.
410. Rukov JL, Shomron N. MicroRNA pharmacogenomics: Post-transcriptional regulation of drug response. Trends Mol Med. 2011 Jun 6. **NO ACCESS**
411. Russell SJ, Rajkumar SV. Multiple myeloma and the road to personalised medicine. Lancet Oncol. 2011 Jul;12(7):617-9.
412. Sériès F. Sleep apnoea syndrome: how will physiologic knowledge position personalised medicine? Eur Respir Rev. 2011 Sep 1;20(121):127-9. **NO ACCESS**
413. Sadee W. Genomics and personalized medicine. Int J Pharm. 2011 Aug 30;415(1-2):2-4.
414. Sadee W. Pharmacogenomic biomarkers: validation needed for both the molecular genetic mechanism and clinical effect. Pharmacogenomics. 2011 May;12(5):675-80. **NO ACCESS**
415. Salari K, Pizzo PA, Prober CG. Commentary: to genotype or not to genotype? Addressing the debate through the development of a genomics and personalized medicine curriculum. Acad Med. 2011 Aug;86(8):925-7.
416. Salto-Tellez M, Tsao MS, Shih JY, Thongprasert S, Lu S, Chang GC, Au JS, Chou TY, Lee JS, Shi YK, Radzi A, Kang JH, Kim SW, Tan SY, Yang JC. Clinical and testing protocols for the analysis of epidermal growth factor receptor mutations in East Asian patients with non-small cell lung cancer: a combined clinical-molecular pathological approach. J Thorac Oncol. 2011 Oct;6(10):1663-9. **NO ACCESS**
417. Schellekens RC, Stellaard F, Woerdenbag HJ, Frijlink HW, Kosterink JG. Applications of stable isotopes in clinical pharmacology. Br J Clin Pharmacol. 2011 Dec;72(6):879-97. doi: 10.1111/j.1365-2125.2011.04071.x. Review.
418. Scher HI, Nasso SF, Rubin EH, Simon R. Adaptive clinical trial designs for simultaneous testing of matched diagnostics and therapeutics. Clin Cancer Res. 2011 Nov 1;17(21):6634-40.
419. Schink JC, Copeland LJ. Point: chemosensitivity assays have a role in the management of recurrent ovarian cancer. J Natl Compr Canc Netw. 2011 Jan;9(1):115-20. **NO ACCESS**
420. Schwab M, Schaeffeler E, Zanger UM, Brauch H, Kroemer HK. [Pharmacogenomics: hype or hope?]. Dtsch Med Wochenschr. 2011 Mar;136(10):461-7. doi: 10.1055/s-0031-1274528. Epub 2011 Mar 1. Review. German.
421. Schwaiger M, Wester HJ. How many PET tracers do we need? J Nucl Med. 2011 Dec;52 Suppl 2:36S-41S.
422. Sempere LF. Integrating contextual miRNA and protein signatures for diagnostic and treatment decisions in cancer. Expert Rev Mol Diagn. 2011 Nov;11(8):813-27. Review.
423. Shacham-Shmueli E, Beny A, Geva R, Blachar A, Figer A, Aderka D. Response to temozolomide in patients with metastatic colorectal cancer with loss of MGMT expression: a new approach in the era of personalized medicine? J Clin Oncol. 2011 Apr 1;29(10):e262-5. **NO ACCESS**
424. Shah JB, McConkey DJ, Dinney CP. New strategies in muscle-invasive bladder cancer: on the road to personalized medicine. Clin Cancer Res. 2011 May 1;17(9):2608-12.
425. Shapiro SD. Merging personalized medicine and biology of aging in chronic obstructive pulmonary disease. Am J Respir Crit Care Med. 2011 Oct 15;184(8):864-6.
426. Shen LH, Tseng YC, Liao MH, Fu YK. The role of molecular imaging in the diagnosis and management of neuropsychiatric disorders. J Biomed Biotechnol. 2011;2011:439397.
427. Shi SR, Shi Y, Taylor CR. Antigen retrieval immunohistochemistry: review and future prospects in research and diagnosis over two decades. J Histochem Cytochem. 2011 Jan;59(1):13-32. Review.
428. Shimizu K, Nishimura M. [Evidence of pharmacotherapy in COPD--key findings from recently-conducted randomized clinical studies]. Nihon Rinsho. 2011 Oct;69(10):1815-20. Review. Japanese.
429. Shirts BH, Wilson AR, Jackson BR. Partitioning reference intervals by use of genetic information. Clin Chem. 2011 Mar;57(3):475-81.
430. Shuster M. Can genetics and genomics nursing competencies be successfully taught in a prenursing microbiology course? CBE Life Sci Educ. 2011 Summer;10(2):216-21.
431. Sigdel TK, Sarwal MM. Recent advances in biomarker discovery in solid organ transplant by proteomics. Expert Rev Proteomics. 2011 Dec;8(6):705-15. Review.
432. Simon RM, Freidlin B. Re: Designing a randomized clinical trial to evaluate personalized medicine: a new approach based on risk prediction. J Natl Cancer Inst. 2011 Mar 2;103(5):445; author reply 445-6, discussion 446.
433. Sinha AA. The genetics of pemphigus. Dermatol Clin. 2011 Jul;29(3):381-91, vii. **NO ACCESS**
434. Siqueira RC, Jorge R. Translational research in retinology. Clin Ophthalmol. 2011;5:1493-8. Epub 2011 Oct 14.
435. Smith GD. Epidemiology, epigenetics and the 'Gloomy Prospect': embracing randomness in population health research and practice. Int J Epidemiol. 2011 Jun;40(3):537-62.
436. Snyderman R. Personalized health care: From theory to practice. Biotechnol J. 2011 Dec 16. doi: 10.1002/biot.201100297. **NO ACCESS**
437. Soh TI, Yong WP, Innocenti F. Recent progress and clinical importance on pharmacogenetics in cancer therapy. Clin Chem Lab Med. 2011 Oct;49(10):1621-32. Epub 2011 Sep 28. Review.
438. Song Q, Stadler LK, Peng J, Ko Ferrigno P. Peptide aptamer microarrays: bridging the bio-detector interface. Faraday Discuss. 2011;149:79-92; discussion 137-57. **NO ACCESS**
439. Sonntag J, Mannsperger H, Jöcker A, Korf U. Microspot immunoassay-based analysis of plasma protein profiles for biomarker discovery strategies. Methods Mol Biol. 2011;785:237-45. **NO ACCESS**
440. Sottas PE, Robinson N, Rabin O, Saugy M. The athlete biological passport. Clin Chem. 2011 Jul;57(7):969-76.
441. Spencer KL, Olson LM, Schnetz-Boutaud N, Gallins P, Agarwal A, Iannaccone A, Kritchevsky SB, Garcia M, Nalls MA, Newman AB, Scott WK, Pericak-Vance MA, Haines JL. Using genetic variation and environmental risk factor data to identify individuals at high risk for age-related macular degeneration. PLoS One. 2011 Mar 24;6(3):e17784.
442. Springer JA, Iannotti NV, Kane MD, Haynes K, Sprague JE. Pharmacogenomics training using an instructional software system. Am J Pharm Educ. 2011 Mar 10;75(2):32.
443. Squire JA, Park PC, Yoshimoto M, Alami J, Williams JL, Evans A, Joshua AM. Prostate cancer as a model system for genetic diversity in tumors. Adv Cancer Res. 2011;112:183-216. Review. **NO ACCESS**
444. Stack CB, Gharani N, Gordon ES, Schmidlen T, Christman MF, Keller MA. Genetic risk estimation in the Coriell Personalized Medicine Collaborative. Genet Med. 2011 Feb;13(2):131-9.
445. Stamatakos GS, Georgiadi EC, Graf N, Kolokotroni EA, Dionysiou DD. Exploiting clinical trial data drastically narrows the window of possible solutions to the problem of clinical adaptation of a multiscale cancer model. PLoS One. 2011 Mar 3;6(3):e17594.
446. Steffens JA. [On course for knowledge-based and individualized medicine. 63rd Congress of the German Society for Urology]. Urologe A. 2011 Sep;50 Suppl 1:5-6. German.
447. Strobush L, Berg R, Cross D, Foth W, Kitchner T, Coleman L, McCarty CA. Dietary intake in the Personalized Medicine Research Project: a resource for studies of gene-diet interaction. Nutr J. 2011 Jan 28;10:13.
448. Strong JM. French maritime pine bark extract (Pycnogenol**®**) and the use of health supplements in the age of personalized medicine. Panminerva Med. 2011 Sep;53(3 Suppl 1):1-2. **NO ACCESS**
449. Sudhindra A, Ochoa R, Santos ES. Biomarkers, Prediction, and Prognosis in Non-Small-Cell Lung Cancer: A Platform for Personalized Treatment. Clin Lung Cancer. 2011 May 8. **NO ACCESS**
450. Sugiyama M, Mizokami M. [Genome-wide association study on and the clinical application to chronic hepatitis C]. Uirusu. 2011 Jun;61(1):15-24. Review. Japanese.
451. Sullivan SD, Garrison LP, Rinde H, Kolberg J, Moler EJ. Cost-effectiveness of risk stratification for preventing type 2 diabetes using a multi-marker diabetes risk score. J Med Econ. 2011 Jul 11. **NO ACCESS**
452. Sun XY, Nong J, Qin K, Warnock GL, Dai LJ. Mesenchymal stem cell-mediated cancer therapy: A dual-targeted strategy of personalized medicine. World J Stem Cells. 2011 Nov 26;3(11):96-103.
453. Swanton C, Burrell RA, Futreal PA. Breast cancer genome heterogeneity: a challenge to personalised medicine? Breast Cancer Res. 2011 Feb 1;13(1):104.
454. Swords RT, Dezube BJ, Medeiros BC. Personalized medicine for acute myelogenous leukemia-At the entrance gate. Am J Hematol. 2011 Aug;86(8):631-2. doi: 10.1002/ajh.22060.
455. Szafran AT, Sun H, Hartig S, Shen Y, Mediwala SN, Bell J, McPhaul MJ, Mancini MA, Marcelli M. Androgen receptor mutations associated with androgen insensitivity syndrome: a high content analysis approach leading to personalized medicine. Adv Exp Med Biol. 2011;707:63-5. **NO ACCESS**
456. Szefler SJ, Dakhama A. New insights into asthma pathogenesis and treatment. Curr Opin Immunol. 2011 Dec;23(6):801-7. Epub 2011 Aug 11. Review.
457. Szefler SJ. Advances in pediatric asthma in 2010: addressing the major issues. J Allergy Clin Immunol. 2011 Jan;127(1):102-15. Review.
458. Szefler SJ. Personalised medicine for asthma management in pregnancy. Lancet. 2011 Sep 10;378(9795):963-4. **NO ACCESS**
459. Tafe LJ, Tsongalis GJ. The human epidermal growth factor receptor 2 (HER2). Clin Chem Lab Med. 2011 Sep 15;50(1):23-30. doi: 10.1515/CCLM.2011.707. **NO ACCESS**
460. Tassa C, Shaw SY, Weissleder R. Dextran-Coated Iron Oxide Nanoparticles: A Versatile Platform for Targeted Molecular Imaging, Molecular Diagnostics, and Therapy. Acc Chem Res. 2011 Jun 10. **NO ACCESS**
461. Taylor AT, Rogers JC. The ethical implications of genetic testing in the classroom. Biochem Mol Biol Educ. 2011 Jul;39(4):253-60. doi: 10.1002/bmb.20521.
462. Tayo BO, Teil M, Tong L, Qin H, Khitrov G, Zhang W, Song Q, Gottesman O, Zhu X, Pereira AC, Cooper RS, Bottinger EP. Genetic background of patients from a university medical center in Manhattan: implications for personalized medicine. PLoS One. 2011 May 4;6(5):e19166.
463. Thill PG, Goswami P, Berchem G, Domon B. Lung cancer statistics in Luxembourg from 1981 to 2008. Bull Soc Sci Med Grand Duche Luxemb. 2011;(2):43-55. **NO ACCESS**
464. Thomas G. Tissue banking for research: connecting the disconnected. Cell Tissue Bank. 2011 Feb;12(1):29-30.
465. Thompson CA. Regulations, economics hindering adoption of personalized medicine. Am J Health Syst Pharm. 2011 Mar 1;68(5):372-4.
466. Tietjen JR, Donato LJ, Bhimisaria D, Ansari AZ. Sequence-specificity and energy landscapes of DNA-binding molecules. Methods Enzymol. 2011;497:3-30. **NO ACCESS**
467. Toft DJ, Cryns VL. Minireview: Basal-like breast cancer: from molecular profiles to targeted therapies. Mol Endocrinol. 2011 Feb;25(2):199-211. **NO ACCESS**
468. Tonellato PJ, Crawford JM, Boguski MS, Saffitz JE. A national agenda for the future of pathology in personalized medicine: report of the proceedings of a meeting at the Banbury Conference Center on genome-era pathology, precision diagnostics, and preemptive care: a stakeholder summit. Am J Clin Pathol. 2011 May;135(5):668-72. **NO ACCESS**
469. Touma C. Stress and affective disorders: animal models elucidating the molecular basis of neuroendocrine-behavior interactions. Pharmacopsychiatry. 2011 May;44 Suppl 1:S15-26.
470. Toumi Z, Bullen C, Tang AC, Dalal N, Ellenbogen S. Metaplastic breast carcinoma: a case report and systematic review of the literature. Pathol Int. 2011 Oct;61(10):582-8. doi: 10.1111/j.1440-1827.2011.02698.x. Epub 2011 Jul 25. Review.
471. Travers ME, McCarthy MI. Type 2 diabetes and obesity: genomics and the clinic. Hum Genet. 2011 Jul;130(1):41-58.
472. Trosman JR, Van Bebber SL, Phillips KA. Health technology assessment and private payers' coverage of personalized medicine. J Oncol Pract. 2011 May;7(3 Suppl):18s-24s.
473. Trosman JR, Van Bebber SL, Phillips KA. Health technology assessment and private payers's coverage of personalized medicine. Am J Manag Care. 2011 May;17(5 Suppl):SP53-60. **NO ACCESS**
474. Tsouti V, Boutopoulos C, Zergioti I, Chatzandroulis S. Capacitive microsystems for biological sensing. Biosens Bioelectron. 2011 Jun 24. **NO ACCESS**
475. Turner SD, Dudek SM, Ritchie MD. Incorporating Domain Knowledge into Evolutionary Computing for Discovering Gene-Gene Interaction. Lect Notes Comput Sci. 2011 Jan 1;6238(2011):394-403. **NO ACCESS**
476. Tursz T, Andre F, Lazar V, Lacroix L, Soria JC. Implications of personalized medicine--perspective from a cancer center. Nat Rev Clin Oncol. 2011 Mar;8(3):177-83. **NO ACCESS**
477. Ullman-Cullere MH, Mathew JP. Emerging landscape of genomics in the Electronic Health Record for personalized medicine. Hum Mutat. 2011 May;32(5):512-6. doi: 10.1002/humu.21456.
478. Ustünkar G, Aydın Son Y. METU-SNP: an integrated software system for SNP-complex disease association analysis. J Integr Bioinform. 2011 Dec 12;8(1):187. doi: 10.2390/biecoll-jib-2011-187. **NO ACCESS**
479. Vallières E, Peters S, Van Houtte P, Dalal P, Lim E. Therapeutic advances in non-small cell lung cancer. Thorax. 2011 Nov 5.
480. van Asseldonk DP, Sanderson J, de Boer NK, Sparrow MP, Lémann M, Ansari A, Almer SH, Florin TH, Gearry RB, Mulder CJ, Mantzaris G, van Bodegraven AA; Thiopurine Task Force Interest Group. Difficulties and possibilities with thiopurine therapy in inflammatory bowel disease--proceedings of the first Thiopurine Task Force meeting. Dig Liver Dis. 2011 Apr;43(4):270-6. Epub 2010 Oct 8. Review.
481. van Baarsen LG, Lodde BM, Tak PP. [Tailored therapy for rheumatic disease within reach]. Ned Tijdschr Geneeskd. 2011;155(30-31):A3569. Dutch.
482. van de Loo FA. Soluble IL-18 receptor complex: a new star in the firmament of rheumatoid arthritis diagnosis? Arthritis Res Ther. 2011 Apr 27;13(2):111. **NO ACCESS**
483. van der Greef J. The 27th Montreux Symposium on LC-MS. Bioanalysis. 2011 May;3(10):1057-9. doi: 10.4155/BIO.11.108. **NO ACCESS**
484. van Rooij T, Marsh S. Improving oncology outcomes through targeted therapeutics will require electronic delivery systems. Future Oncol. 2011 May;7(5):649-56. **NO ACCESS**
485. van Wietmarschen HA, Reijmers TH, van der Kooij AJ, Schroën J, Wei H, Hankemeier T, Meulman JJ, van der Greef J. Sub-typing of rheumatic diseases based on a systems diagnosis questionnaire. PLoS One. 2011;6(9):e24846.
486. Vaught J, Rogers J, Myers K, Lim MD, Lockhart N, Moore H, Sawyer S, Furman JL, Compton C. An NCI Perspective on Creating Sustainable Biospecimen Resources. J Natl Cancer Inst Monogr. 2011;2011(42):1-7. **NO ACCESS**
487. Velikyan I. Positron Emitting [(68)Ga]Ga-Based Imaging Agents: Chemistry and Diversity. Med Chem. 2011 Jun 28. **NO ACCESS**
488. Verweij CL, Vosslamber S. New insight in the mechanism of action of rituximab: the interferon signature towards personalized medicine. Discov Med. 2011 Sep;12(64):229-36. Review. **NO ACCESS**
489. Verweij CL. Pharmacogenetics: Anti-TNF therapy in RA--towards personalized medicine? Nat Rev Rheumatol. 2011 Mar;7(3):136-8. **NO ACCESS**
490. Vik JO, Gjuvsland AB, Li L, Tøndel K, Niederer S, Smith NP, Hunter PJ, Omholt SW. Genotype-Phenotype Map Characteristics of an In silico Heart Cell. Front Physiol. 2011;2:106.
491. Villagra D, Goethe J, Schwartz HI, Szarek B, Kocherla M, Gorowski K, Windemuth A, Ruaño G. Novel drug metabolism indices for pharmacogenetic functional status based on combinatory genotyping of CYP2C9, CYP2C19 and CYP2D6 genes. Biomark Med. 2011 Aug;5(4):427-38.
492. Virgin HW, Todd JA. Metagenomics and personalized medicine. Cell. 2011 Sep 30;147(1):44-56.
493. Vizirianakis IS. Nanomedicine and personalized medicine toward the application of pharmacotyping in clinical practice to improve drug-delivery outcomes. Nanomedicine. 2011 Feb;7(1):11-7. **NO ACCESS**
494. Voora D, Ginsburg GS. A hub for bench-to-bedside pharmacogenomic-based research. Pharmacogenomics. 2011 Aug;12(8):1095-8.
495. Voss SD. Pediatric oncology and the future of oncological imaging. Pediatr Radiol. 2011 May;41 Suppl 1:S172-85.
496. Wadelius M, Alfirevic A. Pharmacogenomics and personalized medicine: the plunge into next-generation sequencing. Genome Med. 2011 Dec 28;3(12):78.
497. Waldman SA, Terzic A. Patient-centric clinical pharmacology advances the path to personalized medicine. Biomark Med. 2011 Dec;5(6):697-700.
498. Waldman SA, Terzic A. Widening the path to personalized medicine. Clin Transl Sci. 2011 Oct;4(5):392-4. doi: 10.1111/j.1752-8062.2011.00345.x. Review. **NO ACCESS**
499. Wallace GR, Niemczyk E. Genetics in ocular inflammation--basic principles. Ocul Immunol Inflamm. 2011 Feb;19(1):10-8. Review.
500. Walling MA, Shepard JR. Cellular heterogeneity and live cell arrays. Chem Soc Rev. 2011 Jul 20;40(7):4049-76. **NO ACCESS**
501. Walt DR, Kuhlik A, Epstein SK, Demmer LA, Knight M, Chelmow D, Rosenblatt M, Bianchi DW. Lessons learned from the introduction of personalized genotyping into a medical school curriculum. Genet Med. 2011 Jan;13(1):63-6.
502. Walther Z, Sklar J. Molecular tumor profiling for prediction of response to anticancer therapies. Cancer J. 2011 Mar-Apr;17(2):71-9. Review. **NO ACCESS**
503. Wang HQ, Liu WY, Wu Z, Tang LJ, Xu XM, Yu RQ, Jiang JH. Homogeneous label-free genotyping of single nucleotide polymorphism using ligation-mediated strand displacement amplification with DNAzyme-based chemiluminescence detection. Anal Chem. 2011 Mar 15;83(6):1883-9. **NO ACCESS**
504. Wang X, Sun H, Zhang A, Sun W, Wang P, Wang Z. Potential role of metabolomics apporoaches in the area of traditional Chinese medicine: as pillars of the bridge between Chinese and Western medicine. J Pharm Biomed Anal. 2011 Jul 15;55(5):859-68.
505. Wang ZQ, Tao YX. Functional studies on twenty novel naturally occurring melanocortin-4 receptor mutations. Biochim Biophys Acta. 2011 Sep;1812(9):1190-9. **NO ACCESS**
506. Ware JS, Roberts AM, Cook SA. Next generation sequencing for clinical diagnostics and personalised medicine: implications for the next generation cardiologist. Heart. 2012 Feb;98(4):276-81. Epub 2011 Nov 29. Review.
507. Warnke C, Stüve O, Hartung HP, Fogdell-Hahn A, Kieseier BC. Critical appraisal of the role of fingolimod in the treatment of multiple sclerosis. Neuropsychiatr Dis Treat. 2011;7:519-27. Epub 2011 Sep 8.
508. Waudby CJ, Berg RL, Linneman JG, Rasmussen LV, Peissig PL, Chen L, McCarty CA. Cataract research using electronic health records. BMC Ophthalmol. 2011 Nov 11;11:32.
509. Wee A. Fine needle aspiration biopsy of hepatocellular carcinoma and hepatocellular nodular lesions: role, controversies and approach to diagnosis. Cytopathology. 2011 Jul 18. doi: 10.1111/j.1365-2303.2011.00882.x. **NO ACCESS**
510. Wee A. Fine-needle aspiration biopsy of hepatocellular carcinoma and related hepatocellular nodular lesions in cirrhosis: controversies, challenges, and expectations. Patholog Res Int. 2011;2011:587936. Epub 2011 Jun 30.
511. Weigelt B, Pusztai L, Ashworth A, Reis-Filho JS. Challenges translating breast cancer gene signatures into the clinic. Nat Rev Clin Oncol. 2011 Aug 30;9(1):58-64. doi: 10.1038/nrclinonc.2011.125.
512. Weissman MM, Brown AS, Talati A. Translational epidemiology in psychiatry: linking population to clinical and basic sciences. Arch Gen Psychiatry. 2011 Jun;68(6):600-8. PubMed PMID: 21646577.
513. Weitzel JN, Blazer KR, Macdonald DJ, Culver JO, Offit K. Genetics, genomics, and cancer risk assessment: State of the Art and Future Directions in the Era of Personalized Medicine. CA Cancer J Clin. 2011 Aug 19. doi: 10.3322/caac.20128.
514. Welch JS, Link DC. Genomics of AML: clinical applications of next-generation sequencing. Hematology Am Soc Hematol Educ Program. 2011;2011:30-5.
515. Wening K, Breitkreutz J. Oral drug delivery in personalized medicine: unmet needs and novel approaches. Int J Pharm. 2011 Feb 14;404(1-2):1-9. **NO ACCESS**
516. Whitcomb DC. Going MAD: development of a "matrix academic division" to facilitate translating research to personalized medicine. Acad Med. 2011 Nov;86(11):1353-9. Review. **NO ACCESS**
517. Wilcken B. Newborn screening: how are we travelling, and where should we be going? J Inherit Metab Dis. 2011 Jun;34(3):569-74.
518. Wilschanski M, Kerem E. New drugs for cystic fibrosis. Expert Opin Investig Drugs. 2011 Jul 11. **NO ACCESS**
519. Wilson NJ, Leachman SA, Hansen CD, McMullan AC, Milstone LM, Schwartz ME, McLean WH, Hull PR, Smith FJ. A large mutational study in pachyonychia congenita. J Invest Dermatol. 2011 May;131(5):1018-24.
520. Witte MH. Translational/personalized medicine, pharmaco/surgico/radiogenomics, lymphatic spread of cancer, and medical ignoromes. J Surg Oncol. 2011 May 1;103(6):501-7. doi: 10.1002/jso.21738. Review.
521. Wojnicz R. [Nanomedicine as the basis of personalised medicine]. Kardiol Pol. 2011;69(10):1107-8. Polish.
522. Wong EH, Fox JC, Ng MY, Lee CM. Toward personalized medicine in the neuropsychiatric field. Int Rev Neurobiol. 2011;101:329-49. **NO ACCESS**
523. Wu AH. Drug metabolizing enzyme activities versus genetic variances for drug of clinical pharmacogenomic relevance. Clin Proteomics. 2011 Jul 28;8(1):12.
524. Wu BQ, Liu Y. [Genotype analysis and personalized medicine]. Zhonghua Bing Li Xue Za Zhi. 2011 Oct;40(10):651-4. Chinese.
525. Xie J, Liu G, Eden HS, Ai H, Chen X. Surface-Engineered Magnetic Nanoparticle Platforms for Cancer Imaging and Therapy. Acc Chem Res. 2011 May 6. **NO ACCESS**
526. Xiong Q, Ge F. Identification and evaluation of a panel of serum biomarkers for predicting response to thalidomide in multiple myeloma patients. Expert Rev Proteomics. 2011 Aug;8(4):439-42. doi: 10.1586/EPR.11.42.
527. Xu Y, Xu Q, Ni S, Liu F, Cai G, Wu F, Ye X, Meng X, Mougin B, Cai S, Du X. Decrease in natural killer cell associated gene expression as a major characteristic of the immune status in the bloodstream of colorectal cancer patients. Cancer Biol Ther. 2011 Jan 15;11(2):188-95. **NO ACCESS**
528. Yan B, Li P. An integrative view of mechanisms underlying generalized spike-and-wave epileptic seizures and its implication on optimal therapeutic treatments. PLoS One. 2011;6(7):e22440.
529. Yang H, Chen X, Wong WH. Completely phased genome sequencing through chromosome sorting. Proc Natl Acad Sci U S A. 2011 Jan 4;108(1):12-7.
530. Yang L, Wang KJ, Wang LS, Jegga AG, Qin SY, He G, Chen J, Xiao Y, He L. Chemical-protein interactome and its application in off-target identification. Interdiscip Sci. 2011 Mar;3(1):22-30. **NO ACCESS**
531. Yang MD, Tsai CW, Chang WS, Tsou YA, Wu CN, Bau DT. Predictive role of XRCC5/XRCC6 genotypes in digestive system cancers. World J Gastrointest Oncol. 2011 Dec 15;3(12):175-81.
532. Yip YL. Genome, and beyond. Findings from the Section on Bioinformatics. Yearb Med Inform. 2011;6(1):156-9. **NO ACCESS**
533. Yoshida S, Sugawara T, Nishio T, Kaneko S. [Personalized medicine for epilepsy based on the pharmacogenomic testing]. Brain Nerve. 2011 Apr;63(4):295-9. Japanese.
534. You W, Widmer N, De Micheli G. Example-based support vector machine for drug concentration analysis. Conf Proc IEEE Eng Med Biol Soc. 2011;2011:153-7. **NO ACCESS**
535. Yu X, Schneiderhan-Marra N, Joos TO. [Protein microarrays and personalized medicine]. Ann Biol Clin (Paris). 2011 Jan-Feb;69(1):17-29. Review. French.
536. Zadák Z, Tichá A, Hronek M, Hyspler R. [Advances in metabolism and nutrition 2011 and the route to personalized treatment]. Vnitr Lek. 2011 Nov;57(11):970-4. Review. Czech.
537. Zaky SS, Lund M, May KA, Godette KD, Beitler JJ, Holmes LR, O'Regan RM, Yu ES, Yu DS, Landry JC. The Negative Effect of Triple-Negative Breast Cancer on Outcome after Breast-Conserving Therapy. Ann Surg Oncol. 2011 Mar 26. **NO ACCESS**
538. Zambon A. Residual cardiovascular risk in secondary prevention. Intern Emerg Med. 2011 Oct;6 Suppl 1:61-8.
539. Zhang H, Tian M, Ignasi C, Cheng Z, Shen LH, Yang DJ. Molecular image-guided theranostic and personalized medicine. J Biomed Biotechnol. 2011;2011:673697.
540. Zhang H, Wang X, Ma Q, Zhou Z, Fang J. Rapid detection of low-abundance K-ras mutation in stools of colorectal cancer patients using chip-based temperature gradient capillary electrophoresis. Lab Invest. 2011 May;91(5):788-98. Epub 2011 Jan 17. PubMed PMID: 21242956.
541. Zhang JP, Malhotra AK. Pharmacogenetics and antipsychotics: therapeutic efficacy and side effects prediction. Expert Opin Drug Metab Toxicol. 2011 Jan;7(1):9-37. Review.
542. Zhang L, Yang H, Xu J. Gene expression significance in personalized medicine of non-small-cell lung cancer and gene expression analyzing platforms. Curr Drug Metab. 2011 Jun 1;12(5):455-9. **NO ACCESS**
543. Zhang W, Zhou HH. [Translational approach for pharmacogenomics and personalized medicine]. Yao Xue Xue Bao. 2011 Jan;46(1):1-5. Chinese.
544. Zhou H, Jiao P, Yang L, Li X, Yan B. Enhancing cell recognition by scrutinizing cell surfaces with a nanoparticle array. J Am Chem Soc. 2011 Feb 2;133(4):680-2. **NO ACCESS**
545. Zhou T, Garcia JG, Zhang W. Integrating microRNAs into a system biology approach to acute lung injury. Transl Res. 2011 Apr;157(4):180-90. **NO ACCESS**
546. Zika E, Paci D, Braun A, Rijkers-Defrasne S, Deschênes M, Fortier I, Laage-Hellman J, Scerri CA, Ibarreta D. A European survey on biobanks: trends and issues. Public Health Genomics. 2011;14(2):96-103.
547. Ziliak D, O'Donnell PH, Im HK, Gamazon ER, Chen P, Delaney S, Shukla S, Das S, Cox NJ, Vokes EE, Cohen EE, Dolan ME, Huang RS. Germline polymorphisms discovered via a cell-based, genome-wide approach predict platinum response in head and neck cancers. Transl Res. 2011 May;157(5):265-72. **NO ACCESS**
548. Zimmer V, Lammert F. Genetics in liver disease: new concepts. Curr Opin Gastroenterol. 2011 May;27(3):231-9.
549. Zineh I, Huang SM. Biomarkers in drug development and regulation: a paradigm for clinical implementation of personalized medicine. Biomark Med. 2011 Dec;5(6):705-13. Review. **NO ACCESS**
550. Zinn KR, Anderson CJ, Bradbury M, Cutler CS, Peterson TE, Morgan DE, Price JC, Graham MM, Contag CH, Wittstrom K, Norenberg JP. Components of a curriculum for molecular imaging scientists. J Nucl Med. 2011 Apr;52(4):650-6.
551. Zografos GC, Roukos DH. Innovative biomarker development for personalized medicine in breast cancer care. Biomark Med. 2011 Feb;5(1):73-8.

**No author stated:**

1. Personalized medicine may prove faster and more effective. Oncology (Williston Park). 2011 Nov 15;25(12):1234. **NO ACCESS**
2. Personalized medicine: identifying the appropriate patient through biomarkers in oncology. P T. 2011 Jul;36(7 Suppl 1):3-10.

**2010**

1. Aartsma-Rus A. Antisense-mediated modulation of splicing: therapeutic implications for Duchenne muscular dystrophy. RNA Biol. 2010 Jul-Aug;7(4):453-61. Epub 2010 Jul 1. Review.
2. Abernethy AP, Etheredge LM, Ganz PA, Wallace P, German RR, Neti C, Bach PB, Murphy SB. Rapid-learning system for cancer care. J Clin Oncol. 2010 Sep 20;28(27):4268-74.
3. Ackerman MJ, Filart R, Burgess LP, Lee I, Poropatich RK. Developing next-generation telehealth tools and technologies: patients, systems, and data perspectives. Telemed J E Health. 2010 Jan-Feb;16(1):93-5.
4. Acosta BM, Bianco AC. New insights into thyroid hormone replacement therapy. F1000 Med Rep. 2010 May 11;2. pii: 34. **NO ACCESS**
5. Al-Hoqail IA. Personalized medicine in psoriasis: concept and applications. Curr Vasc Pharmacol. 2010 May 1;8(3):432-6. Review.
6. Allen KE, Weiss GJ. Resistance may not be futile: microRNA biomarkers for chemoresistance and potential therapeutics. Mol Cancer Ther. 2010 Dec;9(12):3126-36. **NO ACCESS**
7. Almeida JS. Computational ecosystems for data-driven medical genomics. Genome Med. 2010 Sep 20;2(9):67. **NO ACCESS**
8. An Z. Monoclonal antibodies - a proven and rapidly expanding therapeutic modality for human diseases. Protein Cell. 2010 Apr;1(4):319-30. Epub 2010 May 8. Review. **NO ACCESS**
9. Anderson CJ, Bulte JW, Chen K, Chen X, Khaw BA, Shokeen M, Wooley KL, VanBrocklin HF. Design of targeted cardiovascular molecular imaging probes. J Nucl Med. 2010 May 1;51 Suppl 1:3S-17S.
10. Andrykowski MA, Burris JL, Walsh E, Small BJ, Jacobsen PB. Attitudes toward information about genetic risk for cognitive impairment after cancer chemotherapy: breast cancer survivors compared with healthy controls. J Clin Oncol. 2010 Jul 20;28(21):3442-7.
11. Anitua E, Sánchez M, Orive G. Potential of endogenous regenerative technology for in situ regenerative medicine. Adv Drug Deliv Rev. 2010 Jun 15;62(7-8):741-52. Epub 2010 Jan 25. Review.
12. Arnold D, Bokemeyer C. [Clinical trials and personalized medicine in oncology?]. Onkologie. 2010;33 Suppl 7:25-9. German.
13. Asselah T. Genetic polymorphism and response to treatment in chronic hepatitis C: the future of personalized medicine. J Hepatol. 2010 Mar;52(3):452-4.
14. Aung KL, Board RE, Ellison G, Donald E, Ward T, Clack G, Ranson M, Hughes A, Newman W, Dive C. Current status and future potential of somatic mutation testing from circulating free DNA in patients with solid tumours. Hugo J. 2010 Dec;4(1-4):11-21.
15. Babic A, Loftin IR, Stanislaw S, Wang M, Miller R, Warren SM, Zhang W, Lau A, Miller M, Wu P, Padilla M, Grogan TM, Pestic-Dragovich L, McElhinny AS. The impact of pre-analytical processing on staining quality for H&E, dual hapten, dual color in situ hybridization and fluorescent in situ hybridization assays. Methods. 2010 Dec;52(4):287-300. **NO ACCESS**
16. Bachas C, Schuurhuis GJ, Hollink IH, Kwidama ZJ, Goemans BF, Zwaan CM, van den Heuvel-Eibrink MM, de Bont ES, Reinhardt D, Creutzig U, de Haas V, Assaraf YG, Kaspers GJ, Cloos J. High-frequency type I/II mutational shifts between diagnosis and relapse are associated with outcome in pediatric AML: implications for personalized medicine. Blood. 2010 Oct 14;116(15):2752-8. **NO ACCESS**
17. Bailey RC. Grand challenge commentary: Informative diagnostics for personalized medicine. Nat Chem Biol. 2010 Dec;6(12):857-9. **NO ACCESS**
18. Baker SG, Sargent DJ. Designing a randomized clinical trial to evaluate personalized medicine: a new approach based on risk prediction. J Natl Cancer Inst. 2010 Dec 1;102(23):1756-9. **NO ACCESS**
19. Barnes KC. Ancestry, ancestry-informative markers, asthma, and the quest for personalized medicine. J Allergy Clin Immunol. 2010 Dec;126(6):1139-40. **NO ACCESS**
20. Bartova L, Berger A, Pezawas L. Is there a personalized medicine for mood disorders? Eur Arch Psychiatry Clin Neurosci. 2010 Nov;260 Suppl 2:S121-6. Epub 2010 Oct 19. Review. **NO ACCESS**
21. Basu S. From pure research imaging tool to PET-guided personalized medicine in oncology: a true revolution in modern medicine. Indian J Cancer. 2010 Apr-Jun;47(2):98-9.
22. Basu S. Personalized versus evidence-based medicine with PET-based imaging. Nat Rev Clin Oncol. 2010 Nov;7(11):665-8. **NO ACCESS**
23. Bates S. Progress towards personalized medicine. Drug Discov Today. 2010 Feb;15(3-4):115-20. Epub 2009 Nov 13. Review.
24. Bathen TF, Sitter B, Sjøbakk TE, Tessem MB, Gribbestad IS. Magnetic resonance metabolomics of intact tissue: a biotechnological tool in cancer diagnostics and treatment evaluation. Cancer Res. 2010 Sep 1;70(17):6692-6. Epub 2010 Aug 10. Review.
25. Becker ML, Leeder JS. Identifying genomic and developmental causes of adverse drug reactions in children. Pharmacogenomics. 2010 Nov;11(11):1591-602. Review. **NO ACCESS**
26. Behrens J. [Evidence-based medicine - the current self-reflection of an individualised approach to medicine as an action science]. Z Evid Fortbild Qual Gesundhwes. 2010;104(8-9):617-24. Epub 2010 Oct 29. German.
27. Beijnen JH, Schellens JH. Personalized medicine in oncology: a personal view with myths and facts. Curr Clin Pharmacol. 2010 Aug;5(3):141-7. Review.
28. Belloso WH, Redal MA. [Pharmacogenomics and the path towards personalized medicine]. Medicina (B Aires). 2010;70(3):265-74. Review. Spanish.
29. Bendtzen K. Critical review: assessment of interferon-β immunogenicity in multiple sclerosis. J Interferon Cytokine Res. 2010 Oct;30(10):759-66. Review. **NO ACCESS**
30. Besse B, Vignot S, Soria JC. [Lung cancer: an update in 2010]. Bull Cancer. 2010 Jan;97(1):161-9. French.
31. Blakemore AI, Froguel P. Investigation of Mendelian forms of obesity holds out the prospect of personalized medicine. Ann N Y Acad Sci. 2010 Dec;1214:180-9. doi: 10.1111/j.1749-6632.2010.05880.x. Review.
32. Blanchet KD. Redefining personalized medicine in the postgenomic era: developing bladder cancer therapeutics with proteomics. BJU Int. 2010 Jan;105(2):i-iii.
33. Blokzijl A, Friedman M, Pontén F, Landegren U. Profiling protein expression and interactions: proximity ligation as a tool for personalized medicine. J Intern Med. 2010 Sep;268(3):232-45. Review. **NO ACCESS**
34. Blum HE. Individualized medicine 2010. J Cell Mol Med. 2010 Sep;14(9):2257-63. doi: 10.1111/j.1582-4934.2010.01155.x. **NO ACCESS**
35. Bolton D. Conceptualisation of mental disorder and its personal meanings. J Ment Health. 2010 Aug;19(4):328-36. Review. **NO ACCESS**
36. Booth FW, Laye MJ. The future: genes, physical activity and health. Acta Physiol (Oxf). 2010 Aug;199(4):549-56. doi: 10.1111/j.1748-1716.2010.02117.x.
37. Bouchelouche K, Capala J. 'Image and treat': an individualized approach to urological tumors. Curr Opin Oncol. 2010 May;22(3):274-80. Review.
38. Brasier AR, Victor S, Ju H, Busse WW, Curran-Everett D, Bleecker E, Castro M, Chung KF, Gaston B, Israel E, Wenzel SE, Erzurum SC, Jarjour NN, Calhoun WJ. Predicting intermediate phenotypes in asthma using bronchoalveolar lavage-derived cytokines. Clin Transl Sci. 2010 Aug;3(4):147-57. **NO ACCESS**
39. Bristol-Gould S, Desjardins M, Woodruff TK. The Illinois Women's Health Registry: advancing women's health research and education in Illinois, USA. Womens Health (Lond Engl). 2010 Mar;6(2):183-96.
40. Brown TS, Safford S, Caramanica J, Elster EA. Biomarker use in tailored combat casualty care. Biomark Med. 2010 Jun;4(3):465-73. **NO ACCESS**
41. Burg T, Cass CA, Groff R, Pepper M, Burg KJ. Building off-the-shelf tissue-engineered composites. Philos Transact A Math Phys Eng Sci. 2010 Apr 28;368(1917):1839-62. Review.
42. Burke W, Burton H, Hall AE, Karmali M, Khoury MJ, Knoppers B, Meslin EM, Stanley F, Wright CF, Zimmern RL; Ickworth Group. Extending the reach of public health genomics: what should be the agenda for public health in an era of genome-based and “personalized” medicine? Genet Med. 2010 Dec;12(12):785-91.
43. Burmester JK, Sedova M, Shapero MH, Mansfield E. DMET microarray technology for pharmacogenomics-based personalized medicine. Methods Mol Biol. 2010;632:99-124. **NO ACCESS**
44. Burns DK. Developing pharmacogenetic evidence throughout clinical development. Clin Pharmacol Ther. 2010 Dec;88(6):867-70. **NO ACCESS**
45. Campr V. [Personalized medicine in haematooncology--the pathologist's perspective]. Cas Lek Cesk. 2010;149(10):464-7. Review. Czech.
46. Carney KK. Personalized medicine. J Calif Dent Assoc. 2010 Apr;38(4):217-8.
47. Cascorbi I. The promises of personalized medicine. Eur J Clin Pharmacol. 2010 Aug;66(8):749-54.
48. Castaldo G, Lembo F, Tomaiuolo R. Molecular diagnostics: between chips and customized medicine. Clin Chem Lab Med. 2010 Jul;48(7):973-82. Review.
49. Catchpoole DR, Kennedy P, Skillicorn DB, Simoff S. The curse of dimensionality: a blessing to personalized medicine. J Clin Oncol. 2010 Dec 1;28(34):e723-4; author reply e725.
50. Cavalieri D. Evolution of transcriptional regulatory networks in yeast populations. Wiley Interdiscip Rev Syst Biol Med. 2010 May-Jun;2(3):324-35. Review. **NO ACCESS**
51. Chakrabarti S, Freedman JE. Review: Nutriceuticals as antithrombotic agents. Cardiovasc Ther. 2010 Aug;28(4):227-35.
52. Chakraborty C, Shah KD, Cao WG, Hsu CH, Wen ZH, Lin CS. Potentialities of induced pluripotent stem (iPS) cells for treatment of diseases. Curr Mol Med. 2010 Nov;10(8):756-62. Review. **NO ACCESS**
53. Chan IS, Ginsburg GS. Personalized Medicine: Progress and Promise. Annu Rev Genomics Hum Genet. 2010 Sep 29. **NO ACCESS**
54. Chan N, Bristow RG. "Contextual" synthetic lethality and/or loss of heterozygosity: tumor hypoxia and modification of DNA repair. Clin Cancer Res. 2010 Sep 15;16(18):4553-60. **NO ACCESS**
55. Chan WC, Armitage JO. Genomic analysis of lymphoma: potential for clinical application. J Natl Compr Canc Netw. 2010 Mar;8(3):353-60.
56. Chang RL, Xie L, Xie L, Bourne PE, Palsson BØ. Drug off-target effects predicted using structural analysis in the context of a metabolic network model. PLoS Comput Biol. 2010 Sep 23;6(9):e1000938.
57. Charkoudian N, Gusman E, Joyner MJ, Wallin BG, Osborn J. Integrative mechanisms of blood pressure regulation in humans and rats: cross-species similarities. Am J Physiol Regul Integr Comp Physiol. 2010 Mar;298(3):R755-9. doi: 10.1152/ajpregu.00607.2009.
58. Chen K, Chen X. Design and development of molecular imaging probes. Curr Top Med Chem. 2010;10(12):1227-36. Review.
59. Cleynen I, Mahachie John JM, Henckaerts L, Van Moerkercke W, Rutgeerts P, Van Steen K, Vermeire S. Molecular reclassification of Crohn's disease by cluster analysis of genetic variants. PLoS One. 2010 Sep 23;5(9):e12952.
60. Coelho T. A patient advocate's perspective on patient-centered comparative effectiveness research. Health Aff (Millwood). 2010 Oct;29(10):1885-90. **NO ACCESS**
61. Conti R, Veenstra DL, Armstrong K, Lesko LJ, Grosse SD. Personalized medicine and genomics: challenges and opportunities in assessing effectiveness, cost-effectiveness, and future research priorities. Med Decis Making. 2010 May-Jun;30(3):328-40.
62. Cross DS, Ivacic LC, Stefanski EL, McCarty CA. Population based allele frequencies of disease associated polymorphisms in the Personalized Medicine Research Project. BMC Genet. 2010 Jun 17;11:51.
63. Dai XJ, Jiang WJ, Wang WM, Zhao SJ. Drug or vaccine?: selecting the appropriate treatment for malignant glioma patients. Drugs. 2010 Aug 20;70(12):1477-86. doi: 10.2165/11538040-000000000-00000. Review.
64. Dalca AV, Brudno M. Genome variation discovery with high-throughput sequencing data. Brief Bioinform. 2010 Jan;11(1):3-14.
65. Dalton WS, Sullivan DM, Yeatman TJ, Fenstermacher DA. The 2010 Health Care Reform Act: a potential opportunity to advance cancer research by taking cancer personally. Clin Cancer Res. 2010 Dec 15;16(24):5987-96. Review. **NO ACCESS**
66. Dandekar T, Dandekar G. Pharmacogenomic strategies against microbial resistance: from bright to bleak to innovative. Pharmacogenomics. 2010 Sep;11(9):1193-6. doi: 10.2217/pgs.10.18.
67. Dangl A, Demiroglu SY, Gaedcke J, Helbing K, Jo P, Rakebrandt F, Rienhoff O, Sax U. The IT-infrastructure of a biobank for an academic medical center. Stud Health Technol Inform. 2010;160(Pt 2):1334-8. **NO ACCESS**
68. Das T, Cagan R. Drosophila as a novel therapeutic discovery tool for thyroid cancer. Thyroid. 2010 Jul;20(7):689-95. Review.
69. del Barrio Seoane J. [Pharmacogenomics in clinical trials]. Rev Derecho Genoma Hum. 2010 Jul-Dec;(33):197-216. Spanish.
70. den Exter A. Personalized medicine and access to genetic technologies. Rev Derecho Genoma Hum. 2010 Jul-Dec;(33):49-64. **NO ACCESS**
71. Di Francia R, Frigeri F, Berretta M, Cecchin E, Orlando C, Pinto A, Pinzani P. Decision criteria for rational selection of homogeneous genotyping platforms for pharmacogenomics testing in clinical diagnostics. Clin Chem Lab Med. 2010 Apr;48(4):447-59. Review.
72. Diamandis M, White NM, Yousef GM. Personalized medicine: marking a new epoch in cancer patient management. Mol Cancer Res. 2010 Sep;8(9):1175-87. Epub 2010 Aug 6. Review. **NO ACCESS**
73. Dion-Labrie M, Fortin MC, Hébert MJ, Doucet H. The use of personalized medicine for patient selection for renal transplantation: physicians' views on the clinical and ethical implications. BMC Med Ethics. 2010 Apr 9;11:5.
74. Disanto G, Berlanga AJ, Handel AE, Para AE, Burrell AM, Fries A, Handunnetthi L, De Luca GC, Morahan JM. Heterogeneity in multiple sclerosis: scratching the surface of a complex disease. Autoimmune Dis. 2010 Dec 9;2011:932351.
75. Djordjević V, Rakićević L, Radojković D. An overview of genetic risk factors in thrombophilia. Srp Arh Celok Lek. 2010 Jan;138 Suppl 1:79-81. Review. **NO ACCESS**
76. Donovan MJ, Costa J, Cordon-Cardo C. Personalized approach to prostate cancer prognosis. Minerva Urol Nefrol. 2010 Sep;62(3):231-9. Review. **NO ACCESS**
77. Durner J. Clinical chemistry: challenges for analytical chemistry and the nanosciences from medicine. Angew Chem Int Ed Engl. 2010 Feb 1;49(6):1026-51. Review.
78. Dzik-Jurasz A. Imaging in drug development: will it deliver on the promise of personalised medicine? Br J Radiol. 2010 Jun;83(990):453-5. **NO ACCESS**
79. Ekroos K, Jänis M, Tarasov K, Hurme R, Laaksonen R. Lipidomics: a tool for studies of atherosclerosis. Curr Atheroscler Rep. 2010 Jul;12(4):273-81.
80. Elbein SC. Genetics factors contributing to type 2 diabetes across ethnicities. J Diabetes Sci Technol. 2009 Jul 1;3(4):685-9. Review.
81. Ellsworth RE, Decewicz DJ, Shriver CD, Ellsworth DL. Breast cancer in the personal genomics era. Curr Genomics. 2010 May;11(3):146-61.
82. Emerson CH. May 2010: a personalized medicine month in Minneapolis, Minnesota. Thyroid. 2010 Apr;20(4):355-6.
83. Epstein R, Teagarden JR. Comparative effectiveness and personalized medicine: evolving together or apart? Health Aff (Millwood). 2010 Oct;29(10):1783-7. **NO ACCESS**
84. Epstein RS, Teagarden JR. Comparative effectiveness research and personalized medicine: catalyzing or colliding? Pharmacoeconomics. 2010;28(10):905-13. doi: 10.2165/11535830-000000000-00000. **NO ACCESS**
85. Erichsen R, Lash TL, Hamilton-Dutoit SJ, Bjerregaard B, Vyberg M, Pedersen L. Existing data sources for clinical epidemiology: the Danish National Pathology Registry and Data Bank. Clin Epidemiol. 2010 Aug 9;2:51-6.
86. Erickson HS, Gannot G, Tangrea MA, Chuaqui RF, Gillespie JW, Emmert-Buck MR. High throughput screening of normal and neoplastic tissue samples. Comb Chem High Throughput Screen. 2010 Mar;13(3):253-67. Review.
87. Esch MB, King TL, Shuler ML. The role of body-on-a-chip devices in drug and toxicity studies. Annu Rev Biomed Eng. 2011 Aug 15;13:55-72. doi: 10.1146/annurev-bioeng-071910-124629. Review. **NO ACCESS**
88. Etgar L, Nakhmani A, Tannenbaum A, Lifshitz E, Tannenbaum R. Trajectory control of PbSe-gamma-Fe2O3 nanoplatforms under viscous flow and an external magnetic field. Nanotechnology. 2010 Apr 30;21(17):175702.
89. Fändrich F. Cell therapy approaches aiming at minimization of immunosuppression in solid organ transplantation. Curr Opin Organ Transplant. 2010 Oct 7. **NO ACCESS**
90. Ferroli P, Acerbi F, Finocchiaro G. From standard treatment to personalized medicine: role of IDH1 mutations in low-grade glioma evolution and treatment.World Neurosurg. 2010 Apr;73(4):234-6. **NO ACCESS**
91. Foley KF, Quigley DI. Pharmacogenomic potential of psychiatric medications and CYP2D6. MLO Med Lab Obs. 2010 Jan;42(1):32-4. . **NO ACCESS**
92. Foster A, Miller del D, Buckley P. Pharmacogenetics and schizophrenia. Clin Lab Med. 2010 Dec;30(4):975-93. Review. **NO ACCESS**
93. Francis LP. You are born with your genes: justice and protection against discrimination in the use of genetic information. Mt Sinai J Med. 2010 Mar;77(2):188-96.
94. Fredolini C, Liotta LA, Petricoin EF. Application of proteomic technologies for prostate cancer detection, prognosis, and tailored therapy. Crit Rev Clin Lab Sci. 2010 May-Jun;47(3):125-38. doi: 10.3109/10408363.2010.503558. Review. **NO ACCESS**
95. Freeman RA. Personalized medicine and therapeutic decision-making in oncology: a commentary on environmental issues. J Oncol Pharm Pract. 2010 Jun 15. **NO ACCESS**
96. Freidlin B, McShane LM, Korn EL. Randomized clinical trials with biomarkers: design issues. J Natl Cancer Inst. 2010 Feb 3;102(3):152-60.
97. Freire A, Seoane JA, Rodríguez A, Ruiz-Romero C, López-Campos G, Dorado J. A Block-matching based technique for the analysis of 2D gel images. Stud Health Technol Inform. 2010;160(Pt 2):1282-6. **NO ACCESS**
98. French B, Joo J, Geller NL, Kimmel SE, Rosenberg Y, Anderson JL, Gage BF, Johnson JA, Ellenberg JH; COAG (Clarification of Optimal Anticoagulation through Genetics) Investigators. Statistical design of personalized medicine interventions: the Clarification of Optimal Anticoagulation through Genetics (COAG) trial. Trials. 2010 Nov 17;11:108.
99. Fucic A, Gamulin M, Ferencic Z, Rokotov DS, Katic J, Bartonova A, Lovasic IB, Merlo DF. Lung cancer and environmental chemical exposure: a review of our current state of knowledge with reference to the role of hormones and hormone receptors as an increased risk factor for developing lung cancer in man. Toxicol Pathol. 2010;38(6):849-55.
100. Fujio Y. RGS2 determines the preventive effects of ARBs against vascular remodeling: toward personalized medicine of anti-hypertensive therapy with ARBs. Hypertens Res. 2010 Dec;33(12):1221-2. **NO ACCESS**
101. Fujiwara Y, Minami H. An overview of the recent progress in irinotecan pharmacogenetics. Pharmacogenomics. 2010 Mar;11(3):391-406. Review. **NO ACCESS**
102. Furukori N. [Clinical pharmacogenetics in the treatment of schizophrenia]. Nihon Shinkei Seishin Yakurigaku Zasshi. 2010 Apr;30(2):65-9. Review. Japanese.
103. Galsky MD, Hall SJ. Bladder cancer: current management and opportunities for a personalized approach. Mt Sinai J Med. 2010 Nov Dec;77(6):587-96. doi: 10.1002/msj.20224. Review. **NO ACCESS**
104. Gandellini P, Folini M, Zaffaroni N. Emerging role of microRNAs in prostate cancer: implications for personalized medicine. Discov Med2010 Mar;9(46):212-8. Review.
105. Ganz PA. Harnessing personalised medicine to prevent late effects. Lancet Oncol. 2010 Jan;11(1):7-9. **NO ACCESS**
106. Gao J, Chen X, Cheng Z. Near-infrared quantum dots as optical probes for tumor imaging. Curr Top Med Chem. 2010;10(12):1147-57. Review.
107. Geisler T, Gawaz M, Steinhubl SR, Bhatt DL, Storey RF, Flather M. Current strategies in antiplatelet therapy--does identification of risk and adjustment of therapy contribute to more effective, personalized medicine in cardiovascular disease? Pharmacol Ther. 2010 Aug;127(2):95-107.
108. Gelenberg AJ. Depression symptomatology and neurobiology. J Clin Psychiatry. 2010 Jan;71(1):e02. Review. **NO ACCESS**
109. Gerber DE, Minna JD. ALK inhibition for non-small cell lung cancer: from discovery to therapy in record time. Cancer Cell. 2010 Dec 14;18(6):548-51. Review.
110. Gesteira A, Barros F, Martín A, Pérez V, Cortés A, Baiget M, Carracedo A.Pharmacogenetic studies on the antipsychotic treatment. Current status and perspectives. Actas Esp Psiquiatr. 2010 Sep-Oct;38(5):301-16. Epub 2010 Sep 1. Review. English, Spanish.
111. Ghebranious N, Mukesh B, Giampietro PF, Glurich I, Mickel SF, Waring SC, McCarty CA. A pilot study of gene/gene and gene/environment interactions in Alzheimer disease. Clin Med Res. 2011 Mar;9(1):17-25.
112. Ghosh R, Ghosh S, Chawla S. Pharmacogenomics--practice and challenges. Aust Fam Physician. 2010 Oct;39(10):788-90.
113. Girard N, Mornex F. Highlights of the 2nd European Lung Cancer Conference. Expert Rev Anticancer Ther. 2010 Jul;10(7):1023-5
114. Giusiano S, Formisano-Tréziny C, Benziane A, Maroc N, Picard C, Hermitte F, Taranger-Charpin C, Gabert J. Development of a biochip-based assay integrated in a global strategy for identification of fusion transcripts in acute myeloid leukemia: a work flow for acute myeloid leukemia diagnosis. Int J Lab Hematol. 2010 Aug 1;32(4):398-409. **NO ACCESS**
115. Gladding P, White H, Webster M. Prasugrel, Māori, and personalised medicine in New Zealand. N Z Med J. 2010 Mar 5;123(1310):86-90. NO ACCESS
116. Goldfarb-Rumyantzev AS. Personalized medicine and prediction of outcome in kidney transplant. Am J Kidney Dis. 2010 Nov;56(5):817-9. **NO ACCESS**
117. Goldie MP. Personalized medicine and informatics. Int J Dent Hyg. 2010 Feb;8(1):76-7. **NO ACCESS**
118. Gomah ME, Turley JP, Lu H, Jones D. Modeling complex workflow in molecular diagnostics: design specifications of laboratory software for support of personalized medicine. J Mol Diagn. 2010 Jan;12(1):51-7.
119. Gonzalez-Angulo AM, Hennessy BT, Mills GB. Future of personalized medicine in oncology: a systems biology approach. J Clin Oncol. 2010 Jun 1;28(16):2777-83. Epub 2010 Apr 20. Review.
120. González-Tejera G, Gaedigk A, Corey S. Genetic variants of the drug-metabolizing enzyme CYP2D6 in Puerto Rican psychiatry patients: a preliminary report and potential implications for breast cancer patients. P R Health Sci J. 2010 Sep;29(3):299-304. **NO ACCESS**
121. Greeley SA, Tucker SE, Naylor RN, Bell GI, Philipson LH. Neonatal diabetes mellitus: a model for personalized medicine. Trends Endocrinol Metab. 2010 Aug;21(8):464-72. Epub 2010 Apr 29. Review.
122. Green JS, O'Brien TJ, Chiappinelli VA, Harralson AF. Pharmacogenomics instruction in US and Canadian medical schools: implications for personalized medicine. Pharmacogenomics. 2010 Sep;11(9):1331-40. **NO ACCESS**
123. Greenberger PA. Personalized medicine for patients with asthma. J Allergy Clin Immunol. 2010 Feb;125(2):305-6. **NO ACCESS**
124. Grizzle WE, Srivastava S, Manne U. Translational pathology of neoplasia. Cancer Biomark. 2010;9(1-6):7-20. **NO ACCESS**
125. Grossman I, Lutz MW, Crenshaw DG, Saunders AM, Burns DK, Roses AD. Alzheimer's disease: diagnostics, prognostics and the road to prevention. EPMA J. 2010 Jun;1(2):293-303.
126. Guo J, Yu L, Turro NJ, Ju J. An integrated system for DNA sequencing by synthesis using novel nucleotide analogues. Acc Chem Res. 2010 Apr 20;43(4):551-63.
127. Gurwitz D, Pirmohamed M. Pharmacogenomics: the importance of accurate phenotypes. Pharmacogenomics. 2010 Apr;11(4):469-70.
128. Guţiu IA, Andrieş A, Mircioiu C, Rădulescu F, Georgescu AM, Cioacă D. Pharmacometabonomics, pharmacogenomics and personalized medicine. Rom J Intern Med. 2010;48(2):187-91. Review.
129. Hüsing B. [Individualised medicine - potentials and need for action]. Z Evid Fortbild Qual Gesundhwes. 2010;104(10):727-31. German.
130. Haddy CA, Ward HM, Angley MT, McKinnon RA. Consumers' views of pharmacogenetics--A qualitative study. Res Social Adm Pharm. 2010 Sep;6(3):221-31. **NO ACCESS**
131. Hadorn M, Eggenberger Hotz P. DNA-mediated self-assembly of artificial vesicles. PLoS One. 2010 Mar 26;5(3):e9886.
132. Hahn E, Kraus S, Arber N. Role of cyclooxygenase-2 in pathogenesis and prevention of colorectal cancer. Dig Dis. 2010;28(4-5):585-9.
133. Hamburg MA, Collins FS. The path to personalized medicine. N Engl J Med. 2010 Jul 22;363(4):301-4. Epub 2010 Jun 15. Erratum in: N Engl J Med. 2010 Sep 9;363(11):1092.
134. Hammer RR. An education that pierces what the knife cannot: a student perspective. Anat Sci Educ. 2010 May-Jun;3(3):151-3. **NO ACCESS**
135. Haspel RL, Arnaout R, Briere L, Kantarci S, Marchand K, Tonellato P, Connolly J, Boguski MS, Saffitz JE. A call to action: training pathology residents in genomics and personalized medicine. Am J Clin Pathol. 2010 Jun;133(6):832-4.
136. Hayes DF. Contribution of biomarkers to personalized medicine. Breast Cancer Res. 2010 Dec 20;12 Suppl 4:S3.
137. Hays JL, Kim G, Giuroiu I, Kohn EC. Proteomics and ovarian cancer: integrating proteomics information into clinical care. J Proteomics. 2010 Sep 10;73(10):1864-72. Epub 2010 Jun 1. Review.
138. Heidecker B, Lamirault G, Kasper EK, Wittstein IS, Champion HC, Breton E, Russell SD, Hall J, Kittleson MM, Baughman KL, Hare JM. The gene expression profile of patients with new-onset heart failure reveals important gender-specific differences. Eur Heart J. 2010 May;31(10):1188-96.
139. Helmy KY, Patel SA, Silverio K, Pliner L, Rameshwar P. Stem cells and regenerative medicine: accomplishments to date and future promise. Ther Deliv. 2010 Nov;1(5):693-705.
140. Hermsdorff HH, Puchau B, Zulet MA, Martínez JA. Association of body fat distribution with proinflammatory gene expression in peripheral blood mononuclear cells from young adult subjects. OMICS. 2010 Jun;14(3):297-307. **NO ACCESS**
141. Hernandez-Fuentes MP, Lechler RI. A 'biomarker signature' for tolerance in transplantation. Nat Rev Nephrol. 2010 Oct;6(10):606-13. **NO ACCESS**
142. Hildebrandt F. Genetic kidney diseases. Lancet. 2010 Apr 10;375(9722):1287-95. Review.
143. Hirsch FR, Wynes MW, Gandara DR, Bunn PA Jr. The tissue is the issue: personalized medicine for non-small cell lung cancer. Clin Cancer Res. 2010 Oct 15;16(20):4909-11. **NO ACCESS**
144. Hirschfield GM, Amos CI, Siminovitch KA. Navigating the road to personalized medicine: can we believe? CMAJ. 2010 Apr 20;182(7):651-2.
145. Hjelle SM, Forthun RB, Haaland I, Reikvam H, Sjøholt G, Bruserud O, Gjertsen BT. Clinical proteomics of myeloid leukemia. Genome Med. 2010 Jun 29;2(6):41.
146. Hojo Y, Echizenya M, Ohkubo T, Shimizu T. Drug interaction between St John's wort and zolpidem in healthy subjects. J Clin Pharm Ther. 2010 Nov 8. doi: 10.1111/j.1365-2710.2010.01223.x. **NO ACCESS**
147. Holman CM. Bilski: assessing the impact of a newly invigorated patent-eligibility doctrine on the pharmaceutical industry and the future of personalized medicine. Curr Top Med Chem. 2010;10(18):1937-49. **NO ACCESS**
148. Holsboer F, Ising M. Stress hormone regulation: biological role and translation into therapy. Annu Rev Psychol. 2010;61:81-109, C1-11. Review. **NO ACCESS**
149. Hong H, Yang Y, Zhang Y, Cai W. Non-invasive cell tracking in cancer and cancer therapy. Curr Top Med Chem. 2010;10(12):1237-48. Review.
150. Hong KW, Oh B. Overview of personalized medicine in the disease genomic era. BMB Rep. 2010 Oct;43(10):643-8. Review.
151. Hopkins PN. Defining the challenges of familial hypercholesterolemia screening: introduction. J Clin Lipidol. 2010 Sep-Oct;4(5):342-5. **NO ACCESS**
152. Hricak H, Choi BI, Scott AM, Sugimura K, Muellner A, von Schulthess GK, Reiser MF, Graham MM, Dunnick NR, Larson SM. Global trends in hybrid imaging. Radiology. 2010 Nov;257(2):498-506.
153. Huizenga CR, Lowstuter K, Banks KC, Lagos VI, Vandergon VO, Weitzel JN. Evolving perspectives on genetic discrimination in health insurance among health care providers. Fam Cancer. 2010 Jun;9(2):253-60. **NO ACCESS**
154. Hulin-Curtis SL, Petit D, Figg WD, Hsing AW, Reichardt JK. Finasteride metabolism and pharmacogenetics: new approaches to personalized prevention of prostate cancer. Future Oncol. 2010 Dec;6(12):1897-913. Review. **NO ACCESS**
155. Hulot JS. Pharmacogenomics and personalized medicine: lost in translation? Genome Med. 2010 Feb 22;2(2):13.
156. Hutchison KE. Substance use disorders: realizing the promise of pharmacogenomics and personalized medicine. Annu Rev Clin Psychol. 2010 Apr 27;6:577-89. Review. **NO ACCESS**
157. Inczédy-Farkas G, Benkovits J, Balogh N, Almos P, Scholtz B, Zahuczky G, Török Z, Nagy K, Réthelyi J, Makkos Z, Kassai-Farkas A, Egerházy A, Tuzko J, Janka Z, Bitter I, Németh G, Nagy L, Molnár MJ. [SCHIZOBANK - The Hungarian national schizophrenia biobank and its role in schizophrenia research and in personalized medicine]. Orv Hetil. 2010 Aug 29;151(35):1403-8. Hungarian.
158. Ingelman-Sundberg M, Sim SC. Pharmacogenetic biomarkers as tools for improved drug therapy; emphasis on the cytochrome P450 system. Biochem Biophys Res Commun. 2010 May 21;396(1):90-4. Review.
159. Ioannidis JP. Expectations, validity, and reality in omics. J Clin Epidemiol. 2010 Sep;63(9):945-9. **NO ACCESS**
160. Ishikawa T, Aw W, Lezhava A, Hayashizaki Y. [To promise personalized medicine: development of world's fastest SNP detection method to circumvent drug-induced adverse reactions]. Arerugi. 2010 Feb;59(2):98-108. Review. Japanese.
161. Ishikawa T, Sakurai A, Hirano H, Lezhava A, Sakurai M, Hayashizaki Y. Emerging new technologies in Pharmacogenomics: rapid SNP detection, molecular dynamic simulation, and QSAR analysis methods to validate clinically important genetic variants of human ABC Transporter ABCB1 (P-gp/MDR1). Pharmacol Ther. 2010 Apr;126(1):69-81. doi: 10.1016/j.pharmthera.2010.01.005. Epub 2010 Feb 4. Review. **NO ACCESS**
162. Ishkanian AS, Zafarana G, Thoms J, Bristow RG. Array CGH as a potential predictor of radiocurability in intermediate risk prostate cancer. Acta Oncol. 2010 Oct;49(7):888-94. Review. **NO ACCESS**
163. Jaffe EK. MORPHEEINS - A NEW PATHWAY FOR ALLOSTERIC DRUG DISCOVERY. Open Conf Proc J. 2010;1:1-6
164. Jain KK. Advances in the field of nanooncology. BMC Med. 2010 Dec 13;8:83. Review.
165. Jain KK. Innovative diagnostic technologies and their significance for personalized medicine. Mol Diagn Ther. 2010 Jun 1;14(3):141-7. doi: 10.2165/11536240-000000000-00000. **NO ACCESS**
166. Jain KK. Personalized cancer vaccines. Expert Opin Biol Ther. 2010 Dec;10(12):1637-47. Review.
167. Jakovljević M. The creative psychopharmacotherapy and personalized medicine: The art & practice of the learning organization. Psychiatr Danub. 2010 Jun;22(2):309-12. **NO ACCESS**
168. Janib SM, Moses AS, MacKay JA. Imaging and drug delivery using theranostic nanoparticles. Adv Drug Deliv Rev. 2010 Aug 30;62(11):1052-63. **NO ACCESS**
169. Jansen M, Yip S, Louis DN. Molecular pathology in adult gliomas: diagnostic, prognostic, and predictive markers. Lancet Neurol. 2010 Jul;9(7):717-26. Review.
170. Jena B, Dotti G, Cooper LJ. Redirecting T-cell specificity by introducing a tumor-specific chimeric antigen receptor. Blood. 2010 Aug 19;116(7):1035-44. Epub 2010 May 3. Review.
171. Jiang S, Tsang J, Tam P. Regulatory T cell immunotherapy for transplantation tolerance: step into clinic. Int Immunopharmacol. 2010 Dec;10(12):1486-90. doi: 10.1016/j.intimp.2010.08.007.
172. Jiang Y, Wang M. Personalized medicine in oncology: tailoring the right drug to the right patient. Biomark Med. 2010 Aug;4(4):523-33. Review. **NO ACCESS**
173. Johnson JM, Yu T, Strobel FH, Jones DP. A practical approach to detect unique metabolic patterns for personalized medicine. Analyst. 2010 Nov;135(11):2864-70. **NO ACCESS**
174. Ju YS, Hong D, Kim S, Park SS, Kim S, Lee S, Park H, Kim JI, Seo JS. Reference-unbiased copy number variant analysis using CGH microarrays. Nucleic Acids Res. 2010 Nov 1;38(20):e190.
175. Kantor IN, Belloso WH. [Pharmacogenomics and the path towards personalized medicine]. Medicina (B Aires). 2010;70(5):482-4. Spanish.
176. Kasai Y, Cagan R. Drosophila as a tool for personalized medicine: a primer. Per Med. 2010 Nov;7(6):621-632.
177. Keicho N. [Biomarkers to assess different aspects of tuberculosis—from development to relapse]. Kekkaku. 2010 Nov;85(11):823-8. Review. Japanese.
178. Khan O, Fotheringham S, Wood V, Stimson L, Zhang C, Pezzella F, Duvic M, Kerr DJ, La Thangue NB. HR23B is a biomarker for tumor sensitivity to HDAC inhibitor-based therapy. Proc Natl Acad Sci U S A. 2010 Apr 6;107(14):6532-7.
179. Khoury MJ. Dealing with the evidence dilemma in genomics and personalized medicine. Clin Pharmacol Ther. 2010 Jun;87(6):635-8.
180. Kimura H, Nishio K. [Molecular targeted therapies]. Nippon Rinsho. 2010 Jun;68(6):1047-53. Review. Japanese.
181. Klein RD. Legal developments and practical implications of gene patenting on targeted drug discovery and development. Clin Pharmacol Ther. 2010 Jun;87(6):633-5.
182. Ko JM, Velez NF, Tsao H. Pathways to melanoma. Semin Cutan Med Surg. 2010 Dec;29(4):210-7. Review. **NO ACCESS**
183. Kohonen-Corish MR, Al-Aama JY, Auerbach AD, Axton M, Barash CI, Bernstein I, Béroud C, Burn J, Cunningham F, Cutting GR, den Dunnen JT, Greenblatt MS, Kaput J, Katz M, Lindblom A, Macrae F, Maglott D, Möslein G, Povey S, Ramesar R, Richards S, Seminara D, Sobrido MJ, Tavtigian S, Taylor G, Vihinen M, Winship I, Cotton RG; Human Variome Project Meeting. How to catch all those mutations—the report of the third Human Variome Project Meeting, UNESCO Paris, May 2010. Hum Mutat. 2010 Dec;31(12):1374-81.
184. Kondo N, Matsui E, Nishimura A, Kaneko H. Pharmacogenetics of asthma in children. Allergy Asthma Immunol Res. 2010 Jan;2(1):14-9.
185. Kooperberg C, LeBlanc M, Obenchain V. Risk prediction using genome-wide association studies. Genet Epidemiol. 2010 Nov;34(7):643-52.
186. Kopp JB, Winkler CA, Nelson GW. MYH9 genetic variants associated with glomerular disease: what is the role for genetic testing? Semin Nephrol. 2010 Jul;30(4):409-17.
187. Koscielny S. Why most gene expression signatures of tumors have not been useful in the clinic. Sci Transl Med. 2010 Jan 13;2(14):14ps2. **NO ACCESS**
188. Koslow SH, Williams LM, Gordon E. Personalized medicine for the brain: a call for action. Mol Psychiatry. 2010 Mar;15(3):229-30. **NO ACCESS**
189. Kota GK, Gupta P, Conyers JM, Mintz A. Targeting angiogenesis in an age of personalized medicine. Cancer Biol Ther. 2010 Nov 23;10(9):874-7. **NO ACCESS**
190. Kotliarov Y, Bozdag S, Cheng H, Wuchty S, Zenklusen JC, Fine HA. CNAReporter: a GenePattern pipeline for the generation of clinical reports of genomic alterations. BMC Med Genomics. 2010 Apr 9;3:11.
191. Kouris I, Tsirmpas C, Mougiakakou SG, Iliopoulou D, Koutsouris D. E-Health towards ecumenical framework for personalized medicine via Decision Support System. Conf Proc IEEE Eng Med Biol Soc. 2010;2010:2881-5. **NO ACCESS**
192. Krishnan KM. Biomedical Nanomagnetics: A Spin Through Possibilities in Imaging, Diagnostics, and Therapy. IEEE Trans Magn. 2010 Jul 1;46(7):2523-2558.
193. Kroemer HK, Meyer zu Schwabedissen HE. A piece in the puzzle of personalized medicine. Clin Pharmacol Ther. 2010 Jan;87(1):19-20. Review.
194. Kucherlapati R. Personalized medicine for non-small-cell lung cancer. Oncology (Williston Park). 2010 Apr 30;24(5):399-400.
195. Kularatne SA, Venkatesh C, Santhapuram HK, Wang K, Vaitilingam B, Henne WA, Low PS. Synthesis and biological analysis of prostate-specific membrane antigen-targeted anticancer prodrugs. J Med Chem. 2010 Nov 11;53(21):7767-77. **NO ACCESS**
196. Kurtz TW. Genome-wide association studies will unlock the genetic basis of hypertension.: con side of the argument. Hypertension. 2010 Dec;56(6):1021-5. **NO ACCESS**
197. Kussmann M, Panchaud A, Affolter M. Proteomics in nutrition: status quo and outlook for biomarkers and bioactives. J Proteome Res. 2010 Oct 1;9(10):4876-87. Review. **NO ACCESS**
198. Lackner MR. Prospects for personalized medicine with inhibitors targeting the RAS and PI3K pathways. Expert Rev Mol Diagn. 2010 Jan;10(1):75-87. Review. **NO ACCESS**
199. Laksman Z, Detsky AS. Personalized medicine: understanding probabilities and managing expectations. J Gen Intern Med. 2011 Feb;26(2):204-6. Epub 2010 Sep 28. **NO ACCESS**
200. Lammers T, Kiessling F, Hennink WE, Storm G. Nanotheranostics and image-guided drug delivery: current concepts and future directions. Mol Pharm. 2010 Dec 6;7(6):1899-912. Epub 2010 Oct 6. Review. **NO ACCESS**
201. Lampreabe I, Gainza de los Rios FJ, Arrieta Gutiérrez A, Jofre-Monseny L, Rodriguez M, Amenabar Iribar JJ, Zárraga Larrondo S, Tejedor D, Martinez A, Olano-Martin E. Toward personalized medicine in renal transplantation. Transplant Proc. 2010 Oct;42(8):2864-7. **NO ACCESS**
202. Landau R, Kraft JC, Flint LY, Carvalho B, Richebé P, Cardoso M, Lavand'homme P, Granot M, Yarnitsky D, Cahana A. An experimental paradigm for the prediction of Post-Operative Pain (PPOP). J Vis Exp. 2010 Jan 27;(35). pii: 1671. doi: 10.3791/1671.
203. Landriscina M, Amoroso MR, Piscazzi A, Esposito F. Heat shock proteins, cell survival and drug resistance: the mitochondrial chaperone TRAP1, a potential novel target for ovarian cancer therapy. Gynecol Oncol. 2010 May;117(2):177-82. Epub 2009 Nov 25. Review.
204. Lang E. In Cereo and in Silico: Tissue Microarray (TMA) Techniques and Bioinformatics Are Thriving Forces in Medical Science and Personalized Medicine. Yearb Med Inform. 2010:75-81. **NO ACCESS**
205. Langer A. A systematic review of PET and PET/CT in oncology: a way to personalize cancer treatment in a cost-effective manner? BMC Health Serv Res. 2010 Oct 8;10:283. Review.
206. Lawson MH, Rassl DM, Cummings NM, Russell R, Morjaria JB, Brenton JD, Murphy G, Rintoul RC. Tissue banking of diagnostic lung cancer biopsies for extraction of high quality RNA. J Thorac Oncol. 2010 Jul;5(7):956-63. **NO ACCESS**
207. Lebeda FJ, Cer RZ, Stephens RM, Mudunuri U. Temporal characteristics of botulinum neurotoxin therapy. Expert Rev Neurother. 2010 Jan;10(1):93-103. Review. **NO ACCESS**
208. Leboyer M, Kupfer DJ. Bipolar disorder: new perspectives in health care and prevention. J Clin Psychiatry. 2010 Dec;71(12):1689-95. Review. **NO ACCESS**
209. Lee HC, Lai CK, Siu TS, Yuen YP, Chan KY, Chan AY, Tam S, Mak CM, Lam CW. Role of postmortem genetic testing demonstrated in a case of glutaric aciduria type II. Diagn Mol Pathol. 2010 Sep;19(3):184-6.
210. Leeder JS, Lantos J, Spielberg SP. Conference scene: pediatric pharmacogenomics and personalized medicine. Pharmacogenomics. 2010 Dec;11(12):1691-702. **NO ACCESS**
211. Leong TY, Cooper K, Leong AS. Immunohistology--past, present, and future. Adv Anat Pathol. 2010 Nov;17(6):404-18. Review. **NO ACCESS**
212. Leuchter AF, Cook IA, Hamilton SP, Narr KL, Toga A, Hunter AM, Faull K, Whitelegge J, Andrews AM, Loo J, Way B, Nelson SF, Horvath S, Lebowitz BD. Biomarkers to predict antidepressant response. Curr Psychiatry Rep. 2010 Dec;12(6):553-62. Review.
213. Levenson D. Personalized medicine presents challenges and opportunities. Am J Med Genet A. 2010 Feb;152A(2):vii-viii.
214. Li A, Bozdag S, Kotliarov Y, Fine HA. GliomaPredict: a clinically useful tool for assigning glioma patients to specific molecular subtypes. BMC Med Inform Decis Mak. 2010 Jul 15;10:38.
215. Li J, Makrigiorgos GM. s-RT-MELT: a novel technology for mutation screening. Methods Mol Biol. 2010;653:207-19. Review. **NO ACCESS**
216. Li NY, Abbott KV, Rosen C, An G, Hebda PA, Vodovotz Y. Translational systems biology and voice pathophysiology. Laryngoscope. 2010 Mar;120(3):511-5. Review.
217. Liaw ST. Genetics and genomics in general practice. Aust Fam Physician. 2010 Sep;39(9):689-91. Review.
218. Licastro F, Caruso C. Predictive diagnostics and personalized medicine for the prevention of chronic degenerative diseases. Immun Ageing. 2010 Dec 16;7 Suppl 1:S1.
219. Lim JE, Hong KW, Jin HS, Kim YS, Park HK, Oh B. Type 2 diabetes genetic association database manually curated for the study design and odds ratio. BMC Med Inform Decis Mak. 2010 Dec 30;10:76.
220. Limaye N. Data management Redefined. Perspect Clin Res. 2010 Jul;1(3):110-2.
221. Lindberg J, Wijbrandts CA, van Baarsen LG, Nader G, Klareskog L, Catrina A, Thurlings R, Vervoordeldonk M, Lundeberg J, Tak PP. The gene expression profile in the synovium as a predictor of the clinical response to infliximab treatment in rheumatoid arthritis. PLoS One. 2010 Jun 25;5(6):e11310.
222. Litton G, Kane D, Clay G, Kruger P, Belnap T, Parkinson B. Multidisciplinary cancer care with a patient and physician satisfaction focus. J Oncol Pract. 2010 Nov;6(6):e35-7.
223. Liu L, Mason RP. Imaging beta-galactosidase activity in human tumor xenografts and transgenic mice using a chemiluminescent substrate. PLoS One. 2010 Aug 6;5(8):e12024.
224. Liu Y, Solomon M, Achilefu S. Perspectives and potential applications of nanomedicine in breast and prostate cancer. Med Res Rev. 2010 Nov 9. **NO ACCESS**
225. Lloret Linares C, Hajj A, Poitou C, Simoneau G, Clement K, Laplanche JL, Lépine JP, Bergmann JF, Mouly S, Peoc'h K. Pilot Study Examining the Frequency of Several Gene Polymorphisms Involved in Morphine Pharmacodynamics and Pharmacokinetics in a Morbidly Obese Population. Obes Surg. 2010 Apr 22. **NO ACCESS**
226. López-Casas PP, López-Fernández LA. Gene-expression profiling in pancreatic cancer. Expert Rev Mol Diagn. 2010 Jul;10(5):591-601. Review. **NO ACCESS**
227. Loukides G, Gkoulalas-Divanis A, Malin B. Anonymization of electronic medical records for validating genome-wide association studies. Proc Natl Acad Sci U S A. 2010 Apr 27;107(17):7898-903.
228. Lubitz SA, Ozcan C, Magnani JW, Kääb S, Benjamin EJ, Ellinor PT. Genetics of atrial fibrillation: implications for future research directions and personalized medicine. Circ Arrhythm Electrophysiol. 2010 Jun 1;3(3):291-9. Review.
229. Lucas JM. Microarrays: Molecular allergology and nanotechnology for personalised medicine (II). Allergol Immunopathol (Madr). 2010 Jul-Aug;38(4):217-23.
230. Lucas JM. Microarrays: molecular allergology and nanotechnology for personalised medicine (I). Allergol Immunopathol (Madr). 2010 May-Jun;38(3):153-61.
231. Mönter N. [When guidelines are confronted with health care reality: purpose of guidelines from the perspective of a psychiatrist]. Nervenarzt. 2010 Sep;81(9):1069-78. doi: 10.1007/s00115-010-3087-4. German.
232. MacDonald DJ, Blazer KR, Weitzel JN. Extending comprehensive cancer center expertise in clinical cancer genetics and genomics to diverse communities: the power of partnership. J Natl Compr Canc Netw. 2010 May;8(5):615-24. **NO ACCESS**
233. Mackoff RL, Iverson EF, Kiekel P, Dorey F, Upperman JS, Metzenberg AB. Attitudes of genetic counselors towards genetic susceptibility testing in children. J Genet Couns. 2010 Aug;19(4):402-16. **NO ACCESS**
234. Maggi N, Arrigo P, Ruggiero C. SNP analysis of Rac1 For personalized ligand interaction. Conf Proc IEEE Eng Med Biol Soc. 2010;2010:1779-82. **NO ACCESS**
235. Mahapatra A. Lung cancer - genomics and personalized medicine. ACS Chem Biol. 2010 Jun 18;5(6):529-31. **NO ACCESS**
236. Malik NN, Khan Y. Personalized medicine: potential impact on the biopharmaceutical industry. Drug Discov Today. 2010 Nov;15(21-22):881-3. **NO ACCESS**
237. Malinowsky K, Wolff C, Ergin B, Berg D, Becker KF. Deciphering signalling pathways in clinical tissues for personalized medicine using protein microarrays. J Cell Physiol. 2010 Nov;225(2):364-70. Review. **NO ACCESS**
238. Manley GT, Diaz-Arrastia R, Brophy M, Engel D, Goodman C, Gwinn K, Veenstra TD, Ling G, Ottens AK, Tortella F, Hayes RL. Common data elements for traumatic brain injury: recommendations from the biospecimens and biomarkers working group. Arch Phys Med Rehabil. 2010 Nov;91(11):1667-72. Review. **NO ACCESS**
239. Mann K, Hermann D. Individualised treatment in alcohol-dependent patients. Eur Arch Psychiatry Clin Neurosci. 2010 Nov;260 Suppl 2:S116-20.
240. Manne U, Shanmugam C, Katkoori VR, Bumpers HL, Grizzle WE. Development and progression of colorectal neoplasia. Cancer Biomark. 2010;9(1-6):235-65. Review. **NO ACCESS**
241. Mansell JL, Tierney RT, Driban JB, Clegg SM, Higgins MJ, Mishra AK, Krynetskiy E. Genetic variation and individualized medicine. J Sport Rehabil. 2010 Nov;19(4):389-98. **NO ACCESS**
242. McCabe ER. Inborn Errors of Metabolism: the metabolome is our world. Presidential address for the 11th International Congress of Inborn Errors of Metabolism (ICIEM). Mol Genet Metab. 2010 May;100(1):1-5.
243. McCabe ER. Nanopediatrics: enabling personalized medicine for children. Pediatr Res. 2010 May;67(5):453-7. Review.
244. McCaughan F, Dear PH. Single-molecule genomics. J Pathol. 2010 Jan;220(2):297-306. **NO ACCESS**
245. Melhem N, Devlin B. Shedding new light on genetic dark matter. Genome Med. 2010 Oct 21;2(10):79. **NO ACCESS**
246. Mendoza MC. HIM and the path to personalized medicine. J AHIMA. 2010 Nov-Dec;81(11):38-42; quiz 43. **NO ACCESS**
247. Mesko B, Poliska S, Szegedi A, Szekanecz Z, Palatka K, Papp M, Nagy L. Peripheral blood gene expression patterns discriminate among chronic inflammatory diseases and healthy controls and identify novel targets. BMC Med Genomics. 2010 May 5;3:15.
248. Meslin EM, Cho MK. Research ethics in the era of personalized medicine: updating science's contract with society. Public Health Genomics. 2010;13(6):378-84.
249. Meyers DA. Genetics of asthma and allergy: what have we learned? J Allergy Clin Immunol. 2010 Sep;126(3):439-46; quiz 447-8. Review.
250. Mi Q, Li NY, Ziraldo C, Ghuma A, Mikheev M, Squires R, Okonkwo DO, Verdolini-Abbott K, Constantine G, An G, Vodovotz Y. Translational systems biology of inflammation: potential applications to personalized medicine. Per Med. 2010 Sep 1;7(5):549-559.
251. Miki Y. Gene expression-based diagnosis of efficacy of chemotherapy for breast cancer. Breast Cancer. 2010 Apr;17(2):97-102. **NO ACCESS**
252. Miller VM. Sex-based differences in vascular function. Womens Health (Lond Engl). 2010 Sep;6(5):737-52. Review.
253. Milletti F, Vulpetti A. Predicting polypharmacology by binding site similarity: from kinases to the protein universe. J Chem Inf Model. 2010 Aug 23;50(8):1418-31. **NO ACCESS**
254. Misra S, Agrawal A, Liao WK, Choudhary A. Anatomy of a hash-based long read sequence mapping algorithm for next generation DNA sequencing. Bioinformatics. 2011 Jan 15;27(2):189-95. **NO ACCESS**
255. Mitri Z, Esmerian MO, Simaan JA, Sabra R, Zgheib NK. Pharmacogenetics and personalized medicine: the future for drug prescribing. J Med Liban. 2010 Apr-Jun;58(2):101-4. **NO ACCESS**
256. Mok TS, Zhou Q, Leung L, Loong HH. Personalized medicine for non-small-cell lung cancer. Expert Rev Anticancer Ther. 2010 Oct;10(10):1601-11. Review. **NO ACCESS**
257. Möller HJ, Rujescu D. Pharmacogenetics--genomics and personalized psychiatry. Eur Psychiatry. 2010 Jun;25(5):291-3. Epub 2010 Apr 13. Review.
258. Montagnana M, Favaloro EJ, Franchini M, Guidi GC, Lippi G. The role of ethnicity, age and gender in venous thromboembolism. J Thromb Thrombolysis. 2010 May;29(4):489-96. Review.
259. Monzon FA, Dumur CI. Diagnosis of uncertain primary tumors with the Pathwork tissue-of-origin test. Expert Rev Mol Diagn. 2010 Jan;10(1):17-25. Review. **NO ACCESS**
260. Monzon FA, Koen TJ. Diagnosis of metastatic neoplasms: molecular approaches for identification of tissue of origin. Arch Pathol Lab Med. 2010 Feb;134(2):216-24. Review.
261. Morère JF. Personalized medicine in lung adenocarcinoma: no longer a hope or a passing fashion, but a new reality. Target Oncol. 2010 Dec;5(4):229-30. **NO ACCESS**
262. Mosher CM, Court MH. Comparative and veterinary pharmacogenomics. Handb Exp Pharmacol. 2010;(199):49-77. Review. **NO ACCESS**
263. Mukherjee K, Chakravarthy AB, Goff LW, El-Rifai W. Esophageal adenocarcinoma: treatment modalities in the era of targeted therapy. Dig Dis Sci. 2010 Dec;55(12):3304-14. Epub 2010 Mar 19. Review. **NO ACCESS**
264. Mullaney JM, Mills RE, Pittard WS, Devine SE. Small insertions and deletions (INDELs) in human genomes. Hum Mol Genet. 2010 Oct 15;19(R2):R131-6. Epub 2010 Sep 21. Review. **NO ACCESS**
265. Muller G. Oral Delivery of Protein Drugs: Driver for Personalized Medicine. Curr Issues Mol Biol. 2010 Jul 30;13(1):13-24.
266. Mullis PE. Biological determinants of responsiveness to growth hormone: pharmacogenomics and personalized medicine. Endocr Dev. 2010;18:67-82. Epub 2010 Jun 3. Review. **NO ACCESS**
267. Munshi A, Kaul S. Genetic basis of stroke: an overview. Neurol India. 2010 Mar-Apr;58(2):185-90. doi: 10.4103/0028-3886.63780. Review. **NO ACCESS**
268. Nair RR, Tolentino J, Hazlehurst LA. The bone marrow microenvironment as a sanctuary for minimal residual disease in CML. Biochem Pharmacol. 2010 Sep 1;80(5):602-12. Epub 2010 Apr 9. Review.
269. Nair SR. Personalized medicine: Striding from genes to medicines. Perspect Clin Res. 2010 Oct;1(4):146-50.
270. Nakamura N, Fukuda T, Nonen S, Hashimoto K, Azuma J, Gemma N. Simple and accurate determination of CYP2D6 gene copy number by a loop-mediated isothermal amplification method and an electrochemical DNA chip. Clin Chim Acta. 2010 Apr 2;411(7-8):568-73. **NO ACCESS**
271. Narod SA. Personalized medicine: a personal view. Curr Oncol. 2010 Oct;17(5):4-5.
272. Nathanson KL. Using genetics and genomics strategies to personalize therapy for cancer: focus on melanoma. Biochem Pharmacol. 2010 Sep 1;80(5):755-61. Epub 2010 Apr 20. Review.
273. Naylor S, Chen JY. Unraveling human complexity and disease with systems biology and personalized medicine. Per Med. 2010 May;7(3):275-289.
274. Nguyen DX. Tracing the origins of metastasis. J Pathol. 2010 Oct 6. **NO ACCESS**
275. Niederlag W, Lemke HU, Rienhoff O. [Personalized medicine and individual healthcare : Medical and information technology aspects]. Bundesgesundheitsblatt Gesundheitsforschung Gesundheitsschutz. 2010 Aug;53(8):776-82. Review. German.
276. Nijwening JH, Beijersbergen RL. Using large-scale RNAi screens to identify novel drug targets for cancer. IDrugs. 2010 Nov;13(11):772-7. Review. **NO ACCESS**
277. Nishiyama M. [Personalized medicine and molecular targets of drugs]. Nippon Rinsho. 2010 Oct;68(10):1917-22. Review. Japanese.
278. Novack GD. Personalized medicine and the ocular surface. Ocul Surf. 2010 Jul;8(3):157-9. **NO ACCESS**
279. Novelli G. Personalized genomic medicine. Intern Emerg Med. 2010 Oct;5 Suppl 1:S81-90. **NO ACCESS**
280. Nunes T, Rocha JF, Vaz-da-Silva M, Igreja B, Wright LC, Falcão A, Almeida L, Soares-da-Silva P. Safety, Tolerability, and Pharmacokinetics of Etamicastat, a Novel Dopamine-β-Hydroxylase Inhibitor, in a Rising Multiple-Dose Study in Young Healthy Subjects. Drugs R D. 2010;10(4):225-42. doi: 10.2165/11586310-000000000-00000. **NO ACCESS**
281. Oberg K. Pancreatic endocrine tumors. Semin Oncol. 2010 Dec;37(6):594-618. Review.
282. Okamoto I, Mitsudomi T, Nakagawa K, Fukuoka M. The emerging role of epidermal growth factor receptor (EGFR) inhibitors in first-line treatment for patientswith advanced non-small cell lung cancer positive for EGFR mutations. Ther Adv Med Oncol. 2010 Sep;2(5):301-7.
283. Opal SM. New perspectives on immunomodulatory therapy for bacteraemia and sepsis. Int J Antimicrob Agents. 2010 Dec;36 Suppl 2:S70-3.
284. Osborn EA, Jaffer FA. The year in molecular imaging. JACC Cardiovasc Imaging. 2010 Nov;3(11):1181-95. Review. **NO ACCESS**
285. Overby CL, Tarczy-Hornoch P, Hoath JI, Kalet IJ, Veenstra DL. Feasibility of incorporating genomic knowledge into electronic medical records for pharmacogenomic clinical decision support. BMC Bioinformatics. 2010 Oct 28;11 Suppl 9:S10.
286. Ozdemir V, Armengaud J, Dubé L, Aziz RK, Knoppers BM. Nutriproteomics and Proteogenomics: Cultivating Two Novel Hybrid Fields of Personalized Medicine with Added Societal Value. Curr Pharmacogenomics Person Med. 2010 Dec 1;8(4):240-244. **NO ACCESS**
287. Pal SK, Hurria A. Impact of age, sex, and comorbidity on cancer therapy and disease progression. J Clin Oncol. 2010 Sep 10;28(26):4086-93. Epub 2010 Jul 19. Review.
288. Palicka V. [Personalized medicine--reality, myth, fiction?]. Cas Lek Cesk. 2010;149(10):460-1. Czech.
289. Pan D, Carauthers SD, Chen J, Winter PM, SenPan A, Schmieder AH, Wickline SA, Lanza GM. Nanomedicine strategies for molecular targets with MRI and optical imaging. Future Med Chem. 2010 Mar;2(3):471-90. Review.
290. Pappalardo F, Pennisi M, Castiglione F, Motta S. Vaccine protocols optimization: in silico experiences. Biotechnol Adv. 2010 Jan-Feb;28(1):82-93.
291. Parikh K, Peppelenbosch MP. Kinome profiling of clinical cancer specimens. Cancer Res. 2010 Apr 1;70(7):2575-8.
292. Park H, Kim JI, Ju YS, Gokcumen O, Mills RE, Kim S, Lee S, Suh D, Hong D, Kang HP, Yoo YJ, Shin JY, Kim HJ, Yavartanoo M, Chang YW, Ha JS, Chong W, Hwang GR, Darvishi K, Kim H, Yang SJ, Yang KS, Kim H, Hurles ME, Scherer SW, Carter NP, Tyler-Smith C, Lee C, Seo JS. Discovery of common Asian copy number variants using integrated high-resolution array CGH and massively parallel DNA sequencing. Nat Genet. 2010 May;42(5):400-5.
293. Parsons BL, Myers MB, Meng F, Wang Y, McKinzie PB. Oncomutations as biomarkers of cancer risk. Environ Mol Mutagen. 2010 Oct-Dec;51(8-9):836-50. Review.
294. Parthasarathy J, Starly B, Raman S, Christensen A. Mechanical evaluation of porous titanium (Ti6Al4V) structures with electron beam melting (EBM). J Mech Behav Biomed Mater. 2010 Apr;3(3):249-59. **NO ACCESS**
295. Pascual V, Chaussabel D, Banchereau J. A genomic approach to human autoimmune diseases. Annu Rev Immunol. 2010 Mar;28:535-71. Review.
296. Pasinetti GM, Fivecoat H, Ho L. Personalized medicine in traumatic brain injury. Psychiatr Clin North Am. 2010 Dec;33(4):905-13. Review. **NO ACCESS**
297. Pasini E, Pirelli S, Di Lenarda A, Dioguardi FS, Maseri A. [The evolution of medical research: from trials to tailored therapy]. G Ital Cardiol (Rome). 2010 Oct;11(10):761-6. Review. Italian.
298. Patel SA, King CC, Lim PK, Habiba U, Dave M, Porecha R, Rameshwar P. Personalizing Stem Cell Research and Therapy: The Arduous Road Ahead or Missed Opportunity? Curr Pharmacogenomics Person Med. 2010 Mar 1;8(1):25-36.
299. Patel SD, Le-Niculescu H, Koller DL, Green SD, Lahiri DK, McMahon FJ, Nurnberger JI Jr, Niculescu AB 3rd. Coming to grips with complex disorders: genetic risk prediction in bipolar disorder using panels of genes identified through convergent functional genomics. Am J Med Genet B Neuropsychiatr Genet. 2010 Jun 5;153B(4):850-77.
300. Patrinos GP, Innocenti F. Pharmacogenomics: paving the path to personalized medicine. Pharmacogenomics. 2010 Feb;11(2):141-6. **NO ACCESS**
301. Peña AS. Personalized medicine: inevitable. Rev Esp Enferm Dig. 2010 Oct;102(10):573-6. **NO ACCESS**
302. Peters BJ, Rodin AS, de Boer A, Maitland-van der Zee AH. Methodological and statistical issues in pharmacogenomics. J Pharm Pharmacol. 2010 Feb;62(2):161-6.
303. Piersma SR, Labots M, Verheul HM, Jiménez CR. Strategies for kinome profiling in cancer and potential clinical applications: chemical proteomics and array-based methods. Anal Bioanal Chem. 2010 Aug;397(8):3163-71. Epub 2010 Jun 8. Review.
304. Piwnica-Worms D, Sharma V. Probing multidrug resistance P-glycoprotein transporter activity with SPECT radiopharmaceuticals. Curr Top Med Chem. 2010;10(17):1834-45. Review. **NO ACCESS**
305. Plenge RM, Bridges SL Jr. Personalized medicine in rheumatoid arthritis: Miles to go before we sleep. Arthritis Rheum. 2010 Nov 4. **NO ACCESS**
306. Potti A, Schilsky RL, Nevins JR. Refocusing the war on cancer: the critical role of personalized treatment. Sci Transl Med. 2010 Apr 21;2(28):28cm13.
307. Prebula RJ. The promise of personalized medicine: regulatory controls and tort influences in the context of personalized risks and benefits. J Contemp Health Law Policy. 2010 Spring;26(2):343-72. **NO ACCESS**
308. Price M, Monteiro AN. Fine tuning chemotherapy to match BRCA1 status. Biochem Pharmacol. 2010 Sep 1;80(5):647-53. Epub 2010 May 25. Review. PubMed
309. Pride DT, Sun CL, Salzman J, Rao N, Loomer P, Armitage GC, Banfield JF, Relman DA. Analysis of streptococcal CRISPRs from human saliva reveals substantial sequence diversity within and between subjects over time. Genome Res. 2011 Jan;21(1):126-36.
310. Pysz MA, Gambhir SS, Willmann JK. Molecular imaging: current status and emerging strategies. Clin Radiol. 2010 Jul;65(7):500-16. Review.
311. Quinn B. Payers and the assessment of clinical utility for companion diagnostics. Clin Pharmacol Ther. 2010 Dec;88(6):751-4. Review.
312. Raaijmakers JA, Koster ES, Maitland-van der Zee AH. Pharmacogenetics and the pharmaceutical industry. Curr Pharm Des. 2010;16(2):238-44. Review. **NO ACCESS**
313. Rai AJ, Yee J, Fleisher M. Biomarkers in the era of personalized medicine - a multiplexed SNP assay using capillary electrophoresis for assessing drug metabolism capacity. Scand J Clin Lab Invest Suppl. 2010 Jul;242:15-8.
314. Rai P, Mallidi S, Zheng X, Rahmanzadeh R, Mir Y, Elrington S, Khurshid A, Hasan T. Development and applications of photo-triggered theranostic agents. Adv Drug Deliv Rev. 2010 Aug 30;62(11):1094-124. Epub 2010 Sep 19. Review. **NO ACCESS**
315. Rajkumar T. Personalized medicine: FAQs. Indian J Med Paediatr Oncol. 2010 Apr;31(2):72-4.
316. Rauser S, Deininger SO, Suckau D, Höfler H, Walch A. Approaching MALDI molecular imaging for clinical proteomic research: current state and fields of application. Expert Rev Proteomics. 2010 Dec;7(6):927-41. Review. **NO ACCESS**
317. Ravdin JI. Personalized medicine brings genetic knowledge to bear. WMJ. 2010 Oct;109(5):289-90. **NO ACCESS**
318. Raynal C, Ciccolini J, Mercier C, Boyer JC, Polge A, Lallemant B, Mouzat K, Lumbroso S, Brouillet JP, Evrard A. High-resolution melting analysis of sequence variations in the cytidine deaminase gene (CDA) in patients with cancer treated with gemcitabine. Ther Drug Monit. 2010 Feb;32(1):53-60.
319. Recht A. Radiotherapy, antihormonal therapy, and personalised medicine. Lancet Oncol. 2010 Mar;11(3):215-6.
320. Ren J, Jiang C, Gao X, Liu Z, Yuan Z, Jin C, Wen L, Zhang Z, Xue Y, Yao X. PhosSNP for systematic analysis of genetic polymorphisms that influence protein phosphorylation. Mol Cell Proteomics. 2010 Apr;9(4):623-34.
321. Richards ML. Familial syndromes associated with thyroid cancer in the era of personalized medicine. Thyroid. 2010 Jul;20(7):707-13. Review.
322. Riklund KA. PET/CT: nuclear medicine imaging in the future. Radiat Prot Dosimetry. 2010 Apr-May;139(1-3):8-11.
323. Roberts PJ, Stinchcombe TE, Der CJ, Socinski MA. Personalized medicine in non-small-cell lung cancer: is KRAS a useful marker in selecting patients for epidermal growth factor receptor-targeted therapy? J Clin Oncol. 2010 Nov 1;28(31):4769-77. **NO ACCESS**
324. Rocca B, Petrucci G. Personalized medicine, pharmacogenetics, and clopidogrel: unraveling variability of response. Mol Interv. 2010 Feb;10(1):12-9.
325. Rodriguez H, Rivers R, Kinsinger C, Mesri M, Hiltke T, Rahbar A, Boja E. Reconstructing the pipeline by introducing multiplexed multiple reaction monitoring mass spectrometry for cancer biomarker verification: an NCI-CPTC initiative perspective. Proteomics Clin Appl. 2010 Dec;4(12):904-14. doi: 10.1002/prca.201000057. Review. **NO ACCESS**
326. Romanelli MG, Pressi L, Regele M, Castellan C. [Genomics in medicine: challenges and perspectives for the nursing care]. Assist Inferm Ric. 2010 Apr-Jun;29(2):62-8. Italian.
327. Roukos DH. Bionetworks-based personalized medicine versus comparative-effectiveness research or harmonization of both in cancer management? Expert Rev Mol Diagn. 2010 Apr;10(3):247-50.
328. Roukos DH. Novel clinico-genome network modeling for revolutionizing genotype-phenotype-based personalized cancer care. Expert Rev Mol Diagn. 2010 Jan;10(1):33-48. Review. **NO ACCESS**
329. Rountree CB, Van Kirk CA, You H, Ding W, Dang H, VanGuilder HD, Freeman WM. Clinical application for the preservation of phospho-proteins through in-situ tissue stabilization. Proteome Sci. 2010 Nov 22;8:61.
330. Ruan Y, Wei CL. Multiplex parallel pair-end-ditag sequencing approaches in system biology. Wiley Interdiscip Rev Syst Biol Med. 2010 Mar-Apr;2(2):224-34. Review.
331. Saito M, Yoshino T. [Clinical development of biomarkers for personalized medicine]. Nippon Rinsho. 2010 Jun;68(6):1111-6. Review. Japanese.
332. Sakamoto JH, van de Ven AL, Godin B, Blanco E, Serda RE, Grattoni A, Ziemys A, Bouamrani A, Hu T, Ranganathan SI, De Rosa E, Martinez JO, Smid CA, Buchanan RM, Lee SY, Srinivasan S, Landry M, Meyn A, Tasciotti E, Liu X, Decuzzi P, Ferrari M. Enabling individualized therapy through nanotechnology. Pharmacol Res. 2010 Aug;62(2):57-89. Epub 2010 Jan 5. Review.
333. Salvadore G, Zarate CA Jr. Magnetic resonance spectroscopy studies of the glutamatergic system in mood disorders: a pathway to diagnosis, novel therapeutics, and personalized medicine? Biol Psychiatry. 2010 Nov 1;68(9):780-2.
334. Samani NJ, Tomaszewski M, Schunkert H. The personal genome--the future of personalised medicine? Lancet. 2010 May 1;375(9725):1497-8.
335. Saminathan R, Bai J, Sadrolodabaee L, Karthik GM, Singh O, Subramaniyan K, Ching CB, Chen WN, Chowbay B. VKORC1 pharmacogenetics and pharmacoproteomics in patients on warfarin anticoagulant therapy: transthyretin precursor as a potential biomarker. PLoS One. 2010 Dec 13;5(12):e15064.
336. Sato Y, Laird NM, Yoshida T. Biostatistic tools in pharmacogenomics--advances, challenges, potential. Curr Pharm Des. 2010;16(20):2232-40. Review.
337. Savas S. Useful genetic variation databases for oncologists investigating the genetic basis of variable treatment response and survival in cancer. Acta Oncol. 2010 Nov;49(8):1217-26.
338. Schilsky RL. Personalized medicine in oncology: the future is now. Nat Rev Drug Discov. 2010 May;9(5):363-6.
339. Schirmer SH, Hohl M, Böhm M. Gender differences in heart failure: paving the way towards personalized medicine? Eur Heart J. 2010 May;31(10):1165-7.
340. Shabo Shvo A. Meaningful use of pharmacogenomics in health records: semantics should be made explicit. Pharmacogenomics. 2010 Jan;11(1):81-7. Review. **NO ACCESS**
341. Sharon D, Chen R, Snyder M. Systems biology approaches to disease marker discovery. Dis Markers. 2010;28(4):209-24. Review.
342. Shastry BS. Genetic diversity and medicinal drug response in eye care. Graefes Arch Clin Exp Ophthalmol. 2010 Aug;248(8):1057-61. Epub 2010 Mar 5. Review.
343. Simon GE, Perlis RH. Personalized medicine for depression: can we match patients with treatments? Am J Psychiatry. 2010 Dec;167(12):1445-55. Epub 2010 Sep 15. Review. **NO ACCESS**
344. Sitaraman R. Impact of personalized medicine on diagnostics and therapeutics. Natl Med J India. 2010 Mar-Apr;23(2):124. **NO ACCESS**
345. Sivapackiam J, Gammon ST, Harpstrite SE, Sharma V. Targeted chemotherapy in drug-resistant tumors, noninvasive imaging of P-glycoprotein-mediated functional transport in cancer, and emerging role of Pgp in neurodegenerative diseases Methods Mol Biol. 2010;596:141-81. **NO ACCESS**
346. Slanar O, Zima T. [Pharmacogenetic aspects of current pharmacotherapy]. Cas Lek Cesk. 2010;149(10):472-5. Review. Czech.
347. Smith FO. Personalized medicine for AML? Blood. 2010 Oct 14;116(15):2622-3.
348. Smith HS. The role of genomic oxidative-reductive balance as predictor of complex regional pain syndrome development: a novel theory. Pain Physician. 2010 Jan-Feb;13(1):79-90. Review.
349. Smulders YM, Levi M, Stehouwer CD, Kramer MH, Thijs A. [The role of epidemiological evidence in providing care for individual patients]. Ned Tijdschr Geneeskd. 2010;154:A1910. Dutch.
350. Sone S, Otsuka K, Tobiume M, Tada H. [Historical aspect of molecular-targeted therapy for cancer]. Nippon Rinsho. 2010 Oct;68(10):1787-95. Japanese.
351. Squassina A, Manchia M, Manolopoulos VG, Artac M, Lappa-Manakou C, Karkabouna S, Mitropoulos K, Del Zompo M, Patrinos GP. Realities and expectations of pharmacogenomics and personalized medicine: impact of translating genetic knowledge into clinical practice. Pharmacogenomics. 2010 Aug;11(8):1149-67. Review. **NO ACCESS**
352. Staels B. Fibrates in CVD: a step towards personalised medicine. Lancet. 2010 May 29;375(9729):1847-8.
353. Statnikov A, Aliferis CF. Analysis and computational dissection of molecular signature multiplicity. PLoS Comput Biol. 2010 May 20;6(5):e1000790.
354. Ströbel P, Hohenberger P, Marx A. Thymoma and thymic carcinoma: molecular pathology and targeted therapy. J Thorac Oncol. 2010 Oct;5(10 Suppl 4):S286-90. Review. **NO ACCESS**
355. Stukel JM, Li RC, Maynard HD, Caplan MR. Two-step synthesis of multivalent cancer-targeting constructs. Biomacromolecules. 2010 Jan 11;11(1):160-7 **NO ACCESS**
356. Suárez-Fariñas M, Shah KR, Haider AS, Krueger JG, Lowes MA. Personalized medicine in psoriasis: developing a genomic classifier to predict histological response to Alefacept. BMC Dermatol. 2010 Feb 12;10:1.
357. Subbiah MT. Application of nutrigenomics in skin health: nutraceutical or cosmeceutical? J Clin Aesthet Dermatol. 2010 Nov;3(11):44-6.
358. Sun J, Masterman-Smith MD, Graham NA, Jiao J, Mottahedeh J, Laks DR, Ohashi M, DeJesus J, Kamei K, Lee KB, Wang H, Yu ZT, Lu YT, Hou S, Li K, Liu M, Zhang N, Wang S, Angenieux B, Panosyan E, Samuels ER, Park J, Williams D, Konkankit V, Nathanson D, van Dam RM, Phelps ME, Wu H, Liau LM, Mischel PS, Lazareff JA, Kornblum HI, Yong WH, Graeber TG, Tseng HR. A microfluidic platform for systems pathology: multiparameter single-cell signaling measurements of clinical brain tumor specimens. Cancer Res. 2010 Aug 1;70(15):6128-38.
359. Swistowski A, Peng J, Liu Q, Mali P, Rao MS, Cheng L, Zeng X. Efficient generation of functional dopaminergic neurons from human induced pluripotent stem cells under defined conditions. Stem Cells. 2010 Oct;28(10):1893-904.
360. Tacu C, Neagu M, Constantin C, Sajin M. Biomarkers discovery in cancer--up-dates in methodology. Roum Arch Microbiol Immunol. 2010 Jan-Mar;69(1):48-55. **NO ACCESS**
361. Teli MK, Mutalik S, Rajanikant GK. Nanotechnology and nanomedicine: going small means aiming big. Curr Pharm Des. 2010 Jun;16(16):1882-92. Review.
362. Terry SF. Standards for personalized medicine. Genet Test Mol Biomarkers. 2010 Apr;14(2):155-6.
363. Terzic A, Perez-Terzic C. Channelopathies: decoding disease pathogenesis. Sci Transl Med. 2010 Jul 28;2(42):42ps37. doi: 10.1126/scitranslmed.3001433. **NO ACCESS**
364. Tesch G, Amur S, Schousboe JT, Siegel JN, Lesko LJ, Bai JP. Successes achieved and challenges ahead in translating biomarkers into clinical applications. AAPS J. 2010 Sep;12(3):243-53.
365. The future of care: Part 3: An executive discussion. Hosp Health Netw. 2010 Oct;84(10):55-65, 2. **NO ACCESS**
366. Thng TG, Lim KS. Personalised medicine for psoriasis: a real possibility ahead. Ann Acad Med Singapore. 2010 Aug;39(8):588-90.
367. Thomas A, Phillips A, Donnelly R, Piech CT. Comparative effectiveness, personalized medicine and innovation: the path forward. Pharmacoeconomics. 2010;28(10):923-30. doi: 10.2165/11539030-000000000-0000010.2165/11537370-000000000-00000. **NO ACCESS**
368. Thomas DM, Wagner AJ. Specific targets in sarcoma and developmental therapeutics. J Natl Compr Canc Netw. 2010 Jun;8(6):677-85; quiz 686. **NO ACCESS**
369. Tingen CM, Kim AM, Wu PH, Woodruff TK. Sex and sensitivity: the continued need for sex-based biomedical research and implementation. Womens Health (Lond Engl). 2010 Jul;6(4):511-6. Review.
370. Trainer AH, Meiser B, Watts K, Mitchell G, Tucker K, Friedlander M. Moving toward personalized medicine: treatment-focused genetic testing of women newly diagnosed with ovarian cancer. Int J Gynecol Cancer. 2010 Jul;20(5):704-16. Review. **NO ACCESS**
371. Tromp G, Kuivaniemi H, Hinterseher I, Carey DJ. Novel genetic mechanisms for aortic aneurysms. Curr Atheroscler Rep. 2010 Jul;12(4):259-66. **NO ACCESS**
372. Trosman JR, Van Bebber SL, Phillips KA. Coverage policy development for personalized medicine: private payer perspectives on developing policy for the 21-gene assay. J Oncol Pract. 2010 Sep;6(5):238-42.
373. Trudeau MA, Wong JM. Genetic Variations in Telomere Maintenance, with Implications on Tissue Renewal Capacity and Chronic Disease Pathologies. Curr Pharmacogenomics Person Med. 2010 Mar 1;8(1):7-24.
374. Tugwell P, Knottnerus JA, Idzerda L. Clinical epidemiologists and EBM proponents need to be able to critically appraise 'Omics,' the fatal flaw of Personalized Medicine. J Clin Epidemiol. 2010 Sep;63(9):943-4. **NO ACCESS**
375. Van Bebber SL, Trosman JR, Liang SY, Wang G, Marshall DA, Knight S, Phillips KA. Capacity building for assessing new technologies: approaches to examining personalized medicine in practice. Per Med. 2010 Jul;7(4):427-439.
376. VanderWeele TJ. Genetic self knowledge and the future of epidemiologic confounding. Am J Hum Genet. 2010 Aug 13;87(2):168-72. Erratum in: Am J Hum Genet. 2010 Sep 10;87(3):446. Weele, Tyler Vander [corrected to VanderWeele, Tyler J].
377. Vasilevskis EE, Pandharipande PP, Girard TD, Ely EW. A screening, prevention, and restoration model for saving the injured brain in intensive care unit survivors. Crit Care Med. 2010 Oct;38(10 Suppl):S683-91.
378. Vaziri SA, Kim J, Ganapathi MK, Ganapathi R. Vascular endothelial growth factor polymorphisms: role in response and toxicity of tyrosine kinase inhibitors. Curr Oncol Rep. 2010 Mar;12(2):102-8. Review. **NO ACCESS**
379. Vera-Ramirez L, Sanchez-Rovira P, Ramirez-Tortosa CL, Quiles JL, Ramirez-Tortosa MC, Alvarez JC, Fernandez-Navarro M, Lorente JA. Gene-expression profiles, tumor microenvironment, and cancer stem cells in breast cancer: latest advances towards an integrated approach. Cancer Treat Rev. 2010 Oct;36(6):477-84. Epub 2010 Mar 17. Review.
380. Vermeire S, Van Assche G, Rutgeerts P. Role of genetics in prediction of disease course and response to therapy. World J Gastroenterol. 2010 Jun 7;16(21):2609-15.
381. Villagra D, Duconge J, Windemuth A, Cadilla CL, Kocherla M, Gorowski K, Bogaard K, Renta JY, Cruz IA, Mirabal S, Seip RL, Ruaño G. CYP2C9 and VKORC1 genotypes in Puerto Ricans: A case for admixture-matching in clinical pharmacogenetic studies. Clin Chim Acta. 2010 Sep 6;411(17-18):1306-11. **NO ACCESS**
382. Villanueva A, Minguez B, Forner A, Reig M, Llovet JM. Hepatocellular carcinoma: novel molecular approaches for diagnosis, prognosis, and therapy. Annu Rev Med. 2010;61:317-28. Review. **NO ACCESS**
383. Villoslada P. Biomarkers for multiple sclerosis. Drug News Perspect. 2010 Nov;23(9):585-95. Review. **NO ACCESS**
384. Visscher PM, Goddard ME. Systems genetics: the added value of gene expression. HFSP J. 2010 Feb;4(1):6-10.
385. Vodovotz Y, Constantine G, Faeder J, Mi Q, Rubin J, Bartels J, Sarkar J, Squires RH Jr, Okonkwo DO, Gerlach J, Zamora R, Luckhart S, Ermentrout B, An G. Translational systems approaches to the biology of inflammation and healing. Immunopharmacol Immunotoxicol. 2010 Jun;32(2):181-95. Review.
386. Vodovotz Y. Translational systems biology of inflammation and healing. Wound Repair Regen. 2010 Jan-Feb;18(1):3-7.
387. Vogenberg FR, Barash CI, Pursel M. Personalized medicine: part 2: ethical, legal, and regulatory issues. P T. 2010 Nov;35(11):624-42.
388. Vogenberg FR, Barash CI, Pursel M. Personalized medicine: part 3: challenges facing health care plans in implementing coverage policies for pharmacogenomic and genetic testing. P T. 2010 Dec;35(12):670-5.
389. Vogenberg FR, Isaacson Barash C, Pursel M. Personalized medicine: part 1: evolution and development into theranostics. P T. 2010 Oct;35(10):560-76.
390. Wörns MA, Galle PR. Novel inhibitors in development for hepatocellular carcinoma. Expert Opin Investig Drugs. 2010 May;19(5):615-29. Review. **NO ACCESS**
391. Walker P, Gregg AR. Screening, testing, or personalized medicine: where do inherited thrombophilias fit best? Obstet Gynecol Clin North Am. 2010 Mar;37(1):87-107, Table of Contents. **NO ACCESS**
392. Wallis D. The search for biomarkers for attention deficit/hyperactivity disorder. Drug News Perspect. 2010 Sep;23(7):438-49. Review. **NO ACCESS**
393. Wang HL, Lopategui J, Amin MB, Patterson SD. KRAS mutation testing in human cancers: The pathologist's role in the era of personalized medicine. Adv Anat Pathol. 2010 Jan;17(1):23-32. Review. **NO ACCESS**
394. Ward E, Chan E, Gustafsson K, Jayasinghe SN. Combining bio-electrospraying with gene therapy: a novel biotechnique for the delivery of genetic material via living cells. Analyst. 2010 May;135(5):1042-9.
395. Wasmuth HE, Weiskirchen R. Personalized medicine in hepatitis C: from genome-wide association studies to clinical practice. Hepatology. 2010 Jun;51(6):2223-5.
396. Watson AP, Egland KA. Pathways to personalized medicine for breast and prostate cancers: emerging diagnostic methods and prognostic biomarkers. S D Med. 2010 Jul;63(7):247-53. **NO ACCESS**
397. Weber WW. The promise of epigenetics in personalized medicine. Mol Interv. 2010 Dec;10(6):363-70. Review.
398. Weinlander KM, Hall DJ. Designing laboratory exercises for the undergraduate molecular biology/biochemistry student: Techniques and ethical implications involved in personalized medicine. Biochem Mol Biol Educ. 2010 May;38(3):180-7. doi: 10.1002/bmb.20366. **NO ACCESS**
399. Weinstein S, Peer D. RNAi nanomedicines: challenges and opportunities within the immune system. Nanotechnology. 2010 Jun 11;21(23):232001. Epub 2010 May 13. Review. **NO ACCESS**
400. Weller M, Stupp R, Reifenberger G, Brandes AA, van den Bent MJ, Wick W, Hegi ME. MGMT promoter methylation in malignant gliomas: ready for personalized medicine? Nat Rev Neurol. 2010 Jan;6(1):39-51. Review. **NO ACCESS**
401. Weller M, Wick W, Hegi ME, Stupp R, Tabatabai G. Should biomarkers be used to design personalized medicine for the treatment of glioblastoma? Future Oncol. 2010 Sep;6(9):1407-14. Review. **NO ACCESS**
402. Wender RC. Family medicine in the research revolution. J Am Board Fam Med. 2010 Jul-Aug;23(4):431-9.
403. Wening K, Breitkreutz J. Novel delivery device for monolithical solid oral dosage forms for personalized medicine. Int J Pharm. 2010 May 26. **NO ACCESS**
404. Wening K, Breitkreutz J. Novel delivery device for monolithical solid oral dosage forms for personalized medicine. Int J Pharm. 2010 Aug 16;395(1-2):174-81. **NO ACCESS**
405. Werner T. Next generation sequencing in functional genomics. Brief Bioinform. 2010 Sep;11(5):499-511. Epub 2010 May 25. Review. **NO ACCESS**
406. Wiessler M, Waldeck W, Pipkorn R, Kliem C, Lorenz P, Fleischhacker H, Hafner M, Braun K. Extension of the PNA world by functionalized PNA monomers eligible candidates for inverse Diels Alder Click Chemistry. Int J Med Sci. 2010 Jun 27;7(4):213-23.
407. Wiles MV, Taft RA. The sophisticated mouse: protecting a precious reagent. Methods Mol Biol. 2010;602:23-36. **NO ACCESS**
408. Wilke RA, Berg RL, Linneman JG, Peissig P, Starren J, Ritchie MD, McCarty CA. Quantification of the clinical modifiers impacting high-density lipoprotein cholesterol in the community: Personalized Medicine Research Project. Prev Cardiol. 2010 Spring;13(2):63-8. **NO ACCESS**
409. Wilson PM, Labonte MJ, Lenz HJ. Molecular markers in the treatment of metastatic colorectal cancer. Cancer J. 2010 May-Jun;16(3):262-72. Review. **NO ACCESS**
410. Winkelmann BR, Herrington D. Pharmacogenomics--10 years of progress: a cardiovascular perspective. Pharmacogenomics. 2010 May;11(5):613-6. Review.
411. Winkler CA, Nelson G, Oleksyk TK, Nava MB, Kopp JB. Genetics of focal segmental glomerulosclerosis and human immunodeficiency virus-associated collapsing glomerulopathy: the role of MYH9 genetic variation. Semin Nephrol. 2010 Mar;30(2):111-25. Review.
412. Winnike JH, Li Z, Wright FA, Macdonald JM, O'Connell TM, Watkins PB. Use of pharmaco-metabonomics for early prediction of acetaminophen-induced hepatotoxicity in humans. Clin Pharmacol Ther. 2010 Jul;88(1):45-51.
413. Wittkowski KM, Song T. Nonparametric methods for molecular biology. Methods Mol Biol. 2010;620:105-53.
414. Wong SH, Happy C, Blinka D, Gock S, Jentzen JM, Donald Hon J, Coleman H, Jortani SA, Lucire Y, Morris-Kukoski CL, Neuman MG, Orsulak PJ, Sander T, Wagner MA, Wynn JR, Wu AH, Yeo KT. From personalized medicine to personalized justice: the promises of translational pharmacogenomics in the justice system. Pharmacogenomics. 2010 Jun;11(6):731-7.
415. Wright CF, Kroese M. Evaluation of genetic tests for susceptibility to common complex diseases: why, when and how? Hum Genet. 2010 Feb;127(2):125-34.
416. Xie J, Chen K, Huang J, Lee S, Wang J, Gao J, Li X, Chen X. PET/NIRF/MRI triple functional iron oxide nanoparticles. Biomaterials. 2010 Apr;31(11):3016-22.
417. Xie J, Lee S, Chen X. Nanoparticle-based theranostic agents. Adv Drug Deliv Rev. 2010 Aug 30;62(11):1064-79. Epub 2010 Aug 4. Review. **NO ACCESS**
418. Xu J, Wise C, Varma V, Fang H, Ning B, Hong H, Tong W, Kaput J. Two new ArrayTrack libraries for personalized biomedical research. BMC Bioinformatics. 2010 Oct 7;11 Suppl 6:S6.
419. Yan Q. Immunoinformatics and systems biology methods for personalized medicine. Methods Mol Biol. 2010;662:203-20. Review. **NO ACCESS**
420. Yan Q. Membrane transporters and drug development: relevance to pharmacogenomics, nutrigenomics, epigenetics, and systems biology. Methods Mol Biol. 2010;637:1-21. **NO ACCESS**
421. Yan Q. Translational bioinformatics and systems biology approaches for personalized medicine. Methods Mol Biol. 2010;662:167-78. **NO ACCESS**
422. Yang JY, Niemierko A, Bajcsy R, Xu D, Athey BD, Zhang A, Ersoy OK, Li GZ, Borodovsky M, Zhang JC, Arabnia HR, Deng Y, Dunker AK, Liu Y, Ghafoor A. 2K09 and thereafter : the coming era of integrative bioinformatics, systems biology and intelligent computing for functional genomics and personalized medicine research. BMC Genomics. 2010 Dec 1;11 Suppl 3:I1
423. Yang S, Xu L, Wu HM. Rapid genotyping of single nucleotide polymorphisms influencing warfarin drug response by surface-enhanced laser desorption and ionization time-of-flight (SELDI-TOF) mass spectrometry. J Mol Diagn. 2010 Mar;12(2):162-8.
424. Yang S, Xu L, Wu HM. Rapid multiplexed genotyping for hereditary thrombophilia by SELDI-TOF mass spectrometry. Diagn Mol Pathol. 2010 Mar;19(1):54-61.
425. Yang X, Grailer JJ, Rowland IJ, Javadi A, Hurley SA, Steeber DA, Gong S. Multifunctional SPIO/DOX-loaded wormlike polymer vesicles for cancer therapy and MR imaging. Biomaterials. 2010 Dec;31(34):9065-73. **NO ACCESS**
426. Yata Y, Xue F, Takahara T, Kudo H, Hirano K, Yasumura S, Minemura M, Scanga AE, Sugiyama T. Docetaxel inhibits progression of human hepatoma cell line in vitro and is effective in advanced hepatocellular carcinoma. Hepatol Res. 2010 Apr;40(4):304-10. **NO ACCESS**
427. Yip YL; Section Editor for the IMIA Yearbook Section on Bioinformatics. Closing the Genotype-phenotype Gap. Findings from the Section on Bioinformatics. Yearb Med Inform. 2010:82-5. **NO ACCESS**
428. Yu X, Schneiderhan-Marra N, Joos TO. Protein microarrays for personalized medicine. Clin Chem. 2010 Mar;56(3):376-87.
429. Yurkiewicz S. The prospects for personalized medicine. Hastings Cent Rep. 2010 Sep-Oct;40(5):14-6.
430. Zatloukal K, Hainaut P. Human tissue biobanks as instruments for drug discovery and development: impact on personalized medicine. Biomark Med. 2010 Dec;4(6):895-903. **NO ACCESS**
431. Zaza G, Granata S, Mangino M, Grandaliano G, Schena FP. [From pharmacogenetics to pharmacogenomics: the start of a new era of personalized medicine in nephrology]. G Ital Nefrol. 2010 Jul-Aug;27(4):353-66. Review. Italian.
432. Zhang W, Dolan ME. Impact of the 1000 genomes project on the next wave of pharmacogenomic discovery. Pharmacogenomics. 2010 Feb;11(2):249-56. Review.
433. Zhang Z, Larner SF, Kobeissy F, Hayes RL, Wang KK. Systems biology and theranostic approach to drug discovery and development to treat traumatic brain injury. Methods Mol Biol. 2010;662:317-29. **NO ACCESS**
434. Zhu WB, Long XY, Fan LQ. Male fecundity prognosis and infertility diagnosis in the era of personalised medicine. Asian J Androl. 2010 Jul;12(4):463-7.
435. Ziogas DE, Roukos DH. Limitations of isolated tumor cells in gastric cancer: heterogeneity requests systems biology approaches towards personalized medicine. Ann Surg Oncol. 2010 Jan;17(1):343-4; author reply 345.

**No author stated:**

1. [21st Congress of the Jan Evangelista Purkyne Czech Medical Society in cooperation withe CLS JEP society on the theme of personalized medicine. 8 June 2010]. Cas Lek Cesk. 2010;149(10):459-493. Czech.

**2009**

1. Abman S, Jobe A, Chernick V, Blaisdell C, Castro M, Ramirez MI, Gern JE, Cutting G, Redding G, Hagood JS, Whitsett J, Abman S, Raj JU, Barst R, Kato GJ, Gozal D, Haddad GG, Prabhakar NR, Gauda E, Martinez FD, Tepper R, Wood RE, Accurso F, Teague WG, Venegas J, Cole FS, Wright RJ, Gail D, Hamvas A, Kercsmar C, Kiley J, Weinmann G; NHLBI working group report. Strategic plan for pediatric respiratory diseases research: an NHLBI working group report. Pediatr Pulmonol. 2009 Jan;44(1):2-13.
2. Abrahams E, Silver M. The case for personalized medicine. J Diabetes Sci Technol. 2009 Jul 1;3(4):680-4. Review.
3. Abrahams E. The era of personalized medicine. In the eyes of Edward Abrahams, treatments should be tailored like suits. Interview by Katherine Hobson. US News World Rep. 2009 Aug;146(7):35. **NO ACCESS**
4. Aburatani H. [Genomic technologies in medicine]. Nippon Rinsho. 2009 Jun;67(6):1156-63. Review. Japanese.
5. Ackerman MJ. Personalized medicine. J Med Pract Manage. 2009 Nov-Dec;25(3):194-5. **NO ACCESS**
6. Adams JD, Tom Soh H. Perspectives on utilizing unique features of microfluidics technology for particle and cell sorting. JALA Charlottesv Va. 2009 Dec 1;14(6):331-340.
7. Aggarwal BB, Danda D, Gupta S, Gehlot P. Models for prevention and treatment of cancer: problems vs promises. Biochem Pharmacol. 2009 Nov 1;78(9):1083-94.
8. Agúndez JA, García-Martín E, Martínez C. Genetically based impairment in CYP2C8- and CYP2C9-dependent NSAID metabolism as a risk factor for gastrointestinal bleeding: is a combination of pharmacogenomics and metabolomics required to improve personalized medicine? Expert Opin Drug Metab Toxicol. 2009 Jun;5(6):607-20. Review. **NO ACCESS**
9. Albert M, Peters AH. Genetic and epigenetic control of early mouse development. Curr Opin Genet Dev. 2009 Apr;19(2):113-21.
10. Alemi F, Erdman H, Griva I, Evans CH. Improved Statistical Methods are Needed to Advance Personalized Medicine. Open Transl Med J. 2009 Jan 1;1:16-20.
11. Ambert KH, Cohen AM. A system for classifying disease comorbidity status from medical discharge summaries using automated hotspot and negated concept detection. J Am Med Inform Assoc. 2009 Jul-Aug;16(4):590-5.
12. Andrade LE. Future perspective for diagnosis in autoimmune diseases. An Acad Bras Cienc. 2009 Sep;81(3):367-80.
13. Aoki J, Ikeda K, Iwahashi K. [Serotonin receptor gene polymorphism and analgesic sensitivity]. Masui. 2009 Sep;58(9):1130-5. Review. Japanese.
14. Arain FA, Kuniyoshi FH, Abdalrhim AD, Miller VM. Sex/gender medicine. The biological basis for personalized care in cardiovascular medicine. Circ J. 2009 Oct;73(10):1774-82. Epub 2009 Sep 4. Review.
15. Arnett DK, Claas SA, Lynch AI. Has pharmacogenetics brought us closer to 'personalized medicine' for initial drug treatment of hypertension? Curr Opin Cardiol. 2009 Jul;24(4):333-9. Review.
16. Arnold D, Jones BL. Personalized medicine: a pediatric perspective. Curr Allergy Asthma Rep. 2009 Nov;9(6):426-32. Review.
17. Aspinall M. Personalized medicine and pathology: friend or foe? Arch Pathol Lab Med. 2009 Apr;133(4):527-8.
18. Ateshian GA, Friedman MH. Integrative biomechanics: a paradigm for clinical applications of fundamental mechanics. J Biomech. 2009 Jul 22;42(10):1444-51. Epub 2009 May 12. Review. **NO ACCESS**
19. Bailey RC. Research Spotlight: New multiparameter bioanalytical technologies for applications in personalized medicine, drug discovery and fundamental biology. Bioanalysis. 2009 Sep;1(6):1043-7.
20. Baird PN, Hageman GS, Guymer RH. New era for personalized medicine: the diagnosis and management of age-related macular degeneration. Clin Experiment Ophthalmol. 2009 Nov;37(8):814-21. Review. **NO ACCESS**
21. Banck MS, Grothey A. Biomarkers of Resistance to Epidermal Growth Factor Receptor Monoclonal Antibodies in Patients with Metastatic Colorectal Cancer. Clin Cancer Res. 2009 Dec 15;15(24):7492-7501. **NO ACCESS**
22. Baraldi E, Carraro S, Giordano G, Reniero F, Perilongo G, Zacchello F. Metabolomics: moving towards personalized medicine. Ital J Pediatr. 2009 Oct 23;35(1):30. **NO ACCESS**
23. Baranowska-Kortylewicz J, Henriksen JB. Availability of molecular imaging and "personalized medicine.". J Nucl Med. 2009 Jan;50(1):16N.
24. Barh D, Kumar A, Chatterjee S, Liloglou T. Molecular features, markers, drug targets, and prospective targeted therapeutics in cardiac myxoma. Curr Cancer Drug Targets. 2009 Sep;9(6):705-16. Review. **NO ACCESS**
25. Baudot A, Gómez-López G, Valencia A. Translational disease interpretation with molecular networks. Genome Biol. 2009;10(6):221. Epub 2009 Jun 29. Review.
26. Beganovic S. Clinical significance of the KRAS mutation. Bosn J Basic Med Sci. 2009 Oct;9 Suppl 1:17-20. **NO ACCESS**
27. Bibeau F, Frugier H, Denouel A, Sabourin JC, Boissiere-Michot F. [Technical considerations for KRAS testing in colorectal cancer. The pathologist's point of view]. Bull Cancer. 2009 Dec;96 Suppl:S15-22. French.
28. Bilder RM, Sabb FW, Cannon TD, London ED, Jentsch JD, Parker DS, Poldrack RA, Evans C, Freimer NB. Phenomics: the systematic study of phenotypes on a genome-wide scale. Neuroscience. 2009 Nov 24;164(1):30-42.
29. Boguski MS, Arnaout R, Hill C. Customized care 2020: how medical sequencing and network biology will enable personalized medicine. F1000 Biol Rep. 2009 Sep 28;1:73. doi: 10.3410/B1-73.
30. Borecki IB. Contemporary approaches to gene discovery: progress toward personalized medicine? Circ Cardiovasc Genet. 2009 Feb;2(1):1-2.
31. Borry P. Coming of age of personalized medicine: challenges ahead. Genome Med. 2009 Nov 24;1(11):109.
32. Boucher E, Forner A, Reig M, Bruix J. New drugs for the treatment of hepatocellular carcinoma. Liver Int. 2009 Jan;29 Suppl 1:148-58. Review. **NO ACCESS**
33. Bourre JC, Vuillez JP. [Imaging in oncology and international rules for evaluation: the nuclear medicine]. Bull Cancer. 2009 Nov;96(11):1127-37. Review. French.
34. Braff JP, Chatterjee B, Hochman M, Kennington J, Kolavala C, Layman K, Parver C, Rice CS, Selby MC, Washlick JR, Wolf R. Patient-tailored medicine, part two: personalized medicine and the legal landscape. J Health Life Sci Law. 2009 Jan;2(2):1-3, 5-43. **NO ACCESS**
35. Brammer M. The role of neuroimaging in diagnosis and personalized medicine--current position and likely future directions. Dialogues Clin Neurosci. 2009;11(4):389-96. **NO ACCESS**
36. Brdicka R. [Personalized medicine--genomic phase]. Cas Lek Cesk. 2009;148(10):477-80. Review. Czech.
37. Brewer GJ. Drug development for orphan diseases in the context of personalized medicine. Transl Res. 2009 Dec;154(6):314-22. Epub 2009 Apr 23. Review. **NO ACCESS**
38. Brookes AJ, Chanock SJ, Hudson TJ, Peltonen L, Abecasis G, Kwok PY, Scherer SW. Genomic variation in a global village: report of the 10th annual Human Genome Variation Meeting 2008. Hum Mutat. 2009 Jul;30(7):1134-8.
39. Brown RE. Morphogenomics and morphoproteomics: a role for anatomic pathology in personalized medicine. Arch Pathol Lab Med. 2009 Apr;133(4):568-79.
40. Buetow KH. An infrastructure for interconnecting research institutions. Drug Discov Today. 2009 Jun;14(11-12):605-10. Epub 2009 Apr 1. Review.
41. Burstein HJ. Personalized medicine and breast cancer care. J Natl Compr Canc Netw. 2009 Feb;7(2):109. **NO ACCESS**
42. Califf RM. A virtuous cycle to improve hypertension outcomes at a national level: linking public health and individualized medicine. Hypertension. 2009 Feb;53(2):105-7. doi: 10.1161/HYPERTENSIONAHA.108.121608.
43. Cao Y, Wang N, Ying X, Li A, Wang H, Zhang X, Li W. BioSunMS: a plug-in-based software for the management of patients information and the analysis of peptide profiles from mass spectrometry. BMC Med Inform Decis Mak. 2009 Feb 17;9:13.
44. Carleton B, Poole R, Smith M, Leeder J, Ghannadan R, Ross C, Phillips M, Hayden M. Adverse drug reaction active surveillance: developing a national network in Canada's children's hospitals. Pharmacoepidemiol Drug Saf. 2009 Aug;18(8):713-21. **NO ACCESS**
45. Carlson RJ. The disruptive nature of personalized medicine technologies: implications for the health care system. Public Health Genomics. 2009;12(3):180-4.
46. Castro M, Ramirez MI, Gern JE, Cutting G, Redding G, Hagood JS, Whitsett J, Abman S, Raj JU, Barst R, Kato GJ, Gozal D, Haddad GG, Prabhakar NR, Gauda E, Martinez FD, Tepper R, Wood RE, Accurso F, Teague WG, Venegas J, Cole FS, Wright RJ. Strategic plan for pediatric respiratory diseases research: an NHLBI working group report. Proc Am Thorac Soc. 2009 Jan 15;6(1):1-10.
47. Chackalamannil S, Desai MC. Personalized medicine: a paradigm for a sustainable pharmaceutical industry? Curr Opin Drug Discov Devel. 2009 Jul;12(4):443-5.
48. Chae H, Park SH, Lee SJ, Kim MG, Wedding D, Kwon YK. Psychological profile of sasang typology: a systematic review. Evid Based Complement Alternat Med. 2009 Sep;6 Suppl 1:21-9.
49. Chandrasekharan S, Cook-Deegan R. Gene patents and personalized medicine - what lies ahead? Genome Med. 2009 Sep 28;1(9):92.
50. Chen BJ, Causton HC, Mancenido D, Goddard NL, Perlstein EO, Pe'er D. Harnessing gene expression to identify the genetic basis of drug resistance. Mol Syst Biol. 2009;5:310.
51. Chun S, Fay JC. Identification of deleterious mutations within three human genomes. Genome Res. 2009 Sep;19(9):1553-61.
52. Cipriani B. Immunological markers of cancer vaccine efficacy and their clinical relevance. Biomark Med. 2009 Jun;3(3):253-64. P **NO ACCESS**
53. Claussen CD. [Radiology: on its way to an "individualized" medicine]. Dtsch Med Wochenschr. 2009 May;134(19):975. doi: 10.1055/s-0029-1222552. Epub 2009 Apr 28. German.
54. Collura CA, Johnson JN, Moir C, Ackerman MJ. Left cardiac sympathetic denervation for the treatment of long QT syndrome and catecholaminergic polymorphic ventricular tachycardia using video-assisted thoracic surgery. Heart Rhythm. 2009 Jun;6(6):752-9.
55. Costa IG, Schönhuth A, Hafemeister C, Schliep A. Constrained mixture estimation for analysis and robust classification of clinical time series. Bioinformatics. 2009 Jun 15;25(12):i6-14.
56. Critchley-Thorne RJ, Miller SM, Taylor DL, Lingle WL. Applications of cellular systems biology in breast cancer patient stratification and diagnostics. Comb Chem High Throughput Screen. 2009 Nov;12(9):860-9. Review. **NO ACCESS**
57. Cuijpers P. Prevention: an achievable goal in personalized medicine. Dialogues Clin Neurosci. 2009;11(4):447-54. Review. **NO ACCESS**
58. Davis JC, Furstenthal L, Desai AA, Norris T, Sutaria S, Fleming E, Ma P. The microeconomics of personalized medicine: today's challenge and tomorrow's promise. Nat Rev Drug Discov. 2009 Apr;8(4):279-86. **NO ACCESS**
59. Dawood S, Leyland-Jones B. Pharmacology and pharmacogenetics of chemotherapeutic agents. Cancer Invest. 2009 Jun;27(5):482-8. Review.
60. de Koning P, Keirns J. Clinical pharmacology, biomarkers and personalized medicine: education please. Biomark Med. 2009 Dec;3(6):685-700. **NO ACCESS**
61. De Marco M, Cykert S, Coad N, Doost K, Schaal J, White B, Young D, Isler MR, Corbie-Smith G. Views on personalized medicine: do the attitudes of African American and white prescription drug consumers differ? Public Health Genomics. 2010;13(5):276-83. Epub 2009 Sep 23. Erratum in: Public Health Genomics. 2010;13(6):367. Cykert, S [added]; Coad, N [added]; Doost, K [added]; Schaal, J [added]; White, B [added]; Young, D [added]; Isler, M R [added]; Corbie-Smith, G [added].
62. De Roock W, Biesmans B, De Schutter J, Tejpar S. Clinical biomarkers in oncology: focus on colorectal cancer. Mol Diagn Ther. 2009;13(2):103-14. doi: 10.2165/01250444-200913020-00004. Review. **NO ACCESS**
63. Dean CE. Personalized medicine: boon or budget-buster? Ann Pharmacother. 2009 May;43(5):958-62.
64. Deeken J. The Affymetrix DMET platform and pharmacogenetics in drug development. Curr Opin Mol Ther. 2009 Jun;11(3):260-8. Review.
65. Delbeke D, Schöder H, Martin WH, Wahl RL. Hybrid imaging (SPECT/CT and PET/CT): improving therapeutic decisions. Semin Nucl Med. 2009 Sep;39(5):308-40. Review. **NO ACCESS**
66. Demeure MJ. Personalized medicine: the future is not what it used to be. Surgery. 2009 Dec;146(6):971-8.
67. Donovan MJ, Costa J, Cordon-Cardo C. Systems pathology: a paradigm shift in the practice of diagnostic and predictive pathology. Cancer. 2009 Jul 1;115(13 Suppl):3078-84. Review.
68. Douma K, Prinzen L, Slaaf DW, Reutelingsperger CP, Biessen EA, Hackeng TM, Post MJ, van Zandvoort MA. Nanoparticles for optical molecular imaging of atherosclerosis. Small. 2009 Mar;5(5):544-57. Review. **NO ACCESS**
69. Doweyko AM, Doweyko LM. What is next for small-molecule drug discovery? Future Med Chem. 2009 Sep;1(6):1029-36. **NO ACCESS**
70. Downing GJ, Boyle SN, Brinner KM, Osheroff JA. Information management to enable personalized medicine: stakeholder roles in building clinical decision support. BMC Med Inform Decis Mak. 2009 Oct 8;9:44.
71. Downing GJ. Key aspects of health system change on the path to personalized medicine. Transl Res. 2009 Dec;154(6):272-6.
72. Downing GJ. Policy perspectives on the emerging pathways of personalized medicine. Dialogues Clin Neurosci. 2009;11(4):377-87. Review. **NO ACCESS**
73. Downs BW, Chen AL, Chen TJ, Waite RL, Braverman ER, Kerner M, Braverman D, Rhoades P, Prihoda TJ, Palomo T, Oscar-Berman M, Reinking J, Blum SH, DiNubile NA, Liu HH, Blum K. Nutrigenomic targeting of carbohydrate craving behavior: can we manage obesity and aberrant craving behaviors with neurochemical pathway manipulation by Immunological Compatible Substances (nutrients) using a Genetic Positioning System (GPS) Map? Med Hypotheses. 2009 Sep;73(3):427-34.
74. Drake RE, Cimpean D, Torrey WC. Shared decision making in mental health: prospects for personalized medicine. Dialogues Clin Neurosci. 2009;11(4):455-63. Review. **NO ACCESS**
75. Dressler LG, Terry SF. How will GINA influence participation in pharmacogenomics research and clinical testing? Clin Pharmacol Ther. 2009 Nov;86(5):472-5. **NO ACCESS**
76. Dumontier M, Villanueva-Rosales N. Towards pharmacogenomics knowledge discovery with the semantic web. Brief Bioinform. 2009 Mar;10(2):153-63. Epub 2009 Feb 24.
77. Eddy JA, Geman D, Price ND. Relative expression analysis for identifying perturbed pathways. Conf Proc IEEE Eng Med Biol Soc. 2009;2009:5456-9.
78. Ehdaie B, Smith SC, Theodorescu D. Personalized medicine in advanced urothelial cancer: when to treat, how to treat and who to treat. Can Urol Assoc J. 2009 Dec;3(6 Suppl 4):S232-6. **NO ACCESS**
79. Ely S. Personalized medicine: individualized care of cancer patients. Transl Res. 2009 Dec;154(6):303-8. Epub 2009 Sep 1. Review.
80. Enchin H. Clinician adoption of genetic testing for drug metabolizing enzymes: is patient safety the low-hanging fruit of personalized medicine? AMIA Annu Symp Proc. 2009 Nov 14;2009:168-72.
81. Ennis DP, Pidgeon GP, Millar N, Ravi N, Reynolds JV. Building a bioresource for esophageal research: lessons from the early experience of an academic medical center. Dis Esophagus. 2010 Jan;23(1):1-7.
82. Epstein RJ. Unblocking blockbusters: using boolean text-mining to optimise clinical trial design and timeline for novel anticancer drugs. Cancer Inform. 2009 Aug 17;7:231-8.
83. Evans CP. Identification of molecular targets in urologic oncology. World J Urol. 2009 Feb;27(1):3-8. doi: 10.1007/s00345-008-0339-z. Epub 2008 Nov 12. Review. **NO ACCESS**
84. Evers K. Personalized medicine in psychiatry: ethical challenges and opportunities. Dialogues Clin Neurosci. 2009;11(4):427-34. Review. **NO ACCESS**
85. Fareed KR, Kaye P, Soomro IN, Ilyas M, Martin S, Parsons SL, Madhusudan S. Biomarkers of response to therapy in oesophago-gastric cancer. Gut. 2009 Jan;58(1):127-43. Review.
86. Filaković P, Petek A. Personalized pharmacotherapy in psychiatry. Psychiatr Danub. 2009 Sep;21(3):341-6. Review.
87. Frueh FW. Back to the future: why randomized controlled trials cannot be the answer to pharmacogenomics and personalized medicine. Pharmacogenomics. 2009 Jul;10(7):1077-81. Review.
88. Füessl HS. [When guidelines reach their limits. Wanted: specialist for personalized medicine]. MMW Fortschr Med. 2009 Jun 25;151(26-29):28. German.
89. Furic L, Livingstone M, Dowling RJ, Sonenberg N. Targeting mTOR-dependent tumours with specific inhibitors: a model for personalized medicine based on molecular diagnoses. Curr Oncol. 2009 Jan;16(1):59-61.
90. Furuta T, Kodaira C, Nishino M, Yamade M, Sugimoto M, Ikuma M, Hishida A, Watanabe H, Umemura K. [13C]-pantoprazole breath test to predict CYP2C19 phenotype and efficacy of a proton pump inhibitor, lansoprazole. Aliment Pharmacol Ther. 2009 Aug;30(3):294-300. doi: 10.1111/j.1365-2036.2009.04044.x. **NO ACCESS**
91. Ganser A, Roth G, van Galen JC, Hilderink J, Wammes JJ, Müller I, van Leeuwen FN, Wiesmüller KH, Brock R. Diffusion-driven device for a high-resolution dose-response profiling of combination chemotherapy. Anal Chem. 2009 Jul 1;81(13):5233-40. **NO ACCESS**
92. Gao Y, Holland RD, Yu LR. Quantitative proteomics for drug toxicity. Brief Funct Genomic Proteomic. 2009 Mar;8(2):158-66. Epub 2009 Apr 7. Review.
93. Garber AM, Tunis SR. Does comparative-effectiveness research threaten personalized medicine? N Engl J Med. 2009 May 7;360(19):1925-7.
94. Garber K. Trial offers early test case for personalized medicine. J Natl Cancer Inst. 2009 Feb 4;101(3):136-8.
95. Gazdar AF. Personalized medicine and inhibition of EGFR signaling in lung cancer. N Engl J Med. 2009 Sep 3;361(10):1018-20.
96. Geraci F, Leoncini M, Montangero M, Pellegrini M, Renda ME. K-Boost: a scalable algorithm for high-quality clustering of microarray gene expression data. J Comput Biol. 2009 Jun;16(6):859-73.
97. Germano S, O'Driscoll L. Breast cancer: understanding sensitivity and resistance to chemotherapy and targeted therapies to aid in personalised medicine. Curr Cancer Drug Targets. 2009 May;9(3):398-418. Review. **NO ACCESS**
98. Gerretsen P, Müller DJ, Tiwari A, Mamo D, Pollock BG. The intersection of pharmacology, imaging, and genetics in the development of personalized medicine. Dialogues Clin Neurosci. 2009;11(4):363-76. Review. **NO ACCESS**
99. Ghodke Y, Joshi K, Patwardhan B. Traditional Medicine to Modern Pharmacogenomics: Ayurveda Prakriti Type and CYP2C19 Gene Polymorphism Associated with the Metabolic Variability. Evid Based Complement Alternat Med. 2009 Dec 16.
100. Giampietro PF, McCarty C, Mukesh B, McKiernan F, Wilson D, Shuldiner A, Liu J, LeVasseur J, Ivacic L, Kitchner T, Ghebranious N. The role of cigarette smoking and statins in the development of postmenopausal osteoporosis: a pilot study utilizing the Marshfield Clinic Personalized Medicine Cohort. Osteoporos Int. 2010 Mar;21(3):467-77. Epub 2009 Jun 9. Review.
101. Ginsburg GS, Willard HF. Genomic and personalized medicine: foundations and applications. Transl Res. 2009 Dec;154(6):277-87. Epub 2009 Oct 1. Review.
102. Glatt SJ, Chandler SD, Bousman CA, Chana G, Lucero GR, Tatro E, May T, Lohr JB, Kremen WS, Everall IP, Tsuang MT. Alternatively Spliced Genes as Biomarkers for Schizophrenia, Bipolar Disorder and Psychosis: A Blood-Based Spliceome-Profiling Exploratory Study. Curr Pharmacogenomics Person Med. 2009 Sep;7(3):164-188.
103. Gómez de la Concha E. [Complex diseases genetics]. An R Acad Nac Med (Madr). 2009;126(2):229-38; discussion 238-41. Spanish.
104. Gondo Y, Fukumura R, Murata T, Makino S. Next-generation gene targeting in the mouse for functional genomics. BMB Rep. 2009 Jun 30;42(6):315-23. Review.
105. Greek J, Greek R. Thoughts on the benefits of personalized medicine. J Am Vet Med Assoc. 2009 Jan 1;234(1):40.
106. Griggs JJ. Personalized medicine: a perk of privilege? Clin Pharmacol Ther. 2009 Jul;86(1):21-3.
107. Gundlapalli AV, Delgado JC, Jackson BR, Tricot GJ, Hill HR. Composite patient reports: a laboratory informatics perspective and pilot project for personalized medicine and translational research. Summit on Translat Bioinforma. 2009 Mar 1;2009:39-43.
108. Harper CC. Personalized medicine in diabetes: regulatory considerations. J Diabetes Sci Technol. 2009 Jul 1;3(4):739-42.
109. Hata A, Onouchi Y. Susceptibility genes for Kawasaki disease: toward implementation of personalized medicine. J Hum Genet. 2009 Feb;54(2):67-73. Epub 2009 Jan 16. Review.
110. Hatzimouratidis K, Hatzichristou DG. Phosphodiesterase type 5 inhibitors: unmet needs. Curr Pharm Des. 2009;15(30):3476-85. Review. **NO ACCESS**
111. Hein DW. N-acetyltransferase SNPs: emerging concepts serve as a paradigm for understanding complexities of personalized medicine. Expert Opin Drug Metab Toxicol. 2009 Apr;5(4):353-66. Review.
112. Heinrich AC, Patel SA, Reddy BY, Milton R, Rameshwar P. Multi- and inter-disciplinary science in personalized delivery of stem cells for tissue repair. Curr Stem Cell Res Ther. 2009 Jan;4(1):16-22. Review.
113. Hernandez JP, Mota LC, Baldwin WS. Activation of CAR and PXR by Dietary, Environmental and Occupational Chemicals Alters Drug Metabolism, Intermediary Metabolism, and Cell Proliferation. Curr Pharmacogenomics Person Med. 2009 Jun 1;7(2):81-105.
114. Herrmann J, Mannheim D, Wohlert C, Versari D, Meyer FB, McConnell JP, Gössl M, Lerman LO, Lerman A. Expression of lipoprotein-associated phospholipase A(2) in carotid artery plaques predicts long-term cardiac outcome. Eur Heart J. 2009 Dec;30(23):2930-8.
115. Hess JL. What hematopathology tells us about the future of pathology informatics. Arch Pathol Lab Med. 2009 Jun;133(6):908-11. Review.
116. Hidalgo-Miranda A, Jiménez-Sánchez G. [Genomic basis for breast cancer: advances in personalized medicine]. Salud Publica Mex. 2009;51 Suppl 2:s197-207. Review. Spanish.
117. Hirsch FR. The role of genetic testing in the prediction of response to EGFR inhibitors in NSCLC. Oncogene. 2009 Aug;28 Suppl 1:S1-3.
118. Ho L, Fivecoat H, Wang J, Pasinetti GM. Alzheimer's disease biomarker discovery in symptomatic and asymptomatic patients: experimental approaches and future clinical applications. Exp Gerontol. 2010 Jan;45(1):15-22. Epub 2009 Sep 29. Review.
119. Holford ME, Rajeevan H, Zhao H, Kidd KK, Cheung KH. Semantic Web-based integration of cancer pathways and allele frequency data. Cancer Inform. 2009 Jan 15;8:19-30.
120. Holmes MV, Shah T, Vickery C, Smeeth L, Hingorani AD, Casas JP. Fulfilling the promise of personalized medicine? Systematic review and field synopsis of pharmacogenetic studies. PLoS One. 2009 Dec 2;4(12):e7960. Review.
121. Hudson TJ. Personalized medicine: a transformative approach is needed. CMAJ. 2009 Apr 28;180(9):911-3.
122. Hudson TJ. Personalized medicine: A transformative approach is needed. CMAJ. 2009 Feb 18.
123. Huh YS, Lowe AJ, Strickland AD, Batt CA, Erickson D. Surface-enhanced Raman scattering based ligase detection reaction. J Am Chem Soc. 2009 Feb 18;131(6):2208-13.
124. Hulot JS, Fuster V. Antiplatelet therapy: Personalized medicine for clopidogrel resistance? Nat Rev Cardiol. 2009 May;6(5):334-6. **NO ACCESS**
125. Hunt CA, Ropella GE, Lam TN, Tang J, Kim SH, Engelberg JA, Sheikh-Bahaei S. At the biological modeling and simulation frontier. Pharm Res. 2009 Nov;26(11):2369-400. Epub 2009 Sep 9. Review.
126. Hunter P. Reading the metabolic fine print. The application of metabolomics to diagnostics, drug research and nutrition might be integral to improved health and personalized medicine. EMBO Rep. 2009 Jan;10(1):20-3.
127. Iseki M, Sato-Takeda M. [Implication of genetic polymorphism on neuropathic pain]. Masui. 2009 Sep;58(9):1112-21. Review. Japanese.
128. Issa AM, Tufail W, Hutchinson J, Tenorio J, Baliga MP. Assessing patient readiness for the clinical adoption of personalized medicine. Public Health Genomics. 2009;12(3):163-9.
129. Jain KK. Personalized clinical laboratory diagnostics. Adv Clin Chem. 2009;47:95-119. Review. **NO ACCESS**
130. Jain KK. Role of nanobiotechnology in the development of personalized medicine. Nanomedicine (Lond). 2009 Apr;4(3):249-52.
131. Jain KK. The role of nanobiotechnology in drug discovery. Adv Exp Med Biol. 2009;655:37-43. Review.
132. Jakobsdottir J, Gorin MB, Conley YP, Ferrell RE, Weeks DE. Interpretation of genetic association studies: markers with replicated highly significant odds ratios may be poor classifiers. PLoS Genet. 2009 Feb;5(2):e1000337.
133. Jakovljević M. New generation vs. first generation antipsychotics debate: pragmatic clinical trials and practice-based evidence. Psychiatr Danub. 2009 Dec;21(4):446-52. **NO ACCESS**
134. Jennings LE, Long NJ. 'Two is better than one'--probes for dual-modality molecular imaging. Chem Commun (Camb). 2009 Jun 28;(24):3511-24. Epub 2009 May 5. Review.
135. Jiang Y, Kimchi ET, Staveley-O'Carroll KF, Cheng H, Ajani JA. Assessment of K-ras mutation: a step toward personalized medicine for patients with colorectal cancer. Cancer. 2009 Aug 15;115(16):3609-17. Review.
136. John T, Liu G, Tsao MS. Overview of molecular testing in non-small-cell lung cancer: mutational analysis, gene copy number, protein expression and other biomarkers of EGFR for the prediction of response to tyrosine kinase inhibitors. Oncogene. 2009 Aug;28 Suppl 1:S14-23. Review. **NO ACCESS**
137. Jolly Graham A, Potti A. Translating genomics into clinical practice: applications in lung cancer. Curr Oncol Rep. 2009 Jul;11(4):263-8. Review. **NO ACCESS**
138. Jordan VC. A century of deciphering the control mechanisms of sex steroid action in breast and prostate cancer: the origins of targeted therapy and chemoprevention. Cancer Res. 2009 Feb 15;69(4):1243-54. doi: 10.1158/0008-5472.CAN-09-0029. Epub 2009 Feb 10.
139. Jørgensen JT. New era of personalized medicine: a 10-year anniversary. Oncologist. 2009 May;14(5):557-8.
140. Kanashiro-Takeuchi RM, Heidecker B, Lamirault G, Dharamsi JW, Hare JM. Sex-specific impact of aldosterone receptor antagonism on ventricular remodeling and gene expression after myocardial infarction. Clin Transl Sci. 2009 Apr;2(2):134-42.
141. Kapse N, Goh V. Functional imaging of colorectal cancer: positron emission tomography, magnetic resonance imaging, and computed tomography. Clin Colorectal Cancer. 2009 Mar;8(2):77-87.
142. Karger BL, Guttman A. DNA sequencing by CE. Electrophoresis. 2009 Jun;30 Suppl 1:S196-202. Review.
143. Katoh Y, Katoh M. FGFR2-related pathogenesis and FGFR2-targeted therapeutics (Review). Int J Mol Med. 2009 Mar;23(3):307-11. Review.
144. Kawamoto K, Lobach DF, Willard HF, Ginsburg GS. A national clinical decision support infrastructure to enable the widespread and consistent practice of genomic and personalized medicine. BMC Med Inform Decis Mak. 2009 Mar 23;9:17.
145. Kedmi R, Peer D. RNAi nanoparticles in the service of personalized medicine. Nanomedicine (Lond). 2009 Dec;4(8):853-5.
146. Kelleher KJ, Stevens J. Evolution of child mental health services in primary care. Acad Pediatr. 2009 Jan-Feb;9(1):7-14.
147. Kim H, Dionne RA. Individualized pain medicine. Drug Discov Today Ther Strateg. 2009;6(3):83-87.
148. Kim PG, Daley GQ. Application of induced pluripotent stem cells to hematologic disease. Cytotherapy. 2009;11(8):980-9. Review. **NO ACCESS**
149. Kirmani S, Babovic-Vuksanovic D. Towards individualized medicine: insights gained from genomic studies. Bosn J Basic Med Sci. 2009 Oct;9 Suppl 1:11-6. **NO ACCESS**
150. Klonoff DC. The personalized medicine for diabetes meeting summary report. J Diabetes Sci Technol. 2009 Jul 1;3(4):677-9.
151. Knight JC. Genetics and the general physician: insights, applications and future challenges. QJM. 2009 Nov;102(11):757-72. Epub 2009 Sep 7. Review.
152. Ko AH, Tempero MA. Personalized medicine for pancreatic cancer: a step in the right direction. Gastroenterology. 2009 Jan;136(1):43-5.
153. Kohane IS. The twin questions of personalized medicine: who are you and whom do you most resemble? Genome Med. 2009 Jan 20;1(1):4.
154. Kojima C, Hirano Y, Kono K. Chapter 7 - Preparation of complexes of liposomes with gold nanoparticles. Methods Enzymol. 2009;464:131-45. **NO ACCESS**
155. Kojima Y, Sasaki S, Hayashi Y, Tsujimoto G, Kohri K. Subtypes of alpha1-adrenoceptors in BPH: future prospects for personalized medicine. Nat Clin Pract Urol. 2009 Jan;6(1):44-53. Review. **NO ACCESS**
156. Kolligs FT. Conference scene: DGVS spring conference 2009. Biomark Med. 2009 Oct;3(5):573-5.
157. Kondo T, Hirohashi S. Application of 2D-DIGE in cancer proteomics toward personalized medicine. Methods Mol Biol. 2009;577:135-54.
158. Konoshita T, Kato N, Fuchs S, Mizuno S, Aoyama C, Motomura M, Makino Y, Wakahara S, Inoki I, Miyamori I, Pinet F; Genomic Disease Outcome Consortium (G-DOC) Study Investigators. Genetic variant of the Renin-Angiotensin system and diabetes influences blood pressure response to Angiotensin receptor blockers. Diabetes Care. 2009 Aug;32(8):1485-90.
159. Koo I, Kim JY, Kim MG, Kim KH. Feature selection from a facial image for distinction of sasang constitution. Evid Based Complement Alternat Med. 2009 Sep;6 Suppl 1:65-71.
160. Kooperberg C, Leblanc M, Dai JY, Rajapakse I. Structures and Assumptions: Strategies to Harness Gene × Gene and Gene × Environment Interactions in GWAS. Stat Sci. 2009;24(4):472-488.
161. Kurova VS, Kurochkin IN, Kalamkarov GR, Bugrova AE, Fedortchenko KY, Varfolomeev SD. Structural and catalytic polymorphism of human enzymes: Novel potential platforms for biomedical diagnostics. Biotechnol Adv. 2009 Nov-Dec;27(6):945-59. Epub 2009 May 21. Review. **NO ACCESS**
162. Landais P, Méresse V, Ghislain JC. Evaluation and validation of diagnostic tests for guiding therapeutic decisions. Therapie. 2009 May-Jun;64(3):187-201. Epub 2009 Aug 13. Review. English, French.
163. Lau KC, Mak CM, Leung KY, Tsoi TH, Tang HY, Lee P, Lam CW. A fast modified protocol for random-access ultra-high density whole-genome scan: a tool for personalized genomic medicine, positional mapping, and cytogenetic analysis. Clin Chim Acta. 2009 Aug;406(1-2):31-5. **NO ACCESS**
164. Laurence J. Getting personal: the promises and pitfalls of personalized medicine. Transl Res. 2009 Dec;154(6):269-71.
165. Le-Niculescu H, Patel SD, Bhat M, Kuczenski R, Faraone SV, Tsuang MT, McMahon FJ, Schork NJ, Nurnberger JI Jr, Niculescu AB 3rd. Convergent functional genomics of genome-wide association data for bipolar disorder: comprehensive identification of candidate genes, pathways and mechanisms. Am J Med Genet B Neuropsychiatr Genet. 2009 Mar 5;150B(2):155-81.
166. Lee LP. Satellite nanoscope and cellular BioASICs for quantitative biomedicine. Conf Proc IEEE Eng Med Biol Soc. 2009;2009:4582-5. **NO ACCESS**
167. Lee SH, Shin DJ, Jang Y. Personalized medicine in coronary artery disease: insights from genomic research. Korean Circ J. 2009 Apr;39(4):129-37.
168. Lee SS, Mudaliar A. Medicine. Racing forward: the Genomics and Personalized Medicine Act. Science. 2009 Jan 16;323(5912):342.
169. Lee SS. Pharmacogenomics and the challenge of health disparities. Public Health Genomics. 2009;12(3):170-9.
170. Leeder JS, Spielberg SP. Personalized medicine: reality and reality checks. Ann Pharmacother. 2009 May;43(5):963-6.
171. Li Y, Jing Y, Yao X, Srinivasan B, Xu Y, Xing C, Wang JP. Biomarkers identification and detection based on GMR sensor and sub 13 nm magnetic nanoparticles. Conf Proc IEEE Eng Med Biol Soc. 2009;2009:5432-5. **NO ACCESS**
172. Li-Wan-Po A, Farndon P, Cooley C, Lithgow J. When Is a Genetic Test Suitable for Prime Time? Predicting the Risk of Prostate Cancer as a Case-Example. Public Health Genomics. 2009 May 13.
173. Lieberman R. Personalized medicine enters the US marketplace: KRAS, anti-EGFR monoclonal antibodies, and colon cancer. Am J Ther. 2009 Nov-Dec;16(6):477-9. **NO ACCESS**
174. Lima JJ, Blake KV, Tantisira KG, Weiss ST. Pharmacogenetics of asthma. Curr Opin Pulm Med. 2009 Jan;15(1):57-62. Review.
175. Lin B, Strickland SJ, Wang J, Qi L, Kolker E, Nam HG, Li L, Gu J. Meeting report: the 2009 Westlake International Conference on Personalized Medicine. OMICS. 2009 Aug;13(4):285-9.
176. Liou G, El-Remessy A, Ibrahim A, Caldwell R, Khalifa Y, Gunes A, Nussbaum J. Cannabidiol As a Putative Novel Therapy for Diabetic Retinopathy: A Postulated Mechanism of Action as an Entry Point for Biomarker-Guided Clinical Development. Curr Pharmacogenomics Person Med. 2009 Sep;7(3):215-222.
177. Littman BH. Tocilizumab and missed personalized medicine opportunities for patients with rheumatoid arthritis? Arthritis Rheum. 2009 May;60(5):1565-6.
178. Liu T, Peng H, Zhou X. Imaging informatics for personalised medicine: applications and challenges. Int J Funct Inform Personal Med. 2009;2(2):125-135.
179. Lostia AM, Lionetto L, Ialongo C, Gentile G, Viterbo A, Malaguti P, Paris I, Marchetti L, Marchetti P, De Blasi A, Simmaco M. A liquid chromatography-tandem mass spectrometry method for the determination of 5-Fluorouracil degradation rate by intact peripheral blood mononuclear cells. Ther Drug Monit. 2009 Aug;31(4):482-8. **NO ACCESS**
180. Luttropp K, Lindholm B, Carrero JJ, Glorieux G, Schepers E, Vanholder R, Schalling M, Stenvinkel P, Nordfors L. Genetics/Genomics in chronic kidney disease--towards personalized medicine? Semin Dial. 2009 Jul-Aug;22(4):417-22. Review.
181. Macdonald IM. Pharmacogenetics - getting closer. Open Ophthalmol J. 2009 Sep 17;3:46-9.
182. Mackay HJ, Oza AM. Other new targets. Int J Gynecol Cancer. 2009 Dec;19 Suppl 2:S49-54. Review. **NO ACCESS**
183. Madhavan S, Zenklusen JC, Kotliarov Y, Sahni H, Fine HA, Buetow K. Rembrandt: helping personalized medicine become a reality through integrative translational research. Mol Cancer Res. 2009 Feb;7(2):157-67.
184. Manace LC, Godiwala TN, Babyatsky MW. Genomics of cardiovascular disease. Mt Sinai J Med. 2009 Dec;76(6):613-23. Review.
185. Mandrekar SJ, Sargent DJ. Clinical trial designs for predictive biomarker validation: one size does not fit all. J Biopharm Stat. 2009;19(3):530-42.
186. Matsushima Y. The new disease models from genetic polymorphisms of Japanese wild mice. Curr Pharm Biotechnol. 2009 Feb;10(2):230-5.
187. McNearney TA, Hunnicutt SE, Fischbach M, Friedman AW, Aguilar M, Ahn CW, Reveille JD, Lisse JR, Baethge BA, Goel N, Mayes MD; GENISOS Study Group. Perceived functioning has ethnic-specific associations in systemic sclerosis: another dimension of personalized medicine. J Rheumatol. 2009 Dec;36(12):2724-32.
188. McShane LM, Hunsberger S, Adjei AA. Effective incorporation of biomarkers into phase II trials. Clin Cancer Res. 2009 Mar 15;15(6):1898-905. Epub 2009 Mar 10. Review.
189. Meckley LM, Neumann PJ. Personalized medicine: factors influencing reimbursement. Health Policy. 2010 Feb;94(2):91-100. **NO ACCESS**
190. Meléndez-Zajgla J. [Personalized medicine. So close and so far away]. Rev Invest Clin. 2009 Nov-Dec;61(6):454-5. Spanish.
191. Mendrick DL, Schnackenberg L. Genomic and metabolomic advances in the identification of disease and adverse event biomarkers. Biomark Med. 2009 Oct;3(5):605-15.
192. Menter DG, Schilsky RL, DuBois RN. Cyclooxygenase-2 and cancer treatment: understanding the risk should be worth the reward. Clin Cancer Res. 2010 Mar 1;16(5):1384-90. doi: 10.1158/1078-0432.CCR-09-0788. Epub 2010 Feb 23. Review.
193. Meschia JF. Pharmacogenetics and stroke. Stroke. 2009 Nov;40(11):3641-5. doi: 10.1161/STROKEAHA.109.562231.
194. Michiue H, Eguchi A, Scadeng M, Dowdy SF. Induction of in vivo synthetic lethal RNAi responses to treat glioblastoma. Cancer Biol Ther. 2009 Dec;8(23):2306-13.
195. Minami Y, Kasukawa T, Kakazu Y, Iigo M, Sugimoto M, Ikeda S, Yasui A, van der Horst GT, Soga T, Ueda HR. Measurement of internal body time by blood metabolomics. Proc Natl Acad Sci U S A. 2009 Jun 16;106(24):9890-5.
196. Mínguez B, Tovar V, Chiang D, Villanueva A, Llovet JM. Pathogenesis of hepatocellular carcinoma and molecular therapies. Curr Opin Gastroenterol. 2009 May;25(3):186-94. Review.
197. Mishra PJ, Bertino JR. MicroRNA polymorphisms: the future of pharmacogenomics, molecular epidemiology and individualized medicine. Pharmacogenomics. 2009 Mar;10(3):399-416. doi: 10.2217/14622416.10.3.399. Review.
198. Mishra PJ. MicroRNA polymorphisms: a giant leap towards personalized medicine. Per Med. 2009 Mar 1;6(2):119-125.
199. Mitani Y, Lezhava A, Sakurai A, Horikawa A, Nagakura M, Hayashizaki Y, Ishikawa T. Rapid and cost-effective SNP detection method: application of SmartAmp2 to pharmacogenomics research. Pharmacogenomics. 2009 Jul;10(7):1187-97. doi: 10.2217/pgs.09.39. **NO ACCESS**
200. Mitchell LR, Albert JM, Lu B. Chemoradiotherapy in locally advanced, unresectable non-small cell lung cancer. Rev Recent Clin Trials. 2009 May;4(2):110-21. Review. **NO ACCESS**
201. Modak AS. (13)C breath tests in personalized medicine: fiction or reality? Expert Rev Mol Diagn. 2009 Nov;9(8):805-15. Review. **NO ACCESS**
202. Mok T, Wu YL, Zhang L. A small step towards personalized medicine for non-small cell lung cancer. Discov Med. 2009 Dec;8(43):227-31. Review.
203. Moridani M, Maitland-van der Zee AH, Sasaki H, McKinnon R, Fleckenstein L, Shah VP. AAPS-FIP summary workshop report: Pharmacogenetics in individualized medicine: methods, regulatory, and clinical applications. AAPS J. 2009 Jun;11(2):214-6. doi: 10.1208/s12248-009-9097-0.
204. Moyer BR, Barrett JA. Biomarkers and imaging: physics and chemistry for noninvasive analyses. Bioanalysis. 2009 May;1(2):321-56. Review.
205. Murakami Y. [Advances in genome analysis of solid tumors]. Nippon Rinsho. 2009 Jun;67(6):1115-9. Review. Japanese.
206. Murphy P, Kean MA. IT leaders: prepare for personalized medicine. PM is a disruptive force in medical practice that has been evolving rapidly over the last few years. Health Manag Technol. 2009 Dec;30(12):10-1.
207. Nakamura N, Ito K, Takahashi M, Hongo S, Hashimoto K, Kawamoto M, Taniguchi A, Kamatani N, Gemma N. Clinical verification of a combination technology of a loop-mediated isothermal amplification method and an electrochemical DNA chip for personalized medicine. Clin Biochem. 2009 Jul;42(10-11):1158-61. **NO ACCESS**
208. Nelson SF, Crosbie RH, Miceli MC, Spencer MJ. Emerging genetic therapies to treat Duchenne muscular dystrophy. Curr Opin Neurol. 2009 Oct;22(5):532-8. Review.
209. Ng PC, Murray SS, Levy S, Venter JC. An agenda for personalized medicine. Nature. 2009 Oct 8;461(7265):724-6. **NO ACCESS**
210. Nikolsky Y, Kirillov E, Zuev R, Rakhmatulin E, Nikolskaya T. Functional analysis of OMICs data and small molecule compounds in an integrated "knowledge-based" platform. Methods Mol Biol. 2009;563:177-96. **NO ACCESS**
211. Niu G, Chen X. From protein-protein interaction to therapy response: molecular imaging of heat shock proteins. Eur J Radiol. 2009 May;70(2):294-304. doi: 10.1016/j.ejrad.2009.01.052. Epub 2009 Mar 20. Review. **NO ACCESS**
212. Norris PR, Canter JA, Jenkins JM, Moore JH, Williams AE, Morris JA Jr. Personalized medicine: genetic variation and loss of physiologic complexity are associated with mortality in 644 trauma patients. Ann Surg. 2009 Oct;250(4):524-30.
213. Ochs MF, Rink L, Tarn C, Mburu S, Taguchi T, Eisenberg B, Godwin AK. Detection of treatment-induced changes in signaling pathways in gastrointestinal stromal tumors using transcriptomic data. Cancer Res. 2009 Dec 1;69(23):9125-32.
214. Oguamanam C. Personalized medicine and complementary and alternative medicine: in search of common grounds. J Altern Complement Med. 2009 Aug;15(8):943-9. **NO ACCESS**
215. Ohmann C, Kuchinke W. Future developments of medical informatics from the viewpoint of networked clinical research. Interoperability and integration. Methods Inf Med. 2009;48(1):45-54.
216. Oliveira E, Pereira R, Amorim A, McLeod H, Prata MJ. Patterns of pharmacogenetic diversity in African populations: role of ancient and recent history. Pharmacogenomics. 2009 Sep;10(9):1413-22. **NO ACCESS**
217. Onitilo AA, McCarty CA, Wilke RA, Glurich I, Engel JM, Flockhart DA, Nguyen A, Li L, Mi D, Skaar TC, Jin Y. Estrogen receptor genotype is associated with risk of venous thromboembolism during tamoxifen therapy. Breast Cancer Res Treat. 2009 Jun;115(3):643-50. **NO ACCESS**
218. Ortiz MF, Rodríguez-García MI, Hermida-Prieto M, Fernández X, Veira E, Barriales-Villa R, Castro-Beiras A, Monserrat L. A homozygous MYBPC3 gene mutation associated with a severe phenotype and a high risk of sudden death in a family with hypertrophic cardiomyopathy. Rev Esp Cardiol. 2009 May;62(5):572-5. English, Spanish.
219. Osamura RY, Gown AM. Personalized medicine-molecular targeted cancer therapy. J Cell Mol Med. 2009 Nov-Dec;13(11-12):4285. **NO ACCESS**
220. Overdevest JB, Theodorescu D, Lee JK. Utilizing the molecular gateway: the path to personalized cancer management. Clin Chem. 2009 Apr;55(4):684-97.
221. Ozdemir V, Husereau D, Hyland S, Samper S, Salleh MZ. Personalized Medicine Beyond Genomics: New Technologies, Global Health Diplomacy and Anticipatory Governance. Curr Pharmacogenomics Person Med. 2009 Dec;7(4):225-230.
222. Ozdemir V, Suarez-Kurtz G, Stenne R, Somogyi AA, Someya T, Kayaalp SO, Kolker E. Risk assessment and communication tools for genotype associations with multifactorial phenotypes: the concept of "edge effect" and cultivating an ethical bridge between omics innovations and society. OMICS. 2009 Feb;13(1):43-61.
223. Pan D, Lanza GM, Wickline SA, Caruthers SD. Nanomedicine: perspective and promises with ligand-directed molecular imaging. Eur J Radiol. 2009 May;70(2):274-85. **NO ACCESS**
224. Panagiotou G, Nielsen J. Nutritional systems biology: definitions and approaches. Annu Rev Nutr. 2009;29:329-39. Review. **NO ACCESS**
225. Park SH, Kim MG, Lee SJ, Kim JY, Chae H. Temperament and Character Profiles of Sasang Typology in an Adult Clinical Sample. Evid Based Complement Alternat Med. 2009 Nov 17.
226. Park SK, Choi JY. Risk assessment and pharmacogenetics in molecular and genomic epidemiology. J Prev Med Public Health. 2009 Nov;42(6):371-6. Review. Erratum in: J Prev Med Public Health. 2010 Jan;43(1):96-7. **NO ACCESS**
227. Parkinson DR, Ziegler J. Educating for personalized medicine: a perspective from oncology. Clin Pharmacol Ther. 2009 Jul;86(1):23-5. Review.
228. Parsons BL, Meng F. K-RAS mutation in the screening, prognosis and treatment of cancer. Biomark Med. 2009 Dec;3(6):757-69.
229. Pauly MV. Is it time to reexamine the patent system's role in spending growth? Health Aff (Millwood). 2009 Sep-Oct;28(5):1466-74. **NO ACCESS**
230. Pavlovic S. TPMT gene polymorphisms: on the doorstep of personalized medicine. Indian J Med Res. 2009 May;129(5):478-80.
231. Pene F, Courtine E, Cariou A, Mira JP. Toward theragnostics. Crit Care Med. 2009 Jan;37(1 Suppl):S50-8. Review. **NO ACCESS**
232. Perlis RH, Patrick A, Smoller JW, Wang PS. When is pharmacogenetic testing for antidepressant response ready for the clinic? A cost-effectiveness analysis based on data from the STAR*D study. Neuropsychopharmacology. 2009 Sep;34(10):2227-36.
233. Petersen A. The ethics of expectations: biobanks and the promise of personalised medicine. Monash Bioeth Rev. 2009 Mar;28(1):5.1-12. Review. **NO ACCESS**
234. Peterson QP, Hsu DC, Goode DR, Novotny CJ, Totten RK, Hergenrother PJ. Procaspase-3 activation as an anti-cancer strategy: structure-activity relationship of procaspase-activating compound 1 (PAC-1) and its cellular co-localization with caspase-3. J Med Chem. 2009 Sep 24;52(18):5721-31.
235. Phan JH, Moffitt RA, Stokes TH, Liu J, Young AN, Nie S, Wang MD. Convergence of biomarkers, bioinformatics and nanotechnology for individualized cancer treatment. Trends Biotechnol. 2009 Jun;27(6):350-8. Epub 2009 May 4. Review.
236. Piatkov I, Jones T, Rochester C. Cytochrome P450 loss-of-function polymorphism genotyping on the Agilent Bioanalyzer and clinical application. Pharmacogenomics. 2009 Dec;10(12):1987-94. **NO ACCESS**
237. Poland GA, Jacobson RM, Ovsyannikova IG. Trends affecting the future of vaccine development and delivery: the role of demographics, regulatory science, the anti-vaccine movement, and vaccinomics. Vaccine. 2009 May 26;27(25-26):3240-4. doi: 10.1016/j.vaccine.2009.01.069. Epub 2009 Feb 5. Review.
238. Primorac D. Human Genome Project-based applications in forensic science, anthropology, and individualized medicine. Croat Med J. 2009 Jun;50(3):205-6.
239. Putre L. Personalized medicine. Getting genomic data into EMRs proves challenging. Hosp Health Netw. 2009 Jul;83(7):20, 22. **NO ACCESS**
240. Qin WJ, Yung LY. Nanoparticle carrying a single probe for target DNA detection and single nucleotide discrimination. Biosens Bioelectron. 2009 Oct 15;25(2):313-9.
241. Quintana LF, Bañon-Maneus E, Solé-Gonzalez A, Campistol JM. Urine proteomics biomarkers in renal transplantation: an overview. Transplantation. 2009 Aug15;88(3 Suppl):S45-9. Review. **NO ACCESS**
242. Ratner M. ARRA boosts cancer programs but impact on personalized medicine unclear. Nat Biotechnol. 2009 Dec;27(12):1062.
243. Rix U, Superti-Furga G. Target profiling of small molecules by chemical proteomics. Nat Chem Biol. 2009 Sep;5(9):616-24. Review.
244. Robertson GR, Grant DM, Piquette-Miller M. Pharmacogenetics of pharmacoecology: which route to personalized medicine? Clin Pharmacol Ther. 2009 Apr;85(4):343-8. **NO ACCESS**
245. Rockhill C, Althoff RR. Personalized medicine and pharmacogenomics in child psychiatry. J Am Acad Child Adolesc Psychiatry. 2009 Dec;48(12):1136.
246. Roederer MW. Cytochrome P450 enzymes and genotype-guided drug therapy. Curr Opin Mol Ther. 2009 Dec;11(6):632-40. Review. **NO ACCESS**
247. Ross JS, Slodkowska EA, Symmans WF, Pusztai L, Ravdin PM, Hortobagyi GN. The HER-2 receptor and breast cancer: ten years of targeted anti-HER-2 therapy and personalized medicine. Oncologist. 2009 Apr;14(4):320-68. Epub 2009 Apr 3. Review.
248. Rougier P, Mitry E. [Targeted biotherapy: a revolution in the management of patients with colorectal cancer?]. Gastroenterol Clin Biol. 2009 Aug-Sep;33(8-9):672-80. Epub 2009 Aug 29. French.
249. Rovin BH, McKinley AM, Birmingham DJ. Can we personalize treatment for kidney diseases? Clin J Am Soc Nephrol. 2009 Oct;4(10):1670-6.
250. Ruaño G, Duconge J, Windemuth A, Cadilla CL, Kocherla M, Villagra D, Renta J, Holford T, Santiago-Borrero PJ. Physiogenomic analysis of the Puerto Rican population. Pharmacogenomics. 2009 Apr;10(4):565-77.
251. Ruderfer DM, Roberts DC, Schreiber SL, Perlstein EO, Kruglyak L. Using expression and genotype to predict drug response in yeast. PLoS One. 2009 Sep 4;4(9):e6907.
252. Rumilla K, Chen D, Baudhuin LM. Pharmacogenetics in hemostasis: friend or foe? Semin Thromb Hemost. 2009 Feb;35(1):42-9. doi: 10.1055/s-0029-1214147. Epub 2009 Mar 23. Review. **NO ACCESS**
253. Sajja HK, East MP, Mao H, Wang YA, Nie S, Yang L. Development of multifunctional nanoparticles for targeted drug delivery and noninvasive imaging of therapeutic effect. Curr Drug Discov Technol. 2009 Mar;6(1):43-51. Review.
254. Salari K. The dawning era of personalized medicine exposes a gap in medical education. PLoS Med. 2009 Aug;6(8):e1000138.
255. Sanders D, Kmiecik T. Personalized medicine gets practical. Healthc Inform. 2009 May;26(5):50-1. **NO ACCESS**
256. Santos ES, Blaya M, Raez LE. Gene expression profiling and non-small-cell lung cancer: where are we now? Clin Lung Cancer. 2009 May;10(3):168-73. Review.
257. Scattereggia J. Financial Times Global Pharmaceutical & Biotechnology Conference 2009. IDrugs. 2010 Jan;13(1):26-9. **NO ACCESS**
258. Schiffer E. The 2nd annual oncology biomarkers conference. Biomark Med. 2009 Apr;3(2):203-9. **NO ACCESS**
259. Schottelius M, Laufer B, Kessler H, Wester HJ. Ligands for mapping alphavbeta3-integrin expression in vivo. Acc Chem Res. 2009 Jul 21;42(7):969-80. doi: 10.1021/ar800243b. **NO ACCESS**
260. Schulman KA, Vidal AV, Ackerly DC. Personalized medicine and disruptive innovation: implications for technology assessment. Genet Med. 2009 Aug;11(8):577-81.
261. Schwarz E, Leweke FM, Bahn S, Liò P. Clinical bioinformatics for complex disorders: a schizophrenia case study. BMC Bioinformatics. 2009 Oct 15;10 Suppl 12:S6.
262. Sethi P, Theodos K. Translational bioinformatics and healthcare informatics: computational and ethical challenges. Perspect Health Inf Manag. 2009 Sep 16;6:1h.
263. Shevde LA, Riker AI. Current concepts in biobanking: development and implementation of a tissue repository. Front Biosci (Schol Ed). 2009 Jun 1;1:188-93. Review. **NO ACCESS**
264. Simpson KL, Whetton AD, Dive C. Quantitative mass spectrometry-based techniques for clinical use: biomarker identification and quantification. J Chromatogr B Analyt Technol Biomed Life Sci. 2009 May 1;877(13):1240-9. Epub 2008 Nov 18. Review.
265. Skrzynia C, Demo EM, Baxter SM. Genetic counseling and testing for hypertrophic cardiomyopathy: an adult perspective. J Cardiovasc Transl Res. 2009 Dec;2(4):493-9. **NO ACCESS**
266. Snead ML, Slavkin HC. Science is the fuel for the engine of technology and clinical practice. J Am Dent Assoc. 2009 Sep;140 Suppl 1:17S-24S.
267. Srinivasan B, Li Y, Jing Y, Xu Y, Yao X, Xing C, Wang JP. A detection system based on giant magnetoresistive sensors and high-moment magnetic nanoparticles demonstrates zeptomole sensitivity: potential for personalized medicine. Angew Chem Int Ed Engl. 2009;48(15):2764-7.
268. Srivastava PK, Callahan MK, Mauri MM. Treating human cancers with heat shock protein-peptide complexes: the road ahead. Expert Opin Biol Ther. 2009 Feb;9(2):179-86.
269. Stimson NF. Personalized medicine: selected web resources. Dialogues Clin Neurosci. 2009;11(4):464-9. **NO ACCESS**
270. Strimpakos AS, Syrigos KN, Saif MW. Pharmacogenetics in pancreatic cancer. Highlights from the 45th ASCO annual meeting. Orlando, FL, USA. May 29-June 2, 2009. JOP. 2009 Jul 6;10(4):357-60.
271. Swan M. Emerging patient-driven health care models: an examination of health social networks, consumer personalized medicine and quantified self-tracking. Int J Environ Res Public Health. 2009 Feb;6(2):492-525.
272. Szafran AT, Hartig S, Sun H, Uray IP, Szwarc M, Shen Y, Mediwala SN, Bell J, McPhaul MJ, Mancini MA, Marcelli M. Androgen receptor mutations associated with androgen insensitivity syndrome: a high content analysis approach leading to personalized medicine. PLoS One. 2009 Dec 9;4(12):e8179.
273. Szantai E, Elek Z, Guttman A, Sasvari-Szekely M. Candidate gene copy number analysis by PCR and multicapillary electrophoresis. Electrophoresis. 2009 Apr;30(7):1098-101.
274. Tailoring cancer therapy - validating basic science with the 'supertrial'. Mol Oncol. 2009 Dec;3(5-6):389-91. **NO ACCESS**
275. Tan DS, Thomas GV, Garrett MD, Banerji U, de Bono JS, Kaye SB, Workman P. Biomarker-driven early clinical trials in oncology: a paradigm shift in drug development. Cancer J. 2009 Sep-Oct;15(5):406-20. Review. **NO ACCESS**
276. Tasciotti E, Sakamoto J, Ferrari M. Conference scene: nanotechnology and medicine: the next big thing is really small. Nanomedicine (Lond). 2009 Aug;4(6):619-21.
277. Thieffry D. [Systems biology: from molecular networks to personalized medicine]. Med Sci (Paris). 2009 Jun-Jul;25(6-7):547-8. French.
278. Topol E. Exciting interventions in individualized medicine. An interview with Eric Topol, MD, of the Scripps Translational Science Institute by Elaine Musgrave, PhD. Clin Transl Sci. 2009 Dec;2(6):392-3. doi: 10.1111/j.1752-8062.2009.00160.x. **NO ACCESS**
279. Toussaint NC, Kohlbacher O. OptiTope--a web server for the selection of an optimal set of peptides for epitope-based vaccines. Nucleic Acids Res. 2009 Jul 1;37(Web Server issue):W617-22.
280. Tripathy D. HER2 status and breast cancer therapy: recent advances. F1000 Med Rep. 2009 Mar 17;1. pii: 20.
281. Tromp G, Kuivaniemi H. Developments in genomics to improve understanding, diagnosis and management of aneurysms and peripheral artery disease. Eur J Vasc Endovasc Surg. 2009 Dec;38(6):676-82. Epub 2009 Sep 26. Review.
282. True MW. Circulating biomarkers of glycemia in diabetes management and implications for personalized medicine. J Diabetes Sci Technol. 2009 Jul 1;3(4):743-7. Review.
283. Tsujimoto G. [Cutting edge of drug discovery based on genomic science]. Nippon Rinsho. 2009 Jun;67(6):1170-4. Review. Japanese.
284. Ueno T, Toi M. [Biomarker]. Gan To Kagaku Ryoho. 2009 Jan;36(1):15-20. Japanese.
285. Ulrich-Merzenich G, Panek D, Zeitler H, Wagner H, Vetter H. New perspectives for synergy research with the "omic"-technologies. Phytomedicine. 2009 Jun;16(6-7):495-508. doi: 10.1016/j.phymed.2009.04.001. Review. **NO ACCESS**
286. van Wietmarschen H, Yuan K, Lu C, Gao P, Wang J, Xiao C, Yan X, Wang M, Schroën J, Lu A, Xu G, van der Greef J. Systems biology guided by Chinese medicine reveals new markers for sub-typing rheumatoid arthritis patients. J Clin Rheumatol. 2009 Oct;15(7):330-7.
287. Vandenbroeck K, Comabella M, Tolosa E, Goertsches R, Brassat D, Hintzen R, Infante-Duarte C, Favorov A, Escorza S, Palacios R, Oksenberg JR, Villoslada P. United Europeans for development of pharmacogenomics in multiple sclerosis network. Pharmacogenomics. 2009 May;10(5):885-94. **NO ACCESS**
288. Verstuyft C, Simon T, Kim RB. Personalized medicine and antiplatelet therapy: ready for prime time? Eur Heart J. 2009 Aug;30(16):1943-63.
289. Verweij CL. Transcript profiling towards personalised medicine in rheumatoid arthritis. Neth J Med. 2009 Dec;67(11):364-71. Review. **NO ACCESS**
290. Vesely R. Getting personal. Advances in biotechnology and the promise of personalized medicine help drive record investment in 2009. Mod Healthc. 2010 Feb 1;40(5):26-8. **NO ACCESS**
291. Villoslada P, Steinman L, Baranzini SE. Systems biology and its application to the understanding of neurological diseases. Ann Neurol. 2009 Feb;65(2):124-39. Review.
292. Vosslamber S, van Baarsen LG, Verweij CL. Pharmacogenomics of IFN-beta in multiple sclerosis: towards a personalized medicine approach. Pharmacogenomics. 2009 Jan;10(1):97-108. Review.
293. Wakelee H, Loo BW Jr, Kernstine KH, Putnam JB Jr, Edelman MJ, Vokes EE, Schiller JH, Baas P, Saijo N, Adjei A, Goss G, Choy H, Gandara DR. Cooperative group research efforts in thoracic malignancies 2009: a review from the 10th Annual International Lung Cancer Congress. Clin Lung Cancer. 2009 Nov;10(6):395-404. **NO ACCESS**
294. Waldman SA, Kraft WK, Nelson TJ, Terzic A. Clinical pharmacology: a paradigm for individualized medicine. Biomark Med. 2009 Dec;3(6):679-84. doi: 10.2217/bmm.09.76. **NO ACCESS**
295. Waldman SA, Kraft WK, Nelson TJ, Terzic A. Experimental therapeutics: a paradigm for personalized medicine. Clin Transl Sci. 2009 Dec;2(6):436-8.
296. Waldman SA, Terzic A. Molecular diagnostics. At the nexus of individualized medicine, health care delivery, and public policy. Clin Transl Sci. 2009 Feb;2(1):6-8. doi: 10.1111/j.1752-8062.2008.00067.x. **NO ACCESS**
297. Walk EE. The role of pathologists in the era of personalized medicine. Arch Pathol Lab Med. 2009 Apr;133(4):605-10.
298. Wang DE. MicroRNA Regulation and its Biological Significance in Personalized Medicine and Aging. Curr Genomics. 2009 May;10(3):143.
299. Wang LB, Seidman JG, Seidman CE. [Using molecular genetics to guide the diagnosis and treatment of hypertrophic cardiomyopathy]. Zhonghua Xin Xue Guan Bing Za Zhi. 2009 Dec;37(12):1063-8. Chinese.
300. Wilke RA, Simpson RU, Mukesh BN, Bhupathi SV, Dart RA, Ghebranious NR, McCarty CA. Genetic variation in CYP27B1 is associated with congestive heart failure in patients with hypertension. Pharmacogenomics. 2009 Nov;10(11):1789-97.
301. Wilson ID. Drugs, bugs, and personalized medicine: pharmacometabonomics enters the ring. Proc Natl Acad Sci U S A. 2009 Aug 25;106(34):14187-8.
302. Wingler K, Schmidt HH. Good stress, bad stress--the delicate balance in the vasculature. Dtsch Arztebl Int. 2009 Oct;106(42):677-84. doi: 10.3238/arztebl.2009.0677.
303. Wist AD, Berger SI, Iyengar R. Systems pharmacology and genome medicine: a future perspective. Genome Med. 2009 Jan 22;1(1):11.
304. Xu M, Fujita D, Hanagata N. Perspectives and challenges of emerging single-molecule DNA sequencing technologies. Small. 2009 Dec;5(23):2638-49. Review. **NO ACCESS**
305. Yang CH, Chuang LY, Cheng YH, Wen CH, Chang HW. Dynamic programming for single nucleotide polymorphism ID identification in systematic association studies. Kaohsiung J Med Sci. 2009 Apr;25(4):165-76.
306. Yang L, Chen J, He L. Harvesting candidate genes responsible for serious adverse drug reactions from a chemical-protein interactome. PLoS Comput Biol. 2009 Jul;5(7):e1000441.
307. Yang MQ, Athey BD, Arabnia HR, Sung AH, Liu Q, Yang JY, Mao J, Deng Y. High-throughput next-generation sequencing technologies foster new cutting-edge computing techniques in bioinformatics. BMC Genomics. 2009 Jul 7;10 Suppl 1:I1.
308. Yap TA, Carden CP, Kaye SB. Beyond chemotherapy: targeted therapies in ovarian cancer. Nat Rev Cancer. 2009 Mar;9(3):167-81. Review. **NO ACCESS**
309. Yin W. R&D policy, agency costs and innovation in personalized medicine. J Health Econ. 2009 Sep;28(5):950-62.
310. Zhang DY, Ye F, Gao L, Liu X, Zhao X, Che Y, Wang H, Wang L, Wu J, Song D, Liu W, Xu H, Jiang B, Zhang W, Wang J, Lee P. Proteomics, pathway array and signaling network-based medicine in cancer. Cell Div. 2009 Oct 28;4:20.
311. Zhang W, Catenacci DV, Duan S, Ratain MJ. A survey of the population genetic variation in the human kinome. J Hum Genet. 2009 Aug;54(8):488-92.
312. Zheng J, Jaffray D, Allen C. Quantitative CT imaging of the spatial and temporal distribution of liposomes in a rabbit tumor model. Mol Pharm. 2009 Mar-Apr;6(2):571-80. **NO ACCESS**
313. Zoon CK, Starker EQ, Wilson AM, Emmert-Buck MR, Libutti SK, Tangrea MA. Current molecular diagnostics of breast cancer and the potential incorporation of microRNA. Expert Rev Mol Diagn. 2009 Jul;9(5):455-67. Review.
314. Zrazhevskiy P, Gao X. Multifunctional Quantum Dots for Personalized Medicine. Nano Today. 2009 Oct 5;4(5):414-428.

**No author stated:**

1. A favourable (molecular) signal for personalised medicine. Lancet. 2009 Jan 3;373(9657):2.

**2008**

1. Abrahams E. Right drug-right patient-right time: personalized medicine coalition. Clin Transl Sci. 2008 May;1(1):11-2.
2. Al-Hoqail I, Salem R, Monshi M, Zeilinger C, Zimmerman V, Zaher A. Personalized medicine: a world of opportunities in skin diseases. J Eur Acad Dermatol Venereol. 2008 Nov;22(11):1386-7. **NO ACCESS**
3. Allegra CJ, Benedetti JK. Don Quixote and the quest for personalized medicine. J Clin Oncol. 2008 Jun 1;26(16):2619-20.
4. Allison M. Is personalized medicine finally arriving? Nat Biotechnol. 2008 May;26(5):509-17. Erratum in: Nat Biotechnol. 2008 Jul;26(7):831.
5. Amur S, Frueh FW, Lesko LJ, Huang SM. Integration and use of biomarkers in drug development, regulation and clinical practice: a US regulatory perspective. Biomark Med. 2008 Jun;2(3):305-11. **NO ACCESS**
6. Anderson GD. Pharmacokinetic, pharmacodynamic, and pharmacogenetic targeted therapy of antiepileptic drugs. Ther Drug Monit. 2008 Apr;30(2):173-80. Review.
7. Apetoh L, Mignot G, Panaretakis T, Kroemer G, Zitvogel L. Immunogenicity of anthracyclines: moving towards more personalized medicine. Trends Mol Med. 2008 Apr;14(4):141-51. Epub 2008 Mar 18. Review.
8. Arber N, Levin B. Chemoprevention of colorectal neoplasia: the potential for personalized medicine. Gastroenterology. 2008 Apr;134(4):1224-37. Review.
9. Asleh R, Blum S, Kalet-Litman S, Alshiek J, Miller-Lotan R, Asaf R, Rock W, Aviram M, Milman U, Shapira C, Abassi Z, Levy AP. Correction of HDL dysfunction in individuals with diabetes and the haptoglobin 2-2 genotype. Diabetes. 2008 Oct;57(10):2794-800.
10. AVMA Group Health and Life Insurance Trust. Getting the right drug at the right dosage. Personalized medicine brings precision to drug treatment. J Am Vet Med Assoc. 2008 Oct 15;233(8):1212-3.
11. Awasthi S. Next generation of human vaccines: what does the future hold? Hum Vaccin. 2008 Sep-Oct;4(5):344-6.
12. Baek S, Moon H, Ahn H, Kodell RL, Lin CJ, Chen JJ. Identifying high-dimensional biomarkers for personalized medicine via variable importance ranking. J Biopharm Stat. 2008;18(5):853-68. **NO ACCESS**
13. Bains W. Truly personalised medicine: self-experimentation in medical discovery. Med Hypotheses. 2008;70(4):714-8. **NO ACCCESS**
14. Ballantyne C. In tough times, personalized medicine needs specific partners. Nat Med. 2008 Dec;14(12):1294.
15. Bandrés Moya F, Delgado Bueno S, Bandrés Hernández S. [Ethical and legal implications of biomedical research]. Med Clin (Barc). 2008 Dec;131 Suppl 5:87-90. Spanish.
16. Barba I, Fernandez-Montesinos R, Garcia-Dorado D, Pozo D. Alzheimer's disease beyond the genomic era: nuclear magnetic resonance (NMR) spectroscopy-based metabolomics. J Cell Mol Med. 2008 Sep-Oct;12(5A):1477-85. Epub 2008 Jun 28. Review.
17. Barbet J, Kraeber-Bodéré F, Chatal JF. What can be expected from nuclear medicine tomorrow? Cancer Biother Radiopharm. 2008 Aug;23(4):483-504. Review. **NO ACCESS**
18. Benner SA, Hoshika S, Sukeda M, Hutter D, Leal N, Yang Z, Chen F. Synthetic biology for improved personalized medicine. Nucleic Acids Symp Ser (Oxf). 2008;(52):243-4.
19. Bixby DL, Talpaz M. Efficacy of various doses and schedules of second-generation tyrosine kinase inhibitors. Clin Lymphoma Myeloma. 2008 Mar;8 Suppl 3:S95-S106. Review. **NO ACCESS**
20. Bläuer M, Heinonen PK, Rovio P, Ylikomi T. Effects of tamoxifen and raloxifene on normal human endometrial cells in an organotypic in vitro model. Eur J Pharmacol. 2008 Sep 11;592(1-3):13-8. **NO ACCESS**
21. Boku N. Chemotherapy for metastatic gastric cancer in Japan. Int J Clin Oncol. 2008 Dec;13(6):483-7. Epub 2008 Dec 18. Review.
22. Borangíu T, Purcarea V. The future of healthcare--information based medicine. J Med Life. 2008 Apr-Jun;1(2):233-7.
23. Braff JP, Chatterjee B, Hochman M, Kelton T, Kennington J, Kolavala C, Layman K, Parver C, Selby MC, Washlick JR, Wolf R. Patient-tailored medicine, Part one: the impact of race and genetics on medicine. J Health Life Sci Law. 2008 Oct;2(1):1-3, 5-36. **NO ACCESS**
24. Bridges SL Jr. Personalized medicine in rheumatoid arthritis: hopes and challenges. Bull NYU Hosp Jt Dis. 2007;65(3):174-7. Review. Erratum in: Bull NYU Hosp Jt Dis. 2008;66(1):72.
25. Cai W, Niu G, Chen X. Imaging of integrins as biomarkers for tumor angiogenesis. Curr Pharm Des. 2008;14(28):2943-73. Review. **NO ACCESS**
26. Cassier PA, Dufresne A, El Sayadi H, Pissaloux D, Alberti L, DecouvelaereAV, Ranchere D, Ray-Coquard I, Blay JY. [Targeted therapy of sarcomas]. Bull Cancer. 2008 Oct;95(10):963-74. French.
27. Ceglarek U, Leichtle A, Brügel M, Kortz L, Brauer R, Bresler K, Thiery J, Fiedler GM. Challenges and developments in tandem mass spectrometry based clinical metabolomics. Mol Cell Endocrinol. 2009 Mar 25;301(1-2):266-71.
28. Celi LA, Hinske LC, Alterovitz G, Szolovits P. An artificial intelligence tool to predict fluid requirement in the intensive care unit: a proof-of-concept study. Crit Care. 2008;12(6):R151.
29. Chaudhuri JP, Karamanov S, Paulraj P, McGill JR, Walther J. Identification of parental chromosomes involved in translocations BCR-ABL, t(9;22) and PML-RARA, t(15;17). Anticancer Res. 2008 Nov-Dec;28(6A):3573-8. Review.
30. Cheah PY. Recent advances in colorectal cancer genetics and diagnostics. Crit Rev Oncol Hematol. 2009 Jan;69(1):45-55. Epub 2008 Sep 6. Review.
31. Chen L, Qin S, Xie J, Tang J, Yang L, Shen W, Zhao X, Du J, He G, Feng G, He L, Xing Q. Genetic polymorphism analysis of CYP2C19 in Chinese Han populations from different geographic areas of mainland China. Pharmacogenomics. 2008 Jun;9(6):691-702. **NO ACCESS**
32. Christie JD. The interleukin-6 gene and critical illness: is inflammatory gene variation the key to personalized medicine in the intensive care unit? Crit Care Med. 2008 May;36(5):1647-9. **NO ACCESS**
33. Collins F. Francis Collins interview. Departing U.S. genome institute director takes stock of personalized medicine. Interview by Jocelyn Kaiser. Science. 2008 Jun 6;320(5881):1272. **NO ACCESS**
34. Confalonieri M. [Life sciences: a new area of action for the physician]. Recenti Prog Med. 2008 Mar;99(3):129-33. Italian.
35. Cross J. DxS Ltd. Pharmacogenomics. 2008 Apr;9(4):463-7. Review. **NO ACCESS**
36. Danila MI, Hughes LB, Bridges SL. Pharmacogenetics of etanercept in rheumatoid arthritis. Pharmacogenomics. 2008 Aug;9(8):1011-5. Review.
37. Danilov SM, Balyasnikova IV, Albrecht RF 2nd, Kost OA. Simultaneous determination of ACE activity with 2 substrates provides information on the status of somatic ACE and allows detection of inhibitors in human blood. J Cardiovasc Pharmacol. 2008 Jul;52(1):90-103.
38. David SP, Munafò MR. Genetic variation in the dopamine pathway and smoking cessation. Pharmacogenomics. 2008 Sep;9(9):1307-21. Review. **NO ACCESS**
39. de Leon J, Greenlee B, Barber J, Sabaawi M, Singh NN. Practical guidelines for the use of new generation antipsychotic drugs (except clozapine) in adult individuals with intellectual disabilities. Res Dev Disabil. 2009 Jul-Aug;30(4):613-69. Epub 2008 Dec 11. Review. **NO ACCESS**
40. de Leon J. Pharmacogenomics: the promise of personalized medicine for CNS disorders. Neuropsychopharmacology. 2009 Jan;34(1):159-72. Epub 2008 Sep 17. Review.
41. de Leon J. The future (or lack of future) of personalized prescription in psychiatry. Pharmacol Res. 2009 Feb;59(2):81-9. Epub 2008 Oct 17. Review.
42. De Paoli P. Future of biobanking in microbiology for medical research. Future Microbiol. 2008 Feb;3(1):79-86. Review. **NO ACCESS**
43. Del Barrio J. [From human genome to individualized medicine]. Rev Derecho Genoma Hum. 2008 Jul-Dec;(28):15-29. Spanish.
44. Del Barrio J. [From human genome to individualized medicine]. Rev Derecho Genoma Hum. 2008 Jul-Dec;(28):15-29. Spanish.
45. Doi K, Okamoto K, Tokunaga K, Fujita T, Noiri E. Genome study of kidney disease in the age of post genome-sequencing. Endocr Metab Immune Disord Drug Targets. 2008 Sep;8(3):173-83. Review. **NO ACCESS**
46. Dowsett M, Dunbier AK. Emerging biomarkers and new understanding of traditional markers in personalized therapy for breast cancer. Clin Cancer Res. 2008 Dec 15;14(24):8019-26. Review. **NO ACCESS**
47. Draisma HH, Reijmers TH, Bobeldijk-Pastorova I, Meulman JJ, Estourgie-Van Burk GF, Bartels M, Ramaker R, van der Greef J, Boomsma DI, Hankemeier T. Similarities and differences in lipidomics profiles among healthy monozygotic twin pairs. OMICS. 2008 Mar;12(1):17-31. **NO ACCESS**
48. Du P, Lin SM, Yang MQ, Yang JY. 2009 and beyond: the decade of personalised medicine. Int J Comput Biol Drug Des. 2008;1(4):329-33. **NO ACCCESS**
49. Eckelman WC, Reba RC, Kelloff GJ. Targeted imaging: an important biomarker for understanding disease progression in the era of personalized medicine. Drug Discov Today. 2008 Sep;13(17-18):748-59. Epub 2008 Jul 24. Review. **NO ACCESS**
50. Elias DJ, Topol EJ. Warfarin pharmacogenomics: a big step forward for individualized medicine: enlightened dosing of warfarin. Eur J Hum Genet. 2008 May;16(5):532-4. doi: 10.1038/sj.ejhg.5201945.
51. Eng-Wong J, Zujewski JA. Current NCI-sponsored Cooperative Group trials of endocrine therapies in breast cancer. Cancer. 2008 Feb 1;112(3 Suppl):723-9.
52. Enzmann H, Lütz J. [European incentives for orphan medicinal products]. Bundesgesundheitsblatt Gesundheitsforschung Gesundheitsschutz. 2008 May;51(5):500-8. doi: 10.1007/s00103-008-0523-8. Review. German.
53. Eschrich SA, Hoerter AM, Bloom GC, Fenstermacher DA. Tissue-specific RMA models to incrementally normalize Affymetrix GeneChip data. Conf Proc IEEE Eng Med Biol Soc. 2008;2008:2419-22. **NO ACCESS**
54. Esfandyarpour H, Zheng B, Pease RF, Davis RW. Structural optimization for heat detection of DNA thermosequencing platform using finite element analysis. Biomicrofluidics. 2008 Apr 11;2(2):24102.
55. Farragher SM, Tanney A, Kennedy RD, Paul Harkin D. RNA expression analysis from formalin fixed paraffin embedded tissues. Histochem Cell Biol. 2008 Sep;130(3):435-45. Epub 2008 Aug 5. Review.
56. Foekens JA, Wang Y, Martens JW, Berns EM, Klijn JG. The use of genomic tools for the molecular understanding of breast cancer and to guide personalized medicine. Drug Discov Today. 2008 Jun;13(11-12):481-7. Review. **NO ACCESS**
57. Freeman SD, Jovanovic JV, Grimwade D. Development of minimal residual disease-directed therapy in acute myeloid leukemia. Semin Oncol. 2008 Aug;35(4):388-400. Review.
58. Frey LJ, Maojo V, Mitchell JA. Bioinformatics linkage of heterogeneous clinical and genomic information in support of personalized medicine. Yearb Med Inform. 2007:98-105. Erratum in: Yearb Med Inform. 2008:19.
59. Fritze J. Drug development in neuropsychopharmacology. Eur Arch Psychiatry Clin Neurosci. 2008 Mar;258 Suppl 1:3-5. **NO ACCESS**
60. Frost JJ. Molecular imaging to biomarker development in neuroscience. Ann N Y Acad Sci. 2008 Nov;1144:251-5. **NO ACCESS**
61. Gambacorti-Passerini C. Part I: Milestones in personalised medicine--imatinib. Lancet Oncol. 2008 Jun;9(6):600.
62. Gelmon K. Part II: Milestones in personalised medicine--trastuzumab. Lancet Oncol. 2008 Jul;9(7):698.
63. Gladding P. Personalised medicine in New Zealand. N Z Med J. 2008 Apr 18;121(1272):10-3. **NO ACCCESS**
64. Glinskii VG, Glinsky GV. Emerging genomic technologies and the concept of personalized medicine. Cell Cycle. 2008 Aug;7(15):2278-85.
65. Goldknopf IL. Blood-based proteomics for personalized medicine: examples from neurodegenerative disease. Expert Rev Proteomics. 2008 Feb;5(1):1-8.
66. Gómez de la Concha E. [Address by his Excellency Emilio Gómez de la Concha, Academic of Number of the National Royal Academy of Medicine, "Personalized Medicine: the new Medicine of the 21st Century]. An R Acad Nac Med (Madr). 2008;125(3):527-30. Spanish.
67. Gotoh N. Feedback inhibitors of the epidermal growth factor receptor signaling pathways. Int J Biochem Cell Biol. 2009 Mar;41(3):511-5. Epub 2008 Aug 9. Review.
68. Grassi R, Lagalla R, Rotondo A. Genomics, proteomics, MEMS and SAIF: which role for diagnostic imaging? Radiol Med. 2008 Sep;113(6):775-8. Epub 2008 Jul 25. English, Italian. **NO ACCESS**
69. Guidi GC, Lippi G. Will "personalized medicine" need personalized laboratory approach? Clin Chim Acta. 2009 Feb;400(1-2):25-9.
70. Gurbel PA, Antonino MJ, Bliden KP, Dichiara J, Suarez TA, Singla A, Tantry US. Platelet reactivity to adenosine diphosphate and long-term ischemic event occurrence following percutaneous coronary intervention: a potential antiplatelet therapeutic target. Platelets. 2008 Dec;19(8):595-604.
71. Habermann JK, Bader FG, Franke C, Zimmermann K, Gemoll T, Fritzsche B, Ried T, Auer G, Bruch HP, Roblick UJ. From the genome to the proteome--biomarkers in colorectal cancer. Langenbecks Arch Surg. 2008 Jan;393(1):93-104. Epub 2007 Oct 16. Review.
72. Hall JL. Translating genetic discoveries to improvements in cardiovascular care: the path to personalized medicine. J Cardiovasc Transl Res. 2008 Mar;1(1):37-40. Epub 2008 Feb 8. Review. **NO ACCESS**
73. Hatzimouratidis K, Hatzichristou DG. Looking to the future for erectile dysfunction therapies. Drugs. 2008;68(2):231-50. Review. **NO ACCESS**
74. Hewel JA, Emili A. High-resolution biomarker discovery: Moving from large-scale proteome profiling to quantitative validation of lead candidates. Proteomics Clin Appl. 2008 Oct;2(10-11):1422-34. doi: 10.1002/prca.200800030.
75. Holmes E, Wilson ID, Nicholson JK. Metabolic phenotyping in health and disease. Cell. 2008 Sep 5;134(5):714-7.
76. Holsinger FC. Swing of the pendulum: optimizing functional outcomes in larynx cancer. Curr Oncol Rep. 2008 Mar;10(2):170-5. Review.
77. Hong H, Sun J, Cai W. Anatomical and molecular imaging of skin cancer. Clin Cosmet Investig Dermatol. 2008 Oct 7;1:1-17.
78. Hong H, Sun J, Cai W. Radionuclide-Based Cancer Imaging Targeting the Carcinoembryonic Antigen. Biomark Insights. 2008 Sep 23;3:435-451.
79. Hou C, Herr AE. Clinically relevant advances in on-chip affinity-based electrophoresis and electrochromatography. Electrophoresis. 2008 Aug;29(16):3306-19. Review.
80. Hulse NC, Taylor DP, Wood G, Haug PJ. Analysis of family health history data collection patterns in consumer-oriented Web-based tools. AMIA Annu Symp Proc. 2008 Nov 6:982. **NO ACCESS**
81. Hutchinson L, DeVita VT Jr. The era of personalized medicine: back to basics. Nat Clin Pract Oncol. 2008 Nov;5(11):623.
82. Ideker T, Sharan R. Protein networks in disease. Genome Res. 2008 Apr;18(4):644-52. Review.
83. Ikeda S, Sasazuki S, Natsukawa S, Shaura K, Koizumi Y, Kasuga Y, Ohnami S, Sakamoto H, Yoshida T, Iwasaki M, Tsugane S. Screening of 214 single nucleotide polymorphisms in 44 candidate cancer susceptibility genes: a case-control study on gastric and colorectal cancers in the Japanese population. Am J Gastroenterol. 2008 Jun;103(6):1476-87.
84. Ikediobi ON. Personalized medicine: are we there yet? Pharmacogenomics J. 2009 Apr;9(2):85.
85. Ikediobi ON. Somatic pharmacogenomics in cancer. Pharmacogenomics J. 2008 Oct;8(5):305-14. Epub 2008 Aug 5. Review. **NO ACCESS**
86. Issa AM. Evaluating the value of genomic diagnostics: implications for clinical practice and public policy. Adv Health Econ Health Serv Res. 2008;19:191-206. Review.
87. Jain KK. Drug delivery systems - an overview. Methods Mol Biol. 2008;437:1-50. Review. **NO ACCESS**
88. Jain KK. Nanomedicine: application of nanobiotechnology in medical practice. Med Princ Pract. 2008;17(2):89-101. Epub 2008 Feb 19. Review.
89. Janku F, Bird B. Personalized medicine in advanced non-small-cell lung cancer: reality or wishful thinking? J Clin Oncol. 2008 Dec 1;26(34):5651; author reply 5651-2.
90. Janssens AC, van Duijn CM. Genome-based prediction of common diseases: advances and prospects. Hum Mol Genet. 2008 Oct 15;17(R2):R166-73. Review.
91. Jones J, Pantuck AJ. Genomics and proteomics in renal cell carcinoma: diagnosis, prognosis, and treatment selection. Curr Urol Rep. 2008 Jan;9(1):9-14. Review. **NO ACCCESS**
92. Jørgensen JT. Are we approaching the post-blockbuster era? Pharmacodiagnostics and rational drug development. Expert Rev Mol Diagn. 2008 Nov;8(6):689-95. Review. **NO ACCESS**
93. Judge DP, Johnson NM. Genetic evaluation of familial cardiomyopathy. J Cardiovasc Transl Res. 2008 Jun;1(2):144-54. Epub 2008 Apr 22. Review. **NO ACCESS**
94. Kang SP, Martel M, Harris LN. Triple negative breast cancer: current understanding of biology and treatment options. Curr Opin Obstet Gynecol. 2008 Feb;20(1):40-6. doi: 10.1097/GCO.0b013e3282f40de9. Review. PubMed PMID: 18197004. **NO ACCCESS**
95. Kassis AI, Korideck H, Wang K, Pospisil P, Adelstein SJ. Novel prodrugs for targeting diagnostic and therapeutic radionuclides to solid tumors. Molecules. 2008 Feb 18;13(2):391-404. Review. **NO ACCESS**
96. Katsanis SH, Javitt G, Hudson K. Public health. A case study of personalized medicine. Science. 2008 Apr 4;320(5872):53-4. Erratum in: Science.2008 Apr 18;320(5874):316. **NO ACCESS**
97. Khlusov IA, Zagrebin LV, Shestov SS, Itin VI, Sedoi VS, Feduschak TA, Terekhova OG, Magaeva AA, Naiden EP, Antipov SA, Puchkovskaya ES, Slepchenko GB, Sukhikh GT. Colony-forming activity of unipotent hemopoietic precursors under the effect of nanosized ferrites in a constant magnetic field in vitro. Bull Exp Biol Med. 2008 Jan;145(1):151-7. **NO ACCESS**
98. Kim FJ, Campagna A, Khandrika L, Koul S, Byun SS, vanBokhoven A, Moore EE, Koul H. Individualized medicine for renal cell carcinoma: establishment of primary cell line culture from surgical specimens. J Endourol. 2008 Oct;22(10):2361-6. doi: 10.1089/end.2008.9703. Erratum in: J Endourol. 2009 Jan;23(1):181.
99. Klein CA. The direct molecular analysis of metastatic precursor cells in breast cancer: a chance for a better understanding of metastasis and for personalised medicine. Eur J Cancer. 2008 Dec;44(18):2721-5. Epub 2008 Nov 18. Review.
100. Klonoff DC. Personalized medicine for diabetes. J Diabetes Sci Technol. 2008 May;2(3):335-41.
101. Komen J, Wolbers F, Franke HR, Andersson H, Vermes I, van den Berg A. Viability analysis and apoptosis induction of breast cancer cells in a microfluidic device: effect of cytostatic drugs. Biomed Microdevices. 2008 Oct;10(5):727-37.
102. Koomen JM, Haura EB, Bepler G, Sutphen R, Remily-Wood ER, Benson K, Hussein M, Hazlehurst LA, Yeatman TJ, Hildreth LT, Sellers TA, Jacobsen PB, Fenstermacher DA, Dalton WS. Proteomic contributions to personalized cancer care. Mol Cell Proteomics. 2008 Oct;7(10):1780-94. Epub 2008 Jul 29. Review.
103. Krusche CA, von Wasielewski R, Rüschoff J, Fisseler-Eckhoff A, Kreipe HH. [Interlaboratory trials for quality assurance of breast cancer biomarkers in Germany]. Pathologe. 2008 Jul;29(4):315-20. German.
104. Kulkarni H, Agan BK, Marconi VC, O'Connell RJ, Camargo JF, He W, Delmar J, Phelps KR, Crawford G, Clark RA, Dolan MJ, Ahuja SK. CCL3L1-CCR5 genotype improves the assessment of AIDS Risk in HIV-1-infected individuals. PLoS One. 2008 Sep 8;3(9):e3165.
105. Kumar A, Ajilore O. Magnetic resonance imaging and late-life depression: potential biomarkers in the era of personalized medicine. Am J Psychiatry. 2008 Feb;165(2):166-8.
106. Lam CW. A universal algorithm for de novo decrypting of heterozygous indel sequences: a tool for personalized medicine. Clin Chim Acta. 2008 Mar;389(1-2):7-13.
107. Lavallie ER, Dorner AJ, Burczynski ME. Use of ex vivo systems for biomarker discovery. Curr Opin Pharmacol. 2008 Oct;8(5):647-53. Epub 2008 Sep 19. Review. **NO ACCESS**
108. Lea P, Ling M. New molecular assays for cancer diagnosis and targeted therapy. Curr Opin Mol Ther. 2008 Jun;10(3):251-9. Review. **NO ACCESS**
109. Lee C, Morton CC. Structural genomic variation and personalized medicine. N Engl J Med. 2008 Feb 14;358(7):740-1.
110. Lee GH. The Kras2 oncogene and mouse lung carcinogenesis. Med Mol Morphol. 2008 Dec;41(4):199-203. Epub 2008 Dec 24. Review. **NO ACCESS**
111. Levy H, Young JH. Perspectives from the clinic: will the average physician embrace personalized medicine? Clin Pharmacol Ther. 2008 Mar;83(3):492-3. Epub 2008 Feb 6. Review.
112. Li NY, Verdolini K, Clermont G, Mi Q, Rubinstein EN, Hebda PA, Vodovotz Y. A patient-specific in silico model of inflammation and healing tested in acute vocal fold injury. PLoS One. 2008 Jul 30;3(7):e2789.
113. Li TK. Quantifying the risk for alcohol-use and alcohol-attributable health disorders: present findings and future research needs. J Gastroenterol Hepatol. 2008 Mar;23 Suppl 1:S2-8. Review. **NO ACCESS**
114. Lim LE, Rando TA. Technology insight: therapy for Duchenne muscular dystrophy-an opportunity for personalized medicine? Nat Clin Pract Neurol. 2008 Mar;4(3):149-58. Epub 2008 Feb 12. Review. **NO ACCESS**
115. Lin B, Wang J, Cheng Y. Recent Patents and Advances in the Next-Generation Sequencing Technologies. Recent Pat Biomed Eng. 2008;2008(1):60-67.
116. Lin H, Zhang Z, Zhang MQ, Ma B, Li M. ZOOM! Zillions of oligos mapped. Bioinformatics. 2008 Nov 1;24(21):2431-7.
117. Lin KM, Perlis RH, Wan YJ. Pharmacogenomic strategy for individualizing antidepressant therapy. Dialogues Clin Neurosci. 2008;10(4):401-8. Review. **NO ACCESS**
118. Lindemann K, Harbeck N, Lengyel E, Resau JH. A special key for unlocking the door to targeted therapies of breast cancer. ScientificWorldJournal. 2008 Sep 21;8:905-8.
119. Liotta L, Petricoin E. Nanomedicine--the power of proteins: a conversation with Lance Liotta and Emanual Petricoin. Interview by Barbara J Culliton. Health Aff (Millwood). 2008 Jul-Aug;27(4):w310-4.
120. Liu JP, Chow SC. Statistical issues on the diagnostic multivariate index assay for targeted clinical trials. J Biopharm Stat. 2008;18(1):167-82.
121. Llovet JM, Bruix J. Molecular targeted therapies in hepatocellular carcinoma. Hepatology. 2008 Oct;48(4):1312-27. Review.
122. Lubomirov R, Telenti A, Rotger M. [General concepts and study methods in pharmacogenetics]. Enferm Infecc Microbiol Clin. 2008 May;26 Suppl 6:4-9. Spanish. Erratum in: Enferm Infecc Microbiol Clin. 2008 Oct;26(8):536.
123. MacDonald IM. Personalized medicine: the translation of genetic knowledge to eye care. Can J Ophthalmol. 2008 Feb;43(1):15-7.
124. Maier W, Zobel A. Contribution of allelic variations to the phenotype of response to antidepressants and antipsychotics. Eur Arch Psychiatry Clin Neurosci. 2008 Mar;258 Suppl 1:12-20. doi: 10.1007/s00406-007-1004-z. Review.
125. Marian AJ. Surprises of the genome and "personalized" medicine. J Am Coll Cardiol. 2008 Jan 29;51(4):456-8.
126. Marrer E, Dieterle F. Biomarkers in oncology drug development: rescuers or troublemakers? Expert Opin Drug Metab Toxicol. 2008 Nov;4(11):1391-402. Review.
127. Martinez-Forero I, Pelaez A, Villoslada P. Pharmacogenomics of multiple sclerosis: in search for a personalized therapy. Expert Opin Pharmacother. 2008 Dec;9(17):3053-67. Review.
128. McCarty CA, Burmester JK, Mukesh BN, Patchett RB, Wilke RA. Intraocular pressure response to topical beta-blockers associated with an ADRB2 single-nucleotide polymorphism. Arch Ophthalmol. 2008 Jul;126(7):959-63.
129. McCarty CA, Chapman-Stone D, Derfus T, Giampietro PF, Fost N; Marshfield Clinic PMRP Community Advisory Group. Community consultation and communication for a population-based DNA biobank: the Marshfield clinic personalized medicine research project. Am J Med Genet A. 2008 Dec 1;146A(23):3026-33.
130. McCarty CA, Mukesh BN, Kitchner TE, Hubbard WC, Wilke RA, Burmester JK, Patchett RB. Intraocular pressure response to medication in a clinical setting: the Marshfield Clinic Personalized Medicine Research Project. J Glaucoma. 2008 Aug;17(5):372-7.
131. McGuire AL. 1000 Genomes on the Road to Personalized Medicine. Per Med. 2008;5(3):195-197.
132. Merrill JT. Is individualized medicine on the horizon for lupus? Curr Rheumatol Rep. 2008 Aug;10(4):257-8. **NO ACCCESS**
133. Meyer CR. Personalized medicine. Minn Med. 2008 May;91(5):4.
134. Michael H, Hogan J, Kel A, Kel-Margoulis O, Schacherer F, Voss N, Wingender E. Building a knowledge base for systems pathology. Brief Bioinform. 2008 Nov;9(6):518-31. Epub 2008 Dec 10. Review.
135. Mijatovic T, Ingrassia L, Facchini V, Kiss R. Na+/K+-ATPase alpha subunits as new targets in anticancer therapy. Expert Opin Ther Targets. 2008 Nov;12(11):1403-17. Review. **NO ACCESS**
136. Miles A, Loughlin M, Polychronis A. Evidence-based healthcare, clinical knowledge and the rise of personalised medicine. J Eval Clin Pract. 2008 Oct;14(5):621-49.
137. Miller A, Avidan N, Tzunz-Henig N, Glass-Marmor L, Lejbkowicz I, Pinter RY, Paperna T. Translation towards personalized medicine in Multiple Sclerosis. J Neurol Sci. 2008 Nov 15;274(1-2):68-75. Epub 2008 Sep 12. Review.
138. Miller TJ. Health care technology, paving the road to personalized medicine. Hosp Health Netw. 2008 May;82(5):suppl 13. **NO ACCESS**
139. Mizukami T. [Front-line drug discovery system for cancer]. Gan To Kagaku Ryoho. 2008 Dec;35(13):2293-300. Review. Japanese.
140. Modak A. Diagnostic breath tests in personalized medicine. Expert Rev Mol Diagn. 2008 Sep;8(5):563-4.
141. Mojica WD, Sykes DE, Conroy J, Gaile D, Fang X, Nowak N. A comparative analysis of two tissue procurement approaches for the genomic profiling of clinical colorectal cancer samples. Int J Colorectal Dis. 2008 Nov;23(11):1089-98.
142. Montagnana M, Lippi G, Franchini M, Targher G, Cesare Guidi G. Sudden cardiac death: prevalence, pathogenesis, and prevention. Ann Med. 2008;40(5):360-75. Review. **NO ACCESS**
143. Moroi SE, Heckenlively JR. Progress toward personalized medicine for age-related macular degeneration. Ophthalmology. 2008 Jun;115(6):925-6.
144. Morrow T. Personalized medicine complicates medical decision-making. Manag Care. 2008 Feb;17(2):59-60. **NO ACCESS**
145. Musgrave E. Transforming the FDA in the age of personalized medicine-interview with Ray Woosley. Clin Transl Sci. 2008 May;1(1):5.
146. Nagasaki K, Miki Y. Molecular prediction of the therapeutic response to neoadjuvant chemotherapy in breast cancer. Breast Cancer. 2008;15(2):117-20. Review.
147. Naylor S, Culbertson AW, Valentine SJ. Towards a systems level analysis of health and nutrition. Curr Opin Biotechnol. 2008 Apr;19(2):100-9. Epub 2008 Apr 2. Review. **NO ACCESS**
148. Nebert DW, Zhang G, Vesell ES. From human genetics and genomics to pharmacogenetics and pharmacogenomics: past lessons, future directions. Drug Metab Rev. 2008;40(2):187-224. Review.
149. Ng PC, Zhao Q, Levy S, Strausberg RL, Venter JC. Individual genomes instead of race for personalized medicine. Clin Pharmacol Ther. 2008 Sep;84(3):306-9. **NO ACCESS**
150. Ning B, Dial S, Sun Y, Wang J, Yang J, Guo L. Systematic and simultaneous gene profiling of 84 drug-metabolizing genes in primary human hepatocytes. J Biomol Screen. 2008 Mar;13(3):194-201.
151. Nishiyama M. [Genomic markers and anticancer chemotherapy]. Gan To Kagaku Ryoho. 2008 Feb;35(2):194-9. Japanese.
152. Nobori T. [Personalized medicine based on genomic information: the present status in terms of clinical application]. Rinsho Byori. 2008 May;56(5):387-94. Review. Japanese.
153. O'Donnell CJ, Nabel EG. Cardiovascular genomics, personalized medicine, and the National Heart, Lung, and Blood Institute: part I: the beginning of an era. Circ Cardiovasc Genet. 2008 Oct;1(1):51-7. Review.
154. Ohta S, Azuma Y, Shiraishi H, Noguchi Y, Izuhara K. [Establishment of a diagnostic system for allergic diseases aiming at personalized medicine--establishment of a novel ELISA system for SCCA]. Rinsho Byori. 2008 Nov;56(11):980-5. Japanese.
155. Olopade OI, Grushko TA, Nanda R, Huo D. Advances in breast cancer: pathways to personalized medicine. Clin Cancer Res. 2008 Dec 15;14(24):7988-99. Review. **NO ACCESS**
156. Orloff MS, Eng C. Genetic and phenotypic heterogeneity in the PTEN hamartoma tumour syndrome. Oncogene. 2008 Sep 18;27(41):5387-97. Review.
157. Oro AE. Dermatology in the postgenomic era: harnessing human variation for personalized medicine. Arch Dermatol. 2008 Mar;144(3):389-91.
158. Paige AJ, Brown R. Pharmaco(epi)genomics in ovarian cancer. Pharmacogenomics. 2008 Dec;9(12):1825-34. doi: 10.2217/14622416.9.12.1825. Review. **NO ACCCESS**
159. Pallanti S. Brain plasticity and brain stimulation in neuropsychiatry: toward individualized medicine. CNS Spectr. 2008 Apr;13(4):287-92. **NO ACCCESS**
160. Patel KK, Babyatsky MW. Medical education: a key partner in realizing personalized medicine in gastroenterology. Gastroenterology. 2008 Mar;134(3):656-61. **NO ACCESS**
161. Patrignani P, Tacconelli S, Capone ML. Risk management profile of etoricoxib: an example of personalized medicine. Ther Clin Risk Manag. 2008 Oct;4(5):983-97.
162. Personalized medicine means planning for your future health. Analyzing a patient's health risks and focusing on prevention and health planning are key components of the 21st century's new health care. Duke Med Health News. 2008 Nov;14(11):6-7. **NO ACCESS**
163. Personalized medicine: are we there yet? J Cardiovasc Transl Res. 2008 Mar;1(1):3-4. **NO ACCESS**
164. Pettersson E, Lundeberg J, Ahmadian A. Generations of sequencing technologies. Genomics. 2009 Feb;93(2):105-11. Epub 2008 Nov 21. Review.
165. Pharmaceutical innovation: possibilities and limits of personalised medicine. Bundesgesundheitsblatt Gesundheitsforschung Gesundheitsschutz. 2008 Jun;51(6):675-88.
166. Phillips KA, Liang SY, Van Bebber S; Canpers Research Group. Challenges to the translation of genomic information into clinical practice and health policy: Utilization, preferences and economic value. Curr Opin Mol Ther. 2008 Jun;10(3):260-6. Review.
167. Podolec J, Gajos G, Budziaszek Ł, Czaniecka M, Kleczkoska A, Zmudka' K. [New approach to interventional cardiology treatment with personalized medicine]. Przegl Lek. 2008;65(12):850-7. Review. Polish.
168. Poole-Wilson PA. Differences in European and North American approaches to the management of heart failure. Cardiol Clin. 2008 Feb;26(1):107-12, viii. Review. **NO ACCESS**
169. Prasher B, Negi S, Aggarwal S, Mandal AK, Sethi TP, Deshmukh SR, Purohit SG, Sengupta S, Khanna S, Mohammad F, Garg G, Brahmachari SK; Indian Genome Variation Consortium, Mukerji M. Whole genome expression and biochemical correlates of extreme constitutional types defined in Ayurveda. J Transl Med. 2008 Sep 9;6:48.
170. Province MA, Borecki IB. Gathering the gold dust: methods for assessing the aggregate impact of small effect genes in genomic scans. Pac Symp Biocomput. 2008:190-200.
171. Reynolds KK, Ramey-Hartung B, Jortani SA. The value of CYP2D6 and OPRM1 pharmacogenetic testing for opioid therapy. Clin Lab Med. 2008 Dec;28(4):581-98. Review. **NO ACCESS**
172. Rich SS. Approaching biomarker discovery through genomics. J Cardiovasc Transl Res. 2008 Mar;1(1):21-4. Epub 2008 Jan 26. Review.
173. Richmond TD. The current status and future potential of personalized diagnostics: Streamlining a customized process. Biotechnol Annu Rev. 2008;14:411-22. Review.
174. Roberts R. Genetics of premature myocardial infarction. Curr Atheroscler Rep. 2008 Jun;10(3):186-93. Review.
175. Roberts R. Personalized medicine: a reality within this decade. J Cardiovasc Transl Res. 2008 Mar;1(1):11-6. Epub 2008 Jan 26. Review.
176. Roden DM, Pulley JM, Basford MA, Bernard GR, Clayton EW, Balser JR, Masys DR. Development of a large-scale de-identified DNA biobank to enable personalized medicine. Clin Pharmacol Ther. 2008 Sep;84(3):362-9.
177. Rossi CR, De Salvo GL, Bonandini E, Mocellin S, Foletto M, Pasquali S, Pilati P, Lise M, Nitti D, Rizzo E, Montesco MC. Factors predictive of nonsentinel lymph node involvement and clinical outcome in melanoma patients with metastatic sentinel lymph node. Ann Surg Oncol. 2008 Apr;15(4):1202-10. Erratum in: Ann Surg Oncol. 2008 May;15(5):1552. **NO ACCESS**
178. Roth SM. Functional genomics and the path to personalized medicine. Exerc Sport Sci Rev. 2008 Apr;36(2):49-50. **NO ACCESS**
179. Roukos DH. Genetics and genome-wide association studies: surgery-guided algorithm and promise for future breast cancer personalized surgery. Expert Rev Mol Diagn. 2008 Sep;8(5):587-97. **NO ACCESS**
180. Roukos DH. Personal genomics and genome-wide association studies: novel discoveries but limitations for practical personalized medicine. Ann Surg Oncol. 2009 Mar;16(3):772-3.
181. Ruaño G. DNA collage and personalized medicine. Conn Med. 2008 Jun-Jul;72(6):322. **NO ACCESS**
182. Sáenz A, López de Munain A. [DNA arrays: a general overview and specific applications]. Med Clin (Barc). 2008 Apr 12;130(13):504-9. Review. Spanish.
183. Sai K, Sawada J, Minami H. [Irinotecan pharmacogenetics in Japanese cancer patients: roles of UGT1A1*6 and *28]. Yakugaku Zasshi. 2008 Apr;128(4):575-84. Review. Japanese.
184. Satoh J. [Recent progress in bioinformatics for microarray analysis]. Yakugaku Zasshi. 2008 Nov;128(11):1537-45. Review. Japanese.
185. Saver RS. In tepid defense of population health: physicians and antibiotic resistance. Am J Law Med. 2008;34(4):431-91. **NO ACCCESS**
186. Schrohl AS, Würtz S, Kohn E, Banks RE, Nielsen HJ, Sweep FC, Brünner N. Banking of biological fluids for studies of disease-associated protein biomarkers. Mol Cell Proteomics. 2008 Oct;7(10):2061-6. Epub 2008 Aug 1. Review.
187. Sevick-Muraca EM, Rasmussen JC. Molecular imaging with optics: primer and case for near-infrared fluorescence techniques in personalized medicine. J Biomed Opt. 2008 Jul-Aug;13(4):041303.
188. Sfar S, Chouchane L. [Human genome project: a federator program of genomic medicine]. Pathol Biol (Paris). 2008 May;56(3):170-5. Epub 2008 Mar 5. French.
189. Shai RM, Reichardt JK, Chen TC. Pharmacogenomics of brain cancer and personalized medicine in malignant gliomas. Future Oncol. 2008 Aug;4(4):525-34. Review. **NO ACCESS**
190. Signoretti S, Bratslavsky G, Waldman FM, Reuter VE, Haaga J, Merino M, Thomas GV, Pins MR, Libermann T, Gillespie J, Tomaszewski JE, Compton CC, Hruszkewycz A, Linehan WM, Atkins MB. Tissue-based research in kidney cancer: current challenges and future directions. Clin Cancer Res. 2008 Jun 15;14(12):3699-705. Review.
191. Snyderman R, Yoediono Z. Perspective: Prospective health care and the role of academic medicine: lead, follow, or get out of the way. Acad Med. 2008 Aug;83(8):707-14.
192. Somogy A. Evolution of pharmacogenomics. Proc West Pharmacol Soc. 2008;51:1-4. Review. **NO ACCCESS**
193. Stahl SM. Personalized medicine, pharmacogenomics, and the practice ofpsychiatry: on the threshold of predictive therapeutics in psychopharmacology? CNS Spectr. 2008 Feb;13(2):115-8.
194. Su Z, Hong H, Fang H, Shi L, Perkins R, Tong W. Very Important Pool (VIP) genes--an application for microarray-based molecular signatures. BMC Bioinformatics. 2008 Aug 12;9 Suppl 9:S9.
195. Szafran AT, Szwarc M, Marcelli M, Mancini MA. Androgen receptor functional analyses by high throughput imaging: determination of ligand, cell cycle, and mutation-specific effects. PLoS One. 2008;3(11):e3605.
196. Teh BS, Paulino A, Butler EB. [Recent developments in radiation oncology-integrating radiation physics and molecular radiobiology advances into clinical radiotherapy practice and beyond]. Ai Zheng. 2008 Aug;27(8):885-93. Review. Chinese.
197. Tolan NV, Genes LI, Spence DM. Merging Microfluidics with Micro-titre Technology for More Efficient Drug Discovery. JALA Charlottesv Va. 2008 Oct;13(5):275-279.
198. Tomioka N, Oba S, Ohira M, Misra A, Fridlyand J, Ishii S, Nakamura Y, Isogai E, Hirata T, Yoshida Y, Todo S, Kaneko Y, Albertson DG, Pinkel D, Feuerstein BG, Nakagawara A. Novel risk stratification of patients with neuroblastoma by genomic signature, which is independent of molecular signature. Oncogene. 2008 Jan 17;27(4):441-9.
199. Torres-Roca JF, Stevens CW. Predicting response to clinical radiotherapy: past, present, and future directions. Cancer Control. 2008 Apr;15(2):151-6. Review.
200. Tsui NB. Multiple thiopurine S-methyltransferase variation detection: a step toward personalized medicine. Clin Chem. 2008 Oct;54(10):1598-9.
201. Tsujimoto G. [In-silico drug discovery science--research and education]. Yakugaku Zasshi. 2008 Nov;128(11):1519-23. Review. Japanese.
202. Usuda J, Hirata T, Ichinose S, Ishizumi T, Inoue T, Ohtani K, Maehara S, Yamada M, Tsutsui H, Okunaka T, Kato H, Ikeda N. Tailor-made approach to photodynamic therapy in the treatment of cancer based on Bcl-2 photodamage. Int J Oncol. 2008 Oct;33(4):689-96.
203. van't Veer LJ, Bernards R. Enabling personalized cancer medicine through analysis of gene-expression patterns. Nature. 2008 Apr 3;452(7187):564-70. Review. **NO ACCESS**
204. Vandenbroeck K, Matute C. Pharmacogenomics of the response to IFN-beta in multiple sclerosis: ramifications from the first genome-wide screen. Pharmacogenomics. 2008 May;9(5):639-45. Review. **NO ACCESS**
205. Velikyan I, Maecke H, Langstrom B. Convenient preparation of 68Ga-based PET-radiopharmaceuticals at room temperature. Bioconjug Chem. 2008 Feb;19(2):569-73. doi: 10.1021/bc700341x.
206. Vesely R. Breaking the code. When bill barring genetic discrimination is signed into law, 'fear of repercussions' will dissipate among patients: expert. Mod Healthc. 2008 May 12;38(19):6-7, 16, 1. **NO ACCCESS**
207. Villanueva A, Toffanin S, Llovet JM. Linking molecular classification of hepatocellular carcinoma and personalized medicine: preliminary steps. Curr Opin Oncol. 2008 Jul;20(4):444-53. Review.
208. Vladutiu GD. Genetic predisposition to statin myopathy. Curr Opin Rheumatol. 2008 Nov;20(6):648-55. Review.
209. Vladutiu GD. The FDA announces new drug labeling for pharmacogenetic testing: is personalized medicine becoming a reality? Mol Genet Metab. 2008 Jan;93(1):1-4.
210. Waldman SA, Terzic A. Clinical and translational sciences: at the intersection of molecular and individualized medicine. Clin Transl Sci. 2008 May;1(1):6-8. doi: 10.1111/j.1752-8062.2008.00019.x. **NO ACCCESS**
211. Waldman SA, Terzic A. Pharmacoeconomics in the era of individualized medicine. Clin Pharmacol Ther. 2008 Aug;84(2):179-82. doi: 10.1038/clpt.2008.142.
212. Waldman SA, Terzic A. The roadmap to personalized medicine. Clin Transl Sci. 2008 Sep;1(2):93.
213. Waldman SA, Terzic A. Therapeutic targeting: a crucible for individualized medicine. Clin Pharmacol Ther. 2008 May;83(5):651-4. doi: 10.1038/clpt.2008.65.
214. Walid MS, Donahue SN, Darmohray DM, Hyer LA Jr, Robinson JS Jr. The fifth vital sign--what does it mean? Pain Pract. 2008 Nov-Dec;8(6):417-22. doi: 10.1111/j.1533-2500.2008.00222.x. Epub 2008 Jul 25. Review. Retraction in: Pain Pract. 2009 May-Jun;9(3):245. **NO ACCCESS**
215. Wallis D, Russell HF, Muenke M. Review: Genetics of attention deficit/hyperactivity disorder. J Pediatr Psychol. 2008 Nov-Dec;33(10):1085-99. Epub 2008 Jun 3. Review.
216. Walsh C, Abelson HT. Medical professionalism: crossing a generational divide. Perspect Biol Med. 2008 Autumn;51(4):554-64.
217. Wiggs JL. Genomic promise: personalized medicine for ophthalmology. Arch Ophthalmol. 2008 Mar;126(3):422-3. **NO ACCESS**
218. Wijnen PA, Drent M, Nelemans PJ, Kuijpers PM, Koek GH, Neef C, Haenen GR, Bekers O. Role of cytochrome P450 polymorphisms in the development of pulmonary drug toxicity: a case-control study in the Netherlands. Drug Saf. 2008;31(12):1125-34. doi: 10.2165/0002018-200831120-00008.
219. Wilke RA, Berg RL, Linneman JG, Zhao C, McCarty CA, Krauss RM. Characterization of low-density lipoprotein cholesterol-lowering efficacy for atorvastatin in a population-based DNA biorepository. Basic Clin Pharmacol Toxicol. 2008 Oct;103(4):354-9.
220. Wu AC, Fuhlbrigge AL. Economic evaluation of pharmacogenetic tests. Clin Pharmacol Ther. 2008 Aug;84(2):272-4.
221. Yan Q. The integration of personalized and systems medicine: bioinformatics support for pharmacogenomics and drug discovery. Methods Mol Biol. 2008;448:1-19. doi: 10.1007/978-1-59745-205-2_1. Review. **NO ACCCESS**
222. Yang JY, Yang MQ, Arabnia HR, Deng Y. Genomics, molecular imaging, bioinformatics, and bio-nano-info integration are synergistic components of translational medicine and personalized healthcare research. BMC Genomics. 2008 Sep 16;9 Suppl 2:I1. **NO ACCESS**
223. Yang JY, Yang MQ, Zhu MM, Arabnia HR, Deng Y. Promoting synergistic research and education in genomics and bioinformatics. BMC Genomics. 2008;9 Suppl 1:I1. Review. **NO ACCESS**
224. Yeatman TJ, Mule J, Dalton WS, Sullivan D. On the eve of personalized medicine in oncology. Cancer Res. 2008 Sep 15;68(18):7250-2.
225. Yuan X, Bagga S, Shen J, Balakrishnan M, Benhaddou D. DS-MAC: differential service medium access control design for wireless medical information systems. Conf Proc IEEE Eng Med Biol Soc. 2008;2008:1801-4. **NO ACCESS**
226. Zeng X, Rao MS. Controlled genetic modification of stem cells for developing drug discovery tools and novel therapeutic applications. Curr Opin Mol Ther. 2008 Jun;10(3):207-13. Review. **NO ACCESS**
227. Zhang W, Huang RS, Dolan ME. Integrating Epigenomics into Pharmacogenomic Studies. Pharmgenomics Pers Med. 2008 Nov;2008(1):7-14.
228. Zhang W, Ratain MJ, Dolan ME. The HapMap Resource is Providing New Insights into Ourselves and its Application to Pharmacogenomics. Bioinform Biol Insights. 2008;2:15-23.
229. Zhou SF, Di YM, Chan E, Du YM, Chow VD, Xue CC, Lai X, Wang JC, Li CG, Tian M, Duan W. Clinical pharmacogenetics and potential application in personalized medicine. Curr Drug Metab. 2008 Oct;9(8):738-84. Review. **NO ACCESS**
230. Zhou X, Liu S, Kim ES, Herbst RS, Lee JJ. Bayesian adaptive design for targeted therapy development in lung cancer--a step toward personalized medicine. Clin Trials. 2008;5(3):181-93. **NO ACCESS**

**2007**

1. Agrawal S, Khan F. Human genetic variation and personalized medicine. Indian J Physiol Pharmacol. 2007 Jan-Mar;51(1):7-28. Review.
2. Ahn SM, Simpson RJ. Body fluid proteomics: Prospects for biomarker discovery. Proteomics Clin Appl. 2007 Sep;1(9):1004-15. doi: 10.1002/prca.200700217. **NO ACCESS**
3. Ala-Korpela M. Potential role of body fluid 1H NMR metabonomics as a prognostic and diagnostic tool. Expert Rev Mol Diagn. 2007 Nov;7(6):761-73. Review. **NO ACCESS**
4. Anderson JL, Horne BD, Stevens SM, Grove AS, Barton S, Nicholas ZP, Kahn SF, May HT, Samuelson KM, Muhlestein JB, Carlquist JF; Couma-Gen Investigators. Randomized trial of genotype-guided versus standard warfarin dosing in patients initiating oral anticoagulation. Circulation. 2007 Nov 27;116(22):2563-70.
5. Aspinall MG, Hamermesh RG. Realizing the promise of personalized medicine. Harv Bus Rev. 2007 Oct;85(10):108-17, 165. **NO ACCESS**
6. Asslaber M, Zatloukal K. Biobanks: transnational, European and global networks. Brief Funct Genomic Proteomic. 2007 Sep;6(3):193-201.
7. Balyasnikova IV, Skirgello OE, Binevski PV, Nesterovitch AB, Albrecht RF 2nd, Kost OA, Danilov SM. Monoclonal Antibodies 1G12 and 6A12 to the N-domain of human angiotensin-converting enzyme: fine epitope mapping and antibody-based detection of ACE inhibitors in human blood. J Proteome Res. 2007 Apr;6(4):1580-94.
8. Batsis JA, Nieto-Martinez RE, Lopez-Jimenez F. Metabolic syndrome: from global epidemiology to individualized medicine. Clin Pharmacol Ther. 2007 Nov;82(5):509-24. Epub 2007 Sep 12. Review.
9. Bauer JC. An early warning: the -omics data and personalized medicine are coming. J Healthc Inf Manag. 2007 Summer;21(3):6-7. **NO ACCESS**
10. Bawa R. PharmaMedDevice 2007. Issues in nanodrug delivery and personalized medicine. IDrugs. 2007 Jul;10(7):455-8. **NO ACCESS**
11. Beaton B. Racial science now: histories of race and science in the age of personalized medicine. Public Hist. 2007 Summer;29(3):157-62. **NO ACCESS**
12. Bensalah K, Montorsi F, Shariat SF. Challenges of cancer biomarker profiling. Eur Urol. 2007 Dec;52(6):1601-9. Epub 2007 Oct 1. Review. **NO ACCESS**
13. Black JL 3rd, O'Kane DJ, Mrazek DA. The impact of CYP allelic variation on antidepressant metabolism: a review. Expert Opin Drug Metab Toxicol. 2007 Feb;3(1):21-31. Review.
14. Bottinger EP. Foundations, promises and uncertainties of personalized medicine. Mt Sinai J Med. 2007 Apr;74(1):15-21. Review.
15. Brennan DJ, Kelly C, Rexhepaj E, Dervan PA, Duffy MJ, Gallagher WM. Contribution of DNA and tissue microarray technology to the identification and validation of biomarkers and personalised medicine in breast cancer. Cancer Genomics Proteomics. 2007 May-Jun;4(3):121-34. Review.
16. Brown MP, Buckley MF, Rudzki Z, Olver IN. Why we will need to learn new skills to control cancer. Intern Med J. 2007 Mar;37(3):201-4. Review.
17. Burke W, Psaty BM. Personalized medicine in the era of genomics. JAMA. 2007 Oct 10;298(14):1682-4. **NO ACCESS**
18. Cai W, Chen X. Nanoplatforms for targeted molecular imaging in living subjects. Small. 2007 Nov;3(11):1840-54. Review. **NO ACCESS**
19. Campos-Outcalt D. Personalized medicine: the promise, the reality. J Fam Pract. 2007 Aug;56(8):621-6.
20. Cardoso F, Piccart-Gebhart M, Van't Veer L, Rutgers E; TRANSBIG Consortium. The MINDACT trial: the first prospective clinical validation of a genomic tool. Mol Oncol. 2007 Dec;1(3):246-51.
21. Caruthers SD, Wickline SA, Lanza GM. Nanotechnological applications in medicine. Curr Opin Biotechnol. 2007 Feb;18(1):26-30. Epub 2007 Jan 24.
22. Cortese DA. A vision of individualized medicine in the context of global health. Clin Pharmacol Ther. 2007 Nov;82(5):491-3. **NO ACCESS**
23. Daly AK. Individualized drug therapy. Curr Opin Drug Discov Devel. 2007 Jan;10(1):29-36. Review. **NO ACCESS**
24. DeGeorge BR Jr, Koch WJ. Beta blocker specificity: a building block toward personalized medicine. J Clin Invest. 2007 Jan;117(1):86-9.
25. Dickinson AM. Risk assessment in haematopoietic stem cell transplantation: pre-transplant patient and donor factors: non-HLA genetics. Best Pract Res Clin Haematol. 2007 Jun;20(2):189-207. Review. **NO ACCESS**
26. Dietel M. Predictive pathology of cytostatic drug resistance and new anti-cancer targets. Recent Results Cancer Res. 2007;176:25-32. **NO ACCESS**
27. Etheredge LM. A rapid-learning health system. Health Aff (Millwood). 2007 Mar-Apr;26(2):w107-18. Epub 2007 Jan 26. Review.
28. Evans BJ. Distinguishing product and practice regulation in personalized medicine. Clin Pharmacol Ther. 2007 Feb;81(2):288-93. Review.
29. Evans BJ. Finding a liability-free space in which personalized medicine can bloom. Clin Pharmacol Ther. 2007 Oct;82(4):461-5. Epub 2007 Aug 22. Review.
30. Ferrara J. Personalized medicine: challenging pharmaceutical and diagnostic company business models. Mcgill J Med. 2007 Jan;10(1):59-61.
31. Foster A, Wang Z, Usman M, Stirewalt E, Buckley P. Pharmacogenetics of antipsychotic adverse effects: Case studies and a literature review for clinicians. Neuropsychiatr Dis Treat. 2007 Dec;3(6):965-73.
32. Fox JL. Despite glacial progress, US government signals support for personalized medicine. Nat Biotechnol. 2007 May;25(5):489-90.
33. Fukudo M. [Individualized dosage regimen of immunosuppressive drugs based on pharmacokinetic and pharmacodynamic analysis]. Yakugaku Zasshi. 2007 Jul;127(7):1081-9. Review. Japanese.
34. Furge KA, Dykema K, Petillo D, Westphal M, Zhang Z, Kort EJ, Teh BT. Combining differential expression, chromosomal and pathway analyses for the molecular characterization of renal cell carcinoma. Can Urol Assoc J. 2007 Jun;1(2 Suppl):S21-7.
35. Furge LL, Fletke KJ. HPLC determination of caffeine and paraxanthine in urine: An assay for cytochrome P450 1A2 activity. Biochem Mol Biol Educ. 2007 Mar;35(2):138-44. doi: 10.1002/bmb.28. **NO ACCESS**
36. Goldknopf IL. Molecular diagnostics and proteomics on the outskirts of personalized medicine. Expert Rev Mol Diagn. 2007 Jul;7(4):339-43. **NO ACCESS**
37. Goyette P, Labbé C, Trinh TT, Xavier RJ, Rioux JD. Molecular pathogenesis of inflammatory bowel disease: genotypes, phenotypes and personalized medicine. Ann Med. 2007;39(3):177-99. Review.
38. Heidecker B, Hare JM. The use of transcriptomic biomarkers for personalized medicine. Heart Fail Rev. 2007 Mar;12(1):1-11. Epub 2007 Mar 28. Review.
39. Hewitt SM, Takikita M, Braunschweig T, Chung JY. Promises and challenges of predictive tissue biomarkers. Biomark Med. 2007 Aug;1(2):313-8.
40. Hirano T. Cellular pharmacodynamics of immunosuppressive drugs for individualized medicine. Int Immunopharmacol. 2007 Jan;7(1):3-22. Epub 2006 Oct 23. Review. **NO ACCESS**
41. Hsu DS, Balakumaran BS, Acharya CR, Vlahovic V, Walters KS, Garman K, Anders C, Riedel RF, Lancaster J, Harpole D, Dressman HK, Nevins JR, Febbo PG, Potti A. Pharmacogenomic strategies provide a rational approach to the treatment of cisplatin-resistant patients with advanced cancer. J Clin Oncol. 2007 Oct 1;25(28):4350-7. Erratum in: J Clin Oncol. 2010 Jun 1;28(16):2805. Retraction in: J Clin Oncol. 2010 Dec 10;28(35):5229.
42. Issa AM. Personalized medicine and the practice of medicine in the 21st century. Mcgill J Med. 2007 Jan;10(1):53-7.
43. Iyengar SK. The quest for genes causing complex traits in ocular medicine: successes, interpretations, and challenges. Arch Ophthalmol. 2007 Jan;125(1):11-8. Review. PubMed PMID: 17210847.
44. Jørgensen JT, Nielsen KV, Ejlertsen B. Pharmacodiagnostics and targeted therapies - a rational approach for individualizing medical anticancer therapy in breast cancer. Oncologist. 2007 Apr;12(4):397-405. Review.
45. Jain KK. Applications of nanobiotechnology in clinical diagnostics. Clin Chem. 2007 Nov;53(11):2002-9. Epub 2007 Sep 21. Review.
46. Jain KK. Cancer biomarkers: current issues and future directions. Curr Opin Mol Ther. 2007 Dec;9(6):563-71. Review. **NO ACCESS**
47. June CH. Adoptive T cell therapy for cancer in the clinic. J Clin Invest. 2007 Jun;117(6):1466-76. Review.
48. Kahler SC. Out of Rockville and Laurel. FDA-CVM initiatives span individualized medicine to aquaculture research. J Am Vet Med Assoc. 2007 Sep 15;231(6):824-6. **NO ACCESS**
49. Käller M, Lundeberg J, Ahmadian A. Arrayed identification of DNA signatures. Expert Rev Mol Diagn. 2007 Jan;7(1):65-76. Review. **NO ACCESS**
50. Kalow W. Personalized medicine: some thoughts. Mcgill J Med. 2007 Jan;10(1):58.
51. Kalyanasundaram A, Gerhard GS, Skelding KA. Genomics, haplotypes and cardiovascular disease. Future Cardiol. 2007 Nov;3(6):601-10. **NO ACCESS**
52. Kao JH. Appropriate use of interferon for treatment of chronic hepatitis B. Hepatol Res. 2007 Jul;37(s1):S47-54. **NO ACCESS**
53. Kaput J, Noble J, Hatipoglu B, Kohrs K, Dawson K, Bartholomew A. Application of nutrigenomic concepts to Type 2 diabetes mellitus. Nutr Metab Cardiovasc Dis. 2007 Feb;17(2):89-103. Epub 2007 Feb 1. Review.
54. Kato T, Kamoto S, Hyuga M, Karube I. SNPs typing based on the formation of fluorescent signaling DNA aptamers which bind to bile acids. Nucleic Acids Symp Ser (Oxf). 2007;(51):97-8.
55. Katoh M, Katoh M. STAT3-induced WNT5A signaling loop in embryonic stem cells, adult normal tissues, chronic persistent inflammation, rheumatoid arthritis and cancer (Review). Int J Mol Med. 2007 Feb;19(2):273-8.
56. Katoh M. Dysregulation of stem cell signaling network due to germline mutation, SNP, Helicobacter pylori infection, epigenetic change and genetic alteration in gastric cancer. Cancer Biol Ther. 2007 Jun;6(6):832-9. Epub 2007 Mar 26. Review. **NO ACCESS**
57. Khosravi-Darani K, Pardakhty A, Honarpisheh H, Rao VS, Mozafari MR. The role of high-resolution imaging in the evaluation of nanosystems for bioactive encapsulation and targeted nanotherapy. Micron. 2007;38(8):804-18. Epub 2007 Jul 3. Review.
58. Khushf G. Upstream ethics in nanomedicine: a call for research. Nanomedicine (Lond). 2007 Aug;2(4):511-21. Review. **NO ACCESS**
59. Kling J. Diagnosis or drug? Will pharmaceutical companies or diagnostics manufacturers earn more from personalized medicine? EMBO Rep. 2007 Oct;8(10):903-6.
60. Kong W, Choo KW. Predicting single nucleotide polymorphisms (SNP) from DNA sequence by support vector machine. Front Biosci. 2007 Jan 1;12:1610-4. **NO ACCESS**
61. Krynetskiy E, McDonnell P. Building individualized medicine: prevention of adverse reactions to warfarin therapy. J Pharmacol Exp Ther. 2007 Aug;322(2):427-34.
62. Kucherlapati R. Raju Kucherlapati talks about personalised medicine: breathing new life into old drugs. Interview by Steve Carney. Drug Discov Today. 2007 Apr;12(7-8):272-5. **NO ACCESS**
63. Kurdziel KA, Kalen JD, Hirsch JI, Wilson JD, Agarwal R, Barrett D, Bear HD, McCumiskey JF. Imaging multidrug resistance with 4-[18F]fluoropaclitaxel. Nucl Med Biol. 2007 Oct;34(7):823-31. Epub 2007 Jul 5. Review. **NO ACCESS**
64. L'Heureux N, Dusserre N, Marini A, Garrido S, de la Fuente L, McAllister T. Technology insight: the evolution of tissue-engineered vascular grafts--from research to clinical practice. Nat Clin Pract Cardiovasc Med. 2007 Jul;4(7):389-95. Review.
65. Lacaná E, Amur S, Mummanneni P, Zhao H, Frueh FW. The emerging role of pharmacogenomics in biologics. Clin Pharmacol Ther. 2007 Oct;82(4):466-71. Epub 2007 Aug 22. Review.
66. Lalouel JM, Rohrwasser A. Genetic susceptibility to essential hypertension: insight from angiotensinogen. Hypertension. 2007 Mar;49(3):597-603.
67. Lawler P, Miao W, Williams P. From the editor's desk: the emergence of "personalized medicine" in medical care. Mcgill J Med. 2007 Jan;10(1):1-2.
68. Lee SS. The ethical implications of stratifying by race in pharmacogenomics. Clin Pharmacol Ther. 2007 Jan;81(1):122-5.
69. Lesko LJ. Personalized medicine: elusive dream or imminent reality? Clin Pharmacol Ther. 2007 Jun;81(6):807-16.
70. Li SS. Commentary--the proteomics: a new tool for Chinese medicine research. Am J Chin Med. 2007;35(6):923-8. **NO ACCESS**
71. Lin JH. Pharmacokinetic and pharmacodynamic variability: a daunting challenge in drug therapy. Curr Drug Metab. 2007 Feb;8(2):109-36. Review.
72. Lippi G, Franchini M, Montagnana M, Guidi GC. Genomics and proteomics in venous thromboembolism: building a bridge toward a rational personalized medicine framework. Semin Thromb Hemost. 2007 Nov;33(8):759-70. Review. **NO ACCESS**
73. Llerena A, Michel G, Jeannesson E, Wong S, Manolopoulos VG, Hockett RD, Boubekeur K, Siest G, Beaune P, Haefliger C, Arnold HP, Junien C, Petrovic N, Molloy R, Bekers O, Donnelly C, Arens HJ, Kaput J, McComb J. Third Santorini conference pharmacogenomics workshop report: "Pharmacogenomics at the crossroads: what else than good science will be needed for the field to become part of Personalized Medicine?". Clin Chem Lab Med. 2007;45(7):843-50.
74. Manne U. Understanding racial differences in colorectal cancer aids in individualized medicine. Future Oncol. 2007 Jun;3(3):235-41. Review. **NO ACCESS**
75. Marko-Varga G, Ogiwara A, Nishimura T, Kawamura T, Fujii K, Kawakami T, Kyono Y, Tu HK, Anyoji H, Kanazawa M, Akimoto S, Hirano T, Tsuboi M, Nishio K, Hada S, Jiang H, Fukuoka M, Nakata K, Nishiwaki Y, Kunito H, Peers IS, Harbron CG, South MC, Higenbottam T, Nyberg F, Kudoh S, Kato H. Personalized medicine and proteomics: lessons from non-small cell lung cancer. J Proteome Res. 2007 Aug;6(8):2925-35.
76. Markward NJ. Quantifying genomic variation at the individual level: putting the "person" in personalized medicine. AMIA Annu Symp Proc. 2007 Oct 11:1041. **NO ACCESS**
77. Martino TA, Tata N, Bjarnason GA, Straume M, Sole MJ. Diurnal protein expression in blood revealed by high throughput mass spectrometry proteomics and implications for translational medicine and body time of day. Am J Physiol Regul Integr Comp Physiol. 2007 Sep;293(3):R1430-7.
78. Mason CE, Seringhaus MR, Sattler de Sousa e Brito C. Personalized genomic medicine with a patchwork, partially owned genome. Yale J Biol Med. 2007 Dec;80(4):145-51.
79. Massoud TF, Gambhir SS. Integrating noninvasive molecular imaging into molecular medicine: an evolving paradigm. Trends Mol Med. 2007 May;13(5):183-91. Epub 2007 Apr 2. Review.
80. McCarty CA, Nair A, Austin DM, Giampietro PF. Informed consent and subject motivation to participate in a large, population-based genomics study: the Marshfield Clinic Personalized Medicine Research Project. Community Genet. 2007;10(1):2-9.
81. McKinnon RA, Ward MB, Sorich MJ. A critical analysis of barriers to the clinical implementation of pharmacogenomics. Ther Clin Risk Manag. 2007 Oct;3(5):751-9.
82. Menke MN, Strauss JF 3rd. Genetic approaches to polycystic ovarian syndrome. Curr Opin Obstet Gynecol. 2007 Aug;19(4):355-9. Review.
83. Miller GA. Therapeutic success via targeted approaches and strategic partnerships with clinical laboratories. IDrugs. 2007 Mar;10(3):181-4. Review. **NO ACCESS**
84. Mohr S, Liew CC. The peripheral-blood transcriptome: new insights into disease and risk assessment. Trends Mol Med. 2007 Oct;13(10):422-32. Epub 2007 Oct 4. Review. **NO ACCESS**
85. Moon H, Ahn H, Kodell RL, Baek S, Lin CJ, Chen JJ. Ensemble methods for classification of patients for personalized medicine with high-dimensional data. Artif Intell Med. 2007 Nov;41(3):197-207.
86. Morrow KJ Jr, Bawa R, Wei C. Recent advances in basic and clinical nanomedicine. Med Clin North Am. 2007 Sep;91(5):805-43. Review. **NO ACCESS**
87. Nakai K, Tsuboi J, Okabayashi H, Fukuhiro Y, Oka T, Habano W, Fukushima N, Nakai K, Obara W, Fujioka T, Suwabe A, Gurwitz D. Ethnic differences in the VKORC1 gene polymorphism and an association with warfarin dosage requirements in cardiovascular surgery patients. Pharmacogenomics. 2007 Jul;8(7):713-9. **NO ACCESS**
88. Nakamura N, Ito K, Hongo S, Hashimoto K, Furutsuka M, Kubota R, Fukuda T, Ohno M, Azuma J, Gemma N. Determination of single nucleotide polymorphisms in N-acetyltransferase2 gene using an electrochemical DNA chip and an automated DNA detection system. Rinsho Byori. 2007 Mar;55(3):216-23. **NO ACCESS**
89. Nakamura N, Ito K, Takahashi M, Hashimoto K, Kawamoto M, Yamanaka M, Taniguchi A, Kamatani N, Gemma N. Detection of six single-nucleotide polymorphisms associated with rheumatoid arthritis by a loop-mediated isothermal amplification method and an electrochemical DNA chip. Anal Chem. 2007 Dec 15;79(24):9484-93. **NO ACCESS**
90. Niss BJ, Aufses AH Jr. The journal: a retrospective. Mt Sinai J Med. 2007 Apr;74(1):2-6.
91. Nomura S, Kondo M, Nagano M, Matsui K, Egashira T. Development of a novel nano-Invader DNA chip system. J Biochem Biophys Methods. 2007 Aug 1;70(5):787-95.
92. Nunn AD. Molecular imaging and personalized medicine: an uncertain future. Cancer Biother Radiopharm. 2007 Dec;22(6):722-39.
93. Nutt R, Vento LJ, Ridinger MH. In vivo molecular imaging biomarkers: clinical pharmacology's new "PET"? Clin Pharmacol Ther. 2007 Jun;81(6):792-5.
94. Ouzounian M, Lee DS, Gramolini AO, Emili A, Fukuoka M, Liu PP. Predict prevent and personalize: Genomic and proteomic approaches to cardiovascular medicine. Can J Cardiol. 2007 Aug;23 Suppl A:28A-33A. Review.
95. Patrick K. 454 life sciences: illuminating the future of genome sequencing and personalized medicine. Yale J Biol Med. 2007 Dec;80(4):191-4.
96. Perl DP. The role of the pathologist in translational and personalized medicine. Mt Sinai J Med. 2007 Apr;74(1):22-6.
97. Pham TD. Predictive modeling in proteomics-based disease detection. Conf Proc IEEE Eng Med Biol Soc. 2007;2007:3308-11. **NO ACCESS**
98. Phillips E, Mallal S. Drug hypersensitivity in HIV. Curr Opin Allergy Clin Immunol. 2007 Aug;7(4):324-30. Review. **NO ACCESS**
99. Piquette-Miller M, Grant DM. The art and science of personalized medicine. Clin Pharmacol Ther. 2007 Mar;81(3):311-5.
100. Po AL. Personalised medicine: who is an Asian? Lancet. 2007 May 26;369(9575):1770-1.
101. Rackover MA. Embracing the new world of personalized medicine. JAAPA. 2007 May;20(5):12. **NO ACCESS**
102. Raich T, Irwin J. State and federal legislation in personalized medicine.MLO Med Lab Obs. 2007 Aug;39(8):48. **NO ACCESS**
103. Rao M. Scalable human ES culture for therapeutic use: propagation, differentiation, genetic modification and regulatory issues. Gene Ther. 2008 Jan;15(2):82-8. **NO ACCESS**
104. Ratain MJ. Personalized medicine: building the GPS to take us there. Clin Pharmacol Ther. 2007 Mar;81(3):321-2.
105. Razvi ES. The second annual Burrill personalized medicine conference. IDrugs. 2007 Jan;10(1):26-9. **NO ACCESS**
106. Reed GH, Kent JO, Wittwer CT. High-resolution DNA melting analysis for simple and efficient molecular diagnostics. Pharmacogenomics. 2007 Jun;8(6):597-608. Review.
107. Reitman ML, Schadt EE. Pharmacogenetics of metformin response: a step in the path toward personalized medicine. J Clin Invest. 2007 May;117(5):1226-9. Review.
108. Rimm D. Dr. David Rimm is interviewed by Feras Akbik. Yale J Biol Med. 2007 Dec;80(4):183-5.
109. Robson B. The new physician as unwitting quantum mechanic: is adapting Dirac's inference system best practice for personalized medicine, genomics, and proteomics? J Proteome Res. 2007 Aug;6(8):3114-26. Epub 2007 Jul 3. Erratum in: J Proteome Res. 2007 Aug;6(8):3126.
110. Roessler S, Budhu A, Wang XW. Future of molecular profiling of human hepatocellular carcinoma. Future Oncol. 2007 Aug;3(4):429-39. Review.
111. Roses A. "Personalized medicine: elusive dream or imminent reality?": A commentary. Clin Pharmacol Ther. 2007 Jun;81(6):801-5.
112. Rowe DL. Off-label prescription of quetiapine in psychiatric disorders. Expert Rev Neurother. 2007 Jul;7(7):841-52. Review.
113. Ruden DM. Personalized medicine and quantitative trait transcripts. Nat Genet. 2007 Feb;39(2):144-5.
114. Sahab ZJ, Semaan SM, Sang QX. Methodology and applications of disease biomarker identification in human serum. Biomark Insights. 2007 Feb 14;2:21-43.
115. Sakariassen KS. Blood flow devices in medical research and clinical testing in humans: are we approaching personalized medicine? Future Cardiol. 2007 Jan;3(1):71-90. **NO ACCESS**
116. Schmidt C. Newly revealed genome complexity may mean personalized medicine is farther away. J Natl Cancer Inst. 2007 Nov 7;99(21):1568-70.
117. Schnackenberg LK. Global metabolic profiling and its role in systems biology to advance personalized medicine in the 21st century. Expert Rev Mol Diagn. 2007 May;7(3):247-59. Review.
118. Segal E, Sirlin CB, Ooi C, Adler AS, Gollub J, Chen X, Chan BK, Matcuk GR, Barry CT, Chang HY, Kuo MD. Decoding global gene expression programs in liver cancer by noninvasive imaging. Nat Biotechnol. 2007 Jun;25(6):675-80.
119. Seigneuric R, Starmans MH, Fung G, Krishnapuram B, Nuyten DS, van Erk A, Magagnin MG, Rouschop KM, Krishnan S, Rao RB, Evelo CT, Begg AC, Wouters BG, Lambin P. Impact of supervised gene signatures of early hypoxia on patient survival. Radiother Oncol. 2007 Jun;83(3):374-82.
120. Siest G, Marteau JB, Maumus S, Berrahmoune H, Jeannesson E, Samara A, Batt AM, Visvikis-Siest S. [Pharmacogenomics and pharmacoproteomics: a strategy for cardio-vascular drugs]. Ann Pharm Fr. 2007 May;65(3):203-10. Review. French.
121. Sikora K. Personalized medicine for cancer: from molecular signature to therapeutic choice. Adv Cancer Res. 2007;96:345-69. Review.
122. Sioud M, Melien O. Treatment options and individualized medicine. Methods Mol Biol. 2007;361:327-40. Review. **NO ACCESS**
123. Sjöqvist F, Eliasson E. The convergence of conventional therapeutic drug monitoring and pharmacogenetic testing in personalized medicine: focus on antidepressants. Clin Pharmacol Ther. 2007 Jun;81(6):899-902. Epub 2007 Mar 28.
124. Solomon LM, Sieczkiewicz GJ. Impact of the US Patent System on the promise of personalized medicine. Gend Med. 2007 Sep;4(3):187-92. Review. **NO ACCESS**
125. Strecher V. Internet methods for delivering behavioral and health-related interventions (eHealth). Annu Rev Clin Psychol. 2007;3:53-76. Review. **NO ACCESS**
126. Subbiah MT. Nutrigenetics and nutraceuticals: the next wave riding on personalized medicine. Transl Res. 2007 Feb;149(2):55-61. Review.
127. Swen JJ, Huizinga TW, Gelderblom H, de Vries EG, Assendelft WJ, Kirchheiner J, Guchelaar HJ. Translating pharmacogenomics: challenges on the road to the clinic. PLoS Med. 2007 Aug;4(8):e209. Review.
128. Taegtmeyer AB. Personalized medicine. Mcgill J Med. 2007 Jan;10(1):51-2.
129. Taniguchi A, Urano W, Tanaka E, Kamatani N. [Pharmacogenomics of antirheumatic drugs and personalized medicine for rheumatoid arthritis]. Nippon Rinsho. 2007 Feb;65(2):371-9. Review. Japanese.
130. Taylor DL. Past, present, and future of high content screening and the field of cellomics. Methods Mol Biol. 2007;356:3-18. **NO ACCESS**
131. Tegnér J, Skogsberg J, Björkegren J. Thematic review series: systems biology approaches to metabolic and cardiovascular disorders. Multi-organ whole-genome measurements and reverse engineering to uncover gene networks underlying complex traits. J Lipid Res. 2007 Feb;48(2):267-77. Epub 2006 Dec 1. Review. PubMed PMID: 17142807.
132. Torigian DA, Huang SS, Houseni M, Alavi A. Functional imaging of cancer with emphasis on molecular techniques. CA Cancer J Clin. 2007 Jul-Aug;57(4):206-24. Review. Erratum in: CA Cancer J Clin. 2007 Nov-Dec;57(6):380.
133. Trevino V, Falciani F, Barrera-Saldaña HA. DNA microarrays: a powerful genomic tool for biomedical and clinical research. Mol Med. 2007 Sep-Oct;13(9-10):527-41. Review.
134. Triggle DJ. Drug discovery and delivery in the 21st century. Med Princ Pract. 2007;16(1):1-14. Review.
135. Tse KP, Tsang NM, Chen KD, Li HP, Liang Y, Hsueh C, Chang KP, Yu JS, Hao SP, Hsieh LL, Chang YS. MCP-1 Promoter Polymorphism at 2518 is associated with metastasis of nasopharyngeal carcinoma after treatment. Clin Cancer Res. 2007 Nov 1;13(21):6320-6.
136. Turner ST, Schwartz GL, Boerwinkle E. Personalized medicine for high blood pressure. Hypertension. 2007 Jul;50(1):1-5. Epub 2007 Apr 30. Review.
137. Valenti WM. Companion diagnostic tests in HIV medicine: the road to personalized medicine. AIDS Read. 2007 Nov;17(11):546-9. **NO ACCESS**
138. van der Greef J, Martin S, Juhasz P, Adourian A, Plasterer T, Verheij ER, McBurney RN. The art and practice of systems biology in medicine: mapping patterns of relationships. J Proteome Res. 2007 Apr;6(4):1540-59. Epub 2007 Mar 21.
139. Vasto S, Candore G, Duro G, Lio D, Grimaldi MP, Caruso C. Alzheimer's disease and genetics of inflammation: a pharmacogenomic vision. Pharmacogenomics. 2007 Dec;8(12):1735-45. Review. **NO ACCESS**
140. Vizirianakis IS. Clinical translation of genotyping and haplotyping data: implementation of in vivo pharmacology experience leading drug prescription to pharmacotyping. Clin Pharmacokinet. 2007;46(10):807-24. Review.
141. Waldman SA, Terzic A. Individualized medicine and the imperative of global health. Clin Pharmacol Ther. 2007 Nov;82(5):479-83. **NO ACCESS**
142. Wang SJ, O'Neill RT, Hung HM. Approaches to evaluation of treatment effect in randomized clinical trials with genomic subset. Pharm Stat. 2007 Jul-Sep;6(3):227-44.
143. Weljie AM, Dowlatabadi R, Miller BJ, Vogel HJ, Jirik FR. An inflammatory arthritis-associated metabolite biomarker pattern revealed by 1HNMR spectroscopy. J Proteome Res. 2007 Sep;6(9):3456-64. **NO ACCESS**
144. West CM, Elliott RM, Burnet NG. The genomics revolution and radiotherapy. Clin Oncol (R Coll Radiol). 2007 Aug;19(6):470-80. Epub 2007 Apr 6. Review. **NO ACCESS**
145. Whippen D, Deering MJ, Ambinder EP. Advancing high-quality cancer care: cancer biomedical informatics grid supports personalized medicine and the electronic health record. J Oncol Pract. 2007 Jul;3(4):208-11.
146. Wilke RA, Berg RL, Peissig P, Kitchner T, Sijercic B, McCarty CA, McCarty DJ. Use of an electronic medical record for the identification of research subjects with diabetes mellitus. Clin Med Res. 2007 Mar;5(1):1-7.
147. Witherspoon DJ, Wooding S, Rogers AR, Marchani EE, Watkins WS, Batzer MA, Jorde LB. Genetic similarities within and between human populations. Genetics. 2007 May;176(1):351-9. Epub 2007 Mar 4.
148. Wolinsky H. The thousand-dollar genome. Genetic brinkmanship or personalized medicine? EMBO Rep. 2007 Oct;8(10):900-3.
149. Wolters SL, Corsten MF, Reutelingsperger CP, Narula J, Hofstra L. Cardiovascular molecular imaging of apoptosis. Eur J Nucl Med Mol Imaging. 2007 Jun;34 Suppl 1:S86-98. Review.
150. Wong SH. Pharmacogenomics and personalized medicine--a global reality check. Clin Chem Lab Med. 2007;45(7):799-800. **NO ACCESS**
151. Woodcock J. The prospects for "personalized medicine" in drug development and drug therapy. Clin Pharmacol Ther. 2007 Feb;81(2):164-9. Review.
152. Wortham M, Czerwinski M, He L, Parkinson A, Wan YJ. Expression of constitutive androstane receptor, hepatic nuclear factor 4 alpha, and P450 oxidoreductase genes determines interindividual variability in basal expression and activity of a broad scope of xenobiotic metabolism genes in the human liver. Drug Metab Dispos. 2007 Sep;35(9):1700-10.
153. Xu F. Effect of personality type on pharmacodynamics through changing pharmacokinetics. Med Hypotheses. 2007;69(5):1131-4. **NO ACCESS**
154. Xystrakis E, Urry Z, Hawrylowicz CM. Regulatory T cell therapy as individualized medicine for asthma and allergy. Curr Opin Allergy Clin Immunol. 2007 Dec;7(6):535-41. Review. **NO ACCESS**
155. Yokokawa R, Tamaoki S, Sakamoto T, Murakami A, Sugiyama S. Transcriptome analysis device based on liquid phase detection by fluorescently labeled nucleic acid probes. Biomed Microdevices. 2007 Dec;9(6):869-75. **NO ACCESS**
156. Zhang X. Biomarker validation: movement towards personalized medicine. Expert Rev Mol Diagn. 2007 Sep;7(5):469-71. **NO ACCESS**

**2006**

1. Agarwal R, Kaye SB. Expression profiling and individualisation of treatment for ovarian cancer. Curr Opin Pharmacol. 2006 Aug;6(4):345-9. Epub 2006 Jun 5. Review.
2. Agur Z. Biomathematics in the development of personalized medicine in oncology. Future Oncol. 2006 Feb;2(1):39-42.
3. Anderson JE, Hansen LL, Mooren FC, Post M, Hug H, Zuse A, Los M. Methods and biomarkers for the diagnosis and prognosis of cancer and other diseases: towards personalized medicine. Drug Resist Updat. 2006 Aug-Oct;9(4-5):198-210. Epub 2006 Oct 2. Review.
4. Arab S, Gramolini AO, Ping P, Kislinger T, Stanley B, van Eyk J, Ouzounian M, MacLennan DH, Emili A, Liu PP. Cardiovascular proteomics: tools to develop novel biomarkers and potential applications. J Am Coll Cardiol. 2006 Nov 7;48(9):1733-41. Epub 2006 Oct 17. Review.
5. Baba Y. [Diagnosis of diseases by nanodevice]. Nippon Rinsho. 2006 Feb;64(2):271-7. Review. Japanese.
6. Baba Y. [Nanotechnology in medicine]. Nippon Rinsho. 2006 Feb;64(2):189-98. Review. Japanese.
7. Batson BA. Reader's response and author's reply to "personalized medicine: finding the patient's 'doctor within'". MedGenMed. 2006;8(3):1; author reply 1.
8. Bersch C. Nutritional genomics: part of the evolution of personalized medicine. MLO Med Lab Obs. 2006 Jul;38(7):52.
9. Bissonnette L, Bergeron MG. Next revolution in the molecular theranostics of infectious diseases: microfabricated systems for personalized medicine. Expert Rev Mol Diagn. 2006 May;6(3):433-50. Review. **NO ACCESS**
10. Blum RS. Clinical pharmacology of the appeals and exception process: the interface of the patient, physician, and insurance company to assure appropriate care under Medicare Part D. J Clin Pharmacol. 2006 Apr;46(4):394-7.
11. Bogomolov VV, Voronkov IuI, Larina ON, Morgun VV, Kaspranskiĭ RR, Lazebnyĭ OE, Andrianov BV, Kulikov AM. [Identification of individual genotype parameters, informative for medical care in piloted space missions: modern and future capabilities]. Aviakosm Ekolog Med. 2006 Jan-Feb;40(1):22-7. Review. Russian. **NO ACCESS**
12. Brockhoff G. [Cytomics and receptor interaction]. Verh Dtsch Ges Pathol. 2006;90:31-8. German.
13. Broeckel U, Maresso K, Kugathasan S. Functional genomics and its implications for molecular medicine. Pediatr Clin North Am. 2006 Oct;53(5):807-16, vii. Review. **NO ACCESS**
14. Caldwell J. Drug metabolism and pharmacogenetics: the British contribution to fields of international significance. Br J Pharmacol. 2006 Jan;147 Suppl 1:S89-99. PubMed PMID: 16402125; PubMed Central PMCID: PMC1760745.
15. Caruthers SD, Winter PM, Wickline SA, Lanza GM. Targeted magnetic resonance imaging contrast agents. Methods Mol Med. 2006;124:387-400. Review.
16. Catts ZA, Radford CL. Cancer risk assessment and genetics at the Helen F. Graham Cancer Center: model for personalized medicine and early intervention. Del Med J. 2006 Sep;78(9):325-32. **NO ACCESS**
17. Centola M, Frank MB, Bolstad AI, Alex P, Szanto A, Zeher M, Hjelmervik TO, Jonsson R, Nakken B, Szegedi G, Szodoray P. Genome-scale assessment of molecular pathology in systemic autoimmune diseases using microarray technology: a potential breakthrough diagnostic and individualized therapy-design tool. Scand J Immunol. 2006 Sep;64(3):236-42. Review.
18. Chang HW, Yang CH, Chang PL, Cheng YH, Chuang LY. SNP-RFLPing: restriction enzyme mining for SNPs in genomes. BMC Genomics. 2006 Feb 17;7:30.
19. Chawapun N. Update on clinical radiobiology. Biomed Imaging Interv J. 2006 Jan;2(1):e22.
20. Collins CD, Purohit S, Podolsky RH, Zhao HS, Schatz D, Eckenrode SE, Yang P, Hopkins D, Muir A, Hoffman M, McIndoe RA, Rewers M, She JX. The application of genomic and proteomic technologies in predictive, preventive and personalized medicine. Vascul Pharmacol. 2006 Nov;45(5):258-67. Epub 2006 Aug 18. Review. **NO ACCESS**
21. Collins I, Workman P. New approaches to molecular cancer therapeutics. Nat Chem Biol. 2006 Dec;2(12):689-700. Review. Erratum in: Nat Chem Biol. 2007 Feb;3(2):126.
22. Danser AH, Batenburg WW, van den Meiracker AH, Danilov SM. ACE phenotyping as a first step toward personalized medicine for ACE inhibitors. Why does ACE genotyping not predict the therapeutic efficacy of ACE inhibition? Pharmacol Ther. 2007 Mar;113(3):607-18. Epub 2006 Dec 28. Review.
23. Davies SM. Pharmacogenetics, pharmacogenomics and personalized medicine: are we there yet? Hematology Am Soc Hematol Educ Program. 2006:111-7. Review.
24. de Leon J. AmpliChip CYP450 test: personalized medicine has arrived in psychiatry. Expert Rev Mol Diagn. 2006 May;6(3):277-86. Review. **NO ACCESS**
25. Di Bartolo N, Sargeson AM, Smith SV. New 64Cu PET imaging agents for personalised medicine and drug development using the hexa-aza cage, SarAr. Org Biomol Chem. 2006 Sep 7;4(17):3350-7.
26. Diagnostic tests for HIV resistance are created as quickly as the new drugs. Research is leading individualized medicine evolution. AIDS Alert. 2006 Sep;21(9):97-9. **NO ACCESS**
[truncated: 51,739 more chars]
